# Modulating mycobacterial envelope integrity for antibiotic synergy with benzothiazoles

**DOI:** 10.26508/lsa.202302509

**Published:** 2024-05-14

**Authors:** Eva Habjan, Alexander Lepioshkin, Vicky Charitou, Anna Egorova, Elena Kazakova, Vien QT Ho, Wilbert Bitter, Vadim Makarov, Alexander Speer

**Affiliations:** 1 Department of Medical Microbiology and Infection Prevention, Amsterdam UMC, Location VU Medical Center , Amsterdam, Netherlands; 2 Federal Research Centre “Fundamentals of Biotechnology” of the Russian Academy of Sciences (Research Centre of Biotechnology RAS), Moscow, Russia

## Abstract

Developing effective tuberculosis drugs is hindered by mycobacteria’s intrinsic antibiotic resistance because of their impermeable cell envelope. Using benzothiazole compounds, we aimed to increase mycobacterial cell envelope permeability and weaken the defenses of *Mycobacterium marinum*, serving as a model for *Mycobacterium tuberculosis*. Initial hit, BT-08, significantly boosted ethidium bromide uptake, indicating enhanced membrane permeability. It also demonstrated efficacy in the *M. marinum*–zebrafish embryo infection model and *M. tuberculosis*-infected macrophages. Notably, BT-08 synergized with established antibiotics, including vancomycin and rifampicin. Subsequent medicinal chemistry optimization led to BT-37, a non-toxic and more potent derivative, also enhancing ethidium bromide uptake and maintaining synergy with rifampicin in infected zebrafish embryos. Mutants of *M. marinum* resistant to BT-37 revealed that MMAR_0407 (Rv0164) is the molecular target and that this target plays a role in the observed synergy and permeability. This study introduces novel compounds targeting a new mycobacterial vulnerability and highlights their cooperative and synergistic interactions with existing antibiotics.

## Abstract (precis)

This study explores benzothiazoles targeting a novel mycobacterial vulnerability, resulting in increased mycobacterial cell envelope permeability and enhanced antibiotic activity.

## Introduction

Tuberculosis (TB), caused by *Mycobacterium tuberculosis*, remains the deadliest bacterial infectious disease in the world (World Health Organization, 2022a). The current treatment for active drug-sensitive TB consists of six months with at least four-drug cocktail therapy (World Health Organization, 2022a). The rise of multi-drug–resistant TB strains requires the use of second-line agents, which may ultimately result in the emergence of extensively drug-resistant TB (World Health Organization, 2020). Lately, several new therapeutic strategies have been investigated, and improved treatment regimens have been reported (World Health Organization, 2022b). Nevertheless, there is a high demand for new drugs and treatment regimens to combat this life-threatening pathogen.

One of the challenges in developing new effective anti-mycobacterial agents is their delivery across the highly impermeable cell envelope to allow access to intracellular targets. Although mycobacteria are genetically classified as Gram-positive bacteria, their cell envelope has a unique structure that includes an outer membrane known as mycomembrane (Hoffmann et al, 2008; Marchand et al, 2012; Dulberger et al, 2020). This mycomembrane comprises several unique lipids, including the long-chain fatty acids known as mycolic acids (Hoffmann et al, 2008). Mycolic acids and some of the other lipids render the mycomembrane highly impermeable (Jackson, 2014). This barrier protects mycobacteria from the hostile environment during infection and prevents antibiotics from reaching their intracellular target (Benedetto Tiz et al, 2018). Therefore, the permeabilization of the mycobacterial cell envelope displays an intriguing strategy to facilitate antibiotic uptake and potentially improve their activity.

The low permeability of the cell envelope makes the commonly used target-to-drug approach largely unsuccessful for mycobacteria. Even though small molecules designed to combat mycobacteria can show high potency on purified enzymes, they are often unsuccessful in whole-cell assays (Abrahams & Besra, 2020) because of insufficient compound uptake (Lechartier et al, 2014; Benedetto Tiz et al, 2018). Therefore, a drug-to-target approach has proven to be more successful, where phenotypic drug screens first reveal active compounds, and the identification of a drug target is addressed later (Grzelak et al, 2019; Abrahams & Besra, 2020). Because of the increase in antibiotic-resistant strains, it is crucial that novel compounds act on previously unexplored targets. However, even an unbiased drug-to-target approach is no guarantee of identifying compounds with new mechanisms of action since recent drug screens identified compounds with different chemical scaffolds acting on the same targets, such as DprE1 and MmpL3 (Degiacomi et al, 2020).

The treatment regimen for tuberculosis should preferably consist of several therapeutics targeting different essential pathways (Dartois & Rubin, 2022). This approach makes it challenging for the pathogen to become resistant. An additional consideration is that drugs combined in the treatment regimen should have favorable drug-to-drug interactions (Dartois & Rubin, 2022). Ideally, the drugs should have a synergistic effect, where the combined effect of drugs is greater than the sum of each drug’s effect alone (Greco et al, 1996). As such, a lower dosage of every single drug needs to be administrated, thus decreasing the chances of drug-associated toxicity and side effects (Greco et al, 1996; Foucquier & Guedj, 2015). For concentration-dependent antibiotics, the activity of synergistic compounds could also result in shorter therapy, a holy grail in modern TB drug development. Therefore, identifying novel anti-microbial small molecules that can synergize with the currently used drugs is an important approach that is often overlooked.

Benzothiazoles are compounds containing a benzene ring fused with a thiazole ring (Yadav et al, 2011). They exhibit diverse biological activities, encompassing anti-microbial, anti-tumor, and anti-inflammatory effects (Sharma et al, 2013). Besides their activity, they have excellent pharmacological potential, which is highly desirable during the drug optimization process (Sharma et al, 2013). Our study aimed to explore whether the mycobacterial outer membrane could be permeabilized with new benzothiazole-core compounds and render the bacteria more susceptible to antibiotics. We screened a targeted in-house benzothiazole-scaffold compound library using an ethidium bromide uptake assay in *Mycobacterium marinum*, a model organism for *M. tuberculosis*. Compound **BT-08** synergized with antibiotics and showed activity in the *M. marinum*–zebrafish and *M. tuberculosis*–macrophage infection models. We optimized the compound and identified a new essential drug target: protein MMAR_0407 (Rv0164).

## Results

### Cell wall permeability screening identifies benzothiazole BT-08

**Figure 1.**
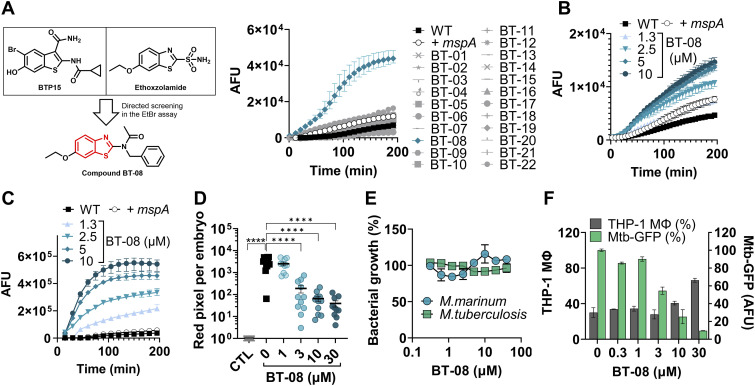
Screening for membrane permeabilizing agents identifies BT-08 as a hit compound with in vivo and ex vivo anti-mycobacterial activity. **(A)** Chemical structure of BTP15 and ethoxzolamide served as a starting point to generate a benzothiazole compound library, from which BT-08 was the most promising hit. WT *M. marinum* cultures were grown (7H9 medium with albumin–dextrose–saline [ADS] and tyloxapol) in the presence of test compounds (10 μM) before the EtBr uptake assay was performed. A WT *M. marinum* strain expressing the porin *mspA* served as a positive control (+*mspA*). After the addition of EtBr, the fluorescence intensity (arbitrary fluorescence units) was measured for 3 h. Data are presented as the mean of triplicates ± range. **(B)** Dose-dependent activity of BT-08 on *M. marinum* during the ethidium bromide uptake assay (7H9 medium with ADS and tyloxapol). Data are presented as the mean of triplicates ± range. **(C)** Dose-dependent activity of BT-08 on *M. marinum* during the resazurin uptake assay (7H9 medium with ADS and tyloxapol). Data are presented as the mean of triplicates ± range. **(D)** Dose-dependent activity of BT-08 in the zebrafish embryo infection model. Embryos were yolk-infected with *M. marinum* expressing *tdTomato*, and treatment was performed by immersion. Each data point represents the integrated red fluorescence intensity of a single zebrafish embryo, and the signal of each group is expressed as the mean ± SD of the mean. Statistical significance was determined by one-way ANOVA, following Dunnett’s multiple comparison test by comparing the signal from the DMSO-treated control sample with each treatment group (****P ≤ 0.0001). CTL represents the non-infected group. **(E)** Effect of BT-08 on bacterial growth of *M. marinum* M^USA^ and *M. tuberculosis* H37Rv was assessed using the resazurin reduction microtiter plate assay. DMSO-treated sample represents 100% bacterial growth. Data are presented as the mean of duplicates ± range. **(F)** Intracellular activity of BT-08 in THP-1 macrophages infected with *M. tuberculosis* expressing *gfp*. The expression of *gfp* was induced by the addition of ATc. To detect macrophages (gray bars), the nuclei were stained with Hoechst dye. The GFP signal within each macrophage was quantified, representing the amount of viable bacteria (green bars). DMSO- and rifampicin (10 μM)-treated samples served as a negative and positive control, respectively. Data are presented as the mean of duplicates ± range.

It has recently been identified that BTP15 and ethoxzolamide (Fig 1A) could reduce mycobacterial virulence by affecting the ESX-1 secretion system (Rybniker et al, 2014; Johnson et al, 2015). Exploiting their structural similarities, we synthesized a set of benzothiazole derivatives (Table S1) to explore the possibility of developing active compounds based on this scaffold. These compounds could improve the permeability of the mycobacterial cell envelope, ultimately enhancing the effectiveness of traditional antibiotics. The model organism *M. marinum* was used for an ethidium bromide (EtBr) uptake assay, assessing membrane permeability. EtBr enters the cell and binds to bacterial DNA, resulting in higher fluorescence, indicating increased cell wall permeability (Rodrigues et al, 2008). As a control strain, we employed *M. marinum* overproducing MspA (*+mspA*), a porin known to increase outer membrane permeability (Niederweis et al, 1999; Stahl et al, 2001). *M. marinum* expressing *mspA* showed, as expected, higher uptake of EtBr compared with the WT strain (Fig 1A). Likewise, some of the WT *M. marinum* cultures incubated with the test compounds showed higher EtBr uptake compared with the non-treated WT strain (Fig 1A, Table S1). Compound **BT-08** was the most prominent, showing a more than 26-fold increase in the fluorescent signal compared with the non-treated strain (Fig 1A). Note that none of the tested compounds inhibited the growth of *M. marinum* at the tested concentration of 10 μM, as determined by optical density (OD_600_) measurements in 7H9 medium supplemented with ADS (albumin–dextrose–saline) and tyloxapol. To confirm these results, we repeated the EtBr uptake assay using various concentrations of **BT-08** (Fig 1B). The increase in EtBr uptake was concentration-dependent, and even the lowest tested concentration of compound **BT-08** (1.3 μM) showed a higher signal as compared to the non-treated WT strain, whereas the maximal activity of **BT-08** was seen at 10 μM.

Table S1. Results of the targeted screening for benzothiazoles in the ethidium bromide (EtBr) uptake assay and an *M. marinum*-infected zebrafish model (Mmar-ZF).

### BT-08 increases mycobacterial cell envelope permeability in a dose-dependent manner

**Figure S1.**
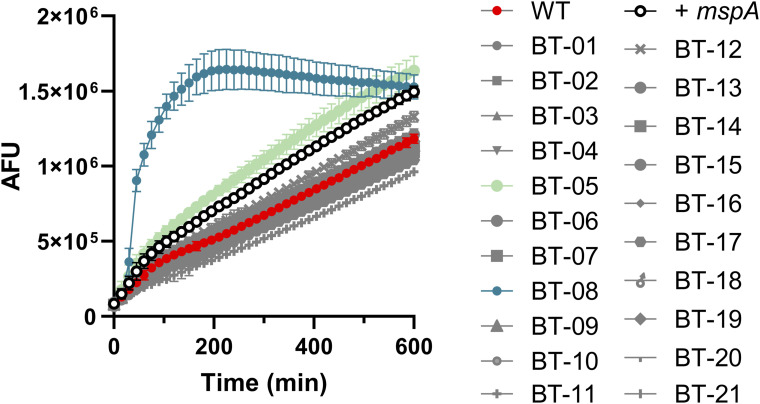
Compound BT-08 increases resazurin uptake. WT *M. marinum* cultures were grown (7H9 medium with albumin–dextrose–saline and tyloxapol) in the presence of test compounds (10 μM) before the resazurin uptake assay was performed. A WT *M. marinum* strain expressing the porin *mspA* served as a positive control (+*mspA*). After the addition of resazurin sodium salt mixed with Tween-80, the fluorescence intensity (arbitrary fluorescence units) was measured for 10 h. Data are presented as the mean of triplicates ± SD.

To study the effects of compound **BT-08** in more detail, we adapted the previously described resazurin-based microtiter plate assay (REMA) to investigate the transport of the dye resazurin across the mycobacterial cell wall (Palomino et al, 2002). *M. marinum* was cultured in the presence of several concentrations of **BT-08** and afterward transferred into a 96-well plate before the resazurin dye was added. The dye resazurin is imported into the cells and reduced during aerobic respiration into a fluorescent product resorufin (Palomino et al, 2002). Generally, the fluorescent signal of resorufin can be detected after 6–12 h of incubation with slow-growing mycobacteria. However, when *M. marinum* was grown in the presence of **BT-08**, we already detected the resorufin signal after 1 h of incubation. This effect followed a dose–response and was stronger with increasing concentrations of **BT-08** over time (Fig 1C). Next, we evaluated our set of 21 benzothiazole-based compounds in the same assay, which demonstrated that compound **BT-08** shows the strongest phenotype (Fig S1), which is in line with the data of the EtBr assay (Fig 1). In addition, compound **BT-05** showed a higher signal compared with the positive control strain (+*mspA*). Our results indicate that both EtBr and resazurin are able to access the mycobacterial cell much more efficiently when these cells are cultured in the presence of compound **BT-08**.

### BT-08 exhibits anti-mycobacterial activity ex vivo and in vivo

To investigate the effect of the benzothiazoles on mycobacteria during infection, we used the previously described *M. marinum*–zebrafish embryo infection model (Habjan et al, 2021). Zebrafish embryos were infected via yolk injection with *M. marinum*, expressing a red fluorescent protein (*tdTomato*). 1 d post-infection, the infected embryos were incubated with 10 μM of the test compound for 3 d. The efficacy of the treatment was evaluated by measuring the red fluorescent signal, which corresponds to the mycobacterial burden within the infected zebrafish embryos (Stoop et al, 2011). When we tested our initial set, only compound **BT-08** showed a statistically significant (*P* < 0.0001) reduction in mycobacterial load in the zebrafish, as compared to the DMSO-treated control group of embryos (Table S1). In a follow-up experiment, compound **BT-08** showed a dose-dependent efficacy in the *M. marinum*–zebrafish infection model, significantly reducing the bacterial burden starting at 3 μM (*P* < 0.0001) (Fig 1D). It is of particular interest that only compound BT-08 demonstrated activity in EtBr and resazurin uptake assays, as well as efficacy in zebrafish infection studies. This suggests a potential correlation between increased membrane permeability and observed activity in the zebrafish model. Although not extensively diverse, our library (**BT-01–BT-22**) was comprised of closely related analogs to compound BT-08. However, other than **BT-08**, none showed significant activity during in vitro and in vivo assays (Figs 1A and S1, Table S1). This observation underscores high specificity and selectivity required for the compound’s activity. Because **BT-08** was the only compound displaying in vivo efficacy and caused the strongest phenotype during the EtBr and resazurin uptake assays, we decided to further focus on **BT-08**.

As mentioned previously, **BT-08** had no effect on the bacterial growth of *M. marinum* in culture (Fig 1E), which stands in contrast with the in vivo results. We hypothesized that the effect of the compound on the bacterial cell wall is only critical in vivo, where the innate immune system of the zebrafish is an additional anti-bacterial factor. Next, we investigated whether this discrepancy between in vitro and in vivo activity translates to clinically relevant mycobacterial species *M. tuberculosis*. Likewise, the compound **BT-08** did not inhibit the *M. tuberculosis* H37Rv growth in culture (Fig 1E), whereas we could demonstrate a dose-dependent reduction in intracellular *M. tuberculosis* when we treated *M. tuberculosis*-infected THP-1 macrophages. Treatment with 30 μM of **BT-08** partially protected THP-1 macrophages from bacterial-induced lysis (Fig 1F).

Compound **BT-08** did not inhibit the in vitro growth of other tested fast-growing mycobacteria and some G+ and G- species (Table S2). Thus, the effect of compound **BT-08** could only be observed during host infection experiments, and it seems specific to mycobacteria, although we cannot rule out the possibility that other species are sensitive to **BT-08** during infection. Because we hypothesized that **BT-08** acts as a membrane-perturbing agent, we further explored the compound’s safety profile. **BT-08** did not show toxicity toward zebrafish embryos, murine-derived macrophage cell line RAW 264.7, or human-derived monocyte THP-1 cell line during cytotoxicity experiments and infection studies (Table S3). In addition, **BT-08** was not hemolytic toward sheep red blood cells (Table S4), hence demonstrating specificity toward slow-growing mycobacterial cells.

Table S2. Activity of BT-08 against selected microorganisms.

Table S3. Toxicity of BT-08 and BT-37 in cytotoxicity assays and zebrafish embryotoxicity assay.

Table S4. Hemolytic activity of compounds BT-08 and BT-37.

### BT-08 in vitro anti-microbial activity is detergent- and media-dependent

**Figure S2.**
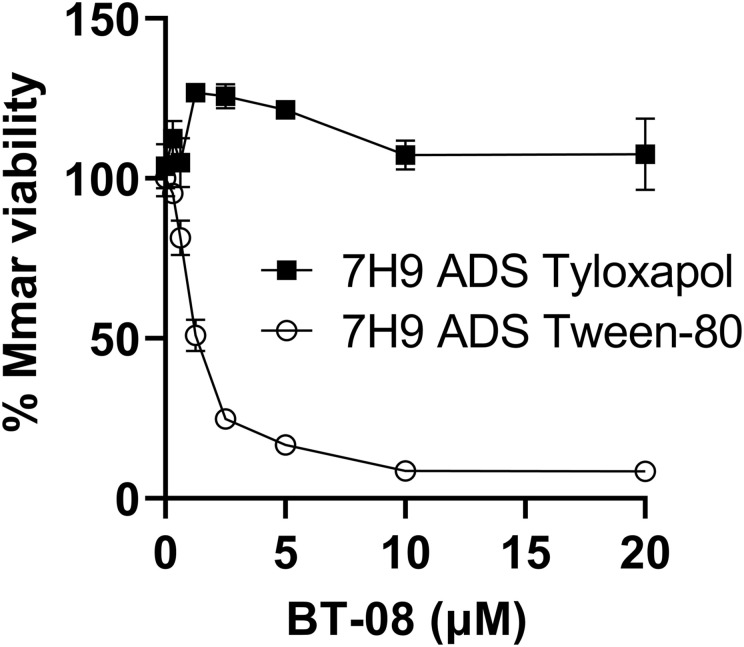
Detergents used in growth media affect the susceptibility of M. marinum to BT-08. Susceptibility of *M. marinum* toward BT-08 in 7H9 medium supplemented with albumin–dextrose–saline and different detergents: Tween-80 or tyloxapol, using the resazurin microtiter plate assay. Data are presented as the mean of duplicates ± SD.

**Figure S3.**
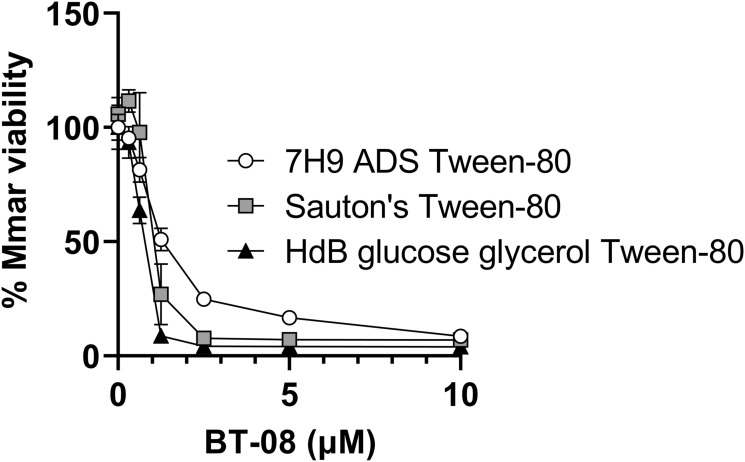
Different growth media affect susceptibility of M. marinum to BT-08. Susceptibility of *M. marinum* toward BT-08 in different growth media using the resazurin microtiter plate assay. Data are presented as the mean of duplicates ± SD.

Even though we observed the activity of **BT-08** during infection studies, we were puzzled by the fact that it showed no in vitro activity by itself. All in vitro experiments up to this point were performed in 7H9 medium supplemented with ADS and the detergent tyloxapol. Although this is a rich standard medium for slow-growing mycobacteria, we wondered whether a defined minimal medium with different detergents would affect the bacteria differently because the medium-dependent activity of anti-microbial compounds has been reported previously (Pethe et al, 2010; Franzblau et al, 2012). First, we replaced the detergent tyloxapol with Tween-80. Interestingly, compound **BT-08** prevented bacterial growth of *M. marinum* in the 7H9 medium supplemented with ADS and Tween-80 (Fig S2). We next compared the standard 7H9 medium supplemented with ADS and Tween-80 with Sauton’s minimal medium supplemented with Tween-80 and Hartman’s de Bond (HdB) medium supplemented with Tween-80 (Fig S3). We observed that **BT-08** is most active against *M. marinum* in the HdB medium with Tween-80 (Fig S3). Consequently, we continued the in vitro experiments using the HdB medium and Tween-80.

### BT-08 potentiates the activity of high molecular weight antibiotics

**Figure S4.**
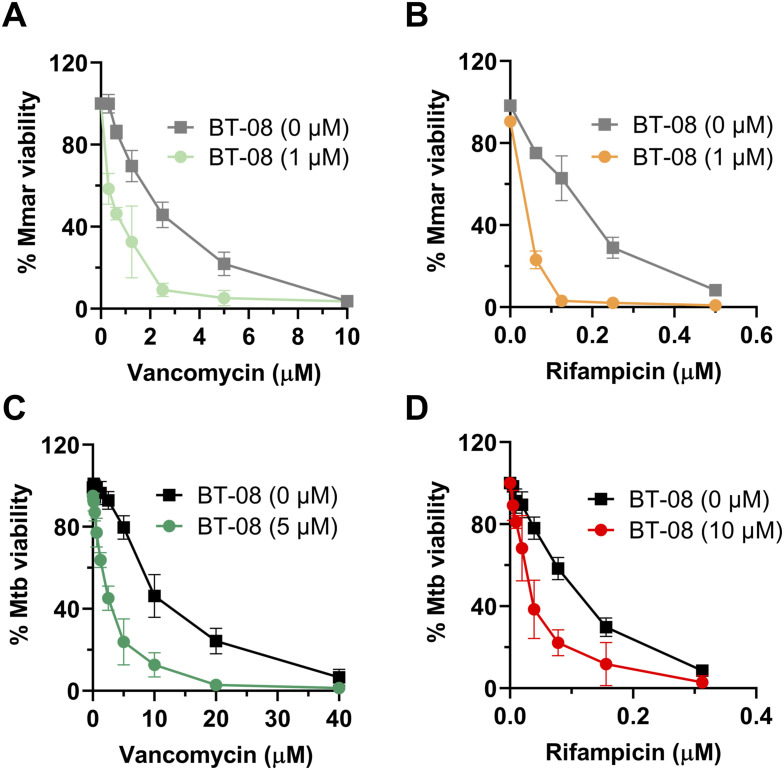
Compound BT-08 increases activity of rifampicin (RIF) and vancomycin in vitro. **(A, B, C, D)** Sensitivity of *M. marinum* toward the combinatorial treatment of compound BT-08 with vancomycin (A) or RIF, (B, C, D) and the sensitivity of *M. tuberculosis* toward the combinatorial treatment of compound BT-08 with vancomycin (C) or RIF (D) using the in vitro checkerboard assay and 7H9 media supplemented with albumin–dextrose–saline and tyloxapol. Data are presented as the mean of duplicates ± SD.

Because compound **BT-08** facilitates the uptake of EtBr and resazurin dye into *M. marinum* cells, we investigated whether this effect extends to antibiotics. To clarify this, we selected two high molecular weight antibiotics, rifampicin (RIF) and vancomycin, as alterations in the mycobacterial cell envelope can enhance the susceptibility of mycobacteria to these two antibiotics (Healy et al, 2020). Moreover, the targets of RIF and vancomycin have distinct locations in the cytoplasm or periplasm, respectively. Drug-to-drug interactions in vitro are generally reported as the fractional inhibitory concentration (FIC) index (FICI). A FICI below 0.5 indicates synergy, whereas the FICI value between 0.5 and 1 represents an additive effect. First, we investigated the effect of **BT-08** on the activity of RIF or vancomycin using checkerboard assays and 7H9 medium supplemented with tyloxapol, because **BT-08** increased uptake of EtBr and resazurin in this medium. The addition of **BT-08** resulted in a marked change in *M. marinum* sensitivity to the two antibiotics; for both vancomycin (Fig S4A) and RIF (Fig S4B), the MIC_90_ was improved fourfold compared with the single-drug treatment. The same assay was used for *M. tuberculosis* H37Rv, where the shift for vancomycin was fourfold (Fig S4C), and for RIF, twofold (Fig S4D) when **BT-08** was added. Because compound **BT-08** alone does not inhibit the growth of *M. marinum* or *M. tuberculosis* in this media, the calculation of the FICI value was not possible. Thus, we repeated experiments using the HdB medium with Tween-80. **BT-08** did not show sufficient activity in the HdB medium with Tween-80 in *M. tuberculosis*; therefore, we could not determine the FIC index. However, for *M. marinum*, the calculated FIC index for **BT-08** and vancomycin was 0.38, and for **BT-08** and RIF, 0.5, indicative of synergistic interactions for both tested drug combinations (Table S5). Thus, the data suggest that compound **BT-08** also facilitates the uptake of RIF and vancomycin inside the cell, increasing their activity.

Table S5. Assessment of drug combinations of BT-08 and different antibiotics.

**Figure 2.**
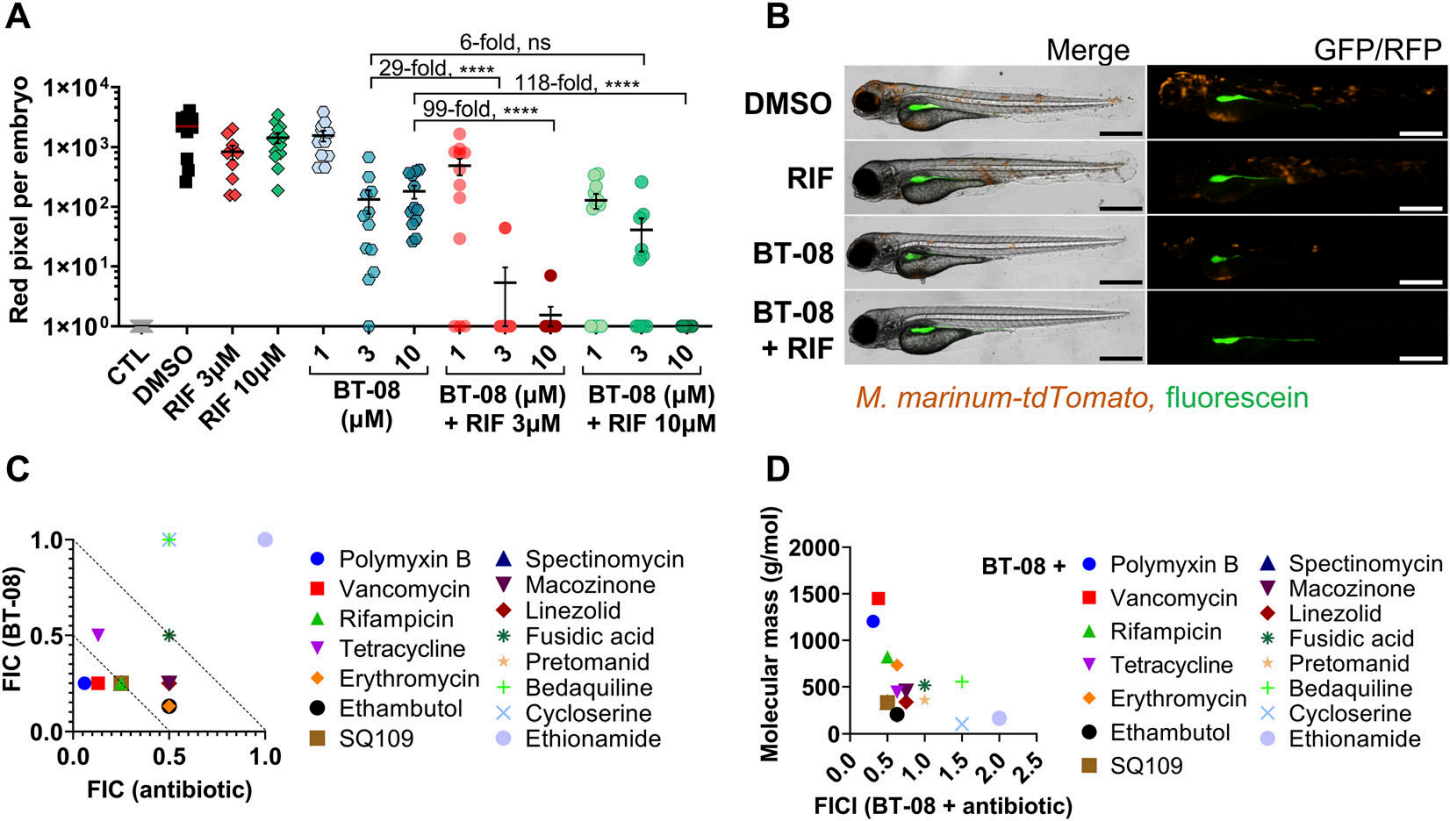
Compound BT-08 and high molecular weight antibiotics act synergistically in vivo and in vitro. **(A)** Activity of rifampicin (RIF) and BT-08 and their combinations in the zebrafish embryo infection model. Zebrafish embryos were yolk-infected with *M. marinum* expressing *tdTomato*, and treatment was performed by immersion. Each data point represents the integrated red fluorescence intensity of a single zebrafish embryo, and the signal of each group is expressed as the mean ± SD of the mean. CTL represents the non-infected group. The fold difference between the means of different treatment groups is depicted. Statistical significance was determined by one-way ANOVA, following Tukey’s multiple comparison test by comparing each treatment group with the rest (****P ≤ 0.0001). CTL represents the non-infected group. **(B)** Representative images of *M. marinum* yolk-infected zebrafish embryos treated with DMSO, BT-08 at 3 μM, RIF at 3 μM, or their combination (BT-08 at 3 μM and RIF at 3 μM) at 4 d post-fertilization. The red color corresponds to a signal from *M. marinum* expressing *tdTomato*, and the green color corresponds to the injection control dye fluorescein. The scale bar represents 500 μm. **(C)** Drug combinations between BT-08 and selected antibiotics reported as the fractional inhibitory concentration (FIC) and the FIC index (FICI) were investigated in *M. marinum* using a checkerboard assay using the HdB medium with Tween-80. **(D)** FICI of BT-08 and selected antibiotics calculated using a checkerboard assay in *M. marinum* (HdB medium with Tween-80) is plotted against the molecular weight (g/mol) of tested antibiotics. The correlation was evaluated using Spearman’s one-tailed correlation test, showing significance of *P* = 0.0315.

Next, we investigated whether we could confirm a similar synergistic effect in vivo using *M. marinum*-infected zebrafish embryos. Indeed, the combination of RIF and **BT-08** was over 100-fold more active than the single agent at the same concentration (Fig 2A and B). This remarkable in vivo synergy was confirmed using various concentrations of both agents (Fig 2A). Inspired by these findings, we then tested several antibiotics, varying in their molecular weight, mechanism of action, and activity against mycobacteria (Fig 2C, Table S5). Interestingly, several combinations tested in *M. marinum* in vitro were shown to be effective, with a FICI value indicating synergistic or additive interactions (Fig 2C, Table S5). Compounds with molecular weights above 800*g*/mol, such as RIF, vancomycin, and polymyxin B, showed synergistic interactions, whereas compounds with a molecular weight below or close to 800*g*/mol showed additive effects (Fig 2C, Table S5). Notably, the compounds with the lowest molecular weight from our test set showed no interaction with **BT-08**. The correlation between the synergistic effect and a higher molecular weight of tested antibiotics was significant (*P = 0.0315) (Fig 2D) and suggests improved delivery of antibiotics inside the cells, which is typically a bottleneck for bulky compounds.

### BT-08 optimization resulted in benzothiazole BT-37 with improved activity against *M. tuberculosis*

To optimize the compound **BT-08** further, we explored the chemical space of the benzothiazoles (Table S6). All synthesized derivatives were screened for their activity against *M. marinum* in the *M. marinum*-infected zebrafish model. In earlier experiments, we observed that with REMA in the HdB medium with Tween-80, we can observe and evaluate growth inhibition of compound **BT-08** (Fig S3). Thus, we evaluated compounds from the benzothiazole library for their in vitro activity using the HdB medium with Tween-80 by REMA (Table S6).

Table S6. Exploration results of the benzothiazoles’ six-position and side phenyl ring testing on *M. marinum* in vitro (MIC assay—REMA) and in the *M. marinum*-infected zebrafish model (Mmar-ZF).

**Figure 3.**
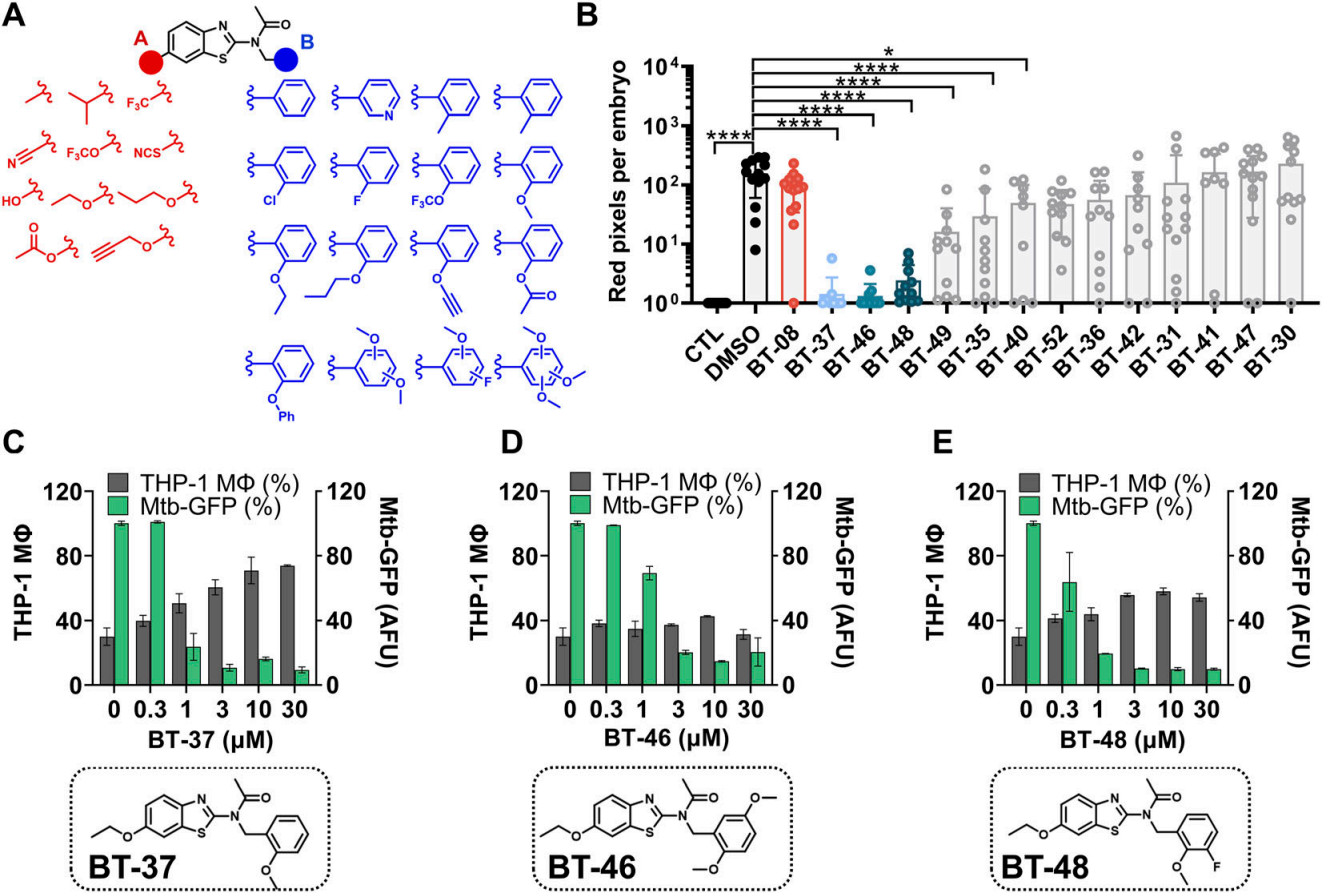
Structure–activity relationship studies revealed the most active derivative BT-37. **(A)** Set of benzothiazole derivatives for structure–activity relationship studies. **(B)** Activity of benzothiazole derivatives at 1 μM in the zebrafish–*M. marinum* infection model. Embryos were yolk-infected with *M. marinum* expressing *tdTomato* and treated with 1 μM of each derivative by immersion. Each data point represents the integrated red fluorescence intensity of a single zebrafish embryo, and the signal of each group is expressed as the mean ± SD of the mean. Statistical significance was determined by one-way ANOVA, following Dunnett’s multiple comparison test by comparing the signal from the DMSO-treated control sample with each treatment group (****P ≤ 0.0001; *P ≤ 0.05). CTL represents the non-infected group. **(C, D, E)** Activity and chemical structures of BT-37, (D) BT-46, and (E) BT-48 in the macrophage infection model. THP-1 macrophages were infected with *M. tuberculosis* expressing *gfp*, induced by adding ATc. Macrophages (gray bars) were detected by staining the nuclei with Hoechst dye. The GFP signal within each macrophage was quantified, representing the amount of viable bacteria (green bars). DMSO- and RIF (10 μM)-treated samples served as a negative and positive control, respectively. Data are presented as the mean of duplicates ± range.

We mainly investigated two parts of the **BT-08** benzothiazole-core molecule—the six-position of the benzothiazole cycle and the substitution in the side benzyl group, named “A” and “B,” respectively (Fig 3A). The introduction of hydroxy- (**BT-25**), as well as methyl (**BT-27**), substituents at the six-position led to the toxicity of compounds in the zebrafish infection model. Only compounds with linear alkyl chains, **BT-30** and **BT-31**, were active in the zebrafish model and demonstrated acceptable MIC_90_ values against *M. marinum*, whereas other compounds with different functional substituents were inactive. For the next SAR evaluation, we decided to keep the 6-ethoxy group. We observed that adding an alkyl chain to the two-position of the phenyl ring (**BT-38** and **BT-39**) resulted in a complete loss of potency in both assays. On the contrary, **BT-40** with a prop-2-yn-1-yloxy substituent showed activity in the zebrafish model. We also found that introducing a fluorine atom at the two-position (**BT-42**) resulted in activity in both assays. However, when introducing a chlorine atom (**BT-41**), we noticed a loss of activity in the zebrafish model. Of the synthesized derivatives, only **BT-37** with a 2-methoxy group demonstrated good activity in both models. The addition of two methoxy groups (**BT-46** and **BT-47**), as well as a methoxy group and fluorine atom (**BT-48** and **BT-49**), resulted in the acceptable activity, with **BT-46** and **BT-48** being the most interesting. However, introducing three methoxy groups led to a complete activity loss in both models, surprisingly, except for **BT-52**.

**Figure S5.**
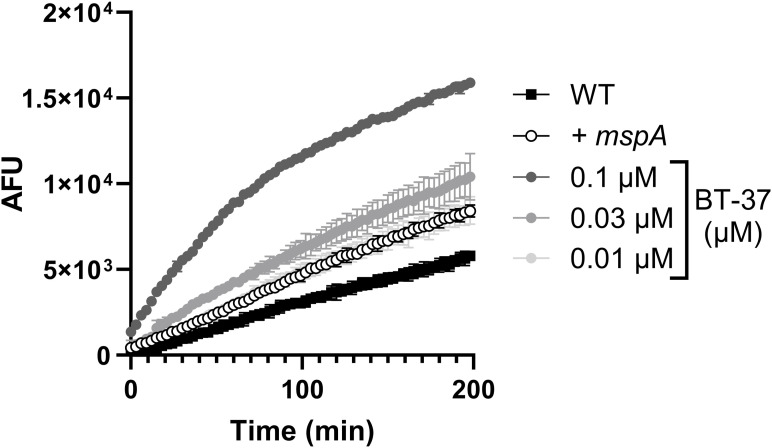
Treatment of M. marinum with BT-37 increases ethidium bromide (EtBr) uptake. The *M. marinum* strain was grown in the HdB medium with Tween-80 and with different concentrations of BT-37. *M. marinum* WT expressing the porin *mspA* was used as a positive control (+*mspA*). After adding EtBr, the fluorescence intensity (arbitrary fluorescence units) was measured for 3 h. Data are presented as the mean of triplicates ± SD.

**Figure S6.**
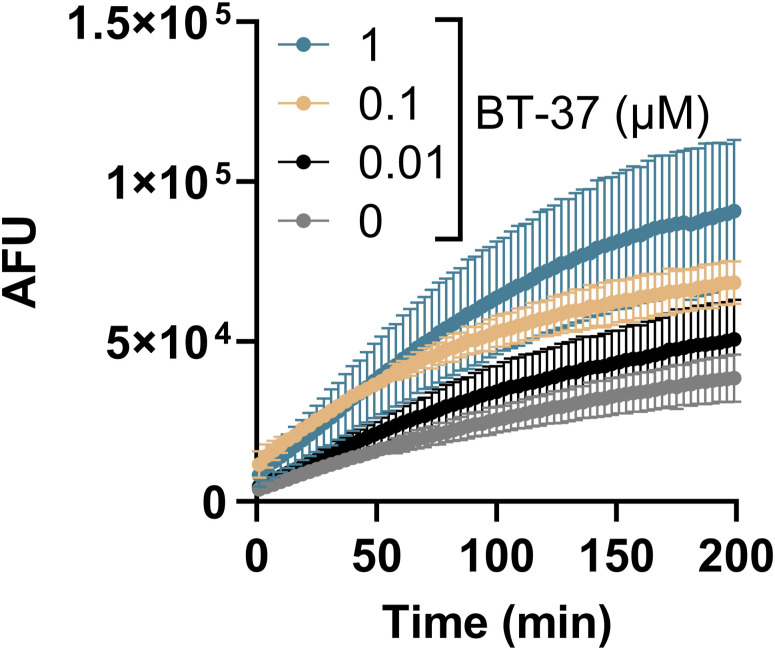
Treatment of M. tuberculosis with BT-37 increases ethidium bromide (EtBr) uptake. The *M. tuberculosis mc*^*2*^*6206* strain was grown in the HdB medium supplemented with pantothenic acid, leucine, and Tween-80, and with different concentrations of BT-37. After adding EtBr, the fluorescence intensity (arbitrary fluorescence units) was measured for 3 h. Data are presented as the mean of duplicates ± SD.

**Figure S7.**
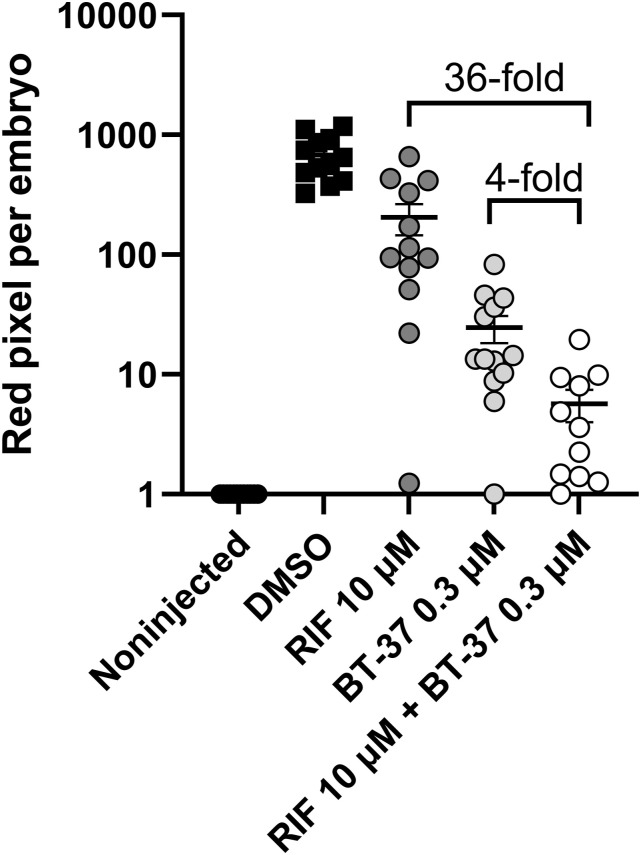
Synergistic effect of RIF and BT-37 in *M. marinum*-infected zebrafish embryos. The activity of rifampicin at 10 μM and BT-37 at 0.3 μM and their combination in the zebrafish embryo infection model. Zebrafish embryos were yolk-infected with *M. marinum* expressing *tdTomato*, and treatment was performed by immersion. Each data point represents the integrated red fluorescence intensity of a single zebrafish embryo, and the signal of each group is expressed as the mean ± SD of the mean. The fold difference between the means of different treatment groups is depicted.

Overall, several derivatives showed significantly improved in vitro and in vivo activity in *M. marinum* as compared to the parent compound **BT-08** (Table S6, Fig 3B). The three best compounds, **BT-37**, **BT-46**, and **BT-48**, were further tested in the macrophage–*M. tuberculosis* infection model (Fig 3C–E). Interestingly, all three compounds showed dose-dependent intracellular activity by reducing the amount of viable *M. tuberculosis* within macrophages (Fig 3C–E). In addition, the compound **BT-37** was the most successful in protecting macrophages from bacterial-induced lysis (Fig 3C). Thus, we decided to continue with compound **BT-37**. This molecule showed no cytotoxicity toward RAW 264.7 macrophages, THP-1 monocytes, and zebrafish embryos (Table S3) and did not lyse sheep red blood cells (Table S4). Compound **BT-37** did not inhibit the in vitro growth of selected fast-growing mycobacteria and some G+ and G- species (Table S2). Moreover, using the EtBr assay, we confirmed that the compound **BT-37** increases membrane permeability in *M. marinum*, as in the case of **BT-08** (Fig S5). In addition, **BT-37** increased EtBr uptake in *M. tuberculosis mc*^*2*^*6206* strain, suggesting a conserved phenotype between these two species (Fig S6). We further confirmed that **BT-37** synergizes with RIF in *M. marinum*-infected zebrafish embryos (Fig S7); however, the effect was less prominent compared with the **BT-08** (Fig 2A).

### Mutations in MMAR_0407 (Rv0164) cause resistance to BT-37

**Figure 4.**
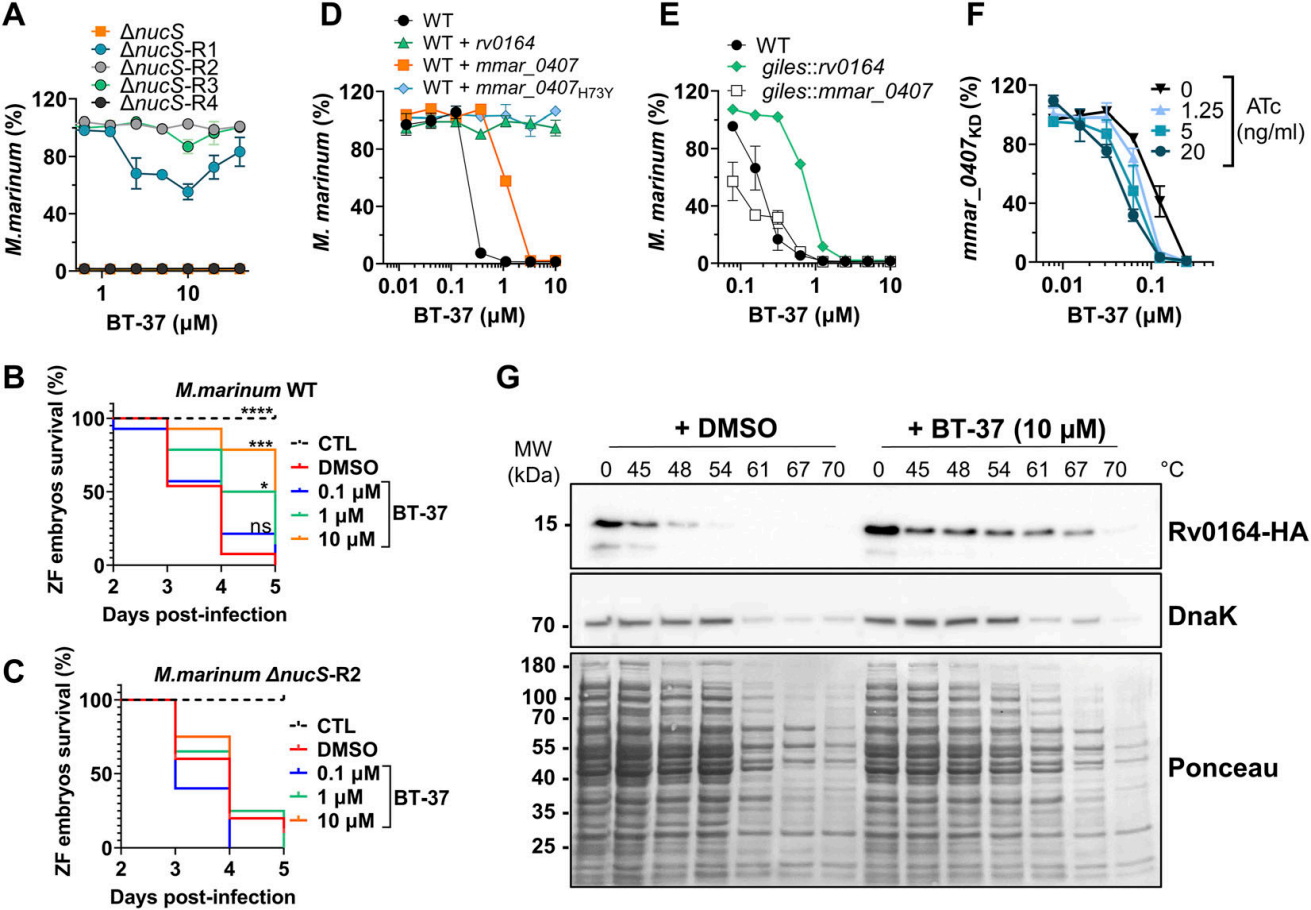
Mutations and expression level of MMAR_0407 (Rv0164) modulate susceptibility to BT-37. **(A)** Spontaneous BT-37–resistant mutants of hyper-mutating *M. marinum* Δ*nucS* strain were tested for their susceptibility toward BT-37 using the resazurin microtiter plate assay in the HdB medium with Tween-80. Data are presented as the mean of duplicates ± range. **(B, C)** Survival of zebrafish embryos that were yolk-infected with the *M. marinum* WT or (C) BT-37–resistant *M. marinum* strain (*M. marinum* Δ*nucS*-R2) after dose-dependent treatment with BT-37. Kaplan–Meier’s survival tests were conducted to generate the survival curves, and *P*-values were calculated by the log-rank test (*P = 0.0173; ***P = 0.0001; ****P ˂ 0.0001). CTL: non-infected control sample. **(D)** Susceptibility of the *M. marinum* WT strain or strains transformed with episomal plasmids pMN016-*rv0164* (WT+*rv0164*), pMN016-*mmar_0407* (WT+*mmar_0407*), or pMN016-*mmar_0407*_H73Y_ (WT+*mmar_0407*_H73Y_) toward compound BT-37 after 4 d of incubation in the HdB medium with Tween-80 using the resazurin microtiter plate assay. Data are presented as the mean of duplicates ± range. **(E)** Susceptibility of the *M. marinum* WT strain or strains transformed with an integrative plasmid pML1357-*mmar_0407* (*giles*::*mmar_0407*) or pML1357-*rv0164* (*giles*::*rv0164*) toward BT-37 in the HdB medium with Tween-80 using the resazurin microtiter plate assay. Data are presented as the mean of duplicates ± range. **(F)** *M. marinum* strain expressing *mmar_0407*-targeting sgRNA (*M. marinum mmar_0407*_KD_) was incubated with a range of concentrations of ATc and BT-37 in the HdB medium with Tween-80. The susceptibility of this ATc-inducible knock-down strain was measured using the resazurin microtiter plate assay. Data are presented as the mean of duplicates ± range. **(G)** Thermal shift assay of Rv0164-HA, which was expressed in *E. coli*. Cell lysates were incubated with BT-37 or DMSO and exposed to different temperatures. Denatured proteins were removed by centrifugation, and the remaining proteins were separated by SDS–PAGE (Ponceau) and analyzed by Western blotting. DnaK served as an internal control.

To identify the molecular target of **BT-37**, we raised spontaneously resistant mutants of *M. marinum*. Our approach was based on culturing *M. marinum* in the presence of a sublethal concentration of **BT-37** in the HdB medium and gradually increasing the **BT-37** concentration with every passaging step. However, the *M. marinum* WT strain did not develop resistance, even after prolonged incubation. Therefore, we employed a strain missing the endonuclease *nucS*, previously shown to have an increased mutation rate in *Mycobacterium smegmatis* by 100-fold compared with WT (Castañeda-García et al, 2017). We observed the growth of single *M. marinum* Δ*nucS* isolates selected during several passages with more than 30-fold higher MIC_90_ as compared to the parental Δ*nucS* strain (Fig 4A). Four isolates were chosen for confirmation experiments in dose–response assays. Although two isolates were completely resistant to the tested compound concentrations (Δ*nucS*-R2 and Δ*nucS*-R3), the isolate Δ*nucS*-R1 showed an intermediate phenotype. We also identified one sensitive isolate (Δ*nucS*-R4) (Fig 4A) that had been subjected to the same number of culturing steps and, therefore, was used as a control for mutations not involved in resistance. The genomes of the four isolates Δ*nucS*-R1-4, and the Δ*nucS* parental strain were sequenced.

The **BT-37**–resistant strains showed several mutated genes compared with the *M. marinum* Δ*nucS* reference strain (Table S7). Among them, isolates Δ*nucS*-R2 and Δ*nucS*-R3, which displayed high resistance to **BT-37** (Fig 4A), carried mutations in *mmar_2080* and *mmar_4794*. However, these mutations were absent in the resistant isolate Δ*nucS*-R1, leading us to exclude them from further investigation despite their potential impact on susceptibility. Instead, we focused on identifying mutations common to all three resistant isolates. In all three resistant strains (Δ*nucS*-R1, Δ*nucS*-R2, and Δ*nucS*-R3), we found mutations in genes *mmar_1438*, *mmar_1347*, and *mmar_0407* (Table S7). Notably, the mutations in genes *mmar_1438* and *mmar_1347* were also present in the **BT-37**–sensitive control strain Δ*nucS*-R4. Thus, we hypothesize that mutations in *mmar_1438* and *mmar_1347* arose during the continuous bacterial passaging and are unrelated to the resistance to compound **BT-37**, which leaves *mmar_0407* as our prime candidate.

Table S7. Single nucleotide polymorphisms identified in BT-37–resistant *M. marinum* Δ*nucS* mutants.

Interestingly, the resistant isolates that showed complete resistance, that is, Δ*nucS*-R2 and Δ*nucS*-R3 (Fig 4A), shared an identical mutation in *mmar_0407*, resulting in an amino acid change H73Y (Table S7), whereas the isolate Δ*nucS*-R1 (Fig 4A) contains a different mutation at a nearby codon, resulting in G69S (Table S7). This could explain the differences in the resistance profile of these isolates (Fig 4A). The gene *mmar_0407* encodes a small hypothetical protein that is conserved across mycobacteria and has an ortholog Rv0164 (TB 18.5) in *M. tuberculosis* (Marmiesse et al, 2004). The proteins of *M. marinum* and *M. tuberculosis* share 85.43% sequence identity. Transposon mutagenesis studies indicate that this gene is essential in both *M. marinum* and *M. tuberculosis* (Griffin et al, 2011; Dejesus et al, 2017), which would be in line with a potential drug target.

**Figure S8.**
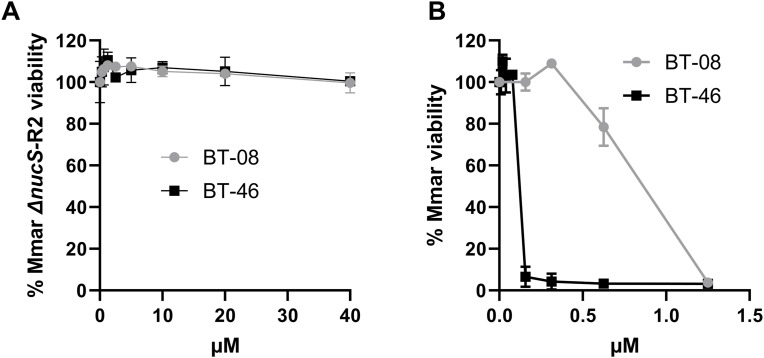
BT-08 and BT-46 exhibit cross-resistance to the BT-37–resistant M. marinum strain. **(A, B)** Susceptibility of *M. marinum* BT-37–resistant isolates (Mmar Δ*nucS*-R2) and (B) *M. marinum* WT strain using the resazurin microtiter plate assay and the HdB medium with Tween-80. Data are presented as the mean of duplicates ± SD.

**Figure S9.**
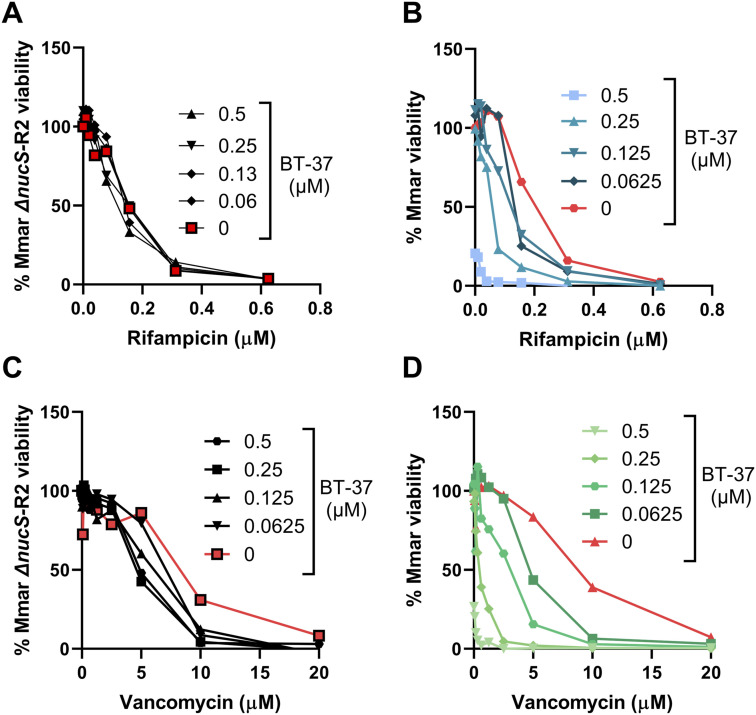
BT-37–resistant M. marinum strain does not show synergy with vancomycin or rifampicin in vitro. **(A, B, C, D)** *M. marinum* WT strain or BT-37–resistant strain (Mmar Δ*nucS*-R2) was investigated for the sensitivity toward the combinatorial treatment of compound BT-37 with rifampicin (A, B) or vancomycin (C, D) using the in vitro checkerboard assay and the HdB medium with Tween-80.

**Figure S10.**
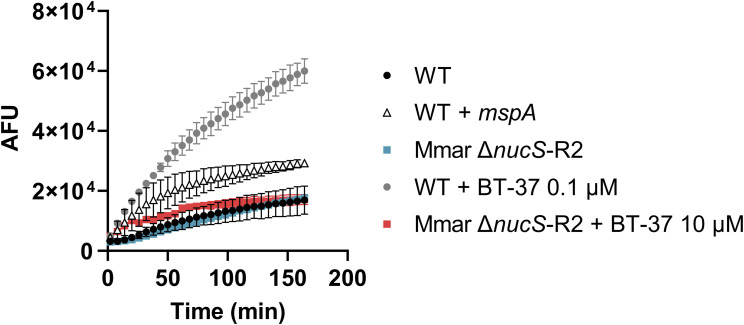
Treatment of BT-37–resistant M. marinum does not increases ethidium bromide (EtBr) uptake. The *M. marinum* WT strain or BT-37–resistant *M. marinum* (Mmar Δ*nucS*-R2) were grown in the HdB medium with Tween-80 and with different concentrations of BT-37. After adding EtBr, the fluorescence intensity (arbitrary fluorescence units) was measured for 3 h. Data are presented as the mean of duplicates ± SD.

We next showed that the identified **BT-37**–resistant *M. marinum* mutants are cross-resistant to other benzothiazole derivatives from our library, such as compounds **BT-08** and **BT-46** (Fig S8A), which exhibit low MIC against *M. marinum* WT (Fig S8B). The resistant strain was also investigated in zebrafish embryo survival assays, where zebrafish embryos are infected with a high bacterial load of *M. marinum* and embryo survival is monitored over several days upon treatment with the test compound (Fig 4B and C). Comparison of the lethality of WT or resistant *M. marinum* strains using the Kaplan–Meier survival test revealed no significant difference in the embryo mortality rate. This observation suggests that the resistant mutants do no exhibit changes in virulence in this model. Although treatment with **BT-37** significantly delayed the death of zebrafish embryos infected with the *M. marinum* WT strain compared with the non-treated group (Fig 4B), the compounds were ineffective against infection with the resistant mutant (Fig 4C), thus suggesting that the compound’s activity in vivo is anti-bacterial and not host-directed. Moreover, the resistant strain no longer exhibited synergistic activity between **BT-37** and RIF or vancomycin when tested using in vitro checkerboard assays (Fig S9A–D), indicating that the synergistic effect is linked to the gene *mmar_0407* (*rv0164*). In the EtBr uptake assay, both the resistant strain treated with compound **BT-37** and the untreated resistant strain exhibited a comparable phenotype to the WT strain (Fig S10). This suggests that the presence of the compound alone may not suffice to permeabilize the membrane. Rather, it must effectively target MMAR_0407.

### BT-37 is targeting MMAR_0407 (Rv0164)

To confirm that the mutations in *mmar_0407* are linked to **BT-37** resistance, we overexpressed the WT *mmar_0407* gene, the mutated gene *mmar_0407* H73Y, and the *M. tuberculosis* ortholog *rv0164* using the replicative vector pMN016 in the *M. marinum* WT strain. We then assessed the susceptibility of these overexpressing strains to **BT-37** (Fig 4D). The overexpression of *mmar_0407* led to a threefold shift in the MIC_90_ (Fig 4D), likely because of increased production of the target protein. On the contrary, the overexpression of *mmar_0407* with the H73Y mutation resulted in complete resistance to **BT-37**, providing further evidence of its involvement in the compound’s resistance (Fig 4D). Interestingly, the overexpression of the *M. tuberculosis* ortholog *rv0164* also resulted in complete resistance (Fig 4D).

To investigate this further, we reduced the overexpression of genes *rv0164* and *mmar_0407* by integrating the genes into the genome using the *giles* integration site targeted by vector pML1357. The resulting strains were both sensitive to the compound; however, even with reduced expression levels, the expression of the *M. tuberculosis* ortholog (*giles*::*rv0164*) still caused a higher level of resistance to compound **BT-37** than *mmar_0407* (*giles*::*mmar_0407*) (Fig 4E).

In an alternative approach, we used CRISPR/Cas9 interference (CRISPRi) to construct a conditional knock-down strain of *mmar_0407* (*mmar_0407*_*KD*_), regulated by the addition of anhydrotetracycline (ATc) (Meijers et al, 2020; Wong & Rock, 2021). We tested the strain in the checkerboard assay, using ATc to gradually reduce the *mmar_0407* expression in a dose-dependent manner. The *mmar_0407*_*KD*_ strain was more sensitive in the presence of ATc to compound **BT-37** as compared to the non-induced strain (Fig 4F).

To directly prove the binding between Rv0164 and the inhibitor, we used a thermal shift assay. We assessed Rv0164’s stability at various temperatures with and without the inhibitor. Proteins have characteristic denaturation temperatures that can change with substrate or inhibitor binding. Rv0164-HA was expressed in *Escherichia coli*, with DMSO or **BT-37** (10 μM). We tested temperatures from 45 to 70°C, blotted lysates on a membrane, and stained with Ponceau dye (Fig 4G). In both **BT-37** and DMSO samples, soluble protein decreased as temperature rose. However, with **BT-37**, Rv0164-HA stayed soluble up to 67°C, whereas the DMSO-treated protein denatured at 54°C. We also probed for DnaK, a chaperone protein, which showed similar heat resistance in both samples. These results confirm that **BT-37** alters Rv0164’s heat stability, likely by binding and stabilizing the protein. This supports MMAR_0407 (Rv0164) as the target for these benzothiazole compounds.

### Molecular docking simulations predict the binding of BT-37 to the hydrophobic pocket of Rv0164

**Figure 5.**
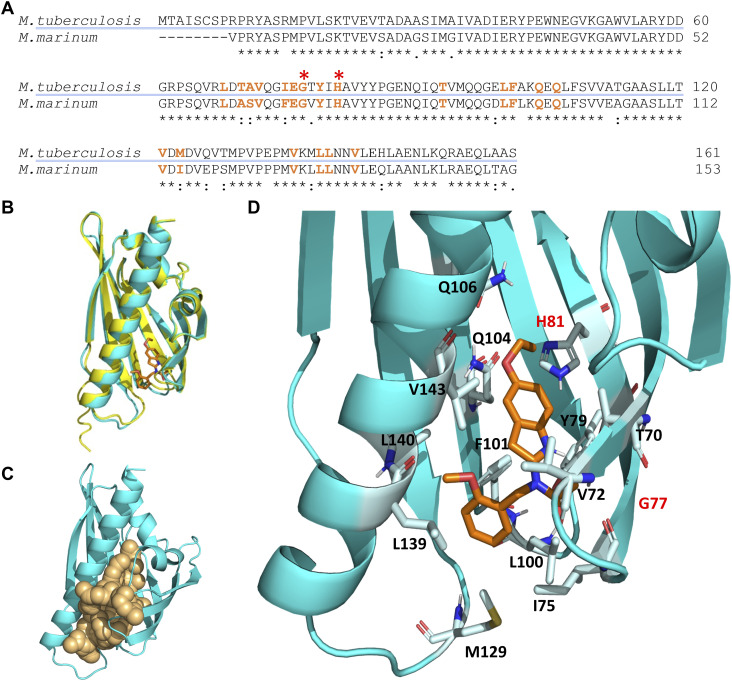
Molecular docking simulation of the structure of the Rv0164 complex with BT-37. **(A)** Protein sequence alignment of MMAR_0407 in *M. marinum* and Rv0164 in *M. tuberculosis* using Clustal Omega. An asterisk (*) indicates positions that have identical residues, a colon (:) indicates that the residues have conserved properties, and a dot (.) indicates residues that are semi-conserved. Amino acids highlighted in orange are in 2 Å vicinity of the compound BT-37 and contribute to inhibitor binding. Red asterisks indicate residues contributing to experimentally confirmed resistance in *M. marinum* (Mmar G69/Mtb G77 and Mmar H73/Mtb H81). **(B)** Structure alignment of MMAR_0407 in *M. marinum* (yellow) and Rv0164 in *M. tuberculosis* (cyan). The RMSD score between these structures is 0.158 Å. In orange is depicted the compound BT-37 as the top model of docking simulation, according to the HADDOCK score. **(C)** Definition of the top-scored binding pocket in Rv0164 that was used to define the binding information of the docking simulation. **(D)** Top model of the docking simulation, according to the HADDOCK score, is shown in more detail. The corresponding residues in *M. tuberculosis* (G77, H81) contributing to experimentally confirmed resistance in *M. marinum* are labeled in red.

Lastly, we sought a comprehensive understanding of how benzothiazole inhibitors interact with the target protein. To achieve this, we used the AlphaFold protein folding prediction system to predict the structures of Rv0164 and MMAR_0407 (Jumper et al, 2021; Varadi et al, 2022). The resulting structures exhibited remarkable similarity, with a sequence identity of 85%, demonstrated by an RMSD score of 0.158 Å upon structure alignment (Fig 5A and B). Notably, these structures consist of seven parallel beta sheets and three alpha helices, with very high similarity to the crystal structure solved for the ortholog in *M. smegmatis* (RMSD 0.722 Å) (Zheng et al, 2018). Next, we aimed to identify potential binding pockets in the Rv0164 structure using the software Fpocket (Le Guilloux et al, 2009; Schmidtke et al, 2010), which employs a geometry-based approach to locate suitable empty spaces. This analysis revealed a substantial pocket situated centrally within the protein’s structure (Fig 5C). This accessible pocket, originating from the protein’s exterior, presents a potential binding site for ligands. Subsequently, we performed docking simulations of Rv0164 and compound **BT-37** using the docking software HADDOCK2.4 (Dominguez et al, 2003; Van Zundert et al, 2016). To this end, we generated 50 conformations of **BT-37** and then docked these ligands into the predicted Rv0164 structure, resulting in 200 models clustered based on their RMSD score and ranked according to the HADDOCK score. All top 10 clusters contained models predicting **BT-37** inside the hydrophobic pocket of Rv0164. Notably, the best-scoring model of the best cluster according to the HADDOCK score predicted the compound in close vicinity to the highlighted amino acids within the binding pocket, including H81 and G77, which correspond to amino acids H73 and G69 of MMAR_0407 (Fig 5A and D). These residues are of particular interest because we have shown that mutations G69S and H73Y in MMAR_0407 cause resistance to **BT-37** (Table S7). Thus, our predicted model suggests that the corresponding residues in Rv0164 are important for **BT-37** to bind the target. Taken together, we confirmed Rv0164 (MMAR_0407) to be the target of our new benzothiazole compounds by genetic, protein-based, and in silico binding studies.

## Discussion

The mycobacterial cell wall plays a crucial role in the interaction of the tubercle bacillus with the host and in resistance to anti-microbial chemotherapy. We identified benzothiazole-based compounds that increase the permeability of the mycobacterial outer membrane, offering insights into possible manipulations to enhance the transport of host immune factors and the influx of antibiotics.

Our primary hit, compound **BT-08**, increases membrane permeability, facilitating the uptake of several substrates, as shown for EtBr and resazurin, and increases susceptibility of several antibiotics. Notably, these effects seem most prominent with the higher molecular weight compounds (>800*g*/mol), which typically face challenges penetrating the mycobacterial cell wall. This observation could serve as a starting point to investigate new combination therapies, broadening treatment options with approved antibiotics previously overlooked for mycobacterial infections because of their limited uptake. The in vitro activity of compound **BT-08** is influenced by the detergent used in the growth media. Hence, it should be considered that the absence of toxicity in mammalian cell cultures might also be influenced by the specific medium conditions used in the corresponding in vitro assays. Nevertheless, **BT-08** and its derivatives showed activity and no toxicity in the zebrafish embryo model, which holds great promise. We are uncertain why tyloxapol reduces activity, whereas Tween-80 enhances it. One possibility is that mycobacteria use Tween-80 as a carbon source but not tyloxapol. Because Tween-80 contains fatty acids, a preferred carbon source by *M. tuberculosis* during infection, inhibition of Rv0164 might cause metabolic issues when grown on fatty acid carbon sources, found in vivo and in Tween-80–containing media.

Using medicinal chemistry approaches, we generated derivative **BT-37** exhibiting enhanced activity in *M. marinum*–zebrafish and *M. tuberculosis*–macrophage infection models. The compound retains the synergistic interaction with RIF during zebrafish infection studies and increases EtBr uptake in *M. marinum* and *M. tuberculosis*, indicating a shared mechanism of action with **BT-08**. We observed that, counterintuitively, as the anti-bacterial activity of **BT-37** increased, the synergistic effect with antibiotics became less pronounced compared with the initial hit compound, **BT-08**. However, we still consider **BT-37** as a more potent analog based on the sole performance during zebrafish and macrophage infection experiments. Further exploration is needed to elucidate the specific mechanisms underlying this phenomenon. We confirmed by cross-resistance studies that spontaneously resistant *M. marinum* mutants were resistant to both analogs, revealing that MMAR_0407 (Rv0164) is the target of the investigated benzothiazoles, and the observed synergy with RIF or vancomycin relies on inhibiting MMAR_0407, not just the presence of the compound. This aligns with Li et al’s study, which used CRISPR interference technology to alter the expression of *M. tuberculosis* genes and assessed bacterial fitness in the presence of different drugs (Li et al, 2022). Using this global approach, they showed increased susceptibility to vancomycin and RIF when silencing *rv0164* (Li et al, 2022). This is significant because, despite the emergence of drug resistance, most of the global TB cases are RIF-sensitive (World Health Organization, 2022a), and enhancing RIF activity with the described benzothiazoles could shorten treatment duration. We also confirmed this synergy in the zebrafish TB model, where it was even more pronounced, suggesting the host’s immune system aids bacterial clearance, which could also be important in the context of human infections.

MMAR_0407 (Rv0164) is a conserved protein across mycobacterial species (Marmiesse et al, 2004). Rv0164 has been previously identified as T-cell (Lim et al, 2004; Sable et al, 2005; Eweda et al, 2010) and B-cell (Ireton et al, 2010; Singh et al, 2017) antigens and is essential for *M. tuberculosis* growth (Griffin et al, 2011; Bosch et al, 2021). The biological function of Rv0164 is unknown to date. However, several studies have investigated its homolog in *M. smegmatis*, MSMEG_0129 (Zheng et al, 2017, 2018; Zhou et al, 2020, 2021), which shares 59% identity with Rv0164. These proteins are classified as polyketide aromatase/cyclase family members of the steroidogenic acute regulatory protein–related lipid transfer (START) domain superfamily based on sequence alignment (Zheng et al, 2018). However, subsequent crystal structure analysis of MSMEG_0129 revealed that the potential catalytic residues are not conserved (Zheng et al, 2017, 2018). Notably, the crystal structure of MSMEG_0129 unveiled a hydrophobic pocket that could potentially accommodate a lipid molecule, suggesting a role in lipid transfer during cell envelope synthesis (Zheng et al, 2017, 2018).

In another study, MSMEG_0129 was found to interact with the ClpP2 protease and the transcription factor CarD, and their expression was found to be dependent on growth conditions (Zhou et al, 2020). For instance, nutrient deficiency led to the down-regulation of MSMEG_0129 and Clp2 and the up-regulation of CarD. A recent study mapped the transcriptional profile of strains with the decreased expression of *msmeg_0129* and found numerous genes involved in cell wall biosynthesis and metabolism to be differentially expressed, leading the authors to propose that MSMEG_0129 might coordinate signal transfer during stress responses (Zhou et al, 2021). It remains to be confirmed whether the same is true for Rv0164.

Despite limited knowledge about the function of Rv0164, its potential as a novel drug target is of great importance. Interestingly, Rv0164 also emerged in a recent report where resistant strains of *M. tuberculosis* were generated against various compounds (Tsui et al, 2022). Mutations in the *rv0164* gene were observed in strains resistant to GSK1458296A (compound 296A), which is structurally distinct from the benzothiazole compounds reported in this study. The emergence of *rv0164* mutations in strains resistant to structurally diverse compounds further validates the protein as a drug target. Notably, the amino acid changes identified in GSK1458296A-resistant strains (V49L, Y79C, H81Q, T93A) were distinct from the mutated residues in our study. However, mutations identified in both studies are located in close proximity to the binding pocket. The distinct mutations between studies reveal a commonality in the importance of the hydrophobic pocket for binding diverse molecules, which can be exploited in future drug design.

Collectively, our findings highlight the discovery of novel compounds containing a benzothiazole core that binds to an unexplored drug target in mycobacteria named Rv0164 (MMAR_0407). Our results demonstrate that inhibition of MMAR_0407 enhances the permeability of the mycobacterial cell envelope, rendering the bacteria more susceptible to the host’s immune system and other antibiotics. Moreover, combination treatment with these compounds and conventional antibiotics exhibited synergistic and cooperative interactions. Thus, our study unveils a novel mycobacterial vulnerability, enhancing cell wall permeability and synergy with established antibiotics, crucial for future drug discovery efforts.

## Materials and Methods

### Reagents and compounds

All commercial chemicals and reagents were solubilized and stored according to the manufacturer’s recommendations. Ampicillin sodium salt, d-cycloserine, erythromycin, ethambutol, ethidium bromide, ethionamide, fluorescein sodium salt, gentamycin, kanamycin sulfate, penicillin G sodium, polymyxin B, resazurin sodium salt, RIF, nisin from *Lactococcus lactis*, tetracycline, and vancomycin were all purchased from Sigma-Aldrich. Bedaquiline, linezolid, macozinone, pretomanid, spectinomycin, streptomycin sulfate salt, and SQ109 were all purchased from MedChem Express. Anhydrotetracycline was purchased from Thermo Fisher Scientific, hygromycin from Roche, and fusidic acid sodium salt from Merck. Compounds from the benzothiazole-core library were stored as stock solutions (10 mM) in DMSO at −20°C.

### Bacteria and cell lines

The bacterial strains used in this study are listed in Table S8. *M. marinum* strains were grown on 7H10 agar (Difco) plates with 10% ADS (0.5% BSA, 0.2% dextrose, 0.085% sodium chloride) at 30°C or cultured in the following liquid media: (i) Middlebrook 7H9 medium supplemented with 10% ADS, 0.2% glycerol, and 0.05% Tween-80 or 0.02% tyloxapol; (ii) Hartman’s de Bond liquid medium (HdB) (Smeulders et al, 1999) supplemented with 0.5% glucose, 0.5% glycerol, and 0.05% Tween-80 or 0.02% tyloxapol; and (iii) Sauton’s medium (Atlas & Parks, 1993) supplemented with 0.05% Tween-80, at 30°C. *M. tuberculosis* H73Rv, *Mycobacterium abscessus*, and *M. smegmatis* were cultured in 7H9 medium supplemented with 10% ADS, 0.2% glycerol, and 0.02% tyloxapol, or 7H10 agar plates supplemented with 10% ADS at 37°C. The *M. tuberculosis mc*^*2*^*6206* strain was cultured in HdB media supplemented with 0.5% glucose, 0.5% glycerol, 0.05% Tween-80, pantothenic acid 24 μg/ml and leucine 50 μg/ml to allow growth of the auxotrophic strain. *E. coli*, *Bacillus subtilis*, *Klebsiella pneumoniae*, and *Acinetobacter baumannii* were cultured in Luria–Bertani (LB; Difco) medium or LB agar plates at 37°C. When appropriate, the antibiotics hygromycin (50 μg/ml) or kanamycin (25 μg/ml) were added to the growth media. THP-1 human monocytes (ATCC TIB-202) were cultured in RPMI medium with GlutaMAX (Gibco) supplemented with 10% FBS at 37°C with 5% CO_2_. RAW 264.7 murine macrophages (ATCC TIB-71) were cultured in DMEM with GlutaMAX (Gibco) supplemented with 10% FBS at 37°C with 5% CO_2_.

Table S8. List of strains used this study.

### Ethidium bromide uptake assay

The ethidium bromide (EtBr) uptake assay was performed as described previously (Boot et al, 2017). The growth medium used in experiments with *M. marinum* strains in Fig 1A and B was 7H9 medium supplemented with 10% ADS, 0.2% glycerol, and 0.02% tyloxapol, whereas in Figs S5 and S10, the growth medium used was the HdB medium supplemented with 0.5% glucose, 0.5% glycerol, and 0.05% Tween-80. The experiment with *M. tuberculosis mc*^*2*^*6206* strain used in Fig S6 was performed in HdB media supplemented with 0.5% glucose, 0.5% glycerol, 0.05% Tween-80, pantothenic acid 24 μg/ml, and leucine 50 μg/ml to allow growth of the auxotrophic strain. The strains were pregrown until the mid-logarithmic phase, inoculated (OD_600_ = 0.1) in specified media with the addition of the compound at indicated concentrations, and grown for an additional 72 h. The cells were harvested by centrifugation (3,000*g*, 10 min) and washed in PBS supplemented with 0.02% tyloxapol. Bacteria were resuspended in PBS with 0.02% tyloxapol and distributed with a final OD_600_ of 0.8 in a transparent, round-bottom, 96-well microtiter plate. EtBr was added with a final concentration of 5 μg/ml per well. Fluorescence was measured every 3 min at 30°C, using a BioTek plate reader (Synergy H1), bottom reading mode, and excitation 300 nm/emission 605 nm. The measurements were taken in triplicates. As a positive control, WT *M. marinum* M^USA^ transformed with plasmid pSMT3-*mspA* (Ates et al, 2015) was used. The EtBr uptake was compared between the compound-treated and non-treated (WT) samples after 60 min. The ratio between compound-treated and non-treated samples was used as a readout.

### Resazurin uptake assay

*M. marinum* was grown in the 7H9 medium supplemented with 10% ADS, 0.2% glycerol, and 0.02% tyloxapol for 3 d at 30°C in the presence of the compound at the indicated concentration. Bacterial cells were harvested by centrifugation (3,000*g*, 10 min) and washed in PBS supplemented with 0.02% tyloxapol. Next, 160 μl of washed bacteria with a final OD of 1 was added to each well of a 96-well plate. The resazurin solution was prepared by mixing resazurin sodium salt (0.025% [wt/vol] in Milli-Q) and 20% Tween-80 in a ratio of 3:1, and 20 μl of the mixture was added to each well. The resazurin dye conversion was measured every 3 min at 30°C, using a BioTek plate reader (Synergy H1), bottom reading mode, and excitation 560 nm/emission 590 nm.

### Bacterial susceptibility assays

The resazurin microtiter plate assay (REMA) was used to determine minimal inhibitory concentrations (MIC) of the compound toward mycobacterial species (Palomino et al, 2002). Selected compounds or antibiotics were diluted in the bacterial growth medium as twofold serial dilutions in a 96-well plate. Bacteria were routinely grown until the mid-logarithmic phase, harvested by centrifugation (3,000*g*, 10 min), washed in PBS supplemented with 0.02% tyloxapol, resuspended in the growth medium, and added to the well plate containing compound dilutions, to achieve the final OD_600_ of 0.001 per well. The growth media used in the assays using *M. marinum* were the HdB medium supplemented with 0.5% glucose, 0.5% glycerol, and 0.05% Tween-80, except for experiments in Fig 1E and experiments with other mycobacteria where 7H9 medium supplemented with 10% ADS, 0.2% glycerol, and 0.02% tyloxapol was used. The plates were sealed with parafilm and incubated for 4 d at 30°C for *M. marinum*, 1 d at 37°C for *M. smegmatis*, 2 d at 37°C for *Mycobacterium abscessus*, and 6 d at 37°C for *M. tuberculosis*. After incubation, the resazurin solution containing resazurin sodium salt (0.025% [wt/vol] in Milli-Q) and 20% Tween-80 (ratio 3:1) was added to each well, and plates were further incubated. When the color conversion of the dye was observed, the fluorescence was measured using a BioTek plate reader (Synergy H1), bottom reading mode, and excitation 560 nm/emission 590 nm. The MIC of compounds toward *E. coli*, *Bacillus subtilis*, *K. pneumoniae*, and *A. baumannii* was determined by tracking changes in the OD_600_. Bacteria were grown overnight, then freshly diluted in LB medium, and grown to the mid-logarithmic phase. Selected compounds were diluted in LB medium as a twofold dilution in a 96-well plate. Bacterial cells were washed in PBS and added as an OD_600_ of 0.001 per well in a 96-well plate containing compound dilutions. Plates were sealed and incubated at 37°C with 3 mm continuous linear shaking in a BioTek plate reader (Synergy H1), and the bacterial growth was tracked by measuring the OD_600_ every 15 min. All compounds were tested in duplicates or triplicates. The data of each 96-well plate were normalized to DMSO-treated wells (100% viability) after the background subtraction (medium only). The MIC_90_ values represent the lowest concentration of the compound that results in 90% growth inhibition.

### Zebrafish (*Danio rerio*) husbandry

All zebrafish experiments in this study were performed using transparent *casper* (*roy*^*a9/a9*^*;nac*^*w2/w2*^) (White et al, 2008) zebrafish (*D. rerio*) embryos. Adult fish were kept in recirculating tank systems at the Amsterdam Animal Research Center of the Vrije Universiteit University according to standard protocols (zfin.org). All protocols followed the international guidelines on the protection of animals used for scientific purposes specified by the EU Directive 2010/63/EU, which allows zebrafish larvae to be used up to the moment of free-living.

### Zebrafish (*D. rerio*) embryo infection studies

Injection stocks of *M. marinum* strains carrying a plasmid pMS2-*tdTomato* were prepared in PBS with 20% glycerol, aliquoted, and stored at −80°C. Before use, the injection stocks were diluted with the green fluorescent dye fluorescein (2.5 μg/ml in PBS) to visualize and control the injection process. The number of injected bacteria was determined by plating the injection volume of bacterial suspension on 7H10 agar plates containing appropriate antibiotics, followed by counting CFU. Infection of zebrafish embryos was performed using an automated microinjection system (Life Science Methods BV) as described previously (Spaink et al, 2013). Briefly, zebrafish embryos were infected 1 h post-fertilization at 2–32 cell stage with *M. marinum* mixed with fluorescein (2.5 μg/ml in PBS). Each embryo was infected with 1 nl containing 80–150 CFU of *M. marinum*, whereas for survival experiments, ∼1,000 CFU was injected into each embryo. Successfully infected embryos were incubated overnight at 31°C in E3 medium (5.0 mM NaCl, 0.17 mM KCl, 0.33 mM CaCl·2H_2_O, 0.33 mM MgCl_2·_6H_2_O) supplemented with 0.3 mg/liter methylene blue until treatment with test compounds. 1 d post-infection, embryos were divided into treatment groups of 12–15 embryos per well in 12-well plates and incubated with the test compounds that were diluted in fish water (60 μg/ml instant ocean sea salts) and incubated at 28°C. The survival rate was determined daily based on the functioning of the embryos’ heart and blood circulation. Determination of the bacterial load in infected zebrafish embryos was performed 3 d after the treatment. Embryos were anesthetized in 0.02% (wt/vol) buffered 3-aminobenzoic acid methyl ester (pH 7.0) (Tricaine; Sigma-Aldrich), and the bacterial load was monitored with an Olympus IX83 fluorescence microscope (4x objective magnification, Hamamatsu ORCA-Flash 4.0 camera) at specific wavelengths (excitation/emission: 470/519 nm; 550/610 nm). Obtained images were analyzed using CellProfiler 3.19 (Broad Institute, Cambridge, USA) with a custom-made pipeline to count and quantify pixel intensity within the embryos. Integrated red fluorescence intensity per embryo was used as a readout for bacterial burden. Image acquisition and image analysis were automated. The effect of drug treatment in infected zebrafish embryos was analyzed and depicted using GraphPad Prism version 9.0.0 (GraphPad Software Inc.). Each data point represents an integrated red fluorescence intensity signal from a single zebrafish embryo. The signal from the non-infected embryos was used to set the signal threshold to 1. Data were log_10_-transformed to achieve normal distribution, and furthermore, statistical analysis was done as a one-way ANOVA, and Dunnett’s multiple comparison test was used, where the signal from the DMSO-treated control sample was compared with each treatment group. Significance is indicated as follows: *P ≤ 0.05; **P ≤ 0.01; ***P ≤ 0.001; ****P ≤ 0.0001. Survival curves for zebrafish survival experiments were generated using Kaplan–Meier survival tests.

### Zebrafish (*D. rerio*) embryotoxicity studies

Fertilized zebrafish embryos were collected and kept overnight at 28°C in E3 medium supplemented with 0.3 mg/liter methylene blue. 1 d post-fertilization (dpf), zebrafish embryos were distributed as 10–12 embryos per well in 12-well plates and treated with compounds diluted in fish water. Zebrafish embryos were incubated at 28°C for 5 d with the test compound at indicated concentrations, and the morphology and mortality of zebrafish embryos were monitored daily.

### Generation of spontaneous compound-resistant *M. marinum* mutants

To generate spontaneously BT-37–resistant *M. marinum* mutants, we used the hyper-mutating *M. marinum* Δ*nucS* strain (Izquierdo Lafuente et al, 2024). We grew *M. marinum* Δ*nucS* in the HdB medium supplemented with 0.5% glucose, 0.5% glycerol, and 0.05% Tween-80 and increasing concentrations of compound BT-37, starting from 0.3x MIC to 10x MIC over six culturing passages. Single colonies were obtained by streaking cultures on solid plates. The resistance to BT-37 was determined by testing the susceptibility of strains to BT-37 using the REMA. Genomic DNA extraction of the parental *M. marinum* Δ*nucS* strains, three BT-37–resistant isolates, and one susceptible isolate was done using phenol/chloroform/isoamyl alcohol extraction as described previously (Mve-Obiang et al, 2001). Whole-genome sequencing of genomic DNA was outsourced to Beijing Novogene Bioinformatics Technology Co., Ltd. (Novogene) using Illumina sequencing technology. Generated reads were aligned to the reference genome of *M. marinum* M^USA^ (NC_010612.1) and compared with the parental *M. marinum* Δ*nucS* strain using the software QIAGEN CLC Genomics Workbench 12 (QIAGEN).

### Cytotoxicity in macrophages

Compounds were prepared as twofold serial dilutions in the appropriate medium. For assays with THP-1 monocytes, the RPMI GlutaMAX with 10% FBS medium was used, whereas for RAW macrophages, the DMEM GlutaMAX with 10% FBS medium was used. Cells were seeded as 2.5 × 10^4^ cells/well in 96-well plates containing compound dilutions, and the plates were incubated for 3 d at 37°C with 5% CO_2_. After incubation, resazurin sodium salt (0.025% [wt/vol] in PBS) was added to each well, and plates were incubated at 37°C with 5% CO_2_. When color conversion was observed (after ∼4 h), fluorescence intensity corresponding to the metabolically active cells was measured using a BioTek plate reader (Synergy H1), bottom reading mode, and excitation 560 nm/emission 590 nm. All compounds were tested in duplicates. Non-treated samples represented 100% viability.

### Macrophage infection studies

The compound’s intracellular activity was investigated in macrophage infection studies, as described previously (Habjan et al, 2021). Infection stocks of *M. tuberculosis* H37Rv transformed with pTetDuo were prepared in RPMI GlutaMAX with 10% FBS as the infection medium, supplemented with 20% glycerol, and stored at −80°C. All incubation steps were performed at 37°C, 5% CO_2_. All solutions were prepared in RPMI GlutaMAX with 10% FBS. THP-1 human monocytes were seeded into black 96-well plates (Ibidi) as 10^5^ cells/well and incubated with phorbol 12-myristate 13-acetate (PMA) at 25 ng/ml for 48 h to induce differentiation into macrophage-like cells. Next, macrophages were washed and infected with *M. tuberculosis*–pTetDuo at a multiplicity of infection (MOI) of 5. After a 3-h incubation, gentamycin (50 μg/ml) was added for 1 h, to remove extracellular bacteria. Meanwhile, serial dilution of test compounds was prepared in separate 96-well plates. Then, gentamycin solution was removed from wells and replenished with compound’s solution. The plates were incubated for 4 d, and then, anhydrotetracycline (ATc) solution to a final concentration of 100 ng/ml was added to the wells followed by a 24-h incubation. Afterward, the medium was removed and the fixating solution (3.2% [wt/vol] PFA in PBS) was added to the wells for 30 min at room temperature and later replaced with quenching/staining solution (0.1 M glycine, 0.2% [wt/vol] Triton X-100, and Hoechst dye at 1:500 in PBS) for 1 h in the dark. All wells were washed twice with PBS before being imaged using an Olympus IX83 fluorescence microscope with a 20x objective magnification and a Hamamatsu ORCA-Flash 4.0 camera. Images of each well were taken at specific wavelengths (excitation/emission: 385/455 nm; 470/519 nm; 550/610 nm). Images were analyzed using a custom-made pipeline in CellProfiler 3.19 (Broad Institute, Cambridge, USA), as described previously (Habjan et al, 2021). Briefly, the fluorescent signal of the ATc-inducible GFP around each macrophage was used as a readout for viable intracellular bacteria in each macrophage, whereas the number of stained and detected nuclei was used as a readout for the number of macrophages in each treatment group.

### Hemolysis assay

The hemolytic effect of a compound on the red blood cells was investigated (Schouten et al, 2022). Compounds were prepared as twofold serial dilutions in phenol red–free DMEM (Gibco, Life Technologies). Meanwhile, defibrinated sheep erythrocytes (BioTrading) were harvested by centrifugation (600*g*, 7 min, 4°C), gently washed five times in phenol red–free DMEM, and seeded as 4.2 × 10^7^ erythrocytes per well, in plates containing dilutions of the test compounds. The plate was centrifuged (610*g*, 5 min) and incubated at 37°C, 5% CO_2_ for 3 h. After incubation, the cells were resuspended and centrifuged, and the supernatant was transferred to a flat-bottom 96-well plate. The hemoglobin release was measured as an absorbance of 405 nm using a BioTek plate reader (Synergy H1). After background subtraction, the data were normalized to Triton X-100–treated samples = 100% hemolysis.

### Checkerboard assay

Drug-to-drug interactions between test compounds A and B were determined using an in vitro checkerboard assay, as described previously (Hsieh et al, 1993). The growth media used in the assay were the HdB medium supplemented with 0.5% glucose, 0.5% glycerol, and 0.05% Tween-80, except for experiments in Fig S4 where 7H9 medium supplemented with 10% ADS, 0.2% glycerol, and 0.02% tyloxapol was used. Compound A was prepared as a twofold serial dilution in the bacterial growth medium in a 96-well plate, with dilutions directed horizontally. Compound B was prepared as a twofold serial dilution in a separate 96-well plate, dilutions directed vertically. Then, the solution from the plate containing dilutions of Compound B was transferred to the plate containing dilutions of Compound A, resulting in a checkerboard titration of Compound A on the horizontal axis and Compound B on the vertical axis. The selected mycobacterial strain was routinely grown until the mid-logarithmic phase, harvested by centrifugation (3,000*g*, 5 min), washed in PBS with 0.02% tyloxapol, and diluted to an OD_600_ of 0.001 per well in 96-well plates. Plates were sealed and incubated (*M. marinum* for 4 d at 30°C, *M. tuberculosis* for 6 d at 37°C). After incubation, a resazurin solution consisting of resazurin sodium salt (0.025% [wt/vol] in Milli-Q) and 20% Tween-80 (ratio 3:1) was added to each well and the plates were further incubated. When a color conversion of the dye was observed, the fluorescence was measured using a BioTek plate reader (Synergy H1), bottom reading mode, and excitation 560 nm/emission 590 nm. First, the MIC_90_ values of compounds A and B alone were determined as the lowest concentration of the compound, which results in 90% growth inhibition. Next, the MIC_90_ values of combinations between Compound A + Compound B and Compound B + Compound A were determined. Thus, the FIC for each compound can be calculated as the difference in MIC between the treatment of a single compound or in combination, following the formula (European Committee for Antimicrobial Susceptibility Testing EUCAST of the European Society of Clinical Microbiology and Infectious Dieases ESCMID, 2000):

$$FIC_{A}=MIC_{A+B}/MIC_{A}$$

$$FIC_{B}=MIC_{B+A}/MIC_{B}$$

Next, the FICI (FIC_index_ or ΣFICI) was calculated as

$$\sum FIC=FIC_{A}+FIC_{B}$$

Based on the obtained ΣFIC value, the drug-to-drug interactions can be determined as follows:

ΣFIC ≤ 0.5 represents synergism, 0.5 < ΣFIC ≤ 1 represents additive effect, 1 < ΣFIC ≤ 2 represents indifference, and ΣFIC > 2 shows antagonism.

### Chemistry

All reagents and solvents were purchased from commercial suppliers (Sigma-Aldrich, Alfa Aesar, Acros, Chimmed) and used without further purification. The ^1^H and ^13^C spectra were recorded on a Bruker AC-300 (200 MHz, ^1^H) or a Bruker AC-200 (50 MHz, ^13^C) NMR spectrometer. Chemical shifts were measured in DMSO-d_6_, using tetramethylsilane as an internal standard, and reported as ppm values. The following abbreviations indicate the multiplicity: s, singlet; d, doublet; t, triplet; m, multiplet; q, quartet; brs, broad singlet; brm, broad multiplet. Mass spectra were recorded on a Finnigan MAT INCOS 50 quadrupole mass spectrometer (EI, 70 eV) with direct injection (Thermo Finnigan). The purity of the final compounds was analyzed by analytical HPLC on an Elute HPLC system (Bruker Daltonik) equipped with an Azura UVD 2.1S UV detector (Knauer) with a wavelength at 254 nm and acquisition rate at 1 Hz. Chromatographic separation was carried out on an Acquity HSS T3 column (2.1 × 100 mm, 1.3 μm, 100 Å; Waters) at 30°C, with sample injection volume—2.0 μl. A mobile phase consisting of 0.1% formic acid in water (A), and 0.1% formic acid in acetonitrile (B) was programmed with a gradient elution of 30–95% over 10 min at a flow rate of 250 μl/min. Data were processed using Compass DataAnalysis 5.1 software (Bruker Daltonik). All final compounds were >95% pure. Elemental analysis (% C, H, N) was performed on a EURO EA elemental analyzer (HEKAtech). IR spectra were recorded on a Bruker ALPHA FT-IR spectrometer (Bruker) in KBr pellets in the range 4,000-400 cm^−1^. The spectra were processed using OPUS software. Melting points were determined on an Electrothermal 9001 melting point apparatus (Electrothermal) (10°C per min) and were uncorrected. Merck KGaA silica gel 60 F254 plates were used for analytical thin-layer chromatography. Spots were detected by a UV lamp. Column chromatography was performed using silica gel Merck 60 (70–230 mesh). Yields refer to purified products and were not optimized.

The scheme and synthetic procedures, as well as ^1^H and ^13^C spectra of the compounds, are presented in Supplemental Data 1.

Supplemental Data 1. Detailed chemical procedures and spectra.

### Molecular docking studies

For the prediction of the protein–ligand complex, we used the program HADDOCK version 2.4 (Dominguez et al, 2003; Van Zundert et al, 2016). Before docking, we generated 3D conformations of the compound starting from its isomeric SMILES (Weininger, 1988; Weininger et al, 1989) with RDKit using the 2020 parameters (only the small aliphatic ring subset) with energy minimization and the ETKDG algorithm (Riniker & Landrum, 2015; Wang et al, 2020). We capped the maximum number of conformers to 50 and provided this ensemble of conformers to HADDOCK for docking analysis. Concurrently, we also employed the program Fpocket (Le Guilloux et al, 2009; Schmidtke et al, 2010), a protein pocket (cavity) detection algorithm based on Voronoi tessellation, and identified the binding pockets of our target protein Rv0164. The top-scored protein pocket of Rv0164 was used for our targeted ligand docking protocol, adapted from the HADDOCK2.4 small molecule binding site screening protocol (Van Zundert et al, 2016). More specifically, two sets of restraints were created to be used at different stages of docking: (a) for the rigid-body docking, we defined the entire binding pocket on the receptor and the ligand as active, and (b) for the subsequent flexible refinement stages, we defined only the binding pocket as passive and the ligand as active. The ligand-specific parameters were used to perform the docking simulation.

### Thermal shift assays

Dh5α *E. coli* strain overexpressing HA-tagged Rv0164 under the lac promoter in a pSMT3 vector (pSMT3-rv0164HA) was inoculated from overnight culture to an OD_600_ of 0.05 in the presence of 10 μM BT-37 or DMSO. Bacteria were grown until they reached an OD_600_ of around 0.6, and protein expression was induced by adding 1 μM IPTG. Bacteria were harvested by centrifugation after 3 h of protein production, and whole cells were subjected to different temperatures ranging from 45 to 70°C in a PCR machine for 3 min. Subsequently, heat-treated cells were lysed by bead beating (100 μm silica beads; BioSpec) for 1 min and were spun at 20,000*g* for 20 min at 4°C to remove cell debris and aggregated proteins. Proteins remaining in the supernatant were denatured with SDS and separated by SDS–PAGE (12.5% polyacrylamide). Gels were transferred to a nitrocellulose membrane (GE Healthcare Life Sciences) and stained with Ponceau dye solution before the membranes were blocked with skim milk. The primary mouse monoclonal antibody anti-HA (1:5,000) and secondary antibody goat anti-mouse IgG conjugated with horseradish peroxidase (1:2,500; American Qualex) were used to detect HA-tagged protein. Visualization of Western blots was done using an ECL substrate (Amersham).

### Construction of plasmids

**Figure S11.**
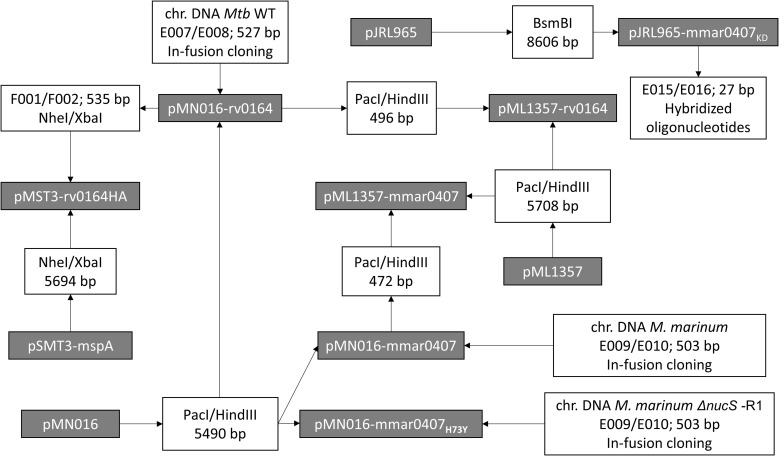
Cloning strategy for new plasmids used in this study. Constructed plasmids are included in gray boxes. The primer pairs used for PCR amplification and correlated restriction enzymes for cloning are listed in white boxes. The DNA template for the PCRs is listed above the primer pairs. If several primer pairs are listed, overlap PCR was used to fuse the PCR fragments. When a DNA fragment was obtained by digestion of a plasmid, the used restriction enzymes and the length of the obtained fragments are indicated. Constructed plasmids with their features and primers with their sequences are listed in Tables S9 and S10, respectively.

All DNA manipulation procedures followed standard molecular cloning techniques. Primers were synthesized and purified by Sigma-Aldrich and are all listed in Table S10. Phusion polymerase, restriction enzymes, and T4 DNA ligase were obtained from New England Biolabs (NEB). iProof polymerase was obtained from Bio-Rad. Where indicated, plasmids were constructed using the ligation-independent cloning method In-Fusion (TaKaRa), following the manufacturer’s recommendations. All plasmids used within this study can be found in Table S9, and cloning experiments are summarized in Fig S11. PCR-amplified inserts were routinely subjected to DNA sequencing (Macrogen).

Table S9. Plasmids used in this work.

Table S10 Oligonucleotides used in this study.

## Supplementary Material

Reviewer comments

## Data Availability

Whole-genome sequencing data have been deposited in the Sequence Read Archive (SRA) within BioProject PRJNA937307 under accession number SRP423768 (BioSample Accession SAMN33399608, SAMN33399607, SAMN33399606, SAMN33399605, SAMN33399604).
