# Supplementary material for: Modulating mycobacterial envelope integrity for antibiotic synergy with benzothiazoles
Source: Life Sci Alliance. 2024 May 14;7(7):e202302509. doi: 10.26508/lsa.202302509 (PMC11094368; doi:10.26508/lsa.202302509)
Supplement: Supplementary file 1 [file LSA-2023-02509_TableS1.docx]

Tables and their legends

**Table S1:** **Results of the targeted screening for benzothiazoles in the ethidium bromide (EtBr) uptake assay and an *M. marinum*-infected zebrafish model (Mmar-ZF).** The fold-difference between the fluorescence signal of EtBr accumulation in compound-treated versus DMSO-treated cultures at 60 min is indicated. The medium used was 7H9, ADS, tyloxapol. The statistical significance between DMSO-treated and compound-treated samples in zebrafish models is indicated as calculated by one-way ANOVA, following Dunnett’s multiple comparison test on log_10_ transformed values. The concentration of compounds used in both assays was 10 µM. The hit compound is highlighted in green.

| **#** | **Structure** | **EtBr assay (**$\frac{\boldsymbol{\lambda compound treated}}{\boldsymbol{\lambda DMSO}}\mathbf{)}$ | **Mmar-ZF** |
| --- | --- | --- | --- |
| BT-01 | 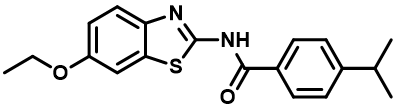 | 6.1 | Not active |
| BT-02 | 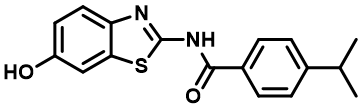 | 3.0 | Not active |
| BT-03 | 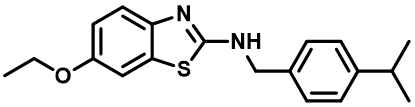 | 1.7 | Not active |
| BT-04 | 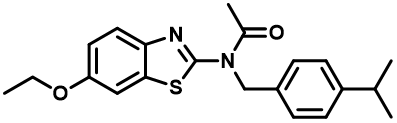 | 2.2 | Not active |
| BT-05 | 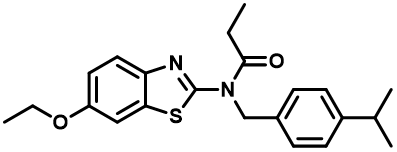 | 5.8 | Not active |
| BT-06 | 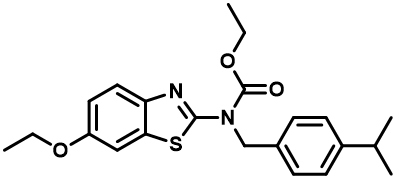 | 0.1 | Not active |
| BT-07 | 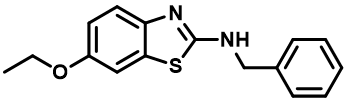 | 11.3 | Not active |
| BT-08 | 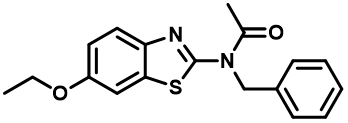 | 26.7 | **Active  (*p* <0.0001)** |
| BT-09 | 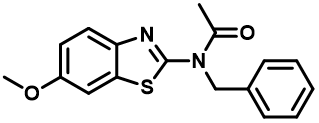 | 5.2 | Not active |
| BT-10 | 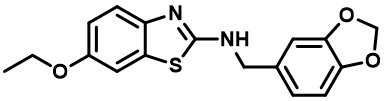 | 5.2 | Not active |
| BT-11 | 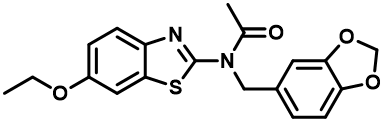 | 3.7 | Not active |
| BT-12 | 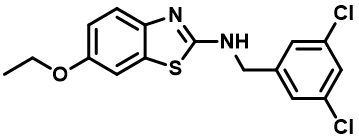 | 4.7 | Not active |
| BT-13 | 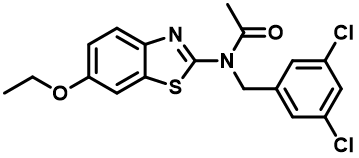 | 0.9 | Not active |
| BT-14 | 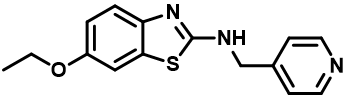 | 5.5 | Not active |
| BT-15 | 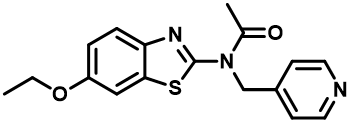 | 1.7 | Not active |
| BT-16 | 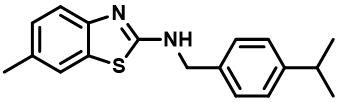 | 0.6 | Not active |
| BT-17 | 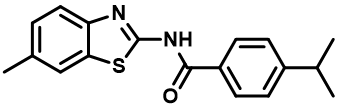 | 1.2 | Not active |
| BT-18 | 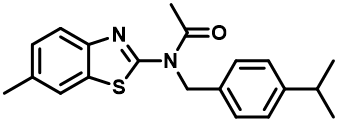 | 1.6 | Not active |
| BT-19 | 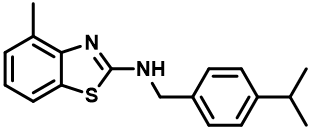 | 0.3 | Not active |
| BT-20 | 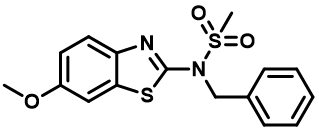 | 5.8 | Not active |
| BT-21 | 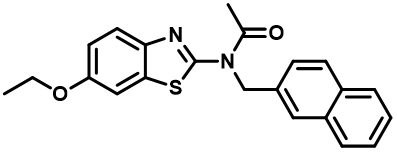 | 5.2 | Not active |
| BT-22 | 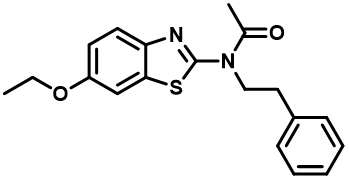 | 6.2 | Not active |
