# Supplementary material for: Modulating mycobacterial envelope integrity for antibiotic synergy with benzothiazoles
Source: Life Sci Alliance. 2024 May 14;7(7):e202302509. doi: 10.26508/lsa.202302509 (PMC11094368; doi:10.26508/lsa.202302509)
Supplement: Supplementary file 2 [file LSA-2023-02509_TableS2.docx]

| **Compound ID** | **Organism** | **MIC_50_** |
| --- | --- | --- |
| BT-08 | *Escherichia coli* K12 | > 40 µM |
| BT-08 | *Bacillus subtilis* 168 | > 40 µM |
| BT-08 | *Klebsiella pneumoniae* LMG20218 | > 40 µM |
| BT-08 | *Acinetobacter baumannii* LMG01041 | > 40 µM |
| BT-08 | *Acinetobacter baumannii* 1757 | > 40 µM |
| BT-08 | *Mycobacterium abscessus* 144C | > 40 µM |
| BT-08 | *Mycobacterium abscessus* RIVM | > 40 µM |
| BT-37 | *Escherichia coli* K12 | > 40 µM |
| BT-37 | *Bacillus subtilis* 168 | > 40 µM |
| BT-37 | *Klebsiella pneumoniae* LMG20218 | > 40 µM |
| BT-37 | *Acinetobacter baumannii* LMG01041 | > 40 µM |
| BT-37 | *Acinetobacter baumannii* 1757 | > 40 µM |
| BT-37 | *Mycobacterium abscessus* 144C | > 40 µM |
| BT-37 | *Mycobacterium abscessus* RIVM | > 40 µM |
| BT-37 | *Mycobacterium smegmatis* MC^2^-155 | > 40 µM |

**Table S2:** **Activity of BT-08 against selected microorganisms.** BT-08 concentration required to inhibit 50% of bacterial growth (MIC_50_) of various bacteria.
