# Supplementary material for: Modulating mycobacterial envelope integrity for antibiotic synergy with benzothiazoles
Source: Life Sci Alliance. 2024 May 14;7(7):e202302509. doi: 10.26508/lsa.202302509 (PMC11094368; doi:10.26508/lsa.202302509)
Supplement: Supplementary file 3 [file LSA-2023-02509_TableS3.docx]

| **Compound ID** | **Organism or cell line** | **LD_50_** |
| --- | --- | --- |
| BT-08 | THP-1 cell line | > 40 µM |
| BT-08 | RAW 264.7 cell line | > 40 µM |
| BT-08 | *Danio rerio* embryos (with 1% DMSO) | > 80 µM |
| BT-37 | THP-1 cell line | > 40 µM |
| BT-37 | RAW 264.7 cell line | > 40 µM |
| BT-37 | *Danio rerio* embryos (with 1% DMSO) | > 80 µM |

**Table S3: Toxicity of BT-08 and BT-37 in cytotoxicity assays and zebrafish embryo toxicity assay.** Median lethal dose (LD_50_) represents the concentration at which 50% of the exposed population dies from the substance's effects.
