# Supplementary material for: Modulating mycobacterial envelope integrity for antibiotic synergy with benzothiazoles
Source: Life Sci Alliance. 2024 May 14;7(7):e202302509. doi: 10.26508/lsa.202302509 (PMC11094368; doi:10.26508/lsa.202302509)
Supplement: Supplementary file 4 [file LSA-2023-02509_TableS4.docx]

**Table S4: Hemolytic activity of compounds BT-08 and BT-37.** Defibrinated sheep blood cells were incubated with compounds BT-08 and BT-37, and hemoglobin release was measured as an indicator of hemolysis.

| **Compound ID** | **Hemolytic activity (%)** |
| --- | --- |
| BT-08 | ˂ 10% |
| BT-37 | ˂ 10% |
