# Supplementary material for: Modulating mycobacterial envelope integrity for antibiotic synergy with benzothiazoles
Source: Life Sci Alliance. 2024 May 14;7(7):e202302509. doi: 10.26508/lsa.202302509 (PMC11094368; doi:10.26508/lsa.202302509)
Supplement: Supplementary file 5 [file LSA-2023-02509_TableS5.docx]

**Table S5: Assessment of drug combinations of BT-08 and different antibiotics**. A symbol ≈ represents estimation and is used for compounds for which MIC_90_ could not be determined, thus, a one-step higher concentration than the highest tested concentration was used as an estimation of MIC_90_. The FIC for each compound was calculated as FIC (A) = MIC_90_(Drug A in combination)/MIC_90_ (Drug A alone) and the FICI as FIC (A) + FIC (B). Based on the obtained ΣFIC value, the drug-to-drug interactions can be determined as: ΣFIC ≤ 0.5 synergism, 0.5 < ΣFIC ≤ 1 additive effect, 1 < ΣFIC ≤ 2 indifference, ΣFIC ˃ 2 antagonism.

| **Strain** | **Compound** | **Mw (g/mol)** | **FIC_abax_** | **FIC _BT-08_** | **ΣFICI** | **Effect** |
| --- | --- | --- | --- | --- | --- | --- |
| ***M. marinum*** | Polymyxin B | 1203.5 | 0.06 | 0.25 | 0.31 | Synergy |
|  | Vancomycin | 1449.3 | 0.13 | 0.25 | 0.38 | Synergy |
|  | Rifampicin | 822.9 | 0.25 | 0.25 | 0.50 | Synergy |
|  | Tetracycline | 444.4 | 0.13 | 0.50 | 0.63 | Additive |
|  | Erythromycin | 733.9 | 0.50 | 0.13 | 0.63 | Additive |
|  | Ethambutol | 204.3 | 0.50 | 0.13 | 0.63 | Additive |
|  | SQ109 | 330.55 | 0.25 | 0.25 | 0.50 | Additive |
|  | Spectinomycin | 332.4 | 0.25 | 0.25 | 0.50 | Additive |
|  | Macozinone | 456.5 | 0.50 | 0.25 | 0.75 | Additive |
|  | Linezolid | 337.3 | 0.50 | 0.25 | 0.75 | Additive |
|  | Fusidic acid | 516.7 | 0.50 | 0.50 | 1.00 | Additive |
|  | Pretomanid | 359.3 | 0.50 | 0.50 | 1.00 | Additive |
|  | Bedaquiline | 555.5 | 0.50 | 1.00 | 1.50 | Indifference |
|  | Cycloserine | 102.1 | 0.50 | 1.00 | 1.50 | Indifference |
|  | Ethionamide | 166.2 | 1.00 | 1.00 | 2.00 | Indifference |
