# Supplementary material for: Modulating mycobacterial envelope integrity for antibiotic synergy with benzothiazoles
Source: Life Sci Alliance. 2024 May 14;7(7):e202302509. doi: 10.26508/lsa.202302509 (PMC11094368; doi:10.26508/lsa.202302509)
Supplement: Supplementary file 6 [file LSA-2023-02509_TableS6.docx]

**Table S6**: **Exploration results of the benzothiazoles' 6-position and side phenyl ring testing on *M. marinum in vitro* (MIC assay – REMA) and in the *M. marinum*-infected zebrafish model (Mmar-ZF).** The medium used for *in vitro* MIC determination was HdB medium with Tween-80**.** In both assays compounds were tested at 10 µM. The statistical significance between DMSO-treated and compound-treated samples in zebrafish models is indicated as calculated by one-way ANOVA, following Dunnett’s multiple comparison test on log_10_ transformed values. Hit compounds are highlighted in green.

| **#** | **Structure** | **Mmar MIC_90_ (µM)** | **Mmar-ZF  (10 µM)** |
| --- | --- | --- | --- |
| **Exploration of the 6-position of the benzothiazole scaffold** | | | |
| BT-23 | 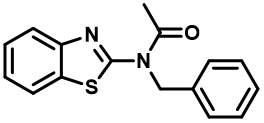 | > 10.0 | Not active |
| BT-24 | 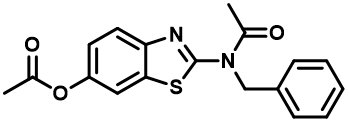 | > 10.0 | Not active |
| BT-25 | 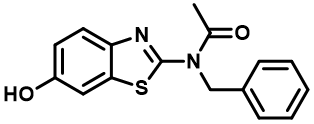 | > 10.0 | Toxic |
| BT-26 | 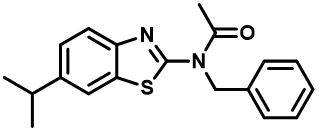 | > 10.0 | Not active |
| BT-27 | 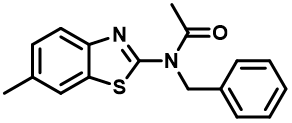 | > 10.0 | Toxic |
| BT-28 | 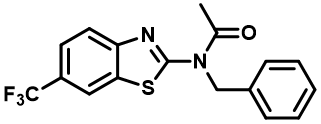 | > 10.0 | Not active |
| BT-29 | 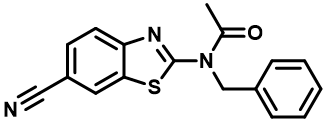 | > 10.0 | Not active |
| BT-30 | 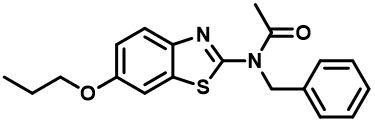 | 2.5 | **Active  (*p* <0.0001)** |
| BT-31 | 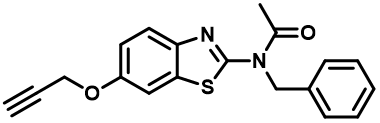 | 5.0 | **Active  (*p* <0.0001)** |
| BT-32 | 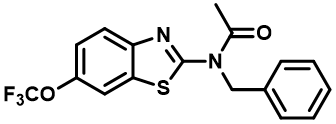 | > 10.0 | Not active |
| BT-33 | 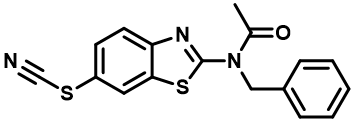 | > 10.0 | Not active |
| **Phenyl ring exploration** | | | |
| BT-34 | 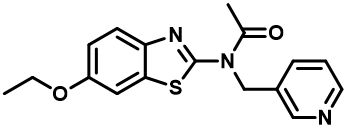 | > 10.0 | Not active |
| BT-35 | 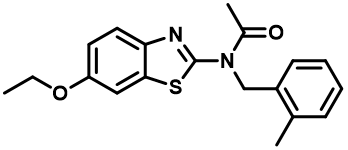 | 0.6 | **Active  (*p* <0.0001)** |
| BT-36 | 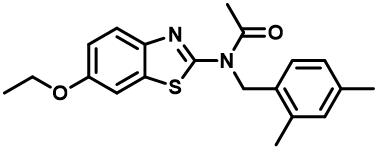 | 0.3 | **Active  (*p* <0.0001)** |
| BT-37 | 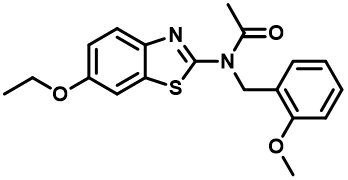 | 0.3 | **Active  (*p* <0.0001)** |
| BT-38 | 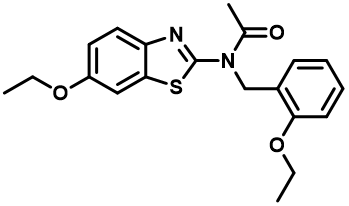 | > 10.0 | Not active |
| BT-39 | 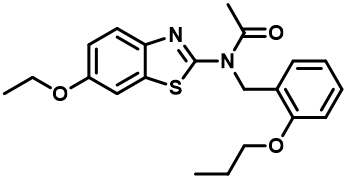 | > 10.0 | Not active |
| BT-40 | 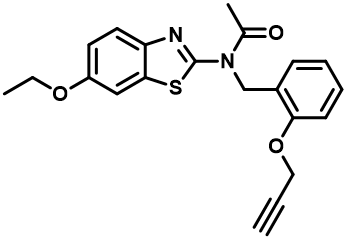 | 2.5 | **Active  (*p* <0.0001)** |
| BT-41 | 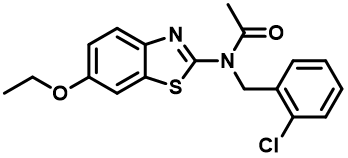 | 1.0 | Not active |
| BT-42 | 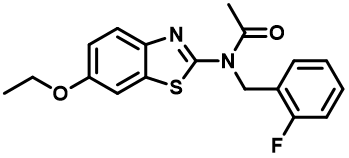 | 2.5 | **Active  (*p* <0.0001)** |
| BT-43 | 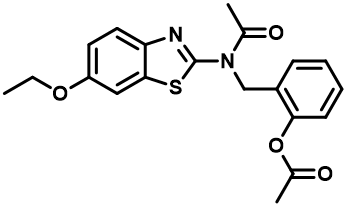 | > 10.0 | Not active |
| BT-44 | 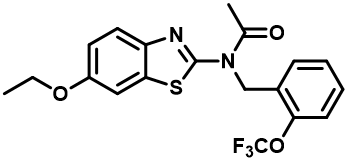 | > 10.0 | Not active |
| BT-45 | 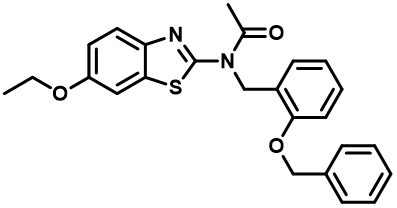 | > 10.0 | Not active |
| BT-46 | 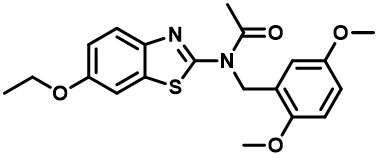 | 0.2 | **Active  (*p* <0.0001)** |
| BT-47 | 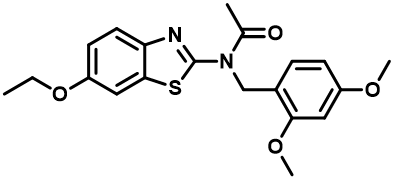 | 2.5 | **Active  (*p* <0.0001)** |
| BT-48 | 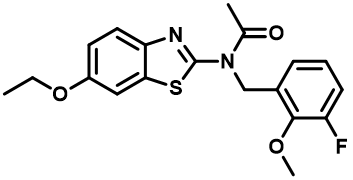 | 0.3 | **Active  (*p* <0.0001)** |
| BT-49 | 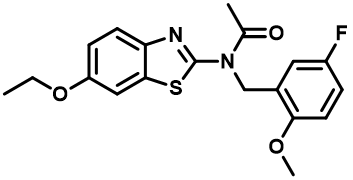 | 1.3 | **Active  (*p* <0.0001)** |
| BT-50 | 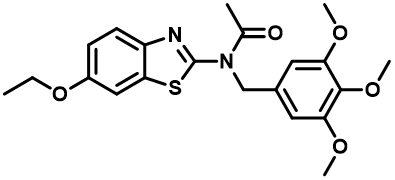 | > 10.0 | Not active |
| BT-51 | 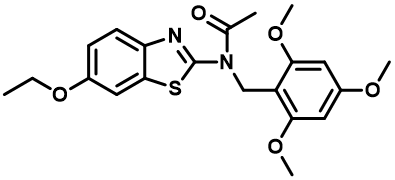 | > 10.0 | Not active |
| BT-52 | 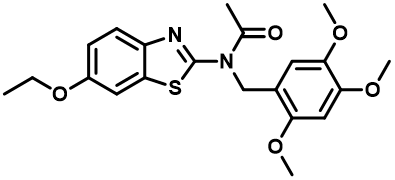 | 2.0 | **Active  (*p* <0.0001)** |
| BT-53 | 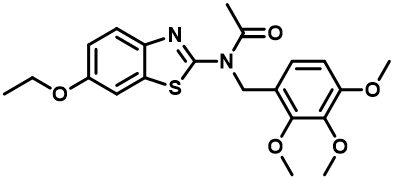 | > 10.0 | Not active |
