# Supplementary material for: Modulating mycobacterial envelope integrity for antibiotic synergy with benzothiazoles
Source: Life Sci Alliance. 2024 May 14;7(7):e202302509. doi: 10.26508/lsa.202302509 (PMC11094368; doi:10.26508/lsa.202302509)
Supplement: Supplementary file 7 [file LSA-2023-02509_TableS7.docx]

**Table S7**: **Single nucleotide polymorphisms identified in BT-37 resistant *M. marinum* Δ*nucS* mutants.** In green are marked mutations unique to all resistant strains. In yellow are marked mutations found in all isolates.

| **Strain** | **Position** | **Count/coverage** | **Frequency** | **Gene mutation** | **Amino acid change** | **Gene** |
| --- | --- | --- | --- | --- | --- | --- |
| Δ*nucS*-R1 | 472293 | 203/203 | 100.0 | G-A | G-S (69) | ***mmar_0407*** |
|  | 820715 | 242/246 | 98.4 | A-G | E-G (158) | *mmar_0688* |
|  | 885839 | 232/232 | 100.0 | G-A | W-stop codon (71) | *mmar_0738* |
|  | 1510305 | 229/229 | 100.0 | T-C | No change | *mmar_1238* |
|  | 1639145 | 262/262 | 100.0 | T-C | S-P (89) | *mmar_1347* |
|  | 1743456 | 235/235 | 100.0 | T-C | L-P (325) | *mmar_1438* |
|  | 2185441 | 174/175 | 99.4 | C-T | R-stop codon (377) | *mmar_1789* |
|  | 3259790 | 214/214 | 100.0 | T-C | V-A (350) | *mmar_2671* |
|  | 3498274 | 251/251 | 100.0 | G-A | No change | *mmar_2893* |
| Δ*nucS*-R2 | 35731 | 235/241 | 97.5 | A-G | Y-H (124) | *mmar_0032* |
|  | 472305 | 211/221 | 95.5 | C-T | H-Y (73) | ***mmar_0407*** |
|  | 1639145 | 216/228 | 94.7 | T-C | S-P (89) | *mmar_1347* |
|  | 1743456 | 202/210 | 96.2 | T-C | L-P (325) | *mmar_1438* |
|  | 2506621 | 251/261 | 96.2 | T-C | L-P (80) | *mmar_2080* |
|  | 4705927 | 259/264 | 98.1 | T-C | No change | *mmar_3798* |
|  | 5582921 | 253/259 | 97.7 | G-A | No change | *mmar_4550* |
|  | 5849071 | 225/241 | 93.4 | T-A | L-H (53) | *mmar_4794* |
| Δ*nucS*-R3 | 35731 | 201/201 | 100.0 | A-G | Y-H (124) | *mmar_0032* |
|  | 472305 | 231/231 | 100.0 | C-T | H-Y (73) | ***mmar_0407*** |
|  | 1151869 | 251/251 | 100.0 | T-C | No change | *mmar_0944* |
|  | 1639145 | 241/242 | 99.6 | T-C | S-P (89) | *mmar_1347* |
|  | 1743456 | 207/207 | 100.0 | T-C | L-P (325) | *mmar_1438* |
|  | 2125127 | 237/238 | 99.6 | T-C | No change | *mmar_1762* |
|  | 2454767 | 174/178 | 97.8 | T-C | I-M (69) | *mmar_2033* |
|  | 2506621 | 211/214 | 98.6 | T-C | L-P (80) | *mmar_2080* |
|  | 4091550 | 168/168 | 100.0 | C-T | No change | *mmar_3316* |
|  | 4705927 | 262/264 | 99.2 | T-C | No change | *mmar_3798* |
|  | 5582921 | 242/244 | 99.2 | G-A | No change | *mmar_4550* |
|  | 5849071 | 221/222 | 99.5 | T-A | L-H (53) | *mmar_4794* |
|  | 6238805 | 246/246 | 100.0 | A-G | V-A (276) | *mmar_5154* |
| Δ*nucS*-R4 | 1639145 | 194/230 | 84.3 | T-C | S-P (89) | *mmar_1347* |
|  | 1743456 | 216/233 | 92.7 | T-C | L-P (325) | *mmar_1438* |
