# Supplementary material for: Modulating mycobacterial envelope integrity for antibiotic synergy with benzothiazoles
Source: Life Sci Alliance. 2024 May 14;7(7):e202302509. doi: 10.26508/lsa.202302509 (PMC11094368; doi:10.26508/lsa.202302509)
Supplement: Supplementary file 8 [file LSA-2023-02509_TableS8.docx]

**Table S8: List of strains used this study.**

| **Strain** | **Features** | **Source** |
| --- | --- | --- |
| *Acinetobacter baumannii* LMG01041 | Laboratory strain | (Li *et al*, 2018) |
| *Bacillus subtilis* 168 | Laboratory strain | ATCC 23857 |
| *Escherichia coli* DH5α | *rec*A1; *end*A1; *gyr*A96; *thi*; *rel*A1; *hsd*R17(r_K_^-^,m_K_^+^); *sup*E44; φ80Δ*lacZ*ΔM15; Δ*lacZ*(YA-argF)UE169 | (Sambrook *et al*, 1989) |
| *Escherichia coli* K12 | Laboratory strain | ATCC 47076 |
| *Klebsiella pneumoniae* LMG20218 | Laboratory strain | (Li *et al*, 2018) |
| *Mycobacterium abscessus* 144C | Clinical isolate, Amsterdam UMC | (Habjan *et al*, 2021) |
| *Mycobacterium abscessus* RIVM | Clinical isolate, Amsterdam UMC | (Habjan *et al*, 2021) |
| *Mycobacterium marinum* M^USA^ | Laboratory strain | ATCC BAA-535 |
| *Mycobacterium marinum* M^USA^ Δ*nucS* | *Mycobacterium marinum* M^USA^, *nucS::loxP* | (Izquierdo Lafuente *et al*) |
| *Mycobacterium marinum ΔnucS* -R1 | *Mycobacterium marinum* M^USA^ Δ*nucS*, *mmar_0407*_G69S_, mutations listed **Table S7** | This study |
| *Mycobacterium marinum ΔnucS* -R2 | *Mycobacterium marinum* M^USA^ Δ*nucS*, *mmar_0407*_H73Y_, mutations listed **Table S7** | This study |
| *Mycobacterium marinum* *ΔnucS* -R3 | *Mycobacterium marinum* M^USA^ Δ*nucS*, *mmar_0407*_H73Y_, mutations listed **Table S7** | This study |
| *Mycobacterium tuberculosis* H37Rv | Laboratory strain | ATCC 25618 |
| *Mycobacterium tuberculosis mc^2^6206* | H37Rv derivative; Δ*panCD*, Δ*leuCD* | (Sampson *et al*, 2004) |
