# Supplementary material for: Modulating mycobacterial envelope integrity for antibiotic synergy with benzothiazoles
Source: Life Sci Alliance. 2024 May 14;7(7):e202302509. doi: 10.26508/lsa.202302509 (PMC11094368; doi:10.26508/lsa.202302509)
Supplement: Supplementary file 9 [file LSA-2023-02509_Supplemental_Data_1.docx]

# **Scheme S1**. Synthesis of the final compounds BT-01-53


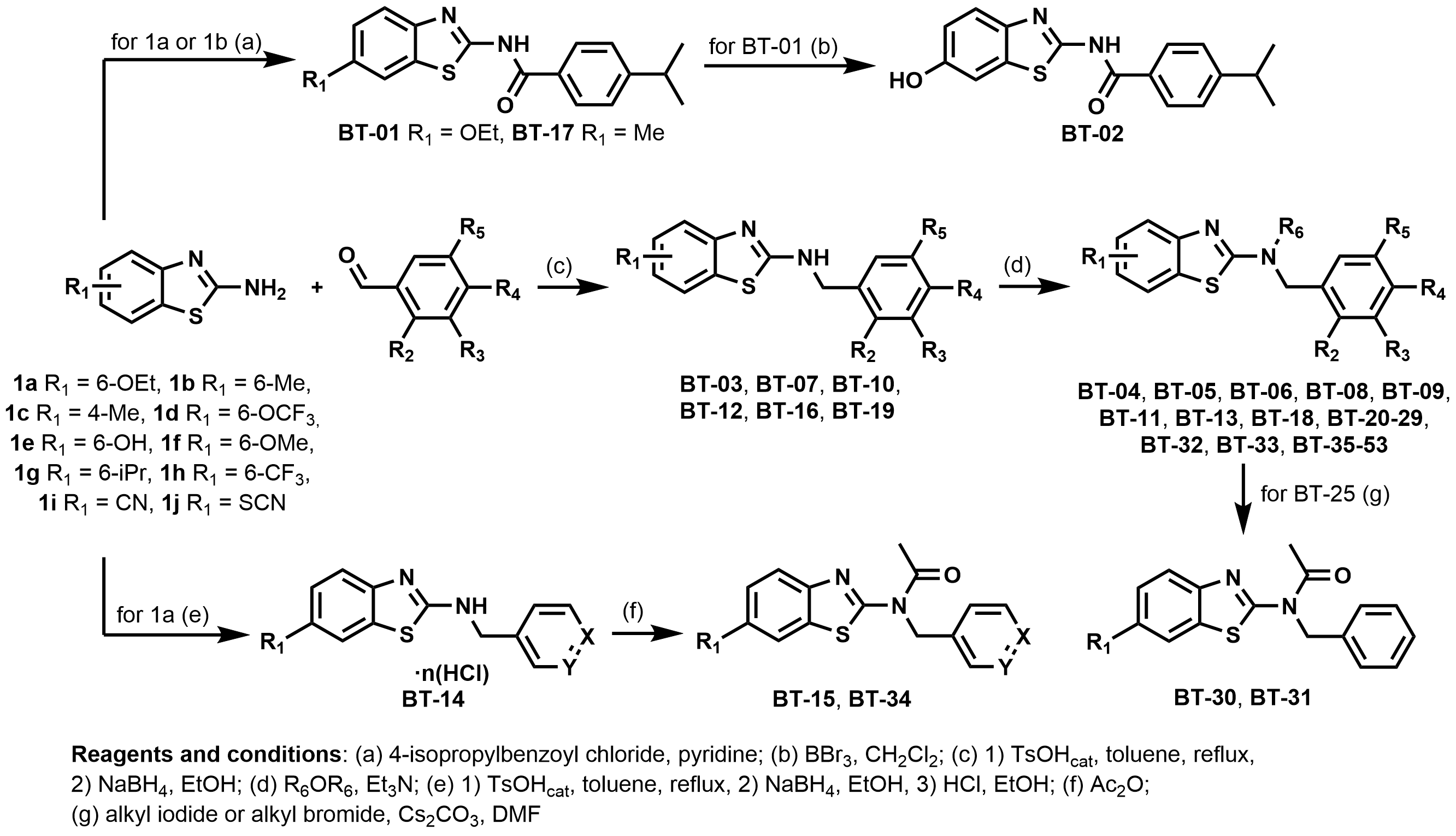


# **Synthetic procedures**

**General procedure for the synthesis of 4-isopropyl-*N*-(6-R-1,3-benzothiazol-2-yl)benzamide BT-01 and BT-17**

A solution of 4-isopropylbenzoyl chloride (403 mg, 2.20 mmol) in acetonitrile (3 mL) is added dropwise to an ice-cold solution of the corresponding 6-*R*-1,3-benzothiazol-2-amine **1** (2.00 mmol) in pyridine (6 mL), and the mixture is stirred at room temperature for 2 h. Then the reaction mixture is cooled with ice water, slowly diluted with water (25 mL) and stirred additional 15 min. The precipitate is filtered, washed with ethanol and water, and crystallized from ethanol to afford the title compounds.

***N*-(6-Ethoxybenzo[*d*]thiazol-2-yl)-4-isopropylbenzamide BT-01**


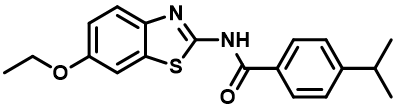
Yield 92 %, mp 192-3 °C. ^1^H NMR (200 MHz; DMSO-d_6_; δ, ppm): 1.23 (d, 6H, *J* = 6.9 Hz, HC(CH_3_)_2_), 1.35 (t, 3H, *J* = 6.9 Hz, CH_3_CH_2_), 2.98 (hept, 1H, *J* = 6.9 Hz, HC(CH_3_)_2_), 4.08 (q, 2H, *J* = 6.9 Hz, CH_3_CH_2_), 7.04 (dd, 1H, *J* = 8.8, 2.4 Hz, HC(5)), 7.42 (d, 2H, *J* = 8.2 Hz, HC(3`, 5`)),7.58 (d, 1H, *J* = 2.4 Hz, HC(7)), 7.66 (d, 1H, *J* = 8.8 Hz, HC(4)), 8.07 (d, 2H, *J* = 8.2 Hz, HC(2`, 6`)), 12.67 (s, 1H, NH). ^13^С NMR (50 MHz; DMSO-d_6_; δ, ppm): 14.66 (CH_3_CH_2_), 23.47 (C(CH_3_)_2_), 33.43 (C(CH_3_)_2_), 63.54 (CH_2_O), 105.21 (C(4)), 115.30 (C(7)), 120.92 (C(5)), 126.55 (C(3`, 5`)),128.37 (C(2`, 6`)), 129.54 (C(1`)), 132.79 (C(7a)), 142.57 (C(3a)), 153.60 (C(4`)),154.39 (C(6)), 155.63 (C(2)), 165.38 (C=O). MS (EI): m/z 340. Anal. calcd for C_19_H_20_N_2_O_2_S: C, 67.03; H, 5.92; N, 8.23. Found: C, 67.09; H, 5.99; N, 8.19.

**4-Isopropyl-*N*-(6-methylbenzo[*d*]thiazol-2-yl)benzamide BT-17**

Yield 91 %, mp 201-2 °C. ^1^H NMR (200 MHz; DMSO-d_6_; δ, ppm): 1.23 (d, 6H, *J* = 6.9 Hz, (H_3_C)_2_CH), 2.43 (s, 3H, H_3_CC(6)), 2.98 (hept, 1H, *J* = 6.9 Hz, (H_3_C)_2_CH), 7.28 (d, 1H, *J* = 8.2 Hz, HC(5)), 7.43 (d, 2H, *J* = 7.8 Hz, HC(3`, 5`)),7.66 (d, 1H, *J* = 8.2 Hz, HC(4)), 7.79 (s, 1H, HC(7)), 8.08 (d, 2H, *J* = 7.8 Hz, HC(2`, 6`)),12.74 (brs, 1H, NH). ^13^С NMR (50 MHz; DMSO-d_6_; δ, ppm): 20.96 (CH_3_C(6)), 23.46 ((H_3_C)_2_CH), 33.44 ((H_3_C)_2_CH), 119.88 (C(4)), 121.24 (C(7)), 126.55 (C(3`, 5`)),127.43 (C(2`, 6`)),128.41 (C(5)), 129.50 (C(7a)), 131.60 (C(6)), 133.05 (C(1`)), 146.36 (C(3a)), 153.64 (C(4`)), 157.96 (C(2)), 165.56 (C=O). MS (EI): m/z 310. Anal. calcd for C_18_H_18_N_2_OS: C, 69.65; H, 5.85; N, 9.02. Found: C, 69.69; H, 5.90; N, 9.07.

**Synthesis of *N*-(6-hydroxy-1,3-benzothiazol-2-yl)-4-isopropylbenzamide BT-02**


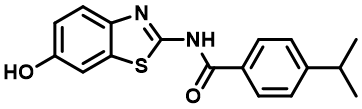
Boron tribromide (2.51 g, 5.00 mmol) is added dropwise to an ice-cold suspension of benzamide **12a** (340 mg, 1.00 mmol) in methylene chloride (5 mL), and the mixture is stirred at room temperature for 3 h and then poured into ice (30 g). After melting of the ice, the organic solvent is evaporated in vacuo, and the precipitate is filtered, washed with water and crystallized from ethanol to afford the title compound. Yield 271 mg (87 %), mp 266-8 °C. ^1^H NMR (200 MHz; DMSO-d_6_; δ, ppm): 1.23 (d, 6H, *J* = 6.9 Hz, HC(CH_3_)_2_), 2.97 (sept, *J* = 6.9 Hz, 1H, HC(CH_3_)_2_), 6.92 (dd, *J* = 8.7, 2.3 Hz, 1H, HC(5)), 7.32 (d, 1H, *J* = 2.3 Hz, HC(7)), 7.43 (d, 2H, *J* = 8.2 Hz, HC(3`, 5`)),7.59 (d, 1H, *J* = 8.7 Hz, HC(4)), 8.06 (d, 2H, *J* = 8.2 Hz, HC(2`, 6`)), 9.60 (brs, 1H, OH), 12.61 (brs, 1H, NH). ^13^С NMR (50 MHz; DMSO-d_6_; δ, ppm): 23.49 (C(CH_3_)_2_), 33.43 (C(CH_3_)_2_), 106.40 (C(4)), 115.25 (C(7)), 120.92 (C(5)), 126.55 (C(3`, 5`)),128.34 (C(2`, 6`)),129.56 (C(1`)),132.80 (C(7a)), 141.45 (C(3a)), 153.54 (C(4`)),154.23 (C(6)), 155.75 (C(2)), 165.28 (C=O). MS (EI): m/z 312. Anal. calcd for C_17_H_16_N_2_O_2_S: C, 65.36; H, 5.16; N, 8.97. Found: C, 65.39; H, 5.21; N, 8.92.

**General procedure for the synthesis of *N*-R^2^,R^3^,R^4^,R^5^-benzyl-R^1^-1,3-benzothiazol-2-amines BT-03, BT-07, BT-10, BT-12, BT-14, BT-16, BT-19**

A mixture of the corresponding R^1^-1,3-benzothiazol-2-amine **1** (1.00 mmol), the corresponding benzaldehyde or nicotinaldehyde (1.25 mmol) and *p*-toluenesulfonic acid (20 mg, 0.116 mmol) in toluene (3 mL) is stirred at reflux with Dean-Stark trap for 30 min. The reaction mixture is cooled and evaporated in vacuo. Sodium borohydride (50 mg, 1.32 mmol) and ethanol (5 mL) are added to the residue, and the resulting mixture is stirred at room temperature for 1 hour, then diluted with water (5 mL) and stirred for additional 30 min. The precipitate is filtered, washed with ethanol and water, and crystallized from ethanol to afford the title compounds.

**6-Ethoxy-*N*-(4-isopropylbenzyl)benzo[*d*]thiazol-2-amine BT-03**

Yield 78 %, mp 138-140 °C. ^1^H NMR (200 MHz; DMSO-d_6_; δ, ppm): 1.18 (d, 6H, *J* = 6.8 Hz, (H_3_C)_2_CH), 1.31 (t, 3H, *J* = 6.9 Hz, CH_3_CH_2_), 2.84 (h, 1H, *J* = 6.9 Hz, HC(1``)), 3.98 (q, 2H, *J* = 6.9 Hz, CH_2_O), 4.50 (d, 2H, *J* = 5.7 Hz, CH_2_N), 6.80 (dd, 1H, *J* = 8.8, 2.6 Hz, HC(5)), 7.13-7.44 (m, 6H, HC(4, 7, 2`, 3`, 5`, 6`)), 8.20 (t, 1H, *J* = 5.8 Hz, NH). ^13^С NMR (50 MHz; DMSO-d_6_; δ, ppm): 14.70 (CH_3_CH_2_), 23.85 (CH_3_CO, (CH_3_)_2_CH), 33.07 (C(1’’)), 46.96 (CH_2_N), 63.48 (CH_2_O), 106.24 (C(7)), 113.42 (C(5)), 118.34 (C(4), 126.19 (C(3`, 5`)), 127.43 (C(2`, 6`)), 131.34 (C(7a)), 146.45 (C(4`)), 147.14 (C(3a)), 153.48 (C(6)), 164.55 (C(2)). MS (EI): m/z 326. Anal. calcd for C_19_H_22_N_2_OS: C, 69.90; H, 6.79; N, 8.58. Found: C, 69.94; H, 6.83; N, 8.62.

***N*-Benzyl-6-ethoxybenzo[*d*]thiazol-2-amine BT-07**

Yield 76 %, mp 131-3 °C. ^1^H NMR (200 MHz; DMSO-d_6_; δ, ppm): 1.31 (t, 3H, *J* = 6.9 Hz, CH_3_CH_2_), 3.98 (q, 2H, *J* = 6.9 Hz, CH_2_O), 4.56 (d, 2H, *J* = 5.8 Hz, CH_2_N), 6.80 (dd, 1H, *J* = 8.7, 2.5 Hz, HC(5)), 7.11-7.47 (m, 7H, Ph, HC(4, 7)), 8.29 (t, 1H, *J* = 5.8 Hz, NH). ^13^С NMR (50 MHz; DMSO-d_6_; δ, ppm): 14.70 (CH_3_CH_2_), 47.11 (CH_2_N), 63.46 (CH_2_O), 106.21 (C(7)), 113.42 (C(5)), 118.37 (C(4)), 126.92 (C(4`)),127.31 (C(2`, 6`)),128.31 (C(3`, 5`)),131.34 (C(7a)), 139.06 (C(1`)),146.38 (C(3a)), 153.51 (C(6)), 164.57 (C(2)). MS (EI): m/z 284. Anal. calcd for C_16_H_16_N_2_OS: C, 67.58; H, 5.67; N, 9.85. Found: C, 67.64; H, 5.71; N, 9.89.


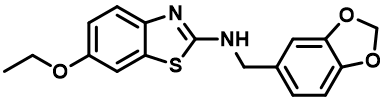
***N*-(Benzo[*d*][1,3]dioxol-5-ylmethyl)-6-ethoxybenzo[*d*]thiazol-2-amine BT-10**

Yield 80 %, mp 156-7 °C. ^1^H NMR (200 MHz; DMSO-d_6_; δ, ppm): 1.31 (t, 3H, *J* = 6.9 Hz, CH_3_CH_2_), 3.99 (q, 2H, *J* = 6.9 Hz, CH_2_O), 4.45 (d, 2H, *J* = 5.7 Hz, CH_2_N), 5.99 (brs, 2H, OCH_2_O), 6.81 (dd, 1H, *J* = 8.8, 2.5 Hz, HC(5)), 6.83-6.90 (m, 2H, HC(6`, 7`)), 6.94 (s, 1H, HC(4`)), 7.28 (d, 1H, *J* = 8.8 Hz, HC(4)), 7.29 (d, 1H, *J* = 2.5 Hz, HC(7)), 8.20 (t, 1H, *J* = 5.7 Hz, NH). ^13^С NMR (50 MHz; DMSO-d_6_; δ, ppm): 14.70 (CH_3_CH_2_), 46.93 (CH_2_N), 63.45 (CH_2_O), 100.81 (C(2`)), 106.20 (C(7)), 107.97 (C(5`)), 108.02 (C(4`)), 113.42 (C(5)), 118.37 (C(4)), 120.61 (C(6`)), 131.32 (C(7a)), 132.89 (C(5`)), 146.17 (C(3a)), 146.38 (C(3a`)),147.20 (C(7a`)),153.51 (C(6)), 164.45 (C(2)). MS (EI): m/z 328. Anal. calcd for C_17_H_16_N_2_O_3_S: C, 62.18; H, 4.91; N, 8.53. Found: C, 62.24; H, 4.98; N, 8.58.

***N*-(3,5-Dichlorobenzyl)-6-ethoxybenzo[*d*]thiazol-2-amine** **BT-12**

Yield 81 %, mp 145-6 °C. ^1^H NMR (200 MHz; DMSO-d_6_; δ, ppm): 1.31 (t, 3H, *J* = 6.9 Hz, CH_3_CH_2_), 3.99 (q, 2H, *J* = 6.9 Hz, CH_2_O), 4.58 (d, 2H, *J* = 5.8 Hz, CH_2_N), 6.82 (dd, 1H, *J* = 8.7, 2.6 Hz, HC(5)), 7.28 (s, 1H, HC(4`)), 7.31 (d, 1H, *J* = 8.7 Hz, HC(4)), 7.37-7.53 (m, 3H, HC(7, 2`, 6`)), 8.30 (t, 1H, *J* = 5.8 Hz, NH). ^13^С NMR (50 MHz; DMSO-d_6_; δ, ppm): 14.67 (CH_3_CH_2_), 46.03 (CH_2_N), 63.56 (CH_2_O), 106.37 (C(7)), 113.64 (C(5)), 118.61 (C(4)), 125.95 C((2`, 6`)), 126.50 (C(4`)), 131.44 (C(1`)), 133.95 (C(3`, 5`)), 143.77 (C(7a)), 146.20 (C(3a)), 153.73 (C(6)), 164.36 (C(2)). MS (EI): m/z 353. Anal. calcd for C_16_H_14_Cl_2_N_2_OS: C, 54.40; H, 3.99; N, 7.93. Found: C, 54.47; H, 4.03; N, 7.98.

**6-Ethoxy-*N*-(pyridin-4-ylmethyl)benzo[*d*]thiazol-2-amine BT-14**

Yield 225 mg (70 %), mp 190-3°C. ^1^H NMR (200 MHz; DMSO-d_6_; δ, ppm): 1.32 (t, 3H, *J* = 6.9 Hz, CH_3_CH_2_), 4.01 (q, 2H, *J* = 6.9 Hz, CH_2_O), 5.11 (brs, 2H, CH_2_N), 6.93 (dd, 1H, *J* = 8.8, 2.4 Hz, HC(5)), 7.39 (d, 1H, *J* = 8.8 Hz, HC(4)), 7.48 (d, 1H, *J* = 2.4 Hz, HC(7)), 8.09 (d, 2H, *J* = 6.5 Hz, HC(3`, 5`)), 8.91 (d, 2H, *J* = 6.5 Hz, HC(2`, 6`)), 10.56 (brs, 1H, NH). ^13^С NMR (50 MHz; DMSO-d_6_; δ, ppm): 14.58 (CH_3_CH_2_), 46.82 (CH_2_N), 63.64 (CH_2_O), 107.19 (C(7)), 114.49 (C(5)), 116.59 (C(4)), 125.11 (C(3`,5`)), 128.32 (C(7a)), 141.38 (C(2`, 6`)), 154.87 (C(4`, 3a)),158.56 (C(6)), 165.77 (C(2)). MS (EI): m/z 285. Anal. calcd for C_15_H_15_N_3_OS: C, 63.13; H, 5.30; N, 14.73. Found: C, 63.19; H, 5.37; N, 14.77.

***N*-(4-Isopropylbenzyl)-6-methylbenzo[*d*]thiazol-2-amine BT-16**

Yield 69 %, mp 155-7 °C. ^1^H NMR (200 MHz; DMSO-d_6_; δ, ppm): 1.18 (d, 6H, *J* = 6.9 Hz, (H_3_C)_2_CH), 2.31 (s, 3H, CH_3_), 2.86 (hept, 1H, *J* = 6.9 Hz, (H_3_C)_2_CH), 4.53 (brs, 2H, NCH_2_), 7.03 (d, 1H, *J* = 8.2 Hz, HC(5)), 7.13-7.40 (m, 5H, HC(4, 2`, 3`, 5`, 6`)), 7.46 (s, 1H, HC(7)), 8.34 (t, 1H, *J* = 5.5 Hz, NH). ^13^С NMR (50 MHz; DMSO-d_6_; δ, ppm): 20.70 (CH_3_C(6)), 23.86 ((H_3_C)_2_CH), 33.08 ((H_3_C)_2_CH), 47.00 (CH_2_N), 117.70 (C(4)), 120.81 (C(7)), 126.19 (C(3`, 5`)), 126.46 (C(2`, 6`)), 127.46 (C(5)), 129.98 (C(7a)), 130.43 (C(6)), 136.29 (C(1`)), 147.17(C(4`)), 150.31 (C(3a)), 165.45 (C(2)). MS (EI): m/z 296. Anal. calcd for C_18_H_20_N_2_S: C, 72.93; H, 6.80; N, 9.45. Found: C, 72.99; H, 6.84; N, 9.37.

***N*-(4-isopropylbenzyl)-4-methylbenzo[*d*]thiazol-2-amine** **BT-19**


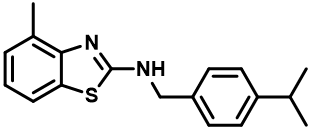
Yield 74 %, mp 80-2 °C. ^1^H NMR (200 MHz; DMSO-d_6_; δ, ppm): 1.18 (d, 6H, *J* = 6.9 Hz, (H_3_C)_2_CH), 2.44 (s, 3H, H_3_CC(4)), 2.86 (sept, 1H, *J* = 6.9 Hz, (H_3_C)_2_CH), 4.53 (d, 2H, *J* = 5.8 Hz, NCH_2_), 6.91 (t, 1H, *J* = 7.6 Hz, HC(6)), 7.05 (d, 1H, *J* = 7.2 Hz, HC(5)), 7.21 (d, 2H, *J* = 8.1 Hz, HC(3`, 5`), 7.33 (d, 2H, *J* = 8.1 Hz, HC(2`, 6`)), 7.47 (d, 1H, *J* = 7.4 Hz, HC(7)), 8.44 (t, 1H, *J* = 5.8 Hz, NH). ^13^С NMR (50 MHz; DMSO-d_6_; δ, ppm): 18.11 (CH_3_C(4)), 23.87 ((H_3_C)_2_CH), 33.10 ((H_3_C)_2_CH), 47.20 (CH_2_N), 118.31 (C(7)), 120.73 (C(6)), 126.19 (С(3`, 5, 5`)),127.16 (C(4)), 127.70 (C(2`, 6`)), 129.77 (C(7a)), 136.26 (C(1`)), 147.20 (C(4`)),151.30 (C(3a)), 165.47 (C(2)). MS (EI): m/z 296. Anal. calcd for C_18_H_20_N_2_S: C, 72.93; H, 6.80; N, 9.45. Found: C, 72.99; H, 6.86; N, 9.38.

**General procedure for the synthesis of *N*-R^2^,R^3^,R^4^,R^5^-benzyl-6-R^1^-*N*-R^6^-1,3-benzothiazol-2-amine** **BT-04-06, BT-08, BT-09, BT-11, BT-13, BT-15, BT-18, BT-20-29, BT-32-53**

A mixture of the corresponding *N*-R^2^,R^3^,R^4^,R^5^-benzyl-R^1^-1,3-benzothiazol-2-amine **2** (0.300 mmol) and the corresponding anhydride (1 mL) is stirred at reflux for 10 min. Water (1.5 mL) is slowly added to the boiling reaction solution, and the resulting mixture stirred at reflux for additional 10 min and then cooled. The precipitate is filtered, washed with ethanol and water.

***N*-(6-Ethoxybenzo[*d*]thiazol-2-yl)-*N*-(4-isopropylbenzyl)acetamide BT-04**

Yield 94 %, mp 115-6°C. ^1^H NMR (200 MHz; DMSO-d_6_; δ, ppm): 1.16 (d, 6H, *J* = 6.9 Hz, HC(CH_3_)_2_), 1.34 (t, 3H, *J* = 6.9 Hz, CH_3_CH_2_), 2.33 (s, 3H, CH_3_CO), 2.84 (hept, 1H, *J* = 6.9 Hz,HC(CH_3_)_2_), 4.06 (q, 2H, *J* = 6.9 Hz, CH_3_CH_2_), 5.52 (s, 2H, CH_2_N), 6.99 (dd, 1H, *J* = 8.8, 2.4 Hz, HC(5)), 7.17 (q, 4H, *J* = 8.1 Hz, HC(2`, 3`, 5`, 6`)), 7.43-7.75 (m, 2H, HC(4,7). ^13^С NMR (50 MHz; DMSO-d_6_; δ, ppm): 171.25 (C=O), 157.32 (C(2)), 155.51 (C(6)), 147.29 (C(4`)), 141.54 (C(3a)), 134.10 (C(7a)), 134.10 (C(1`)), 126.64 (C(2`, 6`)), 125.89 (C(3`, 5`)), 121.55 (C(7)), 115.27 (C(5)), 104.94 (C(7)), 63.55 (CH_2_O), 50.30 (CH_2_N), 32.98 (C(CH_3_)_2_), 23.75 (C(CH_3_)_2_), 22.90 (CH_3_CO), 14.61 (CH_3_CH_2_). MS (EI): m/z 368. Anal. calcd for C_21_H_24_N_2_O_2_S: C, 68.45; H, 6.57; N, 7.60. Found: C, 68.49; H, 6.60; N, 7.63.

***N*-(6-Ethoxybenzo[d]thiazol-2-yl)-*N*-(4-isopropylbenzyl)propionamide BT-05**

Yield 91 %, mp 110-1 °C. ^1^H NMR (200 MHz; DMSO-d_6_; δ, ppm): 1.05 (t, 3H, *J* = 7.1 Hz, H_3_C(3``)), 1.16 (d, 6H, *J* = 6.9 Hz, HC(CH_3_)_2_), 1.35 (t, 3H, *J* = 6.9 Hz, CH_3_CH_2_), 2.64 (q, 2H, *J* = 7.0 Hz, H_2_C(2``)),2.85 (hept, 1H, *J* = 6.9 Hz, HC(CH_3_)_2_), 4.07 (q, 2H, *J* = 6.9 Hz, CH_3_CH_2_), 5.53 (brs, 2H, CH_2_N), 6.99 (dd, 1H, *J* = 8.8, 2.5 Hz, HC(5)), 7.05-7.34 (m, 4H, HC(2`, 3`, 5`, 6`)), 7.44-7.74 (m, 2H, HC(4, 7). ^13^С NMR (50 MHz; DMSO-d_6_; δ, ppm): 8.51 (C(3``)), 14.63 (CH_3_CH_2_), 23.76 (C(CH_3_)_2_), 27.25 (C(2``)), 32.98 (C(CH_3_)_2_), 49.38 (CH_2_N), 63.55 (CH_2_O), 104.90 (C(4)), 115.30 (C(7)), 121.52 (C(5)), 125.79 (C(3`, 5`)),126.64 (C(2`, 6`)),133.98 (C(1`)),134.16 (C(7a)), 141.53 (C(3a)), 147.23 (C(4`)),155.48 (C(6)), 157.54 (C(2)), 174.16 (C=O). MS (EI): m/z 382. Anal. calcd for C_22_H_26_N_2_O_2_S: C, 69.08; H, 6.85; N, 7.32. Found: C, 69.15; H, 6.89; N, 7.27.

**Ethyl ester of (6-ethoxybenzo[*d*]thiazol-2-yl)(4-isopropylbenzyl)carbamic acid BT-06**

Yield 74 %, mp 99-100 °C. ^1^H NMR (200 MHz; DMSO-d_6_; δ, ppm): 0.90-1.67 (m, 12H, HC(CH_3_)_2_, H_3_C(2``), CH_3_CH_2_), 2.85 (hept, 1H, *J* = 6.9 Hz, HC(CH_3_)_2_), 4.06 (q, 2H, *J* = 6.9 Hz, CH_3_CH_2_), 4.31 (q, 2H, *J* = 7.1 Hz, H_2_C(1``)),5.32 (s, 2H, CH_2_N), 6.99 (dd, 1H, *J* = 8.8, 2.4 Hz, HC(5)), 7.22 (q, 4H, *J* = 8.1 Hz, HC(2`, 3`, 5`, 6`)),7.43 (d, 1H, *J* = 2.4 Hz, HC(7)), 7.63 (d, 1H, *J* = 8.8 Hz, HC(4)). ^13^С NMR (50 MHz; DMSO-d_6_; δ, ppm): 14.04 (C(2``)), 14.61 (CH_3_CH_2_), 23.76 (C(CH_3_)_2_), 33.01 (C(CH_3_)_2_), 49.22 (CH_2_N), 63.28 (C(1``), 63.55 (CH_2_O), 105.06 (C(4)), 115.06 (C(7)), 121.41 (C(5)), 126.28 (C(3`, 5`)), 127.18 (C(2`, 6`)), 134.07 (C(1`)), 134.50 (C(7a)), 142.52 (C(3a)), 147.36 (C(4`)),153.75 (C=O), 155.35 (C(6)), 158.43 (C(2)). MS (EI): m/z 398. Anal. calcd for C_22_H_26_N_2_O_3_S: C, 66.31; H, 6.58; N, 7.03. Found: C, 66.39; H, 6.63; N, 7.06.

***N*-Benzyl-*N*-(6-ethoxybenzo[*d*]thiazol-2-yl)acetamide BT-08**

Yield 94 %, mp 133-4 °C. ^1^H NMR (200 MHz; DMSO-d_6_; δ, ppm): 1.35 (t, 3H, *J* = 6.9 Hz, CH_3_CH_2_), 2.32 (s, 3H, CH_3_CO), 4.07 (q, 2H, *J* = 6.9 Hz, CH_2_O), 5.57 (s, 2H, CH_2_N), 6.99 (dd, 1H, *J* = 8.8, 2.6 Hz,HC(5)), 7.09-7.46 (m, 5H, Ph), 7.55 (d, 1H, *J* = 2.5 Hz, HC(7)), 7.62 (d, 1H, *J* = 8.8 Hz, HC(4)). ^13^С NMR (50 MHz; DMSO-d_6_; δ, ppm): 14.62 (CH_3_CH_2_), 22.89 (CH_3_CO), 50.51 (CH_2_N), 63.55 (CH_2_O), 104.96 (C(7)), 115.29 (C(5)), 121.55 (C(4)), 125.89 (C(3`, 5`)),127.18 (C(4`)),128.74 (C(2`, 6`)),134.07 (C(7a)), 136.80 (C(1`)),141.53 (C(3a)), 155.51 (C(6)), 157.33 (C(2)), 171.25 (C=O). MS (EI): m/z 326. Anal. calcd for C_18_H_18_N_2_O_2_S: C, 66.23; H, 5.56; N, 8.58. Found: C, 66.29; H, 5.60; N, 8.61.


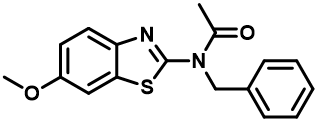
***N*-Benzyl-*N*-(6-methoxybenzo[*d*]thiazol-2-yl)acetamide BT-09**

Yield 86 %, mp 186-7 °C. ^1^H NMR (200 MHz; DMSO-d_6_; δ, ppm): 2.33 (s, 3H, CH_3_CO), 3.82 (s, 3H, CH_3_O), 5.57 (s, 2H, CH_2_N), 7.01 (dd, 1H, *J* = 8.9, 2.7 Hz, HC(5)), 7.18-7.42 (m, 5H, Ph), 7.55 (d, 1H, *J* = 2.7 Hz, HC(7)), 7.63 (d, 1H, *J* = 8.9 Hz, HC(4)). ^13^С NMR (50 MHz; DMSO-d_6_; δ, ppm): 22.88 (CH_3_CO), 50.54 (CH_2_N), 55.60 (CH_3_O), 104.33 (C(7)), 114.94 (C(5)), 121.58 (C(4)), 125.90 (C(2`, 6`)), 127.19 (C(4`)), 128.74 (C(3`, 5`)), 134.07 (C(7a)), 136.80 (C(1`)), 141.62 (C(3a)), 156.33 (C(6)), 157.36 (C(2)), 171.25 (C=O). MS (EI): m/z 312. Anal. calcd for C_17_H_16_N_2_O_2_S: C, 65.36; H, 5.16; N, 8.97. Found: C, 65.41; H, 5.23; N, 9.04.

***N*-(Benzo[*d*][1,3]dioxol-5-ylmethyl)-*N*-(6-ethoxybenzo[*d*]thiazol-2-yl)acetamide BT-11**

Yield 90 %, mp 181-2 °C. ^1^H NMR (200 MHz; DMSO-d_6_; δ, ppm): 1.35 (t, 3H, *J* = 6.9 Hz, CH_3_CH_2_), 2.34 (s, 3H, CH_3_CO), 4.07 (q, 2H, *J* = 6.9 Hz, CH_2_O), 5.48 (brs, 2H, CH_2_N), 5.99 (brs, 2H, H_2_C(2`)), 6.68 (d, 1H, *J* = 8.8 Hz, HC(7`)), 6.78-6.94 (m, 2H, HC(4`, 6`)), 7.01 (dd, 1H, *J* = 8.8, 2.4 Hz, HC(5)), 7.55 (d, 1H, *J* = 2.4 Hz, HC(7)), 7.65 (d, 1H, *J* = 8.8 Hz, HC(4)). ^13^С NMR (50 MHz; DMSO-d_6_; δ, ppm): 14.62 (CH_3_CH_2_), 22.91 (CH_3_CO), 50.28 (CH_2_N), 63.58 (CH_2_O), 101.02 (C(2``)), 105.00 (C(7)), 106.82 (5`),108.40 (C(4`)), 115.31 (C(5)), 119.21 (C(4)), 121.57 (C(6`)), 130.56 (C(7a)), 134.07 (C(5`)), 141.53 (C(3a)), 146.41 (C(3a`)), 147.57 (C(7a`)),155.54 (C(6)), 157.41 (C(2)), 171.24 (C=O). MS (EI): m/z 370. Anal. calcd for C_19_H_18_N_2_O_4_S: C, 61.61; H, 4.90; N, 7.56. Found: C, 61.68; H, 4.98; N, 7.60.

***N*-(3,5-Dichlorobenzyl)-*N*-(6-ethoxybenzo[*d*]thiazol-2-yl)acetamide BT-13**

Yield 93 %, mp 178-9 °C. ^1^H NMR (200 MHz; DMSO-d_6_; δ, ppm): 1.35 (t, 3H, *J* = 6.9 Hz, CH_3_CH_2_), 2.34 (s, 2H, CH_3_CO), 4.07 (q, 2H, *J* = 6.9 Hz, CH_2_O), 5.54 (brs, 2H, CH_2_N), 7.00 (dd, 1H, *J* = 8.8, 2.5 Hz, HC(5)), 7.29 (d, 2H, *J* = 1.6 Hz, HC(2`, 6`)),7.51 (d, 1H, *J* = 1.6 Hz, HC(4`)),7.55 (d, 1H, *J* = 2.5 Hz, HC(7)), 7.62 (d, 1H, *J* = 8.8 Hz, HC(4)). ^13^С NMR (50 MHz; DMSO-d_6_; δ, ppm): 14.61 (CH_3_CH_2_), 22.98 (CH_3_CO), 49.86 (CH_2_N), 63.55 (CH_2_O), 105.00 (C(7)), 115.39 (C(5)), 121.62 (C(4)), 124.91 C((2`, 6`)), 127.01 (C(4`)), 134.07 (C(1`)), 134.35 (C(3`, 5`)), 141.36 (C(7a, 3a)), 155.60 (C(6)), 157.13 (C(2)), 171.18 (C=O). MS (EI): m/z 395. Anal. calcd for C_18_H_16_Cl_2_N_2_O_2_S: C, 54.69; H, 4.08; N, 7.09. Found: C, 54.75; H, 4.12; N, 7.12.

***N*-(6-Ethoxybenzo[*d*]thiazol-2-yl)-*N*-(pyridin-4-ylmethyl)acetamide BT-15**

Yield 78 mg (77 %), mp 120-1 °C. ^1^H NMR (200 MHz; DMSO-d_6_; δ, ppm): 1.34 (t, 3H, *J* = 6.9 Hz, CH_3_CH_2_), 2.32 (s, 3H, CH_3_CO), 4.06 (q, 2H, *J* = 6.9 Hz, CH_2_O), 5.11 (brs, 2H, CH_2_N), 6.98 (dd, 1H, *J* = 8.9, 2.5 Hz, HC(5)), 7.24 (d, 2H, *J* = 5.5 Hz, HC(3`, 5`)), 7.42-7.74 (m, 2H, HC(4, 7)), 8.53 (d, 2H, *J* = 5.6 Hz, HC(2`, 6`)). ^13^С NMR (50 MHz; DMSO-d_6_; δ, ppm): 14.61 (CH_3_CH_2_), 22.83 (CH_3_CO), 49.90 (CH_2_N), 63.56 (CH_2_O), 104.97 (C(7)), 115.33 (C(5)), 121.09 (C(4)), 121.59 (C(3`,5`)), 134.07 (C(7a)), 141.45 (C(2`, 6`)), 145.96 (C(4`)), 149.90 (C(3a)), 155.57 (C(6)), 157.06 (C(2)), 171.10 (C=O). MS (EI): m/z 327. Anal. calcd for C_17_H_17_N_3_O_2_S: C, 62.37; H, 5.23; N, 12.83. Found: C, 62.41; H, 5.28; N, 12.88.


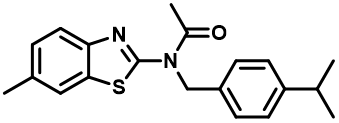
***N*-(4-Isopropylbenzyl)-*N*-(6-methylbenzo[*d*]thiazol-2-yl)acetamide BT-18**

Yield 90 %, mp 146-7 °C. ^1^H NMR (200 MHz; DMSO-d_6_; δ, ppm): 1.16 (d, 6H, *J* = 6.9 Hz, (H_3_C)_2_CH), 2.34 (s, 3H, CH_3_CO), 2.41 (s, 3H, H_3_CC(4)), 2.84 (sept, 1H, *J* = 6.9 Hz, (H_3_C)_2_CH), 5.54 (s, 2H, NCH_2_), 7.10-7.26 (m, 5H, HC(5, 2`, 3`, 5`, 6`)), 7.61 (d, 1H, *J* = 8.2 Hz,HC(4)), 7.77 (s, 1H, HC(7)). ^13^С NMR (50 MHz; DMSO-d_6_; δ, ppm): 20.95 (CH_3_C(6)), 22.97 (CH_3_CO), 23.76 ((H_3_C)_2_CH), 32.98 ((H_3_C)_2_CH, 50.35 (CH_2_N), 120.55 (C(4)), 121.00 (C(7)), 125.89 (C(3`, 5`)), 126.65 (C(2`, 6`)), 127.37 (C(5)), 132.89 (C(7a)), 133.29 (C(6)), 134.08 (C(1`)), 145.47(C(4`)), 147.30 (C(3a)), 158.45 (C(2)), 171.43 (C=O). MS (EI): m/z 338. Anal. calcd for C_20_H_22_N_2_OS: C, 70.97; H, 6.55; N, 8.28. Found: C, 71.03; H, 6.51; N, 8.34.

***N*-Benzyl-*N*-(6-methoxybenzo[*d*]thiazol-2-yl)methanesulfonamide BT-20**


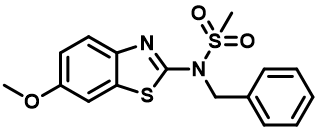
Yield 83 %, mp 128-9 °C. ^1^H NMR (200 MHz; DMSO-d_6_; δ, ppm): 3.38 (s, 3H, CH_3_S). 3.78 (s, 3H, CH_3_O), 5.24 (brs, 2H, CH_2_N), 7.02 (dd, 1H, *J* = 8.9, 2.6 Hz, HC(5)), 7.20-7.46 (m, 5H, Ph), 7.56 (d, 1H, *J* = 2.5 Hz, HC(7)), 7.68 (d, 1H, *J* = 9.0 Hz, HC(4)). ^13^С NMR (50 MHz; DMSO-d_6_; δ, ppm): 39.38 (CH_3_S), 52.20 (CH_2_N), 55.63 (CH_3_O), 104.78 (C(7)), 114.94 (C(5)), 121.82 (C(4)), 127.52 (C(2`, 4`, 6`)), 128.43 (C(3`, 5`)), 134.01 (C(7a)), 136.41 (C(1`)), 143.26 (C(3a)), 156.57 (C(6)), 158.09 (C(2)). MS (EI): m/z 348. Anal. calcd for C_16_H_16_N_2_O_3_S_2_: C, 55.15; H, 4.63; N, 8.04. Found: C, 55.23; H, 4.69; N, 8.08.


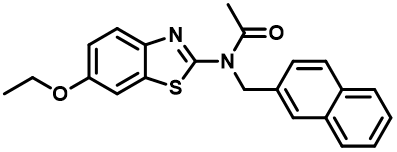
***N*-(6-Ethoxybenzo[*d*]thiazol-2-yl)-*N*-(naphthalen-2-ylmethyl)acetamide BT-21**

Yield 87 %, mp 183-4 °C. ^1^H NMR (200 MHz; DMSO-d_6_; δ, ppm): 1.35 (t, 3H, *J* = 6.9 Hz, CH_3_CH_2_). 2.38 (s, 3H, CH_3_CO), 4.08 (q, 2H, *J* = 7.0 Hz, CH_2_O), 5.73 (brs, 2H, CH_2_N), 6.99 (dd, 1H, *J* = 8.7, 2.6 Hz, HC(5)), 7.38-7.65 (m, 5H, HC(7, 3`, 5`, 6`, 7`)), 7.71 (brs, 1H, HC(1`)), 7.80-7.95 (m, 3H, HC(4, 4`, 8`)). ^13^С NMR (50 MHz; DMSO-d_6_; δ, ppm): 14.61 (CH_3_CH_2_), 22.94 (CH_3_CO), 50.82 (CH_2_N), 63.57 (CH_2_O), 105.00 (C(7)), 115.28 (C(5)), 121.58 (C(4)), 124.00 (C(6)), 124.46 (C(7`)), 125.89 (C(1`)), 126.34 (C(3`)), 127.50 (C(4`)), 127.61 (C(8`)), 128.46 (C(5`)), 132.16 (C(3a)), 132.86 (C(4a)), 134.13 (C(8a)), 134.47 (C(2`)), 141.56 (C(3a)), 155.54 (C(6)), 157.39 (C(2)), 171.34 (C=O). MS (EI): m/z 376. Anal. calcd for C_22_H_20_N_2_O_2_S: C, 70.19; H, 5.35; N, 7.44. Found: C, 70.25; H, 5.28; N, 7.37.


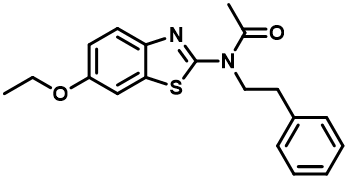
***N*-(6-Ethoxybenzo[*d*]thiazol-2-yl)-*N*-phenethylacetamide BT-22**

Yield 33 %, mp 116-7 °C. ^1^H NMR (200 MHz; DMSO-d_6_; δ, ppm): 1.35 (t, 3H, *J* = 7.0 Hz, CH_3_CH_2_), 2.21 (s, 3H, CH_3_CO), 3.08 (t, 2H, *J* = 7.4 Hz, H_2_C(2``)), 4.07 (q, 2H, *J* = 7.0 Hz, CH_2_O), 4.38 (t, 2H, *J* = 7.4 Hz, H_2_C(1``)), 7.04 (dt, 1H, *J* = 8.9, 1.9 Hz, HC(5)), 7.14-7.42 (m, 5H, Ph), 7.54 (t, 1H, *J* = 2.0 Hz, HC(7)), 7.74 (dd, 1H, *J* = 8.8, 1.5 Hz, HC(4)). ^13^С NMR (50 MHz; DMSO-d_6_; δ, ppm): 14.64 (CH_3_CH_2_), 22.49 (CH_3_CO), 33.37 (C(2``)), 49.45 (C(1``)), 63.55 (CH_2_O), 104.89 (C(7)), 115.21 C(5)), 121.64 (C(4)), 126.52 (C(4`)), 128.49 (C(2`, 6`)), 128.91 (C(3`, 5`)), 133.86 (C(7a)), 138.35 (C(1`)), 141.90 (C(3a)), 155.48 (C(6)), 156.48 (C(2)), 170.82 (C=O). MS (EI): m/z 340. Anal. calcd for C_19_H_20_N_2_O_2_S: C, 67.03; H, 5.92; N, 8.23. Found: C, 67.11; H, 5.99; N, 8.16.

***N*-(Benzo[*d*]thiazol-2-yl)-*N*-benzylacetamide BT-23**

Yield 80 %, mp 135-6 °C. ^1^H NMR (200 MHz; DMSO-d_6_; δ, ppm): 2.34 (s, 3H, CH_3_CO), 5.61 (s, 2H, CH_2_N), 7.18-7.52 (m, 7H, HC(5, 6, Ph), 7.74 (d, *J* = 7.9 Hz, 1H, HC(7)), 7.99 (dd, *J* = 7.9, 1.5 Hz, 1H, HC(4)). ^13^С NMR (50 MHz; DMSO-d_6_; δ, ppm): 22.97 (CH_3_CO), 50.63 (CH_2_N), 120.91 (C(4)), 121.46 (C(7)), 123.82 (C(6)), 125.91 (C(2`, 6`)), 126.04 (C(5)), 127.19 (C(4`)), 128.74 (C(3`, 5`)), 132.74 (C(7a)), 136.71 (C(1`)), 147.47 (C(3a)), 159.30 (C(2)), 171.58 (C=O). MS (EI): m/z 282. Anal. calcd for C_16_H_14_N_2_OS: C, 68.06; H, 5.00; N, 9.92. Found: C, 68.11; H, 5.07; N, 9.86.

**2-(*N*-Benzylacetamido)benzo[*d*]thiazol-6-yl acetate BT-24**


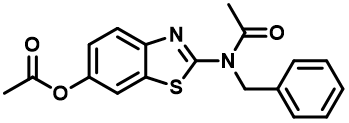
Yield 1.43 g (84 %), mp 189-192 °C. ^1^H NMR (200 MHz; DMSO-d_6_; δ, ppm): 2.29 (s, 3H, CH_3_CON), 2.34 (s, 3H, CH_3_COO), 5.60 (brs, 2H, CH_2_N), 7.03-7.51 (m, 6H, Ph, HC(5)), 7.74 (d, 1H, *J* = 8.7 Hz, HC(4)), 7.81 (d, 1H, *J* = 2.2 Hz, HC(7)). ^13^С NMR (50 MHz; DMSO-d_6_; δ, ppm): 20.79 (CH_3_COO), 22.92 (CH_3_CON), 50.60 (CH_2_N), 114.67 (C(7)), 120.64 (C(5)), 121.34 (C(4)), 125.89 (C(3`, 5`)), 127.22 (C(4`)), 128.77 (C(2`, 6`)), 133.37 (C(7a)), 136.61 (C(1`)), 145.28 (C(3a)), 146.63 (C(6)), 159.57 (C(2)), 169.49 (OC=O), 171.70 (NC=O). MS (EI): m/z 340. Anal. calcd for C_18_H_16_N_2_O_3_S: C, 63.51; H, 4.74; N, 8.23. Found: C, 63.58; H, 4.79; N, 8.20.

***N*-Benzyl-*N*-(6-hydroxybenzo[*d*]thiazol-2-yl)acetamide BT-25**

Yield 680 mg (68 %), mp 175-6°C. ^1^H NMR (200 MHz; DMSO-d_6_; δ, ppm): 2.31 (s, 3H, CH_3_CO), 5.55 (brs, 2H, NCH_2_), 6.87 (dd, 1H, *J* = 8.7, 2.4 Hz, HC(5)), 7.30 (m, 6H, Ph, HC(7))), 7.54 (d, 1H, *J* = 8.7 Hz, HC(7)), 9.60 (brs, 1H, OH). ^13^С NMR (50 MHz; DMSO-d_6_; δ, ppm): 22.90 (CH_3_CO), 50.47 (CH_2_N), 106.06 (C(7)), 115.25 (C(5)), 121.58 (C(4)), 125.88 (C(3`, 5`)), 127.16 (C(4`)), 128.74 (C(2`, 6`)), 134.10 (C(7a)), 136.86 (C(1`)), 140.59 (C(3a)), 154.37 (C(6)), 156.51 (C(2)), 171.13 (C=O). MS (EI): m/z 298. Anal. calcd for C_16_H_14_N_2_O_2_S: C, 64.41; H, 4.73; N, 9.39. Found: C, 64.48; H, 4.78; N, 9.43.

***N*-Benzyl-*N*-(6-isopropylbenzo[*d*]thiazol-2-yl)acetamide BT-26**

Yield 84 %, mp 149-50 °C. ^1^H NMR (200 MHz; DMSO-d_6_; δ, ppm): 1.24 (d, 6H, *J* = 7.0 Hz, (CH_3_)_2_CH), 2.33 (s, 3H, CH_3_CO), 3.00 (p, 1H, *J* = 6.8 Hz, HC(1``)), 5.59 (s, 2H, CH_2_N), 7.19-7.41 (m, 6H, HC(5), Ph), 7.65 (d, 1H, *J* = 8.3 Hz, HC(4)), 7.84 (d, 1H, *J* = 1.8 Hz, HC(7)). ^13^С NMR (50 MHz; DMSO-d_6_; δ, ppm): 22.95 (CH_3_CO), 24.10 ((CH_3_)_2_C(1``)), 33.47 (C(1``)), 50.54 (CH_2_N), 118.46 (C(4)), 120.66 (C(7)), 124.91 (C(2`, 6`)), 125.89 (C(5)), 127.19 (C(4`)), 128.73 (C(3`, 5`)), 132.92 (C(7a)), 136.77 (C(1`)), 144.53 (C(6)), 145.75 (C(3a)), 158.66 (C(2)), 171.40 (C=O). MS (EI): m/z 324. Anal. calcd for C_19_H_20_N_2_OS: C, 70.34; H, 6.21; N, 8.63. Found: C, 70.41; H, 6.33; N, 8.56.

***N*-Benzyl-*N*-(6-methylbenzo[*d*]thiazol-2-yl)acetamide BT-27**

Yield 89 %, mp 189-90 °C. ^1^H NMR (200 MHz; DMSO-d_6_; δ, ppm): 2.34 (s, 3H, CH_3_CO), 2.42 (s, 3H, H_3_CC(6)), 5.59 (s, 2H, CH_2_N), 7.19-7.41 (m, 6H, HC(5), Ph), 7.62 (d, 1H, *J* = 8.1 Hz, HC(4)), 7.75 (s, 1H, HC(7)). ^13^С NMR (50 MHz; DMSO-d_6_; δ, ppm): 20.94 (CH_3_C(6)), 22.94 (CH_3_CO), 50.57 (CH_2_N), 120.55 (C(4)), 121.00 (C(7)), 125.89 (C(2`, 6`)), 127.19 (C(4’)), 127.37 (C(5)), 128.74 (C(3`, 5`)), 132.89 (C(7a)), 133.32 (C(1`)), 136.77 (C(6)), 145.47 (C(3a)), 158.48 (C(2)), 171.43 (C=O). MS (EI): m/z 296. Anal. calcd for C_17_H_16_N_2_OS: C, 68.89; H, 5.44; N, 9.45. Found: C, 68.93; H, 5.32; N, 9.52.

***N*-Benzyl-*N*-(6-(trifluoromethyl)benzo[*d*]thiazol-2-yl)acetamide BT-28**

Yield 79 %, mp 184-5 °C. ^1^H NMR (200 MHz; DMSO-d_6_; δ, ppm): 2.38 (s, 3H, CH_3_CO). 5.64 (s, 2H, CH_2_N), 7.16-7.43 (m, 5H, Ph), 7.71 (dd, 1H, *J* = 8.7, 2.0 Hz, HC(5)), 7.90 (d, 1H, *J* = 8.4 Hz, HC(4)), 8.51 (s, 1H, HC(7)). ^13^С NMR (50 MHz; DMSO-d_6_; δ, ppm): 22.94 (CH_3_CO), 50.78 (CH_2_N), 119.70 (m, C(7)), 121.37 (C(4)), 122.76 (C(5)), 124.0 (q, *J* = 31.6 Hz, C(6)), 125.14 (q, *J* = 122 Hz, CF_3_), 125.95 (C(2`, 6`)), 127.28 (C(4`)), 128.80 (C(3`, 5`)), 133.28 (C(7a)), 136.44 (C(1`)), 150.14 (C(3a)), 162.33 (C(2)), 172.10 (C=O). MS (EI): m/z 350. Anal. calcd for C_17_H_13_F_3_N_2_OS: C, 58.28; H, 3.74; N, 8.00. Found: C, 58.35; H, 3.82; N, 8.09.

**
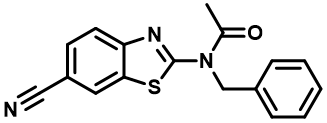
*N*-Benzyl-*N*-(6-cyanobenzo[*d*]thiazol-2-yl)acetamide BT-29**

Yield 88 %, mp 181-2 °C. ^1^H NMR (200 MHz; DMSO-d_6_; δ, ppm): 2.39 (s, 3H, CH_3_), 5.64 (brs, 2H, CH_2_N), 7.00-7.58 (m, 5H, Ph), 7.80 (dd, 1H, *J* = 8.3, 1.6 Hz, HC(5)), 7.88 (d, 1H, *J* = 8.4 Hz, HC(4)), 8.59 (d, 1H, *J* = 1.6 Hz, HC(7)). ^13^С NMR (50 MHz; DMSO-d_6_; δ, ppm): 22.95 (CH_3_), 50.86 (CH_2_N), 105.66 (C(6)), 119.10 (CN), 121.61 (C(4`)), 125.95 (C(2`, 6`)), 126.92 (C(7)), 127.34 (C(4)), 128.80 (C(3`, 5`)), 129.46 (C(5)), 133.47 (C(7a)), 136.32 (C(1)), 150.51 (C(3a)), 162.94 (C(2)), 172.22 (C=O). MS (EI): m/z 307. Anal. calcd for C_17_H_13_N_3_OS: C, 66.43; H, 4.26; N, 13.67. Found: C, 66.52; H, 4.34; N, 13.61.

***N*-Benzyl-*N*-(6-(trifluoromethoxy)benzo[*d*]thiazol-2-yl)acetamide BT-32**


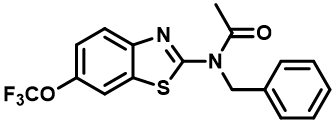
Yield 81 %, mp 135-6 °C. ^1^H NMR (200 MHz; DMSO-d_6_; δ, ppm): 2.37 (s, 3H, CH_3_), 5.62 (s, 2H, CH_2_N), 7.07-7.53 (m, 6H, HC(5), Ph), 7.83 (d, 1H, *J* = 8.8 Hz, HC(4)), 8.14 (d, 1H, *J* = 2.5 Hz, HC(7)). ^13^С NMR (50 MHz; DMSO-d_6_; δ, ppm): 22.91 (CH_3_), 50.71 (CH_2_N), 114.76 (C(7)), 119.79 (C(4), 120.16 (q, *J* = 255 Hz, CF_3_)), 122.00 (C(5)), 125.94 (C(2`, 6`)), 127.25 (C(4`)), 128.77 (C(3`, 5`)), 133.99 (C(7a)), 136.53 (C(1`)), 144.27 (C(3a)), 146.47 (C(6)), 160.76 (C(2)), 171.89 (C=O). MS (EI): m/z 366. Anal. calcd for C_17_H_17_F_3_N_2_O_2_S: C, 55.73; H, 3.58; N, 7.65. Found: C, 55.82; H, 3.65; N, 7.56.

***N*-Benzyl-*N*-(6-thiocyanatobenzo[*d*]thiazol-2-yl)acetamide BT-33**

Yield 52 %, mp 175-6 °C. ^1^H NMR (200 MHz; DMSO-d_6_; δ, ppm): 2.37 (s, 3H, CH_3_CO), 5.61 (s, 2H, CH_2_N), 7.19-7.43 (m, 5H, Ph), 7.67 (dd, 1H, *J* = 8.6, 2.1 Hz, HC(5)), 7.85 (d, 1H, *J* = 8.6 Hz, HC(4)), 8.42 (d, 1H, *J* = 2.0 Hz, HC(7)). ^13^С NMR (50 MHz; DMSO-d_6_; δ, ppm): 22.94 (CH_3_CO), 50.76 (CH_2_N), 114.03 (C(7)), 118.25 (CN), 122.43 (C(4)), 125.40 (C(4`)), 125.92 (C(2`, 6`)), 127.28 (C(5)), 128.79 (C(3`, 5`)), 129.28 (C(6)), 134.58 (C(7a)), 136.44 (C(1`)), 148.66 (C(3a)), 161.45 (C(2)), 172.04 (C=O). MS (EI): m/z 339. Anal. calcd for C_17_H_13_N_3_O_2_S: C, 60.16; H, 3.86; N, 12.38. Found: C, 60.24; H, 3.93; N, 12.45.

***N*-(6-Ethoxybenzo[*d*]thiazol-2-yl)-*N*-(pyridin-3-ylmethyl)acetamide BT-34**

Yield 90 %, mp 162-4 °C. ^1^H NMR (200 MHz; DMSO-d_6_; δ, ppm): 1.35 (t, 3H, *J* = 6.9 Hz, CH_3_CH_2_), 2.38 (s, 3H, CH_3_CO), 4.06 (q, 2H, *J* = 6.9 Hz, CH_2_O), 5.57 (s, 2H, NCH_2_)), 7.00 (dd, 1H, *J* = 8.8, 2.3 Hz, HC(5)), 7.37-7.43 (dd, 1H, *J* = 7.7, 4.6 Hz, HC(5`)), 7.48-7.90 (m, 3H, HC(4, 7, 4`)), 8.50 (d, 1H, *J* = 4.7 Hz, HC(6`)), 8.57 (s, 1H, HC(2`)).^13^С NMR (50 MHz; DMSO-d_6_; δ, ppm): 14.62 (CH_3_CH_2_), 22.98 (CH_3_CO), 48.60 (NCH_2_), 63.54 (CH_2_O), 104.93 (C(7)), 115.33 (C(5)), 121.61 (C(4)), 123.74 (C5`)), 132.49 (C(4`)), 134.04 (C(7a, 3`)), 141.47 (C(3a)), 147.96 (C(6`)), 148.51 (C(2`)), 155.56 (C(6)), 157.13 (C(2)), 171.09 (C=O). MS (EI): m/z 327. Anal. calcd for C_17_H_17_N_3_O_2_S: C, 62.37; H, 5.23; N, 12.83. Found: C, 62.43; H, 5.28; N, 12.87.

***N*-(6-Ethoxybenzo[*d*]thiazol-2-yl)-N-(2-methylbenzyl)acetamide** **BT-35**


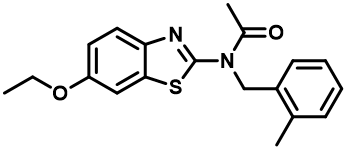
Yield 95 %, mp 185-6 °C. ^1^H NMR (200 MHz; DMSO-d_6_; δ, ppm): 1.35 (t, 3H, *J* = 6.9 Hz, CH_3_CH_2_), 2.28 (s, 3H, CH_3_CO), 2.41 (s, 3H, H_3_CC(2`)), 4.08 (q, 2H, *J* = 6.9 Hz, CH_2_O), 5.49 (brs, 2H, NCH_2_), 6.77 (d, 1H, *J* = 7.0 Hz, HC(6`)),6.98 (dd, 1H, *J* = 8.8, 2.5 Hz, HC(5)), 7.05-7.20 (m, 2H, HC(4`, 5`)), 7.25 (d, 1H, *J* = 6.9 Hz, HC(3`)), 7.53 (d, 1H, *J* = 2.5 Hz, HC(7)), 7.58 (d, 1H, *J* = 8.8 Hz, HC(4)). ^13^С NMR (50 MHz; DMSO-d_6_; δ, ppm): 14.58 (CH_3_CH_2_), 18.55 (H_3_CC(2`)), 22.49 (CH_3_CO), 48.89 (NCH_2_), 63.73 (CH_2_O), 105.24 (C(7)), 115.31 (C(5)), 121.57 (C(4)), 123.58 (C(5`)), 126.20 (C(6`)),126.80 (C(3`)), 130.28 (C(1`)), 134.17 (C(4)), 134.54 (C(7a`)), 134.86 (C(2`)), 141.71 (C(3a)), 155.61 (C(6)), 157.26 (C(2)), 171.24 (C=O). MS (EI): m/z 340. Anal. calcd for C_19_H_20_N_2_O_2_S: C, 67.03; H, 5.92; N, 8.23. Found: C, 67.10; H, 5.99; N, 8.18.


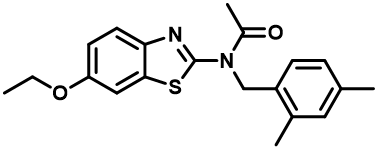
***N*-(2,4-Dimethylbenzyl)-*N*-(6-ethoxybenzo[*d*]thiazol-2-yl)acetamide BT-36**

Yield 94 %, mp 183-4 °C. ^1^H NMR (200 MHz; DMSO-d_6_; δ, ppm): 1.34 (t, 3H, *J* = 6.9 Hz, CH_3_CH_2_), 2.22 (s, 3H, H_3_CC(2`)), 2.27 (s, 3H, CH_3_CO), 2.36 (s, 3H, H_3_CC(4`)),4.06 (q, 2H, *J* = 6.9 Hz, CH_2_O), 5.44 (brs, 2H, NCH_2_), 6.61 (d, 1H, *J* = 7.8 Hz, HC(6`)), 6.90 (d, 1H, *J* = 7.9 Hz, HC(5`)),6.97 (dd, 1H, *J* = 8.8, 2.5 Hz, HC(5)), 7.06 (s, 1H, HC(3`)), 7.55 (d, 1H, *J* = 2.5 Hz, HC(7)), 7.57 (d, 1H, *J* = 8.8 Hz, HC(4)). ^13^С NMR (50 MHz; DMSO-d_6_; δ, ppm): 14.61 (CH_3_CH_2_), 18.55 (H_3_CC(2`)), 20.40 (H_3_CC(4`)), 22.55 (CH_3_CO), 48.64 (NCH_2_), 63.54 (CH_2_O), 104.96 (C(7)), 115.25 (C(5)), 121.52 (C(4)), 123.4 (C(5`)),126.74 (C(6`)), 131.01 (C(3`)), 131.40 (C(1`)), 134.05 (C(7a)), 134.62 (C(2`)), 135.85 (C(4`)), 141.56 (C(3a)), 155.48 (C(6)), 157.05 (C(2)), 171.34 (C=O). MS (EI): m/z 354. Anal. calcd for C_20_H_22_N_2_O_2_S: C, 67.77; H, 6.26; N, 7.90. Found: C, 67.83; H, 6.32; N, 7.87.

***N*-(6-Ethoxybenzo[*d*]thiazol-2-yl)-*N*-(2-methoxybenzyl)acetamide BT-37**

Yield 94 %б 163-4 °С. ^1^H NMR (200 MHz; DMSO-d_6_; δ, ppm): 1.35 (t, 3H, *J* = 6.9 Hz, CH_3_CH_2_O), 2.25 (s, 3H, CH_3_CON), 2.32 (s, 3H, CH_3_O), 4.06 (q, *J* = 6.9 Hz, 2H, CH_2_O), 5.46 (brs, 2H, CH_2_N), 6.95-7.06 (m, 2H, HC(6`, 5)), 7.14-7.26 (m, 2H, HC(3`, 5`)), 7.35 (t, 1H, *J* = 7.5 Hz, HC(4`)), 7.56 (d, 1H, *J* = 2.4 Hz, HC(7)), 7.61 (d, 1H, *J* = 8.9 Hz, HC(4)). ^13^С NMR (50 MHz; DMSO-d_6_; δ, ppm): 14.61 (CH_3_CH_2_), 20.63 (CH_3_O), 22.61 (CH_3_CO), 46.28 (CH_2_N), 63.54 (CH_2_O), 104.95 (C(7)), 115.33 (C(5)), 121.61 (C(4)), 122.91 (C(4`)), 126.06 (C(6`)), 126.43 (C(5`)), 128.34 (C(3`)), 128.76 (C(2`)), 134.07 (C(3a)), 141.47 (C(7a)), 147.90 (C(1`)),155.54 (C(6)), 157.14 (C(2)), 169.00 (C=O), 171.18 (NC=O). MS (EI): m/z 356. Anal. calcd for C_19_H_20_N_2_O_3_S: C, 64.02; H, 5.66; N, 7.86. Found: C, 64.08; H, 5.69; N, 7.82.

***N*-(6-Ethoxybenzo[*d*]thiazol-2-yl)-*N*-(2-ethoxybenzyl)acetamide BT-38**

Yield 93 %, mp 190-1 °C. ^1^H NMR (200 MHz; DMSO-d_6_; δ, ppm): 1.36 (td, 6H, *J* = 6.9, 5.4 Hz, CH_3_CH_2_, CH`_3_CH_2_), 2.30 (s, 3H, CH_3_CO), 4.10 (dq, 4H, *J* = 13.7, 7.0 Hz, CH_2_O, C’H_2_O), 5.46 (s, 2H, CH_2_N), 6.76-6.92 (m, 2H, HC(3`, 4`)), 6.97 (dd, 1H, *J* = 8.8, 2.5 Hz, HC(5)), 7.04 (d, 1H, *J* = 8.2 Hz, HC(6`)), 7.24 (dt, 1H, *J* = 8.7, 4.5 Hz, HC(5`)),7.53 (d, 1H, *J* = 2.6 Hz, HC(7)), 7.59 (d, 1H, *J* = 8.8 Hz, HC(4)). ^13^С NMR (50 MHz; DMSO-d_6_; δ, ppm): 14.61 (CH_3_, C’H_3_), 22.64 (CH_3_CO), 46.81 (NCH_2_), 63.39 (C’H_2_O), 63.55 (CH_2_O), 104.94 (C(3`)), 111.70 (C(7)), 115.22 (C(4)), 120.40 (C(5`)), 121.52 (C(6)), 124.16 (C(6`)), 125.58 (C(1’)), 128.32 (C(4`)), 134.03 (C(7a)), 141.61 (C(3a)), 155.48 (C(2`)), 155.71 (C(2)), 157.08 (C(6)), 171.34 (C=O). MS (EI): m/z 370. Anal. calcd for C_20_H_22_N_2_O_3_S: C, 64.84; H, 5.99; N, 7.56. Found: C, 64.93; H, 6.04; N, 7.59.

***N*-(6-Ethoxybenzo[*d*]thiazol-2-yl)-*N*-(2-propoxybenzyl)acetamide BT-39**

Yield 92 %, mp 177-8 °C. ^1^H NMR (200 MHz; DMSO-d_6_; δ, ppm): 1.03 (t, 3H, *J* = 7.4 Hz, H_3_C(3``)), 1.34 (t, 3H, *J* = 7.0 Hz, CH_3_CH_2_), 1.79 (h, 2H, *J* = 7.1 Hz, H_2_C(2``)), 2.29 (s, 3H, CH_3_CO), 3.86-4.23 (m, 4H, CH_2_OC(2’, 6), 5.47 (s, 2H,CH_2_N), 6.78-6.91 (m, 2H, HC(3`, 4`)), 6.97 (dd, 1H, *J* = 8.8, 2.6 Hz, HC(5)), 7.04 (d, 1H, *J* = 8.2 Hz, HC(6’)), 7.23 (dt, 1H, *J* = 8.8, 4.4 Hz, HC(5`)), 7.53 (d, 1H, *J* = 2.5 Hz, HC(7)), 7.58 (d, 1H, *J* = 8.8 Hz, HC(4)). ^13^С NMR (50 MHz; DMSO-d_6_; δ, ppm): 10.45 (C(3``)), 14.61 (CH_3_CH_2_OC(6)), 22.01 (C(2``)), 22.58 (CH_3_CO), 46.66 (CH_2_N), 63.55 (CH_2_OC(6)), 69.18 (CH_2_OC(2`)), 104.93 (C(7)), 111.65 (C(3`)), 115.21 (C(5)), 120.37 (C(4)), 121.52 (C(4`)), 124.22 (C(1`)), 125.43 (C(6`)), 128.28 (C(7a)), 134.04 (5`), 141.59 (C(3a)), 155.48 (C(6)), 155.75 (C(2`)), 157.11 (C(2)), 171.25 (C=O). MS (EI): m/z 384. Anal. calcd for C_21_H_24_N_2_O_3_S: C, 65.60; H, 6.29; N, 7.29. Found: C, 65.67; H, 6.36; N, 7.35.

***N*-(6-Ethoxybenzo[*d*]thiazol-2-yl)-*N*-(2-(prop-2-yn-1-yloxy)benzyl)acetamide BT-40**

Yield 92 %, mp 182-3 °C. ^1^H NMR (200 MHz; DMSO-d_6_; δ, ppm): 1.34 (t, 3H, *J* = 6.9 Hz, CH_3_CH_2_), 2.30 (s, 3H, CH_3_CO), 3.65 (t, 1H, *J* = 2.2 Hz, HC(3``)), 4.06 (q, 2H, *J* = 6.9 Hz, CH_2_O), 4.95 (d, 2H, *J* = 2.2 Hz, H_2_C(1``)), 5.46 (brs, 2H, NCH_2_), 6.80-6.90 (m, 2H, HC(3`, 4`)), 6.97 (dd, 1H, *J* = 8.8, 2.5 Hz, HC(5)), 7.16 (d, 1H, *J* = 8.0 Hz, HC(6`)), 7.28 (t, 1H, *J* = 7.7 Hz, HC(5`)), 7.54 (d, 1H, *J* = 2.5 Hz, HC(7)), 7.61 (d, 1H, *J* = 8.8 Hz, HC(4)). ^13^С NMR (50 MHz; DMSO-d_6_; δ, ppm): 14.61 (CH_3_CH_2_), 22.61 (CH_3_CO), 46.59 (NCH_2_), 55.85 (C(1``)), 63.54 (CH_3_CH_2_O), 78.50 (C(3``)), 79.16 (C(2``)), 104.92 (C(7)), 112.42 (C(3`)), 115.23 (C(5)), 121.36 (C(5`)), 121.58 (C(4)), 124.64 (C(1`)), 125.43 (C(6`)), 128.16 (C(4`)), 134.03 (C(7a)), 141.56 (C(3a)), 154.36 (C(6)), 155.48 (C(2`)), 157.03 (C(2)), 171.31 (C=O). MS (EI): m/z 380. Anal. calcd for C_21_H_20_N_2_O_3_S: C, 66.30; H, 5.30; N, 7.36. Found: C, 66.37; H, 5.35; N, 7.39.

**
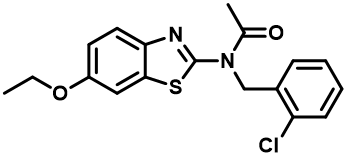
*N*-(2-Chlorobenzyl)-*N*-(6-ethoxybenzo[*d*]thiazol-2-yl)acetamide BT-41**

Yield 93 %, mp 177-8 °C. ^1^H NMR (200 MHz; DMSO-d_6_; δ, ppm): 1.34 (t, 3H, *J* = 6.9 Hz, CH_3_CH_2_), 2.30 (s, 3H, CH_3_CO), 4.06 (q, 2H, *J* = 6.9 Hz, CH_2_O), 5.55 (s, 2H, CH_2_N), 6.89-7.03 (m, 2H, HC(5, 6`)), 7.21-7.39 (m, 2H, HC(4`, 5`)), 7.49-7.64 (m, 3H, HC(4, 7, 3`)). ^13^С NMR (50 MHz; DMSO-d_6_; δ, ppm): 14.61 (CH_3_CH_2_), 22.58 (CH_3_CO), 48.96 (CH_2_N), 63.57 (CH_2_O), 104.99 (C(7)), 115.30 (C(5)), 121.64 (C(4)), 126.01 (C(5`)), 127.76 (C(3`)), 128.89 (C(4`)), 129.61 (C(6`)), 131.37 (C(1`)), 133.71 (C(2`)), 134.08 (C(7a)), 141.50 (C(3a)), 155.57 (C(6)), 156.90 (C(2)), 171.16 (C=O). MS (EI): m/z 360. Anal. calcd for C_18_H_17_ClN_2_O_2_S: C, 59.91; H, 4.75; N, 7.76. Found: C, 60.01; H, 4.83; N, 7.73.

**
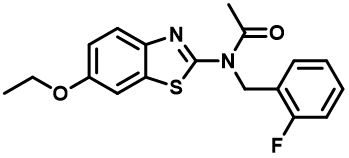
*N*-(6-Ethoxybenzo[*d*]thiazol-2-yl)-*N*-(2-fluorobenzyl)acetamide BT-42**

Yield 89 %, mp 156-7 °C. ^1^H NMR (200 MHz; DMSO-d_6_; δ, ppm): 1.34 (t, 3H, *J* = 7.0 Hz, CH_3_CH_2_), 2.36 (s, 3H, CH_3_CO), 4.06 (q, 2H, *J* = 6.9 Hz, CH_2_O), 5.57 (brs, 2H, CH_2_N), 6.84-7.47 (m, 4H, HC(5, 3`, 4`, 5`, 6`)), 7.54 (d, 1H, *J* = 2.5 Hz, HC(7)), 7.60 (d, 1H, *J* = 8.9 Hz, HC(4)). ^13^С NMR (50 MHz; DMSO-d_6_; δ, ppm): 14.60 (CH_3_CH_2_), 22.64 (СH_3_CO), 45.34 (d, *J* = 5 Hz, CH_2_N), 63.56 (CH_2_O), 104.97 (C(7)), 115.30 (C(5)), 115.67 (d, *J* = 20.5 Hz, C(3`)), 121.62 (C(4)), 123.62 (d, *J* = 14 Hz, C(1`)), 124.80 (d, *J* = 3.5 Hz, C(5`)),127.15 (d, *J* = 4 Hz, C(4`)), 129.20 (d, *J* = 8.5 Hz, C(6`)), 134.04 (C(7a)), 141.50 (С(3a)), 155.55 (С(6)), 156.96 (С(2), 160.69 (d, *J* = 242.5 Hz, C(2`)), 171.14 (C=O). MS (EI): m/z 344. Anal. calcd for C_18_H_17_FN_2_O_2_S: C, 62.77; H, 4.98; N, 8.13. Found: C, 62.86; H, 5.04; N, 8.19.

**2-((*N*-(6-Ethoxybenzo[*d*]thiazol-2-yl)acetamido)methyl)phenyl acetate BT-43**

Yield 90 %, mp 179-181 °C. ^1^H NMR (200 MHz; DMSO-d_6_; δ, ppm): 1.34 (t, 3H, *J* = 6.9 Hz, CH_3_CH_2_), 2.25 (s, 3H, H_3_CCON), 2.32 (s, 3H, H_3_CCOO), 4.06 (q, 2H, *J* = 7.0 Hz, CH_2_O), 5.46 (brs, 2H, CH_2_N), 6.87-7.09 (m, 2H, HC(5, 6`)), 7.15-7.27 (m, 2H, HC(3`, 5`)), 7.34 (td, 1H, *J* = 7.4, 7.0, 1.7 Hz, HC(4`)), 7.55 (d, 1H, *J* = 2.5 Hz, HC(7)), 7.61 (d, 1H, *J* = 8.8 Hz, HC(4)). ^13^С NMR (50 MHz; DMSO-d_6_; δ, ppm): 14.61 (CH_3_CH_2_), 20.61 (CH_3_COO), 22.61 (CH_3_CON), 46.29 (CH_2_N), 63.57 (CH_2_O), 104.99 (C(7)), 115.33 (C(5)), 121.61 (C(4)), 122.89 (C(6’)), 126.09 (C(4’)), 126.40 (C(2’)), 128.34 (C(5’)), 128.77 (C(3’)), 134.09 (C(7a)), 141.50 (C(3a)), 147.91 (C(1’)), 155.57 (C(6)), 157.17 (C(2)), 168.97 (CH_3_COO), 171.15 (CH_3_CON). MS (EI): m/z 384. Anal. calcd for C_20_H_20_N_2_O_4_S: C, 62.48; H, 5.24; N, 7.29. Found: C, 62.51; H, 5.26; N, 7.32.

***N*-(6-Ethoxybenzo[*d*]thiazol-2-yl)-*N*-(2-(trifluoromethoxy)benzyl)acetamide BT-44**

Yield 90 %, mp 174-5 °C. ^1^H NMR (200 MHz; DMSO-d_6_; δ, ppm): 1.34 (t, 3H, *J* = 6.9 Hz, CH_3_CH_2_), 2.31 (s, 3H, CH_3_CO), 4.06 (q, 2H, *J* = 6.9 Hz, CH_2_O), 5.58 (brs, 2H, CH_2_N), 6.98 (dd, 1H, *J* = 8.8, 2.7 Hz, HC(5)), 7.08 (d, 1H, *J* = 7.4 Hz, HC(6`)), 7.27-7.49 (m, 3H, HC(3`, 4`, 5`), 7.54 (s, 1H, HC(7)), 7.57 (d, 1H, *J* = 8.8 Hz, HC(4)). ^13^С NMR (50 MHz; DMSO-d_6_; δ, ppm): 14.61 (CH_3_CH_2_), 22.58 (CH_3_CO), 46.11 (CH_2_N), 63.57 (CH_2_O), 105.00 (C(7)), 115.32 (C(5)), 120.08 (q, *J* = 260 Hz, CF_3_), 120.82 (C(2`)), 121.61 (C(4)), 126.92 (C(3`)), 127.95 (C(4`)), 129.13 (C(6`)), 134.07 (C(7a)), 141.47 (C(3a)), 145.90 (C(1`)), 155.57 (C(6)), 156.93 (C(2)), 171.09 (C=O). MS (EI): m/z 410. Anal. calcd for C_19_H_17_F_3_N_2_O_3_S: C, 55.60; H, 4.18; N, 6.83. Found: C, 55.66; H, 4.24; N, 6.90.

***N*-(2-(Benzyloxy)benzyl)-*N*-(6-ethoxybenzo[*d*]thiazol-2-yl)acetamide BT-45**

Yield 95 %, mp 194-5°C. ^1^H NMR (200 MHz; DMSO-d_6_; δ, ppm): 1.34 (t, 3H, *J* = 6.9 Hz, CH_3_CH_2_), 2.28 (s, 3H, CH_3_CO), 4.07 (q, 2H, *J* = 6.9 Hz, CH_2_O), 5.25 (s, 2H, NCH_2_), 5.52 (brs, 2H, PhCH_2_O), 6.82-6.93 (m, 3H, HC(3`, 2``,6``)), 7.08-7.73 (m, 9H, Ph, HC(4, 5, 7, 3`, 5`, 6`)).^13^С NMR (50 MHz; DMSO-d_6_; δ, ppm): 14.60 (CH_3_CH_2_), 22.52 (CH_3_CO), 46.77 (NCH_2_), 63.66 (CH_3_CH_2_O), 69.49 (CH_2_OPh), 105.11 (C(7)), 112.37 (C(3`)), 115.26 (C(5)), 120.82 (C(5`)), 121.54 (C(4)), 124.67 (C(1`)), 125.53 (C(6`)), 127.44 (C(2``, 6``)), 127.79 (C(4``)), 128.24 (C(4`)), 128.42 (C(3``, 5``)), 134.07 (C(7a)), 136.97 (C(1``)), 141.65 (C(3a)), 155.42 (C(6)), 155.54 (C(2`)),157.16 (C(2)), 171.28 (C=O). MS (EI): m/z 432. Anal. calcd for C_25_H_24_N_2_O_3_S: C, 69.42; H, 5.59; N, 6.48. Found: C, 69.49; H, 5.53; N, 6.52.

***N*-(2,5-Dimethoxybenzyl)-*N*-(6-ethoxybenzo[*d*]thiazol-2-yl)acetamide BT-46**

Yield 94 %, mp 173-4 °C. ^1^H NMR (200 MHz; DMSO-d_6_; δ, ppm): 1.34 (t, 3H, *J* = 6.9 Hz, CH_3_CH_2_), 2.31 (s, 3H, CH_3_CO), 3.58 (s, 3H, CH_3_O), 3.83 (s, 3H, CH_3_O), 4.07 (q, 2H, *J* = 6.9 Hz, CH_2_O), 5.42 (brs, 2H, CH_2_N), 6.38 (brs, 1H, HC(6`)), 6.82 (dd, 1H, *J* = 8.9, 2.3 Hz, HC(5)), 6.93-7.05 (m, 2H, HC(3`, 4`),7.52 (d, 1H, *J* = 2.3 Hz, HC(7)), 7.60 (d, 1H, *J* = 8.8 Hz, HC(4)). ^13^С NMR (50 MHz; DMSO-d_6_; δ, ppm): 14.58 (CH_3_CH_2_), 22.47 (CH_3_CO), 46.67 (CH_2_N), 55.27 (CH_3_O), 55.97 (CH_3_O), 63.71 (CH_2_O), 105.21 (C(7)), 111.84 (C(3`)), 112.05 (C(4`)), 112.83 (C(6`)), 115.32 (C(5)), 121.57 (C(4)), 125.62 (C(1`)), 134.05 (C(7a)), 141.71 (C(3a)), 150.63 (C(5`)),153.32 (C(2`)), 155.60 (C(6)), 157.20 (C(2)), 171.22 (C=O). MS (EI): m/z 386. Anal. calcd for C_20_H_22_N_2_O_4_S: C, 62.16; H, 5.74; N, 7.25. Found: C, 62.21; H, 5.78; N, 7.21.

***N*-(2,4-Dimethoxybenzyl)-*N*-(6-ethoxybenzo[*d*]thiazol-2-yl)acetamide BT-47**

Yield 88 %, mp 147-8 °C. ^1^H NMR (200 MHz; DMSO-d_6_; δ, ppm): 1.34 (t, 3H, *J* = 6.9 Hz, CH_3_CH_2_). 2.30 (s, 3H, CH_3_CO), 3.71 (s, 3H, CH_3_OC(4`)), 3.86 (s, 3H, CH_3_OC(2`)), 4.06 (q, 2H, *J* = 6.9 Hz, CH_2_O), 5.36 (s, 2H, CH_2_N), 6.43 (dd, 1H, *J* = 8.5, 2.3 Hz, HC(5`)), 6.63 (d, 1H, *J* = 2.4 Hz, HC(3`)), 6.74 (d, 1H, *J* = 8.5 Hz, HC(6`)), 6.98 (dd, 1H, *J* = 8.8, 2.5 Hz, HC(5)), 7.53 (d, 1H, *J* = 2.5 Hz, HC(7)), 7.60 (d, 1H, *J* = 8.8 Hz, HC(4)). ^13^С NMR (50 MHz; DMSO-d_6_; δ, ppm): 14.61 (CH_3_CH_2_), 22.49 (CH_3_CO), 46.32 (CH_2_N), 55.14 (CH_3_OC(4`)), 55.48 (CH_3_OC(2`)), 63.55 (CH_2_O), 98.54 (C(3`)), 104.72 (C(5`)), 104.93 (C(7)), 115.21 (C(5)), 116.15 (C(6`)), 121.54 (C(4)), 126.22 (C(1`)), 134.01 (C(7a)), 141.62 (C(3a)), 155.48 (C(2)), 157.09 (C(6)), 157.30 (C(4`)), 159.79 (C(2`)), 171.34 (C=O). MS (EI): m/z 386. Anal. calcd for C_20_H_22_N_2_O_4_S: C, 62.16; H, 5.74; N, 7.25. Found: C, 62.24; H, 5.83; N, 7.29.

***N*-(6-Ethoxybenzo[*d*]thiazol-2-yl)-*N*-(3-fluoro-2-methoxybenzyl)acetamide BT-48**

Yield 91 %, mp 143-4 °C. ^1^H NMR (200 MHz; DMSO-d_6_; δ, ppm): 1.35 (t, 3H, *J* = 6.9 Hz, CH_3_CH_2_), 2.35 (s, 3H, CH_3_CO), 3.99 (s, 3H, CH_3_O), 4.06 (q, 2H, *J* = 7.0 Hz, CH_2_O), 5.53 (brs, 2H, CH_2_N), 6.74 (d, 1H, *J* = 7.7 Hz, HC(5)), 6.98 (dd, 1H, *J* = 8.6, 2.8 Hz, HC(5`)), 7.02-7.09 (m, 1H, HC(4`)), 7.10-7.31 (m, 1H, HC(6’)), 7.55 (d, 1H, *J* = 2.6 Hz, HC(7)), 7.60 (d, 1H, *J* = 8.8 Hz, HC(4)). ^13^С NMR (50 MHz; DMSO-d_6_; δ, ppm): 14.61 (CH_3_CH_2_), 22.70 (CH_3_CO), 46.32 (CH_2_N), 61.14 (d, *J* = 5 Hz, CH_3_O), 63.56 (CH_2_O), 104.96 (C(7)), 115.28 (C(5)), 115.87 (d, *J* = 19.5 Hz, C(4`)), 121.50 (d, *J* = 4 Hz, C(6`)), 121.58 (C(4)), 124.14 (d, *J* = 14 Hz, C(5`)), 131.38 (C(1`)), 134.01 (C(7a)), 141.53 (C(3a)), 144.50 (C(2`)), 154.80 (d, *J* = 244 Hz, C(3`)), 155.54 (C(6)), 156.96 (C(2)), 171.22 (C=O). MS (EI): m/z 374. Anal. calcd for C_19_H_19_FN_2_O_3_S: C, 60.95; H, 5.11; N, 7.48. Found: C, 61.03; H, 5.20; N, 7.55.

***N*-(6-Ethoxybenzo[*d*]thiazol-2-yl)-*N*-(5-fluoro-2-methoxybenzyl)acetamide BT-49**

Yield 91 %, mp 163-4 °C. ^1^H NMR (200 MHz; DMSO-d_6_; δ, ppm): 1.34 (t, 3H, *J* = 7.0 Hz, CH_3_CH_2_), 2.31 (s, 3H, CH_3_CO), 3.88 (s, 3H, CH_3_O), 4.06 (q, 2H, *J* = 6.9 Hz, CH_2_O), 5.42 (brs, 2H, CH_2_N), 6.62 (dd, 1H, *J* = 8.7, 2.0 Hz, HC(3`)), 6.98 (dd, 1H, *J* = 8.8, 2.5 Hz, HC(5)), 7.07 (d, 1H, *J* = 1.7 Hz, HC(6`)), 7.10 (d, 1H, *J* = 1.9 Hz, HC(4`)), 7.54 (d, 1H, *J* = 2.5 Hz, HC(7)), 7.60 (d, 1H, *J* = 8.9 Hz, HC(4)). ^13^С NMR (50 MHz; DMSO-d_6_; δ, ppm): 14.62 (CH_3_CH_2_), 22.58 (CH_3_CO), 46.64 (CH_2_N), 55.99 (CH_3_O), 63.54 (CH_2_O), 104.94 (C(7)), 112.13 (d, *J* = 6.5 Hz, C(3`)), 112.43 (d, *J* = 24 Hz, C(4`)), 114.10 (d, *J* = 22.7 Hz, C(6`)), 115.27 (C(5)), 121.58 (C(4)), 126.24 (d, *J* = 6.20 Hz, C(1`)), 134.01 (C(7a)), 141.50 (C(3a)), 152.64 (C(6)), 154.31 (d, *J* = 234.5 Hz, C(5`)), 155.51 (C(2`)), 156.93 (C(2)), 171.25 (C=O). MS (EI): m/z 374. Anal. calcd for C_19_H_19_FN_2_O_3_S: C, 60.95; H, 5.11; N, 7.48. Found: C, 61.02; H, 5.20; N, 7.56.

***N*-(6-Ethoxybenzo[*d*]thiazol-2-yl)-*N*-(3,4,5-trimethoxybenzyl)acetamide BT-50**

Yield 74 %, mp 176-7 °C. ^1^H NMR (200 MHz; DMSO-d_6_; δ, ppm): 1.35 (t, 3H, *J* = 6.9 Hz, CH_3_CH_2_), 2.37 (s, 3H, CH_3_CON), 3.64 (s, 3H, CH_3_O-C(4`)), 3.69 (s, 6H, CH_3_O-(C(3`, 5`)), 4.08 (q, 2H, *J* = 6.9 Hz, CH_2_O), 5.48 (brs, 2H, CH_2_N), 6.56 (brs, 2H, HC(2`, 6`)), 7.01 (dd, 1H, *J* = 8.8, 2.5 Hz, HC(5)), 7.54 (d, 1H, *J* = 2.1 Hz, HC(7)), 7.65 (d, 1H, *J* = 8.8 Hz, HC(4)). ^13^С NMR (50 MHz; DMSO-d_6_; δ, ppm): 14.63 (CH_3_CH_2_), 22.96 (CH_3_CON), 50.66 (CH_2_N), 55.81 (CH_3_O-C(4`)), 59.93 (CH_3_O-(C(3`, 5`)), 63.57 (CH_2_O), 103.50 (C(2`, 6`)), 104.99 (C(7)), 115.31 (C(5)), 121.55 (C(4)), 132.49 (C(1`)), 134.07 (C(7a)), 136.65 (C(4`)), 141.53 (C(3a)), 153.08 (C(3`, 5`)), 155.55 (C(6)), 157.39 (C(2)), 171.35 (NC=O). MS (EI): m/z 416. Anal. calcd for C_21_H_24_N_2_O_5_S: C, 60.56; H, 5.81; N, 6.73. Found: C, 60.59; H, 5.88; N, 6.69.

***N*-(6-Ethoxybenzo[*d*]thiazol-2-yl)-*N*-(2,4,6-trimethoxybenzyl)acetamide BT-51**

Yield 93 %, mp 191-2 °C. ^1^H NMR (200 MHz; DMSO-d_6_; δ, ppm): 1.34 (t, 3H, *J* = 6.9 Hz, CH_3_CH_2_), 2.34 (s, 3H, CH_3_CO), 3.68 (s, 6H, CH_3_OC(2’, 6’)), 3.73 (s, 3H, CH_3_OC(4`)), 4.06 (q, 2H, *J* = 7.0 Hz, CH_2_O), 5.38 (s, 2H, CH_2_N), 6.20 (s, 2H, HC(3`, 5`)), 6.96 (d, 1H, *J* = 9.0 Hz, HC(5)), 7.45 (s, 1H, H(7)), 7.56 (d, 1H, *J* = 9.0 Hz, HC(4)). ^13^С NMR (50 MHz; DMSO-d_6_; δ, ppm): 14.61 (CH_3_CH_2_), 22.58 (CH_3_CO), 42.41 (CH_2_N), 55.15 (CH_3_OC(4`)), 55.87 (CH_3_OC(2`, 6`)), 63.66 (CH_2_O), 91.38 (C(3`, 5`)), 104.75 (C(1`)), 105.00 (C(7)), 114.96 (C(5)), 121.39 (C(4)), 134.10 (C(7a)), 142.02 (C(3a)), 155.36 (C(6)), 157.51 (C(2)), 159.12 (C(2`, 6`)), 160.48 (C(4`)), 171.25 (C=O). MS (EI): m/z 416. Anal. calcd for C_21_H_24_N_2_O_5_S: C, 60.56; H, 5.81; N, 6.73. Found: C, 60.61; H, 5.89; N, 6.81.

***N*-(6-Ethoxybenzo[*d*]thiazol-2-yl)-*N*-(2,4,5-trimethoxybenzyl)acetamide BT-52**

Yield 94 %, mp 162-3 °C. ^1^H NMR (200 MHz; DMSO-d_6_; δ, ppm): 1.34 (t, 3H, *J* = 7.0 Hz, CH_3_CH_2_). 2.36 (s, 3H, CH_3_CO), 3.51 (s, 3H, CH_3_O), 3.78 (s, 3H, CH_3_O), 3.84 (s, 3H, CH_3_O), 4.06 (q, 2H, *J* = 7.0 Hz, CH_2_O), 5.36 (brs, 2H, CH_2_N), 6.59 (s, 1H, HC(3`)), 6.74 (s, 1H, HC(6`)), 6.99 (dd, 1H, *J* = 8.8, 2.6 Hz, HC(5)), 7.52 (d, 1H, *J* = 2.5 Hz, HC(7)), 7.63 (d, 1H, *J* = 8.8 Hz, HC(4)). ^13^С NMR (50 MHz; DMSO-d_6_; δ, ppm): 14.63 (CH_3_CH_2_), 22.58 (CH_3_CO), 46.42 (CH_2_N), 55.84 (CH_3_O), 56.12 (CH_3_O), 56.51 (CH_3_O), 63.54 (CH_2_O), 98.35 (C(3`)), 104.93 (C(7)), 112.51 (C(6`)), 115.22 (C(5)), 121.49 (C(4)), 133.95 (C(7a)), 141.59 (C(3a)), 142.44 (C(2`)), 149.11 (C(5`)), 151.05 (C(4`)), 155.48 (C(6)), 157.15 (C(2)), 171.37 (C=O). MS (EI): m/z 416. Anal. calcd for C_21_H_24_N_2_O_5_S: C, 60.56; H, 5.81; N, 6.73. Found: C, 60.63; H, 5.93; N, 6.81.

***N*-(6-Ethoxybenzo[*d*]thiazol-2-yl)-*N*-(2,3,4-trimethoxybenzyl)acetamide BT-53**

Yield 90 %, mp 141-2 °C. ^1^H NMR (200 MHz; DMSO-d_6_; δ, ppm): 1.34 (t, 3H, *J* = 7.0 Hz, CH_3_CH_2_), 2.34 (s, 3H, CH_3_CO), 3.73 (s, 3H, CH_3_O), 3.78 (s, 3H, CH_3_O), 3.90 (s, 3H, CH_3_O), 4.06 (q, 2H, *J* = 6.9 Hz, CH_2_O), 5.42 (brs, 2H, CH_2_N), 6.57 (d, 1H, *J* = 8.6 Hz, HC(5`)), 6.71 (d, 1H, *J* = 8.7 Hz, HC(6`)), 6.98 (dd, 1H, *J* = 8.8, 2.6 Hz, HC(5)), 7.53 (d, 1H, *J* = 2.5 Hz, HC(7)), 7.59 (d, 1H, *J* = 8.8 Hz, HC(4)). ^13^С NMR (50 MHz; DMSO-d_6_; δ, ppm): 14.61 (CH_3_CH_2_), 22.67 (CH_3_CO), 46.30 (CH_2_N), 55.72 (CH_3_O), 60.33 (CH_3_O), 60.63 (CH_3_O), 63.55 (CH_2_O), 104.93 (C(7)), 107.93 (C(5`)), 115.24 (C(5)), 120.15 (C(6`)), 121.52 (C(4)), 121.88 (C(1`)), 134.01 (C(7a)), 141.59 (C(3a, 3`)), 150.53 (C(2`)), 152.66 (C(4`)), 155.48 (C(6)), 157.03 (C(2)), 171.28 (C=O). MS (EI): m/z 416. Anal. calcd for C_21_H_24_N_2_O_5_S: C, 60.56; H, 5.81; N, 6.73. Found: C, 60.65; H, 5.90; N, 6.67.

**General procedure for the synthesis of *N*-benzyl-*N*-(6-*R-O*-1,3-benzothiazol-2-yl)acetamide BT-30, BT-31**

1-Iodopropane (98 mg, 0.579 mmol) or 3-bromoprop-1-yne (70 mg, 0.579 mmol) is added to a mixture of *N*-benzyl-*N*-(6-hydroxy-1,3-benzothiazol-2-yl)acetamide **BT-25** (150 mg, 0.503 mmol) and caesium carbonate (189 mg, 0.579 mmol) in anhydrous dimethylformamide (2 mL), and the reaction mixture is stirred at room temperature for 2 h. The mixture is then slowly diluted with water (3 mL), and the precipitate is filtered, washed with ethanol and water and crystallized from ethanol to afford the title compounds.

***N*-Benzyl-*N*-(6-propoxybenzo[*d*]thiazol-2-yl)acetamide BT-30**

 Yield 139 mg (81 %), mp 133-4 °C. ^1^H NMR (200 MHz; DMSO-d_6_; δ, ppm): 0.99 (t, 3H, *J* = 7.4 Hz, CH_3_CH_2_), 1.75 (h, 2H, *J* = 6.9 Hz, CH_3_CH_2_), 2.32 (s, 3H, CH_3_CO), 3.97 (t, 2H, *J* = 6.5 Hz, CH_2_O), 5.57 (brs, 2H, CH_2_N), 7.00 (dd, 1H, *J* = 8.9, 1.7 Hz, HC(5)), 7.12-7.48 (m, 5H, Ph), 7.48-7.85 (m, 2H, HC(4, 7)). ^13^С NMR (50 MHz; DMSO-d_6_; δ, ppm): 10.38 (CH_3_CH_2_), 22.00 (CH_3_CH_2_), 22.90 (CH_3_CO), 50.50 (CH_2_N), 69.48 (CH_2_O), 105.00 (C(7)), 115.31 (C(5)), 121.55 (C(4)), 125.89 (C(3`, 5`)), 127.19 (C(4`)), 128.74 (C(2`, 6`)), 134.07 (C(7a)), 136.80 (C(1`)), 141.52 (C(3a)), 155.67 (C(6)), 157.33 (C(2)), 171.28 (C=O). MS (EI): m/z 340. Anal. calcd for C_19_H_20_N_2_O_2_S: C, 67.03; H, 5.92; N, 8.23. Found: C, 67.08; H, 5.98; N, 8.18.

***N*-Benzyl-*N*-(6-(prop-2-yn-1-yloxy)benzo[*d*]thiazol-2-yl)acetamide BT-31**

Yield 142 mg (84 %), mp 140-1 °C. ^1^H NMR (200 MHz; DMSO-d_6_; δ, ppm): 2.32 (s, 3H, CH_3_CO), 3.58 (t, 1H, *J* = 2.2 Hz, HC(3``)), 4.85 (d, 2H, *J* = 2.2 Hz, H_2_C(1``)), 5.57 (brs, 2H, NCH_2_), 7.06 (dd, 1H, *J* = 8.8, 2.5 Hz, HC(5)), 7.12-7.51 (m, 5H, Ph), 7.51-7.88 (m, 2H, HC(4, 7)). ^13^С NMR (50 MHz; DMSO-d_6_; δ, ppm): 22.90 (CH_3_CO), 50.53 (NCH_2_), 55.98 (C(1``)), 78.28 (C(3``)), 79.16 (C(2``)), 106.00 (C(7)), 115.45 (C(5)), 121.58 (C(4)), 125.88 (C(3`, 5`)), 127.19 (C(4`)), 128.75 (C(2`, 6`)), 133.89 (C(7a)), 136.74 (C(1`)), 142.14 (C(3a)), 154.08 (C(6)), 157.73 (C(2)), 171.37 (C=O). MS (EI): m/z 336. Anal. calcd for C_19_H_16_N_2_O_2_S: C, 67.84; H, 4.79; N, 8.33. Found: C, 67.89; H, 4.72; N, 8.36.

# **Representative HPLC Traces**

***N*-Benzyl-*N*-(6-ethoxybenzo[*d*]thiazol-2-yl)acetamide BT-08**

| **#** | **RT, min** | **Area** | **S/N** | **Area total, %** |
| --- | --- | --- | --- | --- |
| **1** | 7.6 | 39.515 | 152.4 | 0.83 |
| **2** | 10.5 | 4696.466 | 17939.5 | 99.17 |

***N*-(6-Ethoxybenzo[*d*]thiazol-2-yl)-*N*-(2-methoxybenzyl)acetamide BT-37**

| **#** | **RT, min** | **Area** | **S/N** | **Area total, %** |
| --- | --- | --- | --- | --- |
| **1** | 10.6 | 1417.6 | 7020.7 | 100 |

***N*-(2,5-Dimethoxybenzyl)-*N*-(6-ethoxybenzo[*d*]thiazol-2-yl)acetamide BT-46**

| **#** | **RT, min** | **Area** | **S/N** | **Area total, %** |
| --- | --- | --- | --- | --- |
| **1** | 10.5 | 1027.4 | 5175.7 | 100 |

# **^1^H and ^13^C Spectra for the Target Compounds**

^1^H NMR spectrum (200 MHz, DMSO-d_6_) of compound BT-01

**
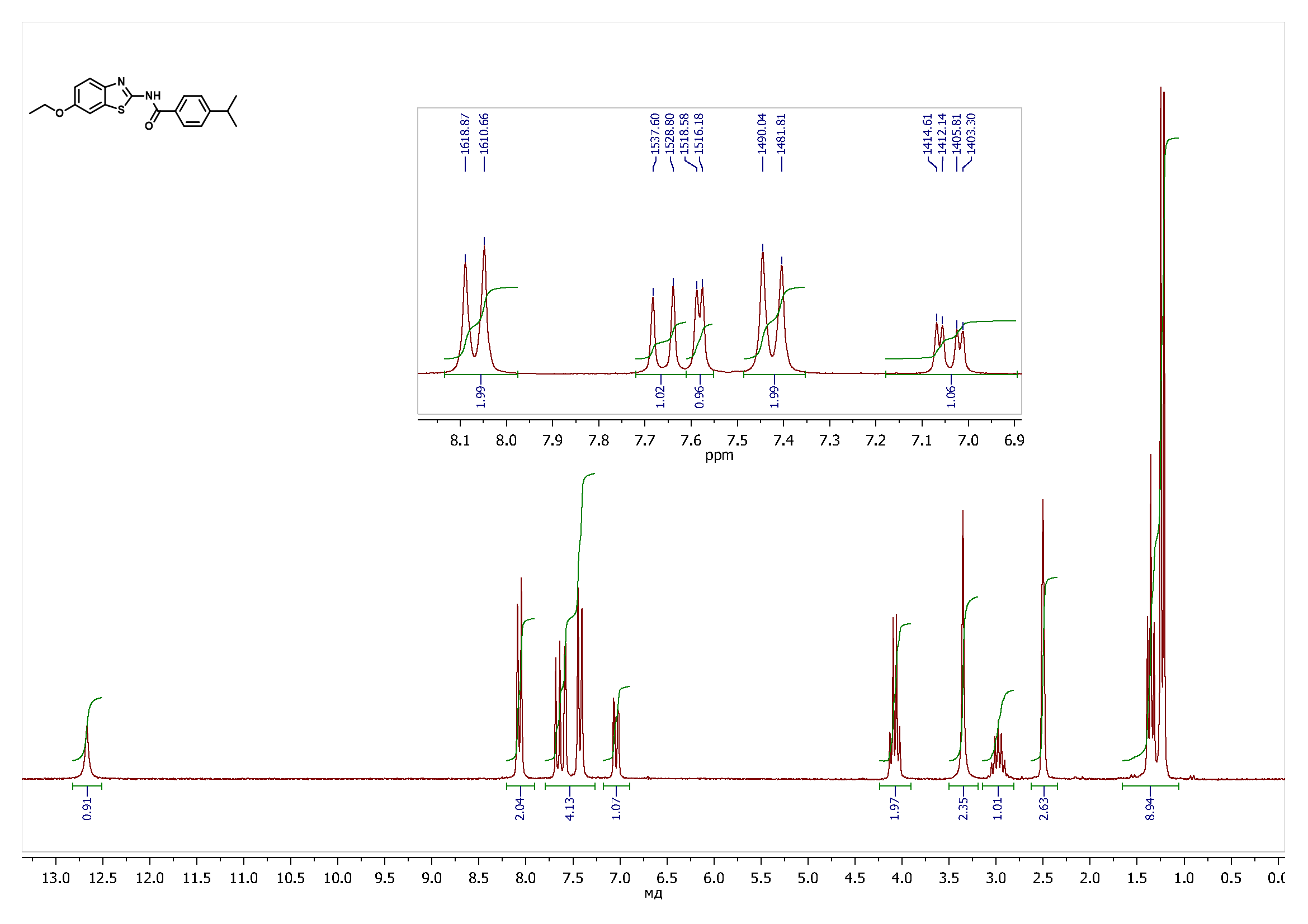
**

^13^C NMR spectrum (50 MHz, DMSO-d_6_) of compound BT-01


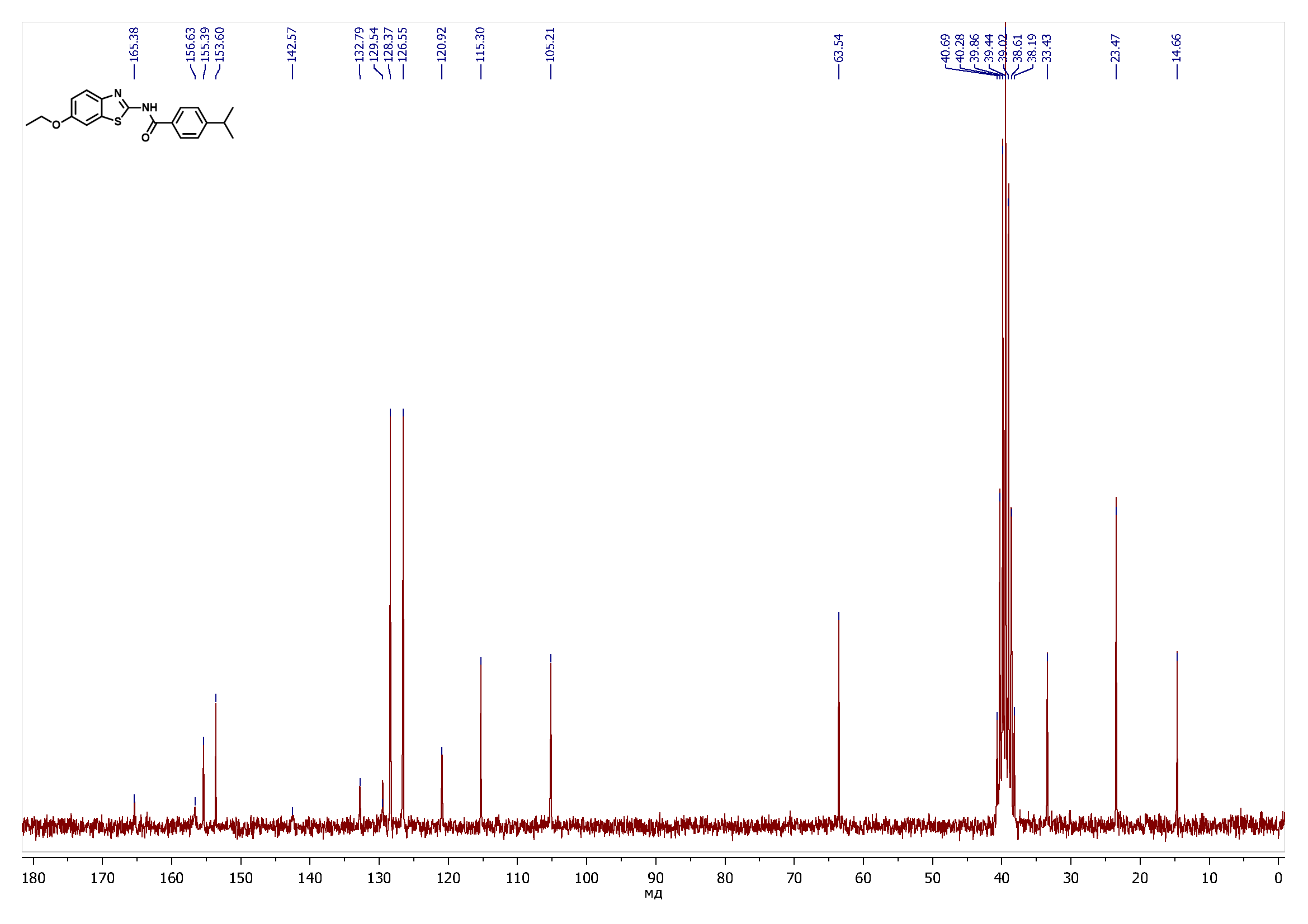


^1^H NMR spectrum (200 MHz, DMSO-d_6_) of compound BT-02

**
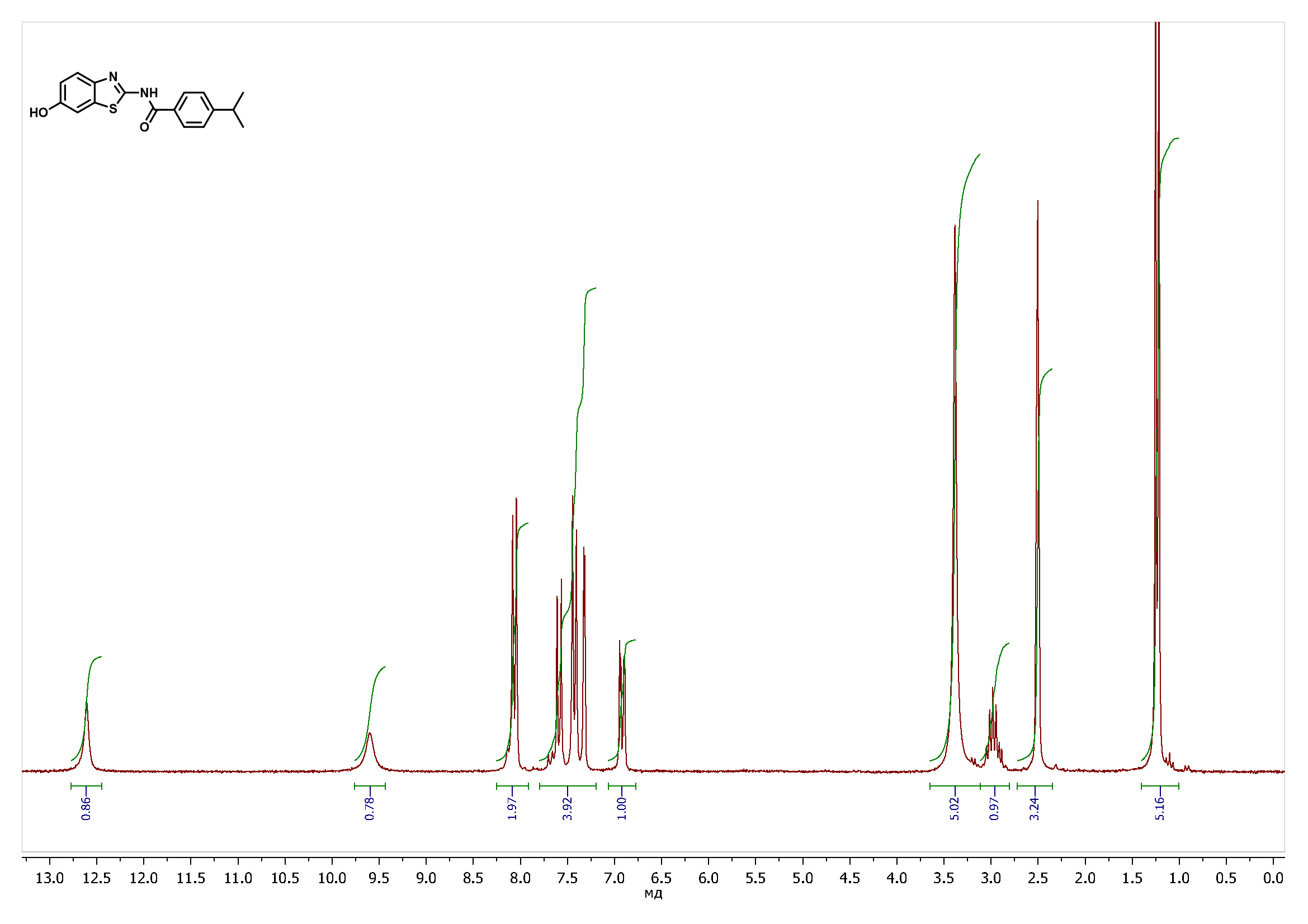
**

^13^C NMR spectrum (50 MHz, DMSO-d_6_) of compound BT-02


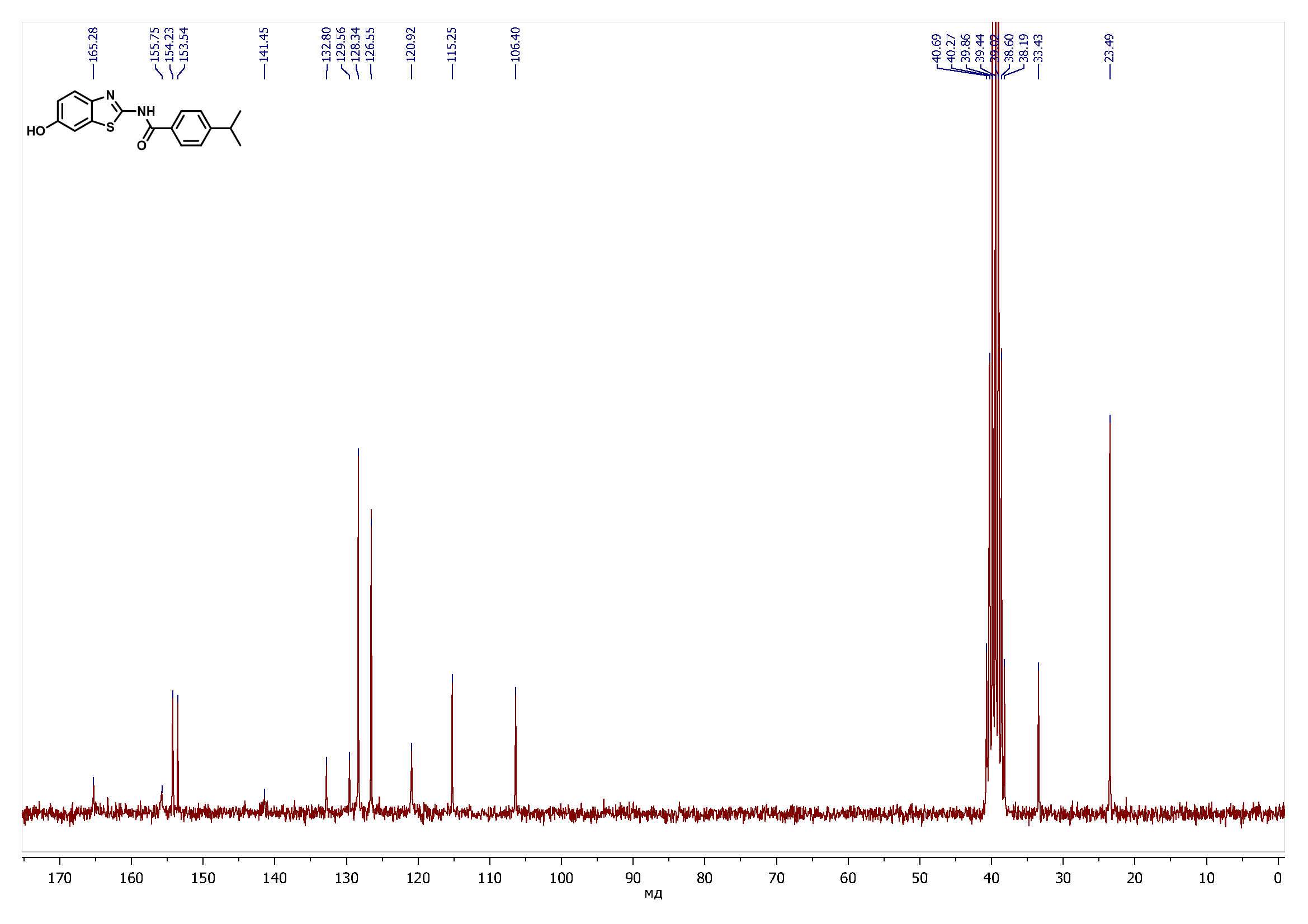


^1^H NMR spectrum (200 MHz, DMSO-d_6_) of compound BT-03

**
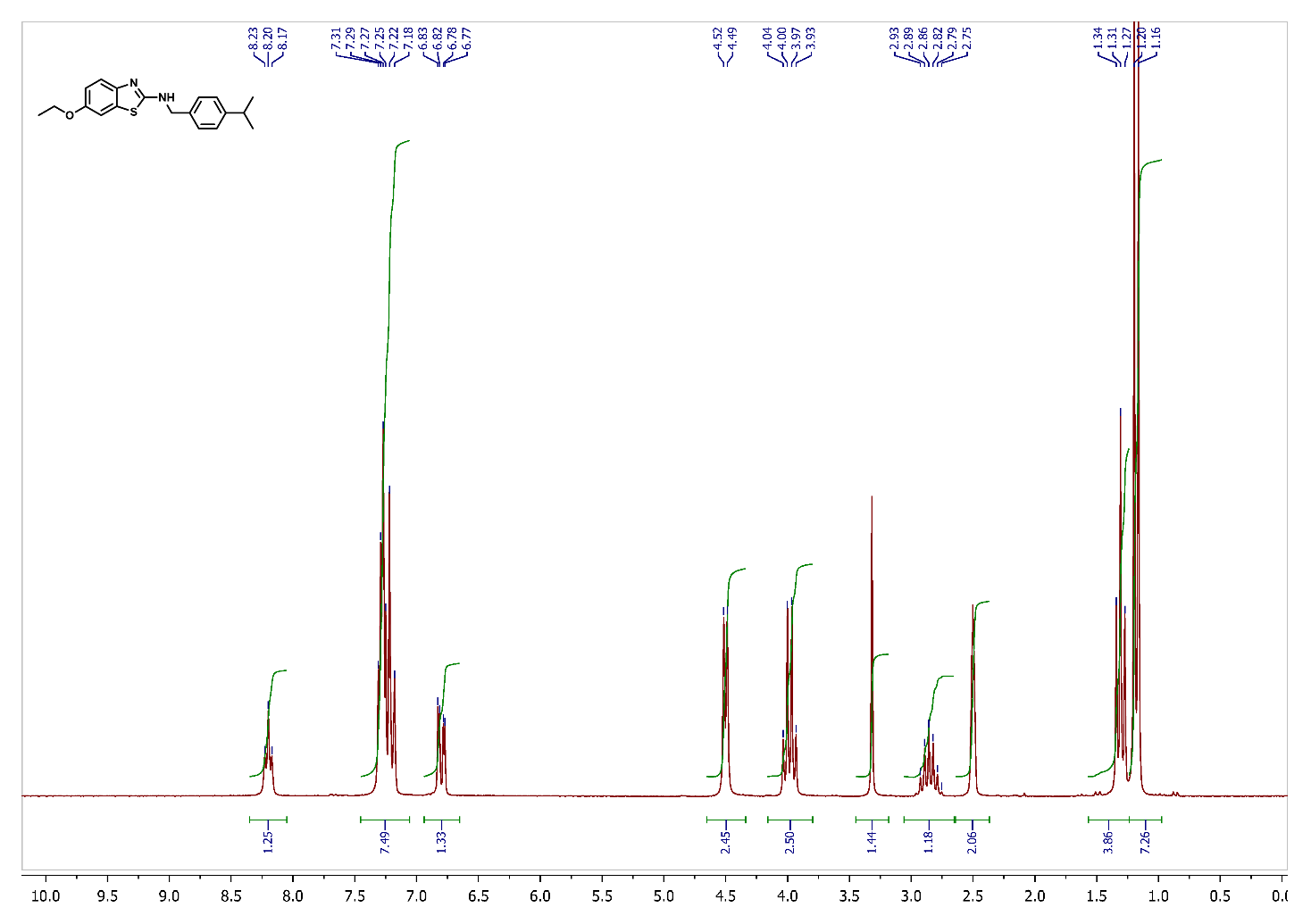
**

^13^C NMR spectrum (50 MHz, DMSO-d_6_) of compound BT-03


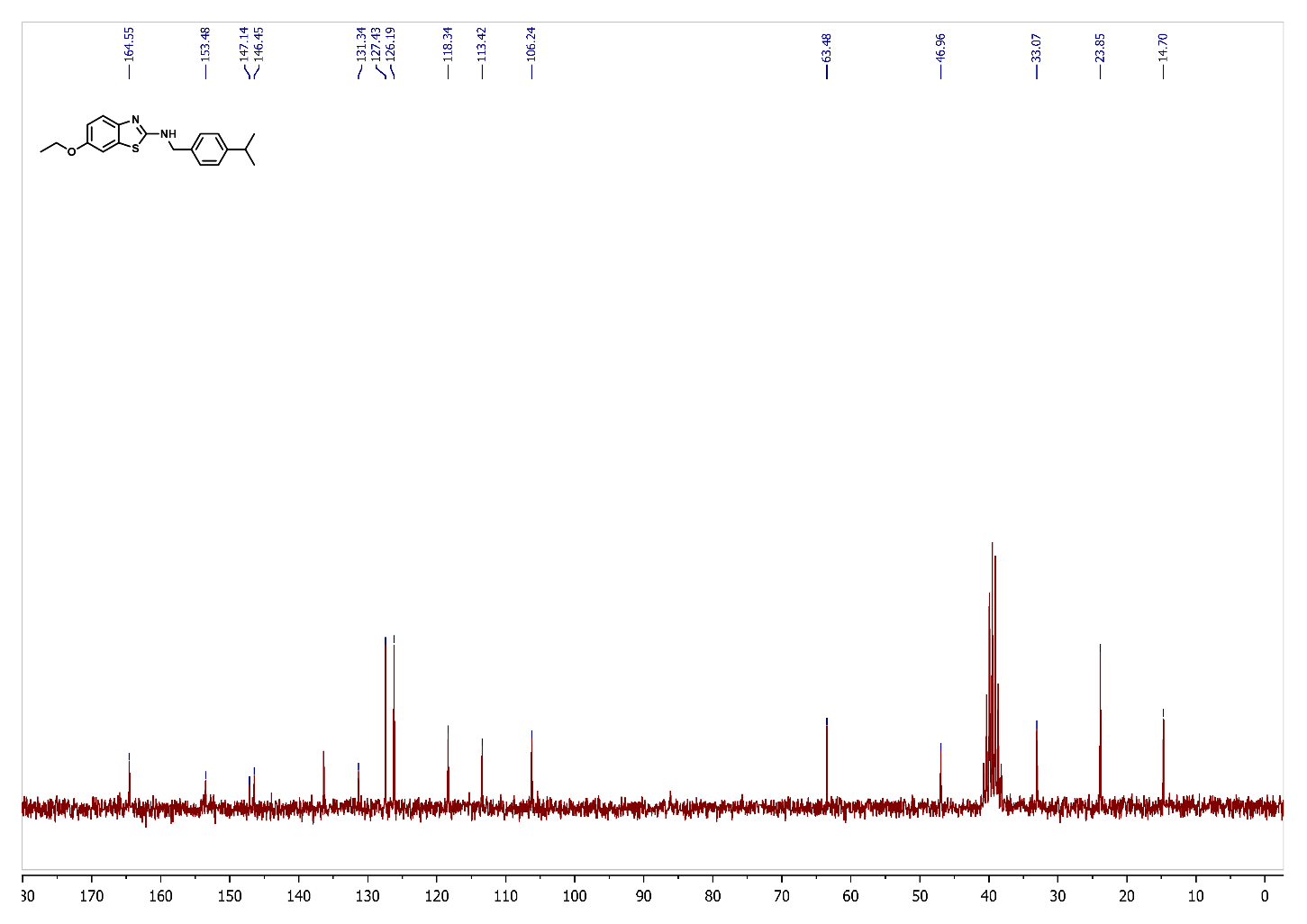


^1^H NMR spectrum (200 MHz, DMSO-d_6_) of compound BT-04

**
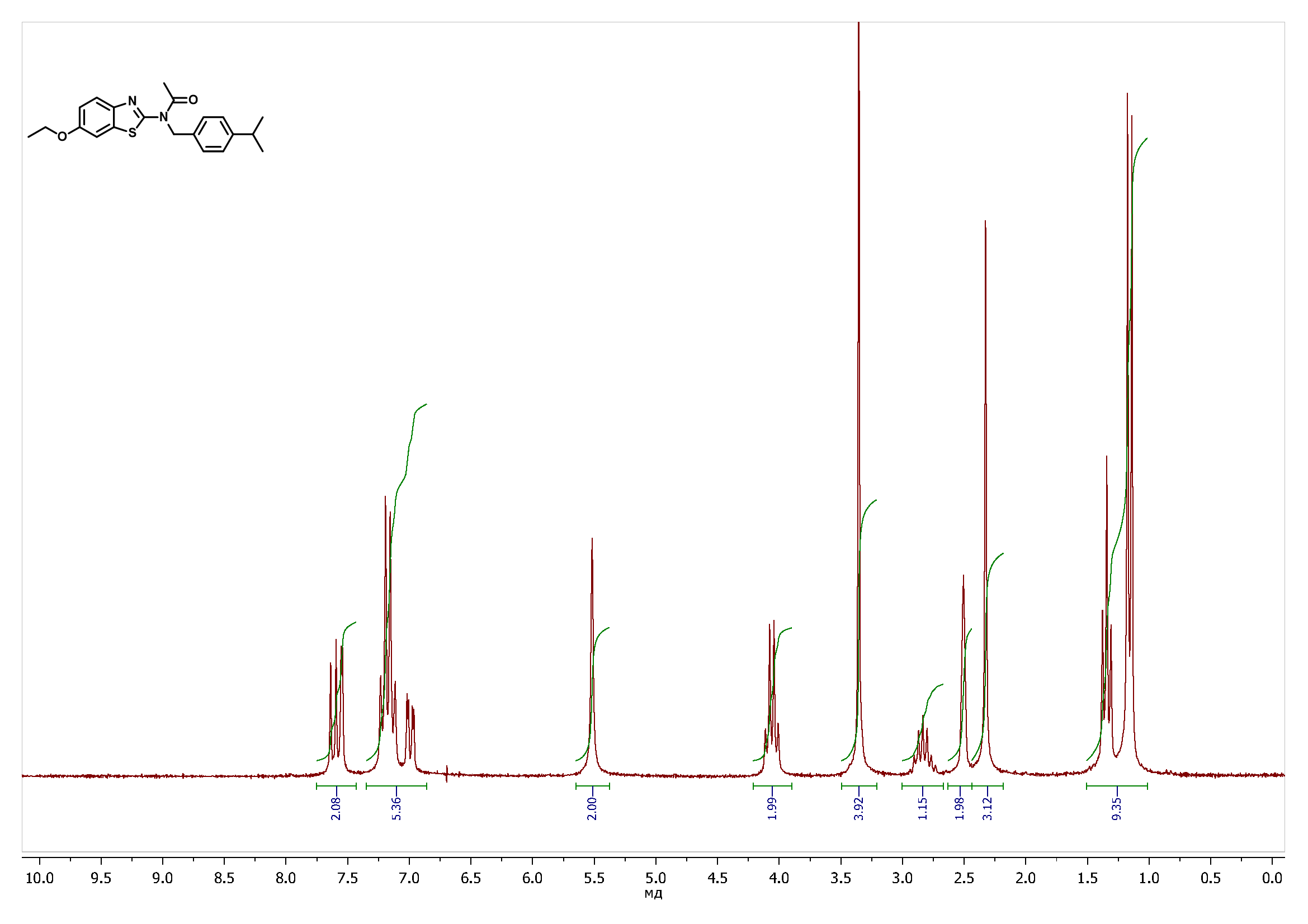
**

^13^C NMR spectrum (50 MHz, DMSO-d_6_) of compound BT-04

**
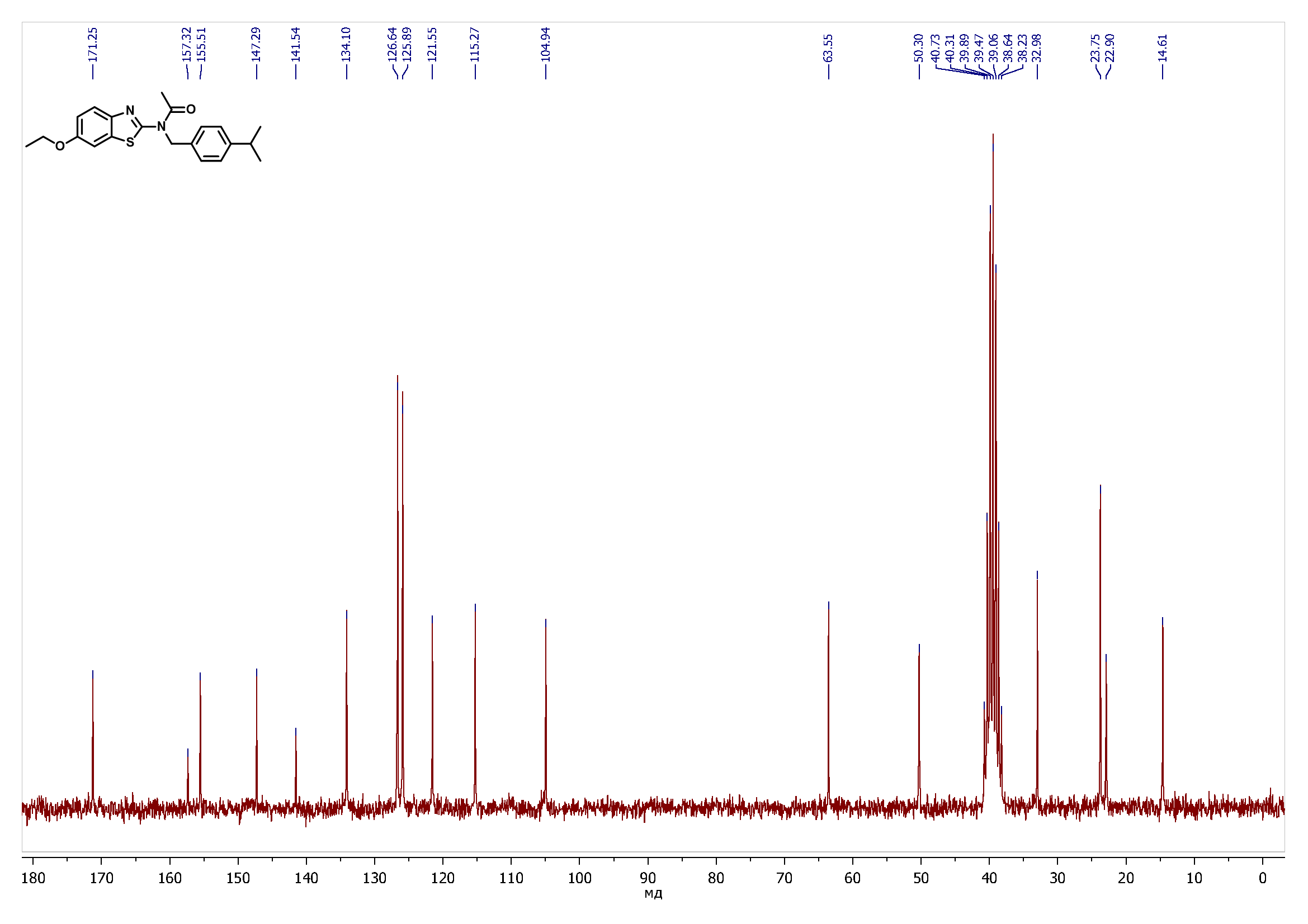
**

^1^H NMR spectrum (200 MHz, DMSO-d_6_) of compound BT-05

**
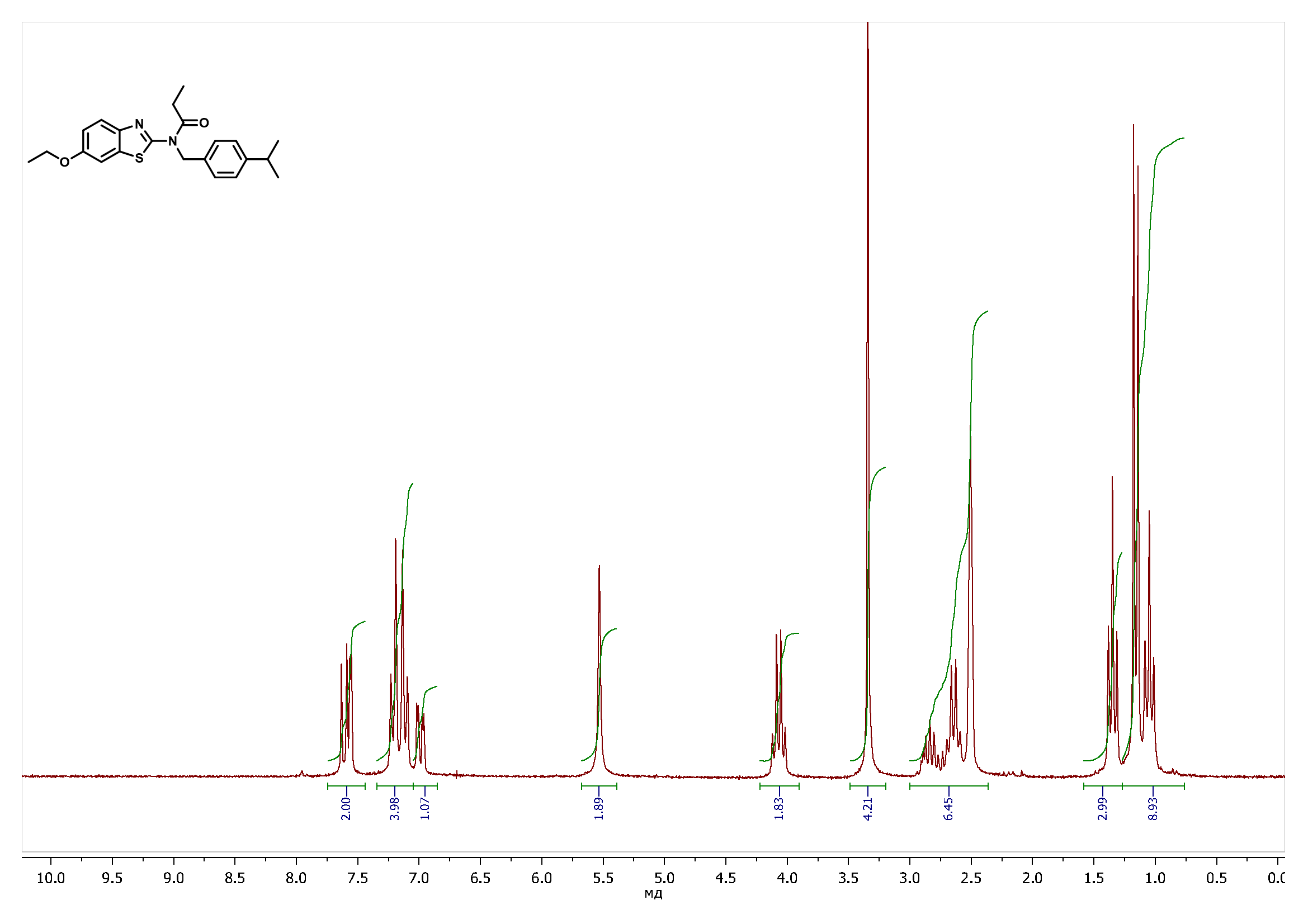
**

^13^C NMR spectrum (50 MHz, DMSO-d_6_) of compound BT-05

**
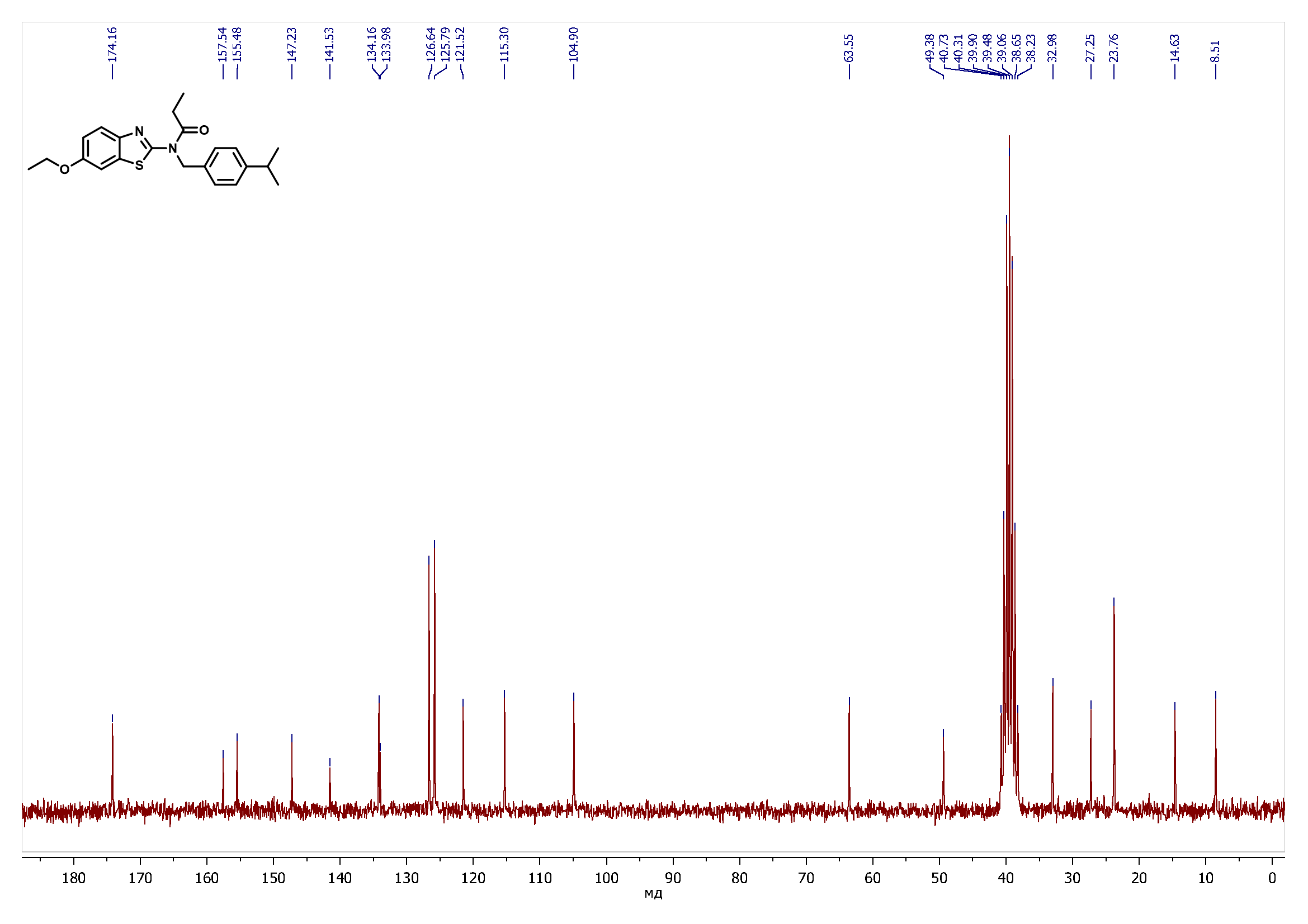
**

^1^H NMR spectrum (200 MHz, DMSO-d_6_) of compound BT-06

**
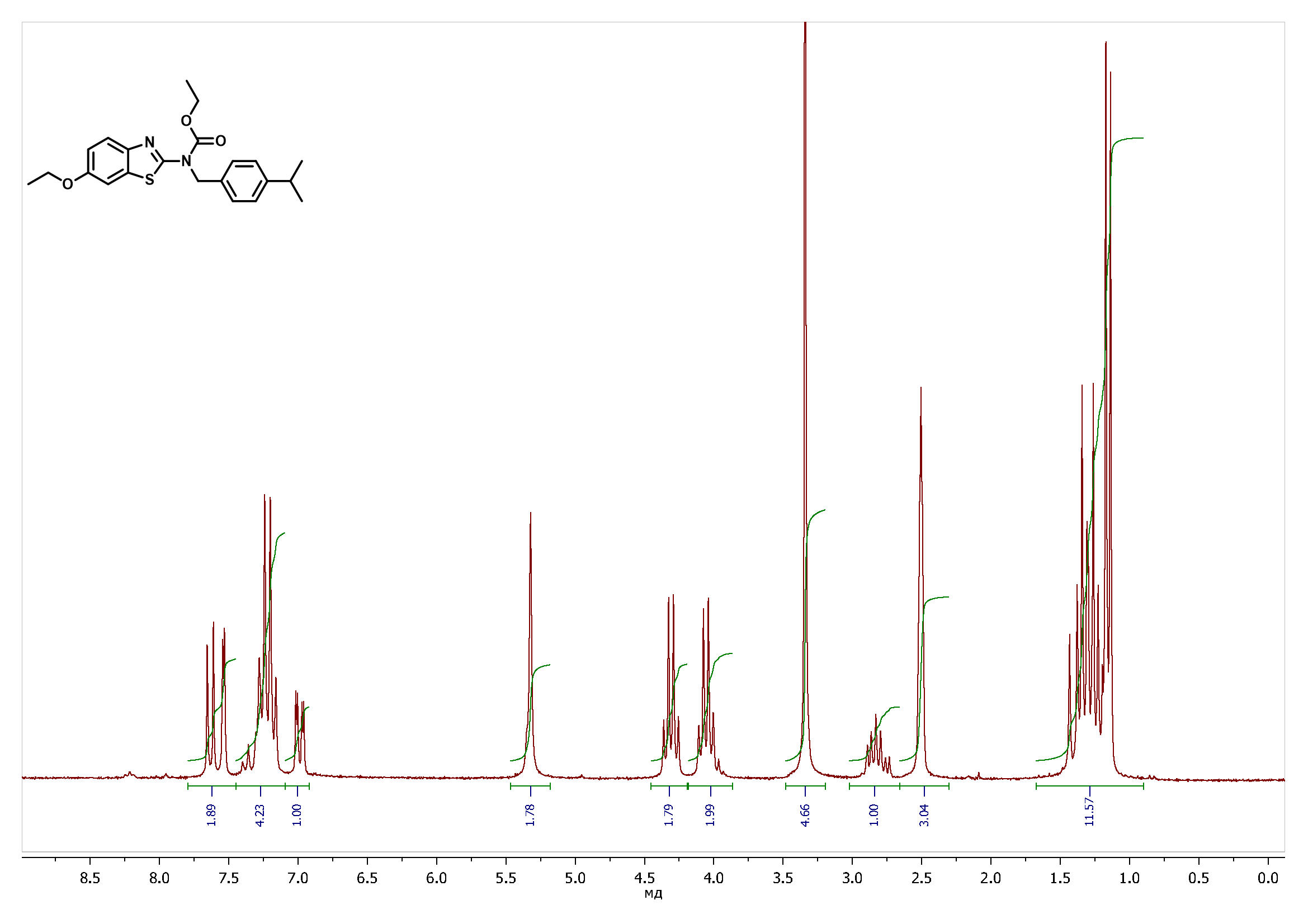
**

^13^C NMR spectrum (50 MHz, DMSO-d_6_) of compound BT-06

**
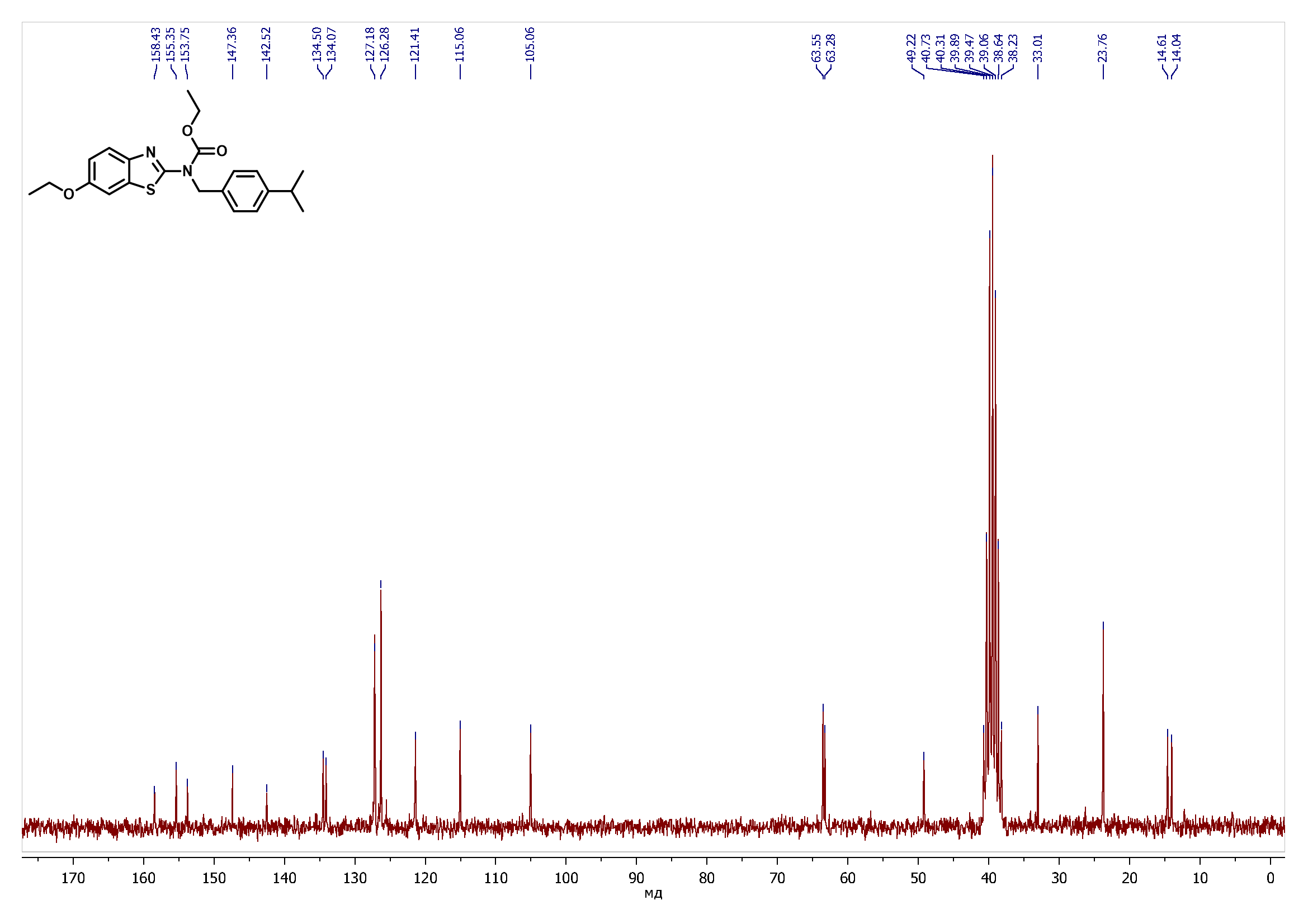
**

^1^H NMR spectrum (200 MHz, DMSO-d_6_) of compound BT-07

**
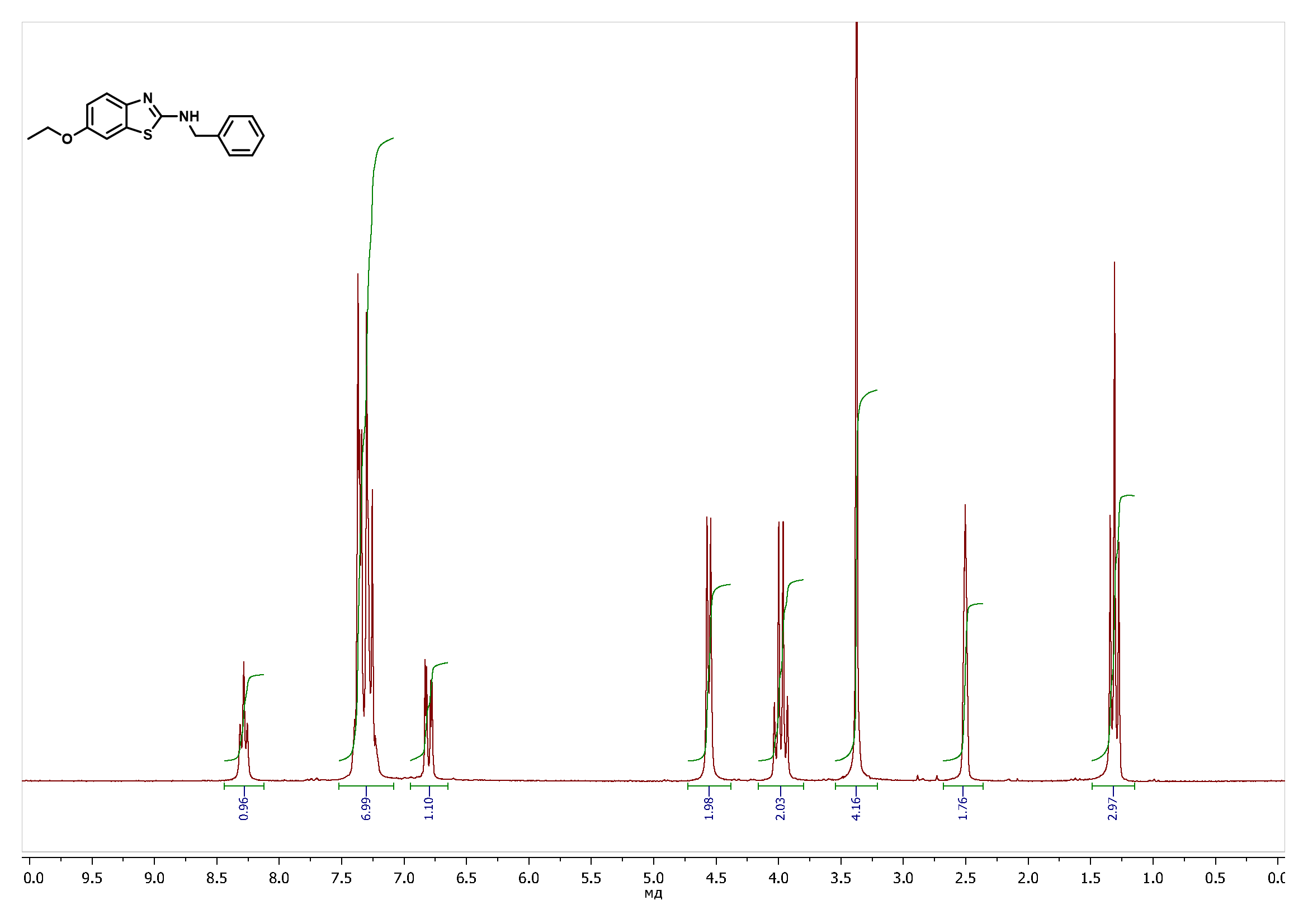
**

^13^C NMR spectrum (50 MHz, DMSO-d_6_) of compound BT-07

**
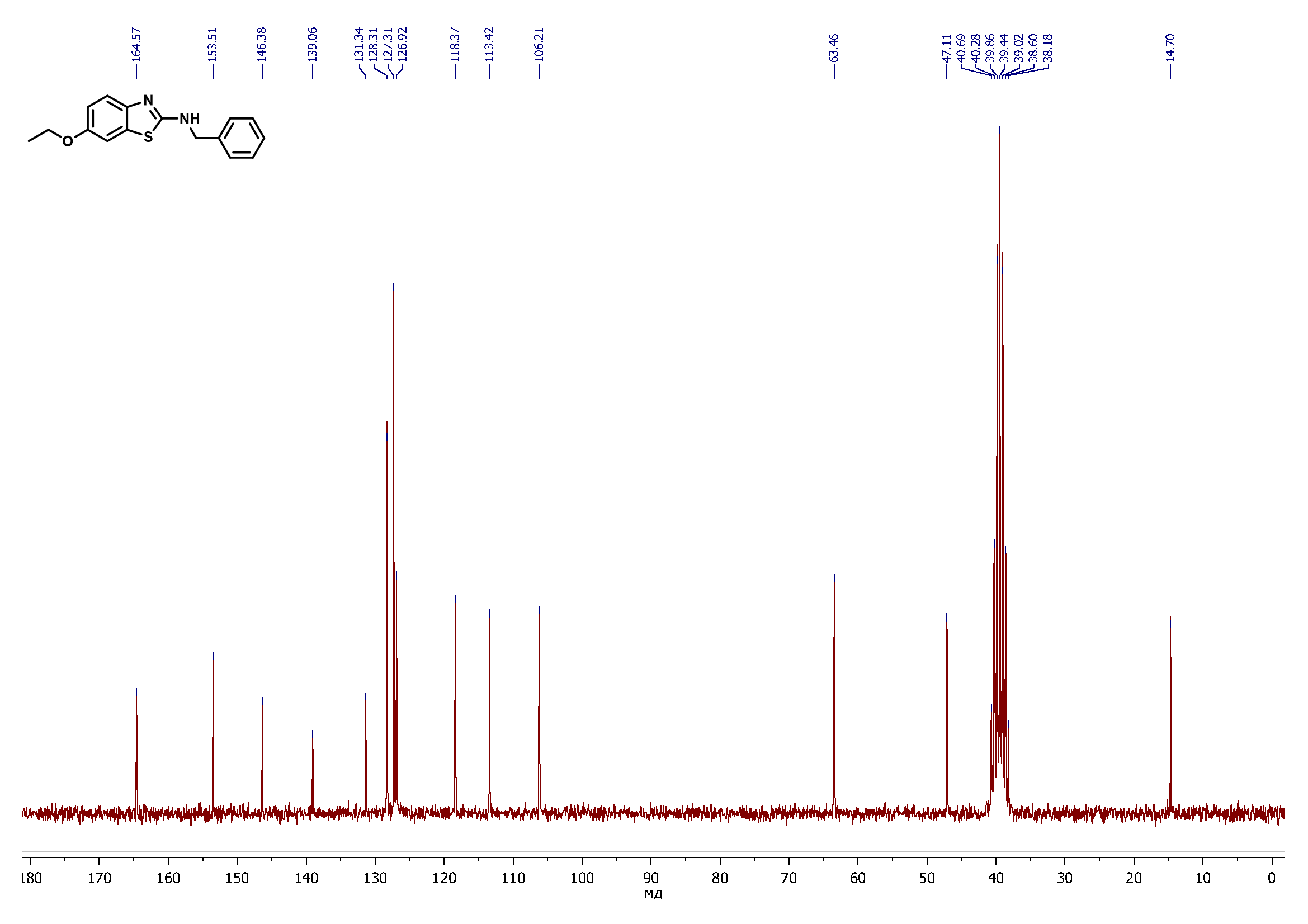
**

^1^H NMR spectrum (200 MHz, DMSO-d_6_) of compound BT-08

**
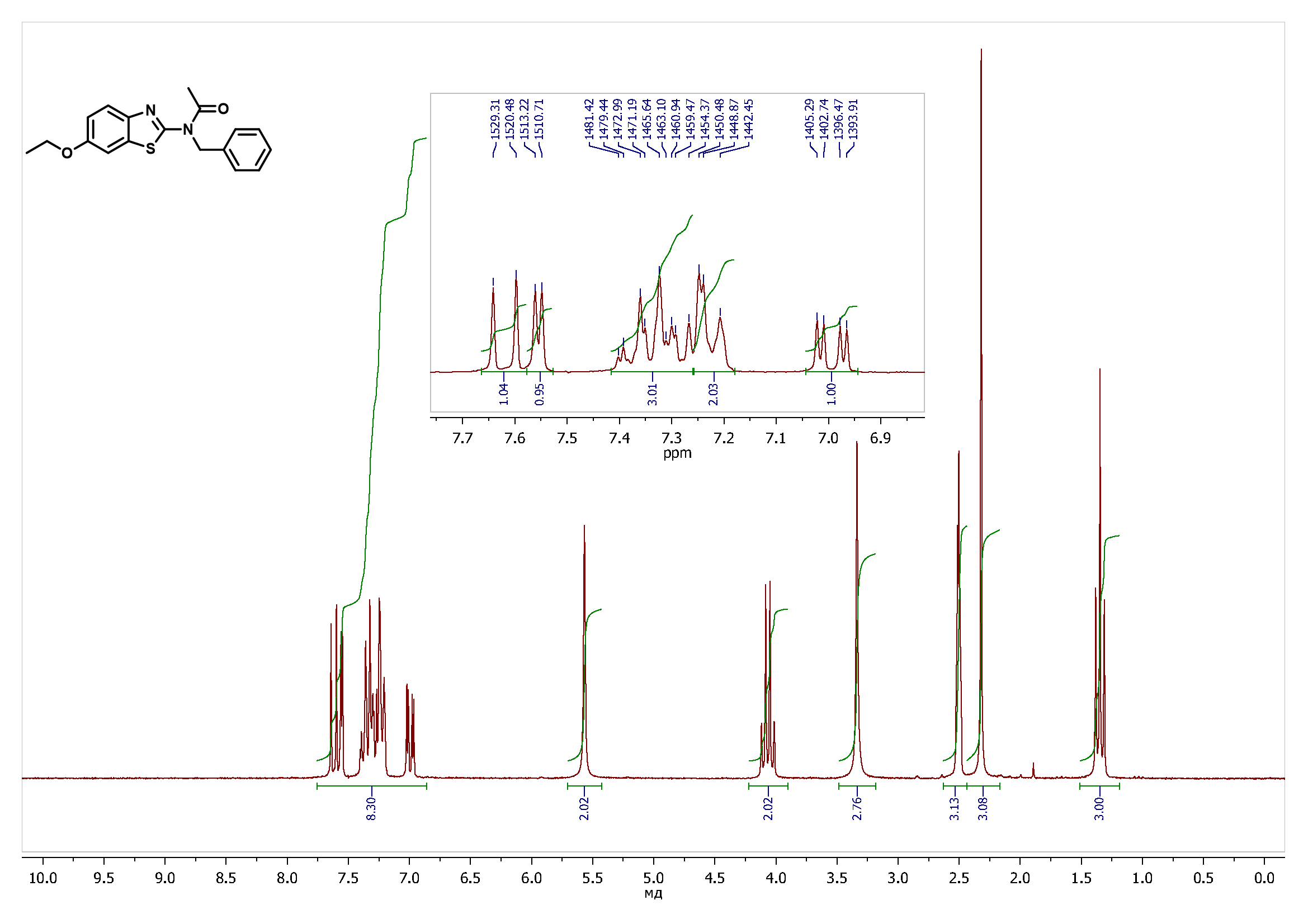
**

^13^C NMR spectrum (50 MHz, DMSO-d_6_) of compound BT-08


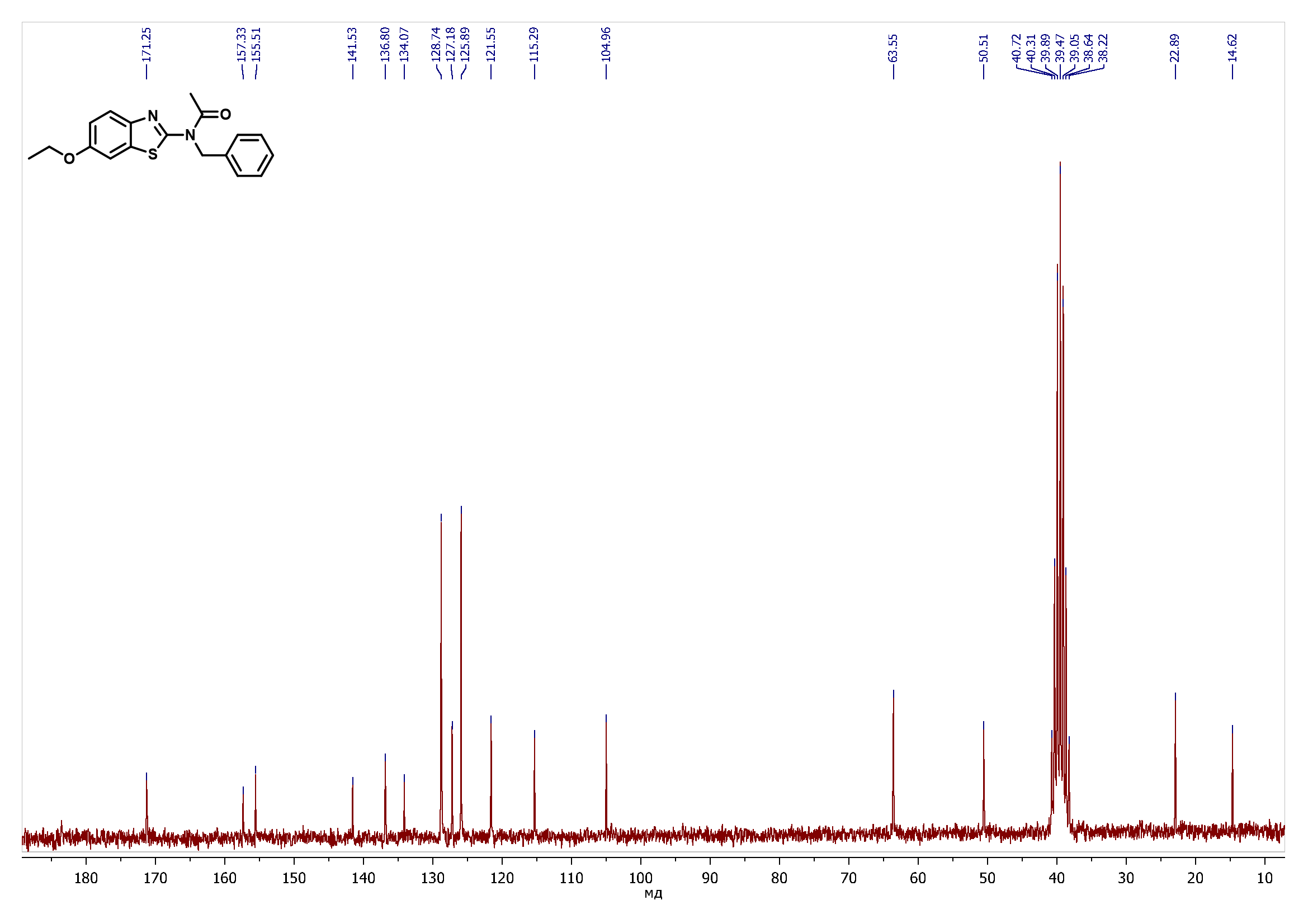


^1^H NMR spectrum (200 MHz, DMSO-d_6_) of compound BT-09

**
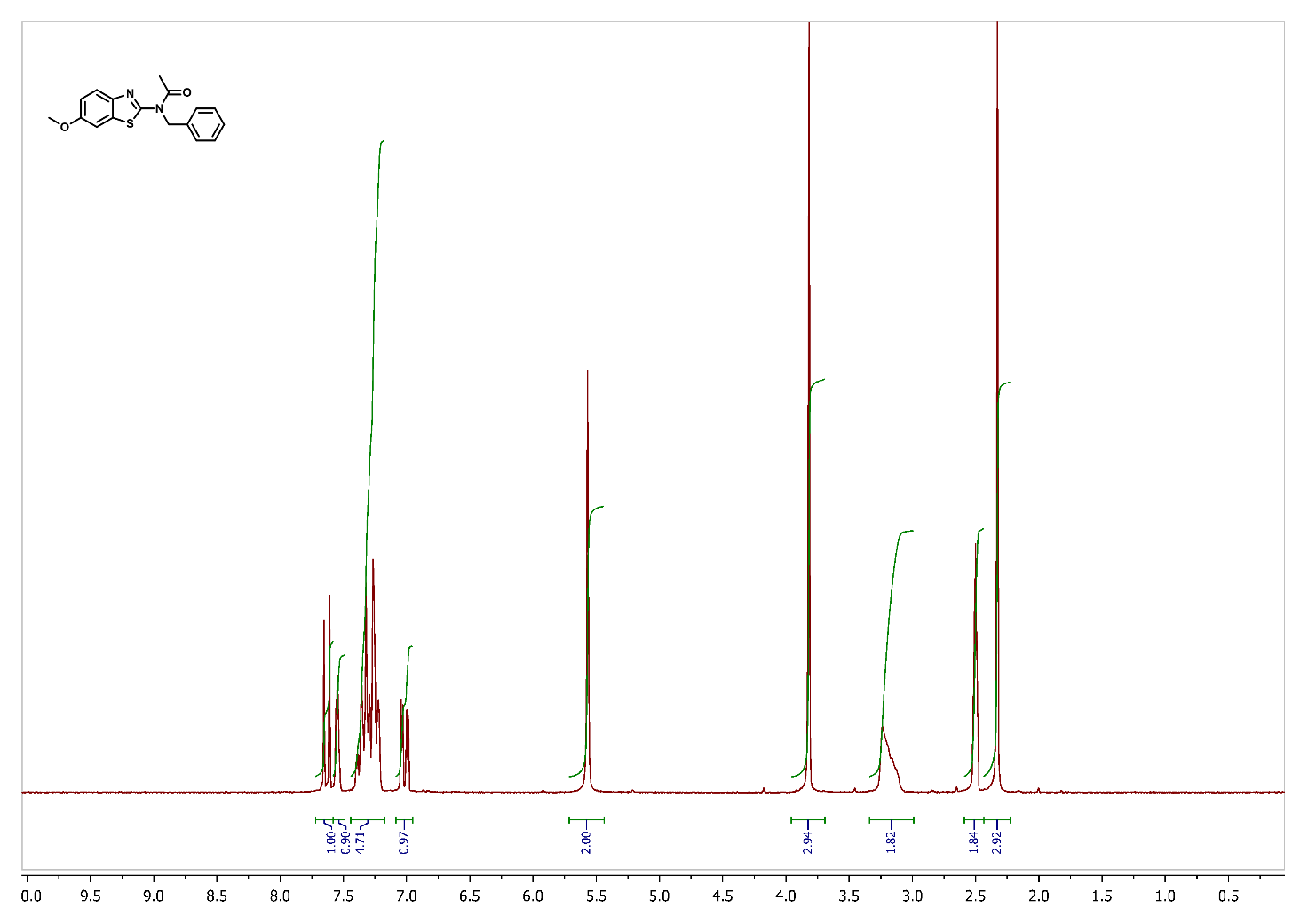
**

^13^C NMR spectrum (50 MHz, DMSO-d_6_) of compound BT-09


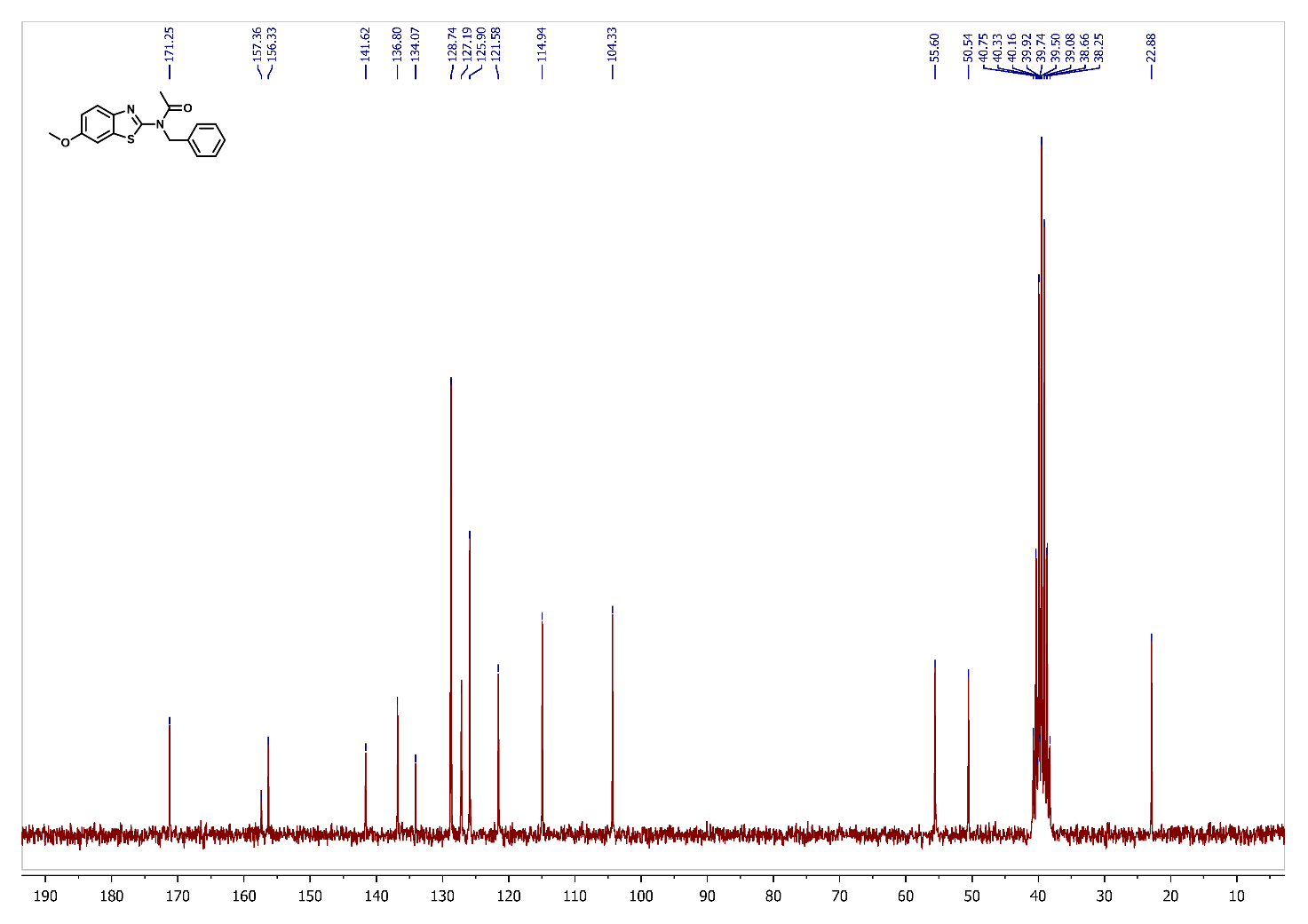


^1^H NMR spectrum (200 MHz, DMSO-d_6_) of compound BT-10

**
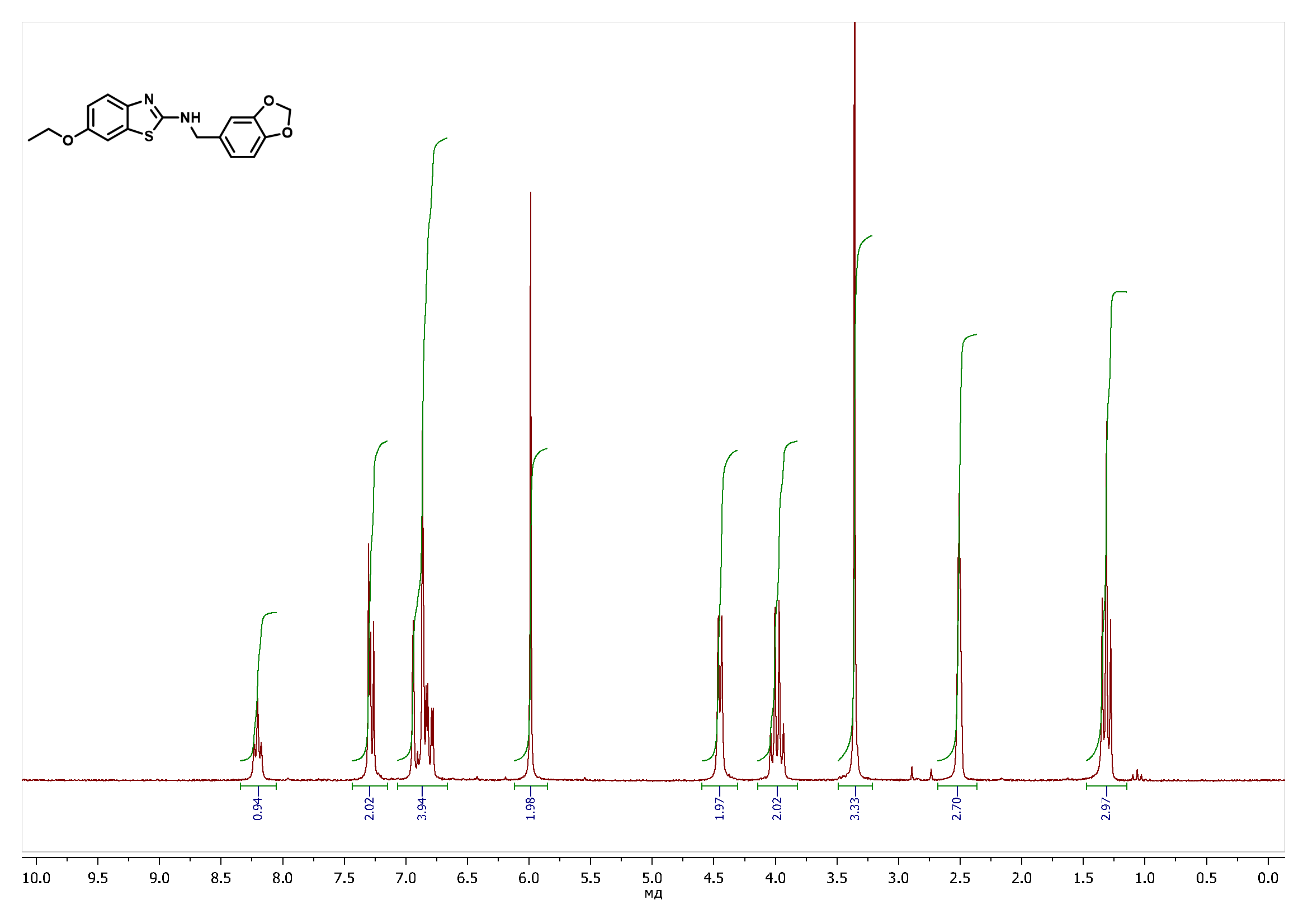
**

^13^C NMR spectrum (50 MHz, DMSO-d_6_) of compound BT-10

**
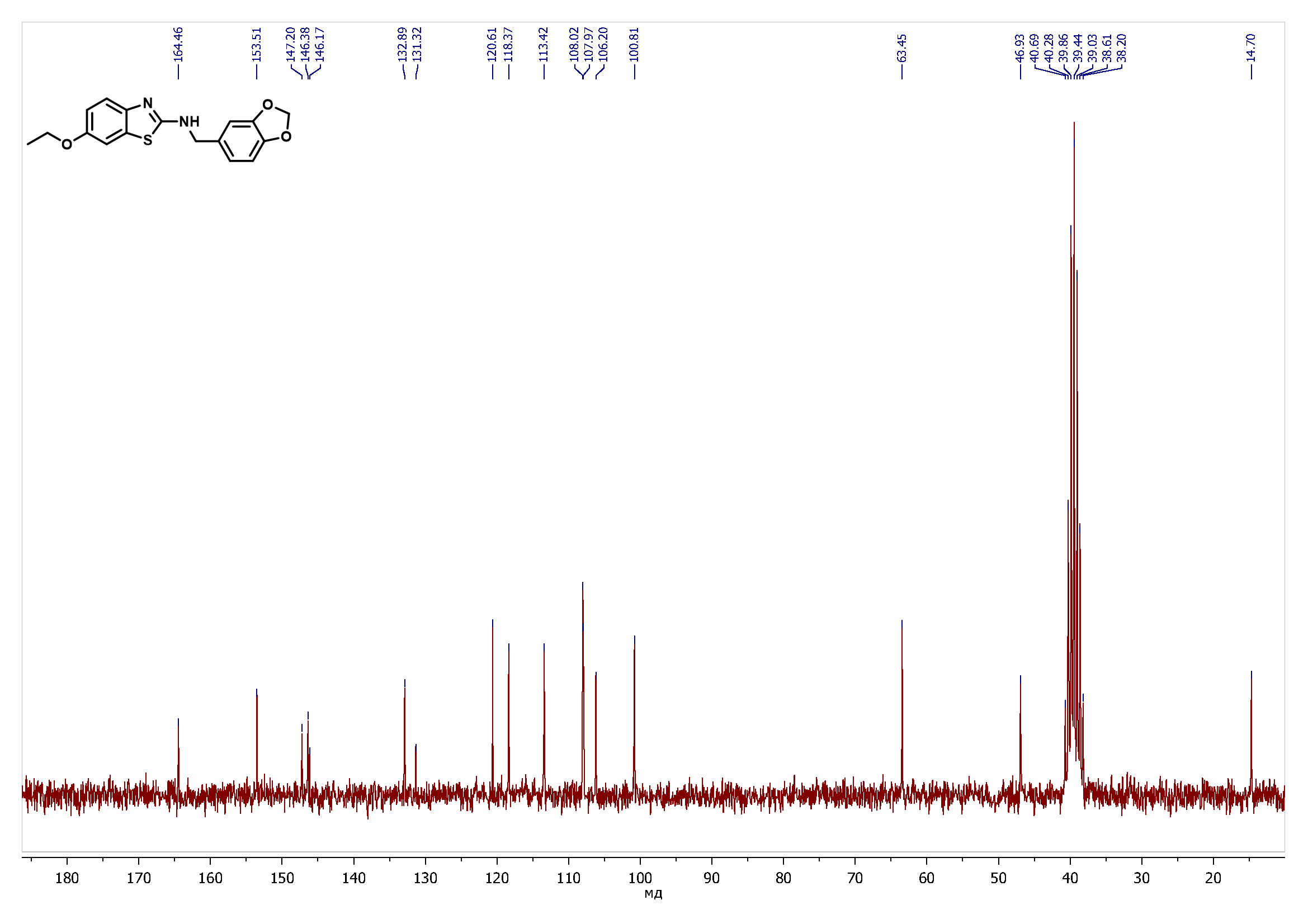
**

^1^H NMR spectrum (200 MHz, DMSO-d_6_) of compound BT-11

**
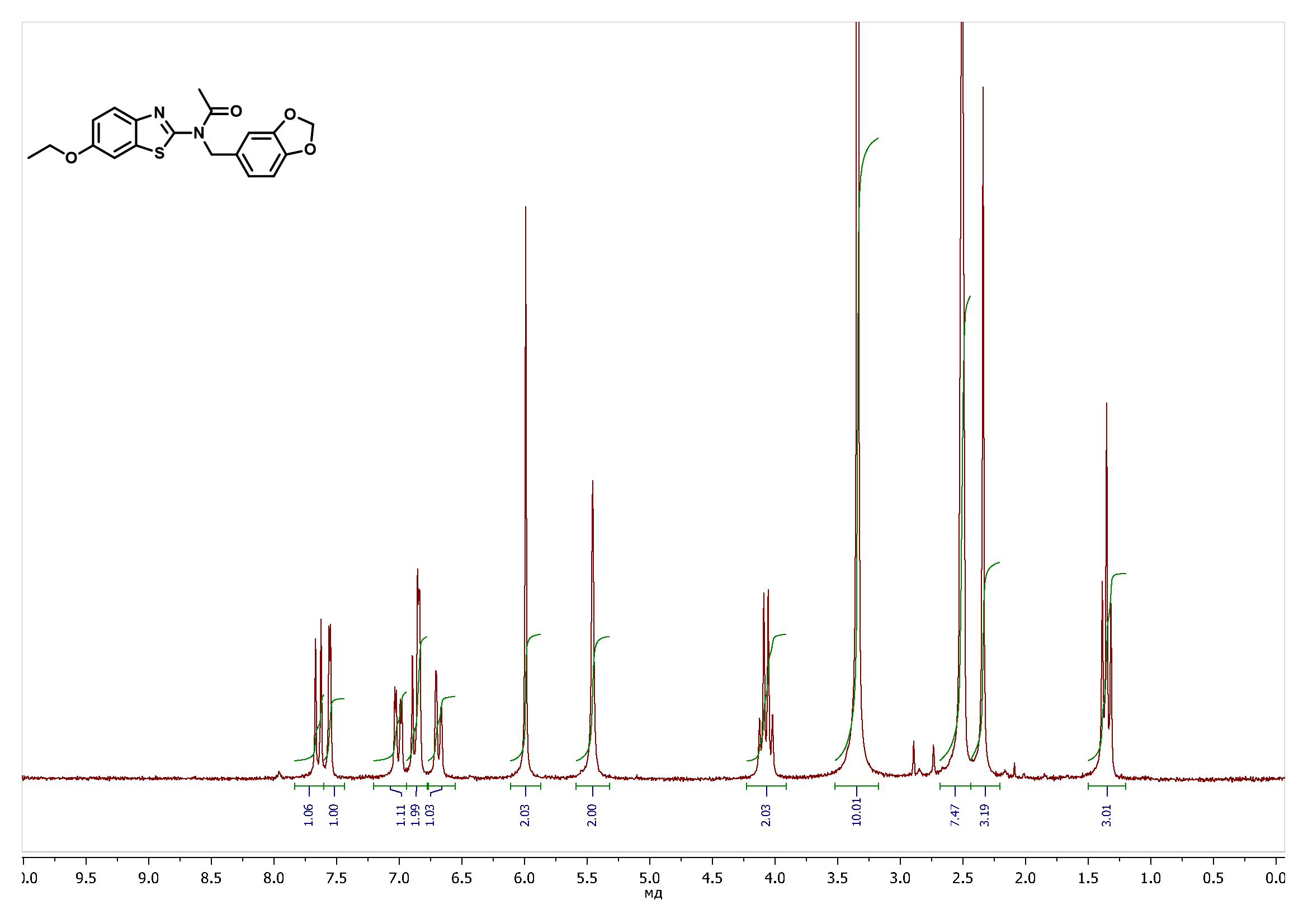
**

^13^C NMR spectrum (50 MHz, DMSO-d_6_) of compound BT-11


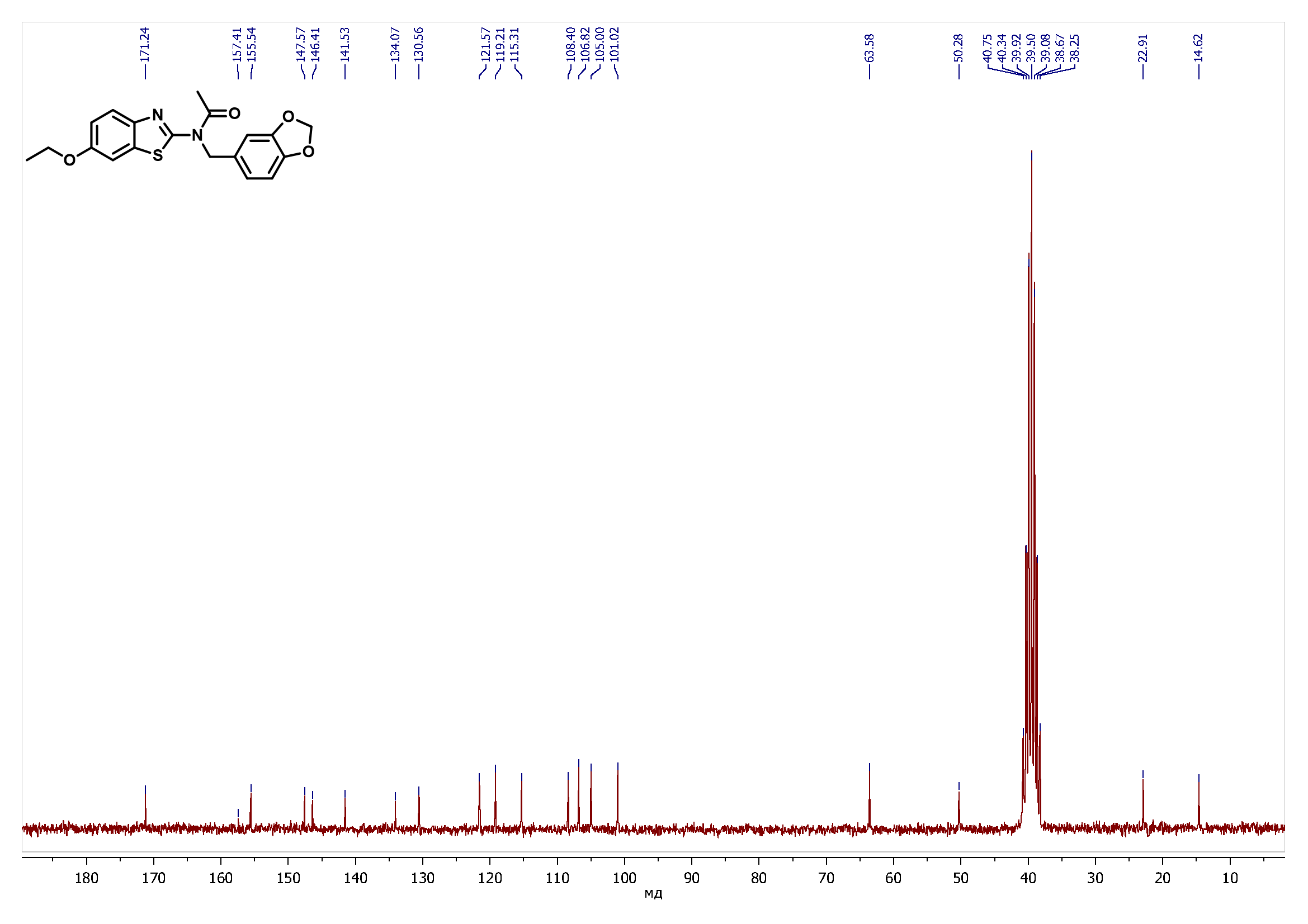


^1^H NMR spectrum (200 MHz, DMSO-d_6_) of compound BT-12

**
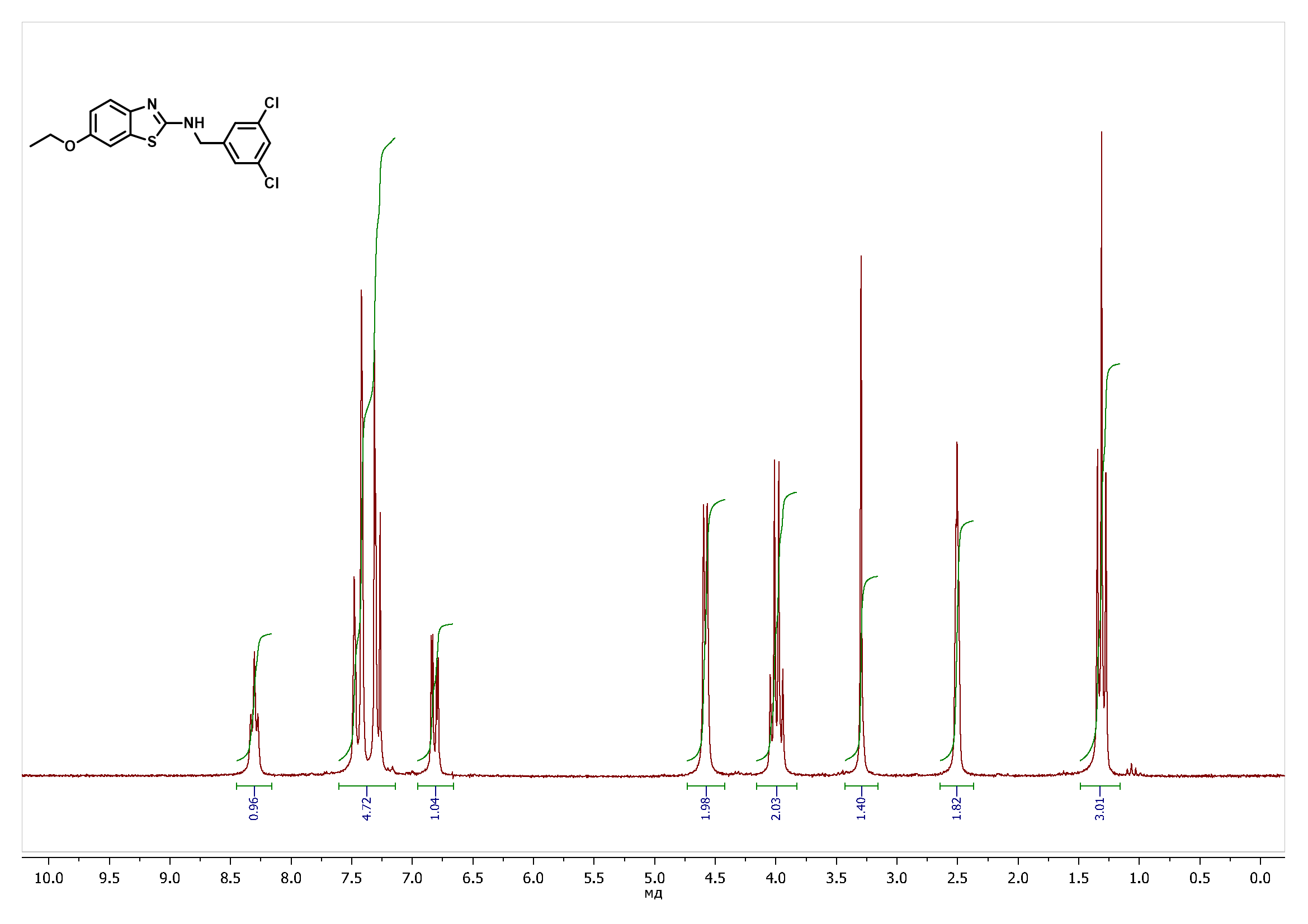
**

^13^C NMR spectrum (50 MHz, DMSO-d_6_) of compound BT-12

**
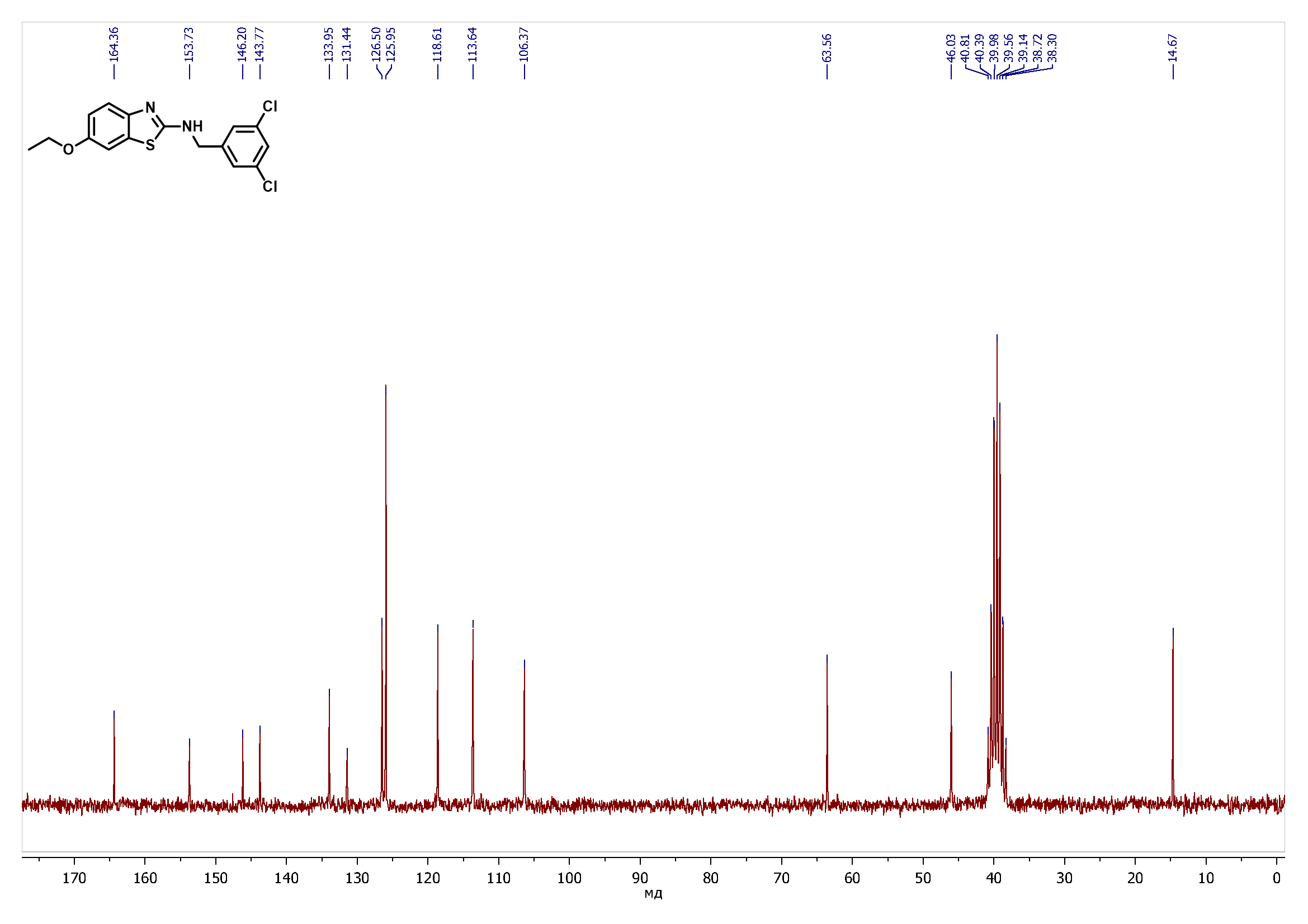
**

^1^H NMR spectrum (200 MHz, DMSO-d_6_) of compound BT-13

**
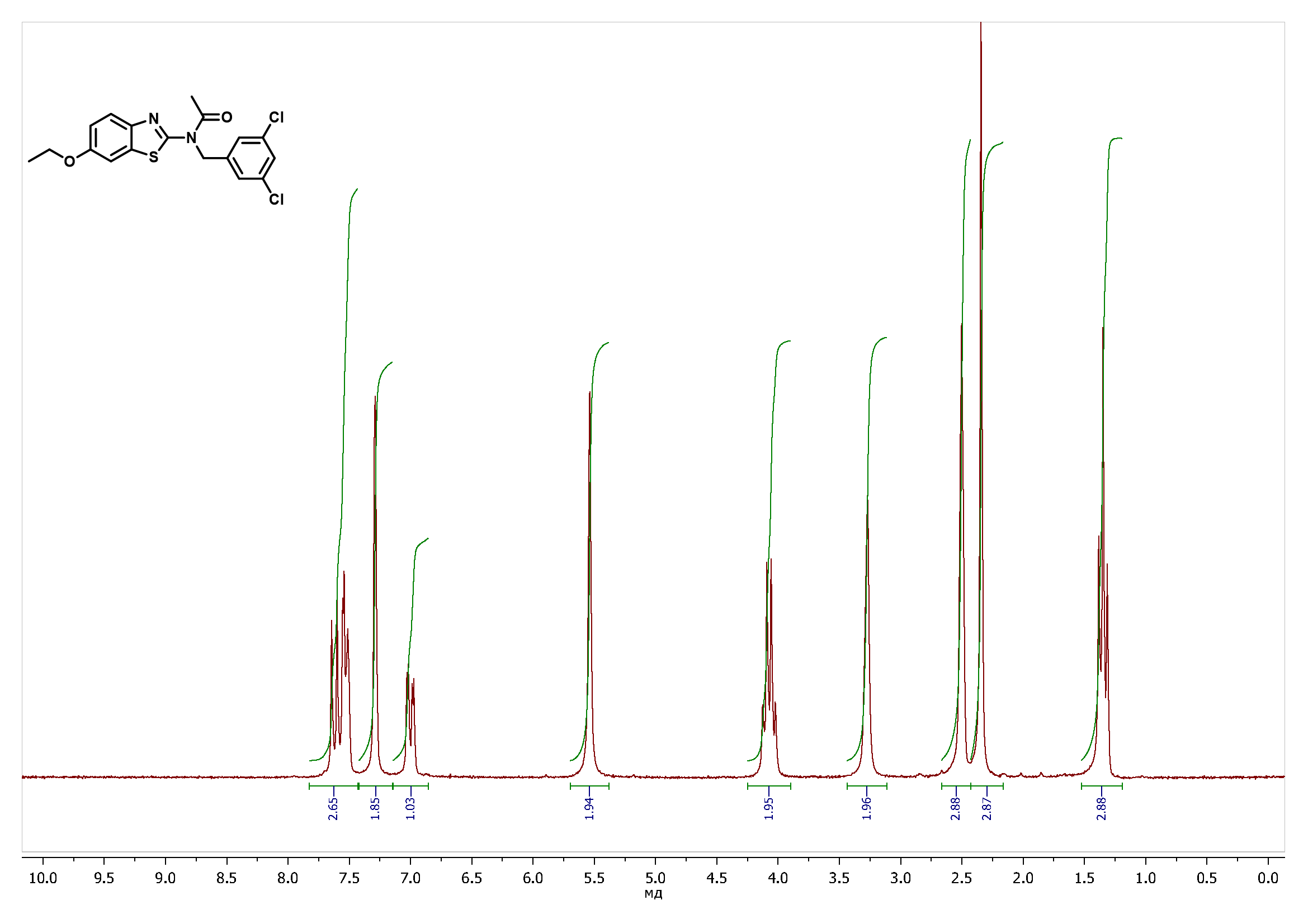
**

^13^C NMR spectrum (50 MHz, DMSO-d_6_) of compound BT-13

**
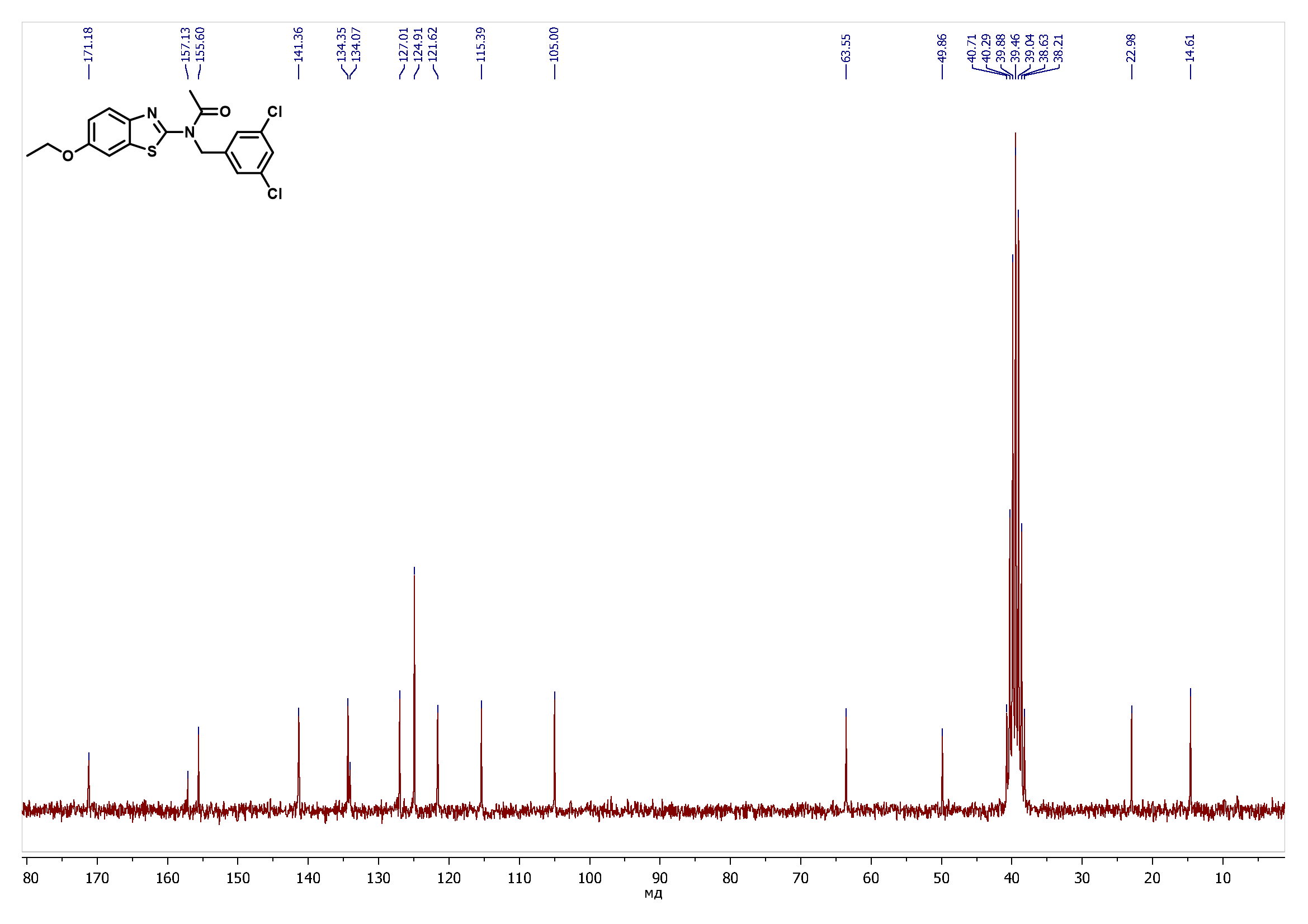
**

^1^H NMR spectrum (200 MHz, DMSO-d_6_) of compound BT-14

**
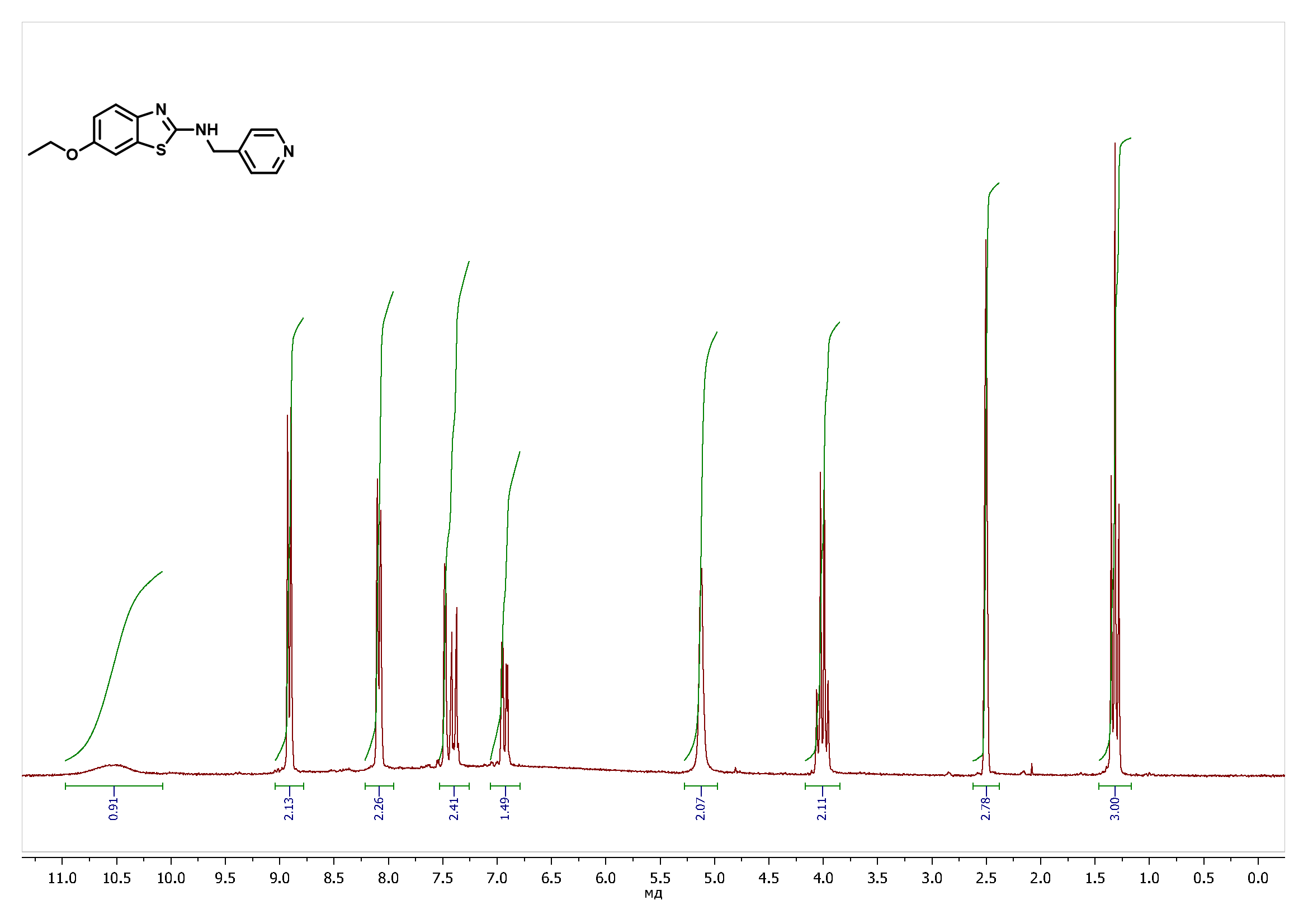
**

^13^C NMR spectrum (50 MHz, DMSO-d_6_) of compound BT-14

**
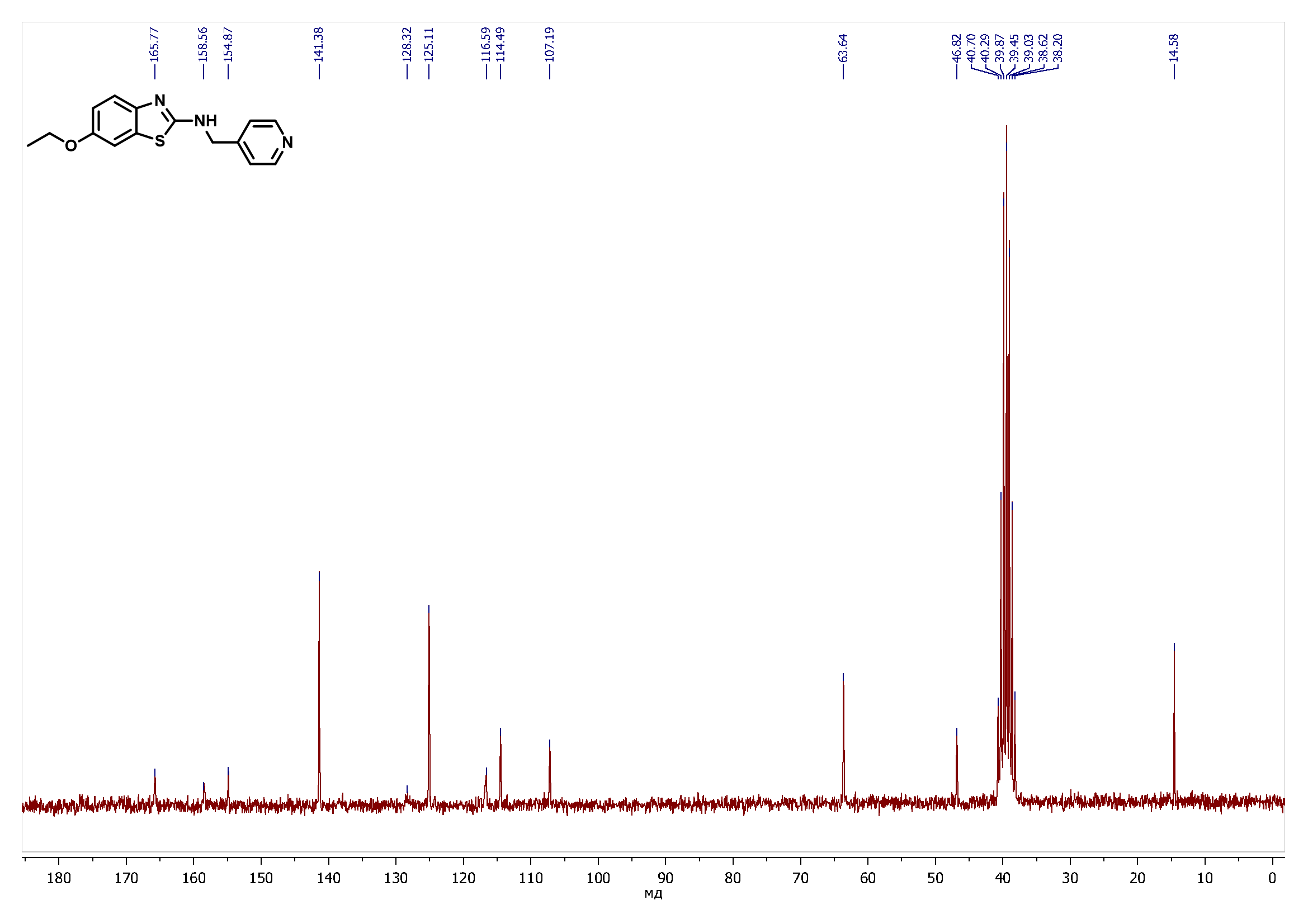
**

^1^H NMR spectrum (200 MHz, DMSO-d_6_) of compound BT-15

**
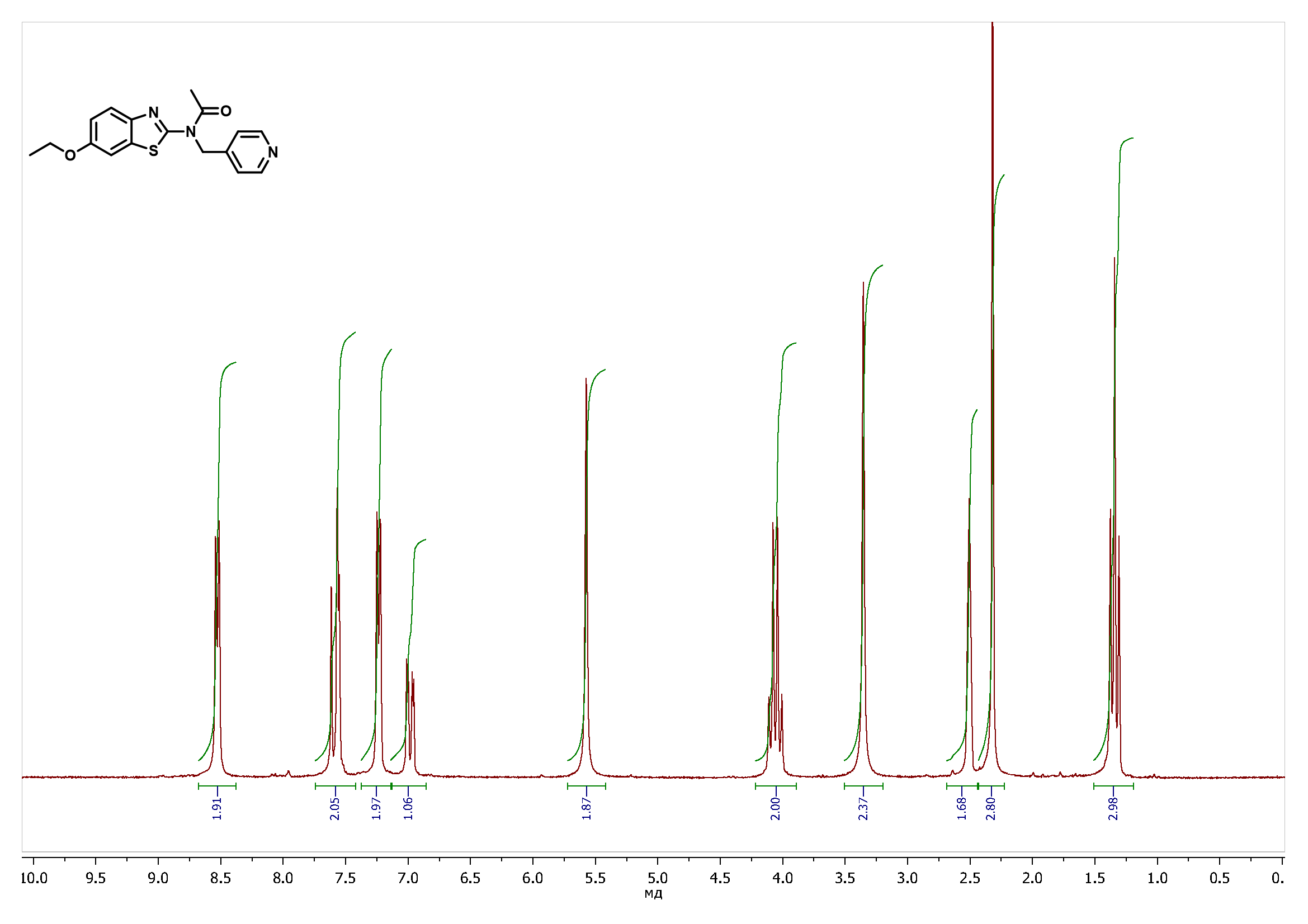
**

^13^C NMR spectrum (50 MHz, DMSO-d_6_) of compound BT-15

**
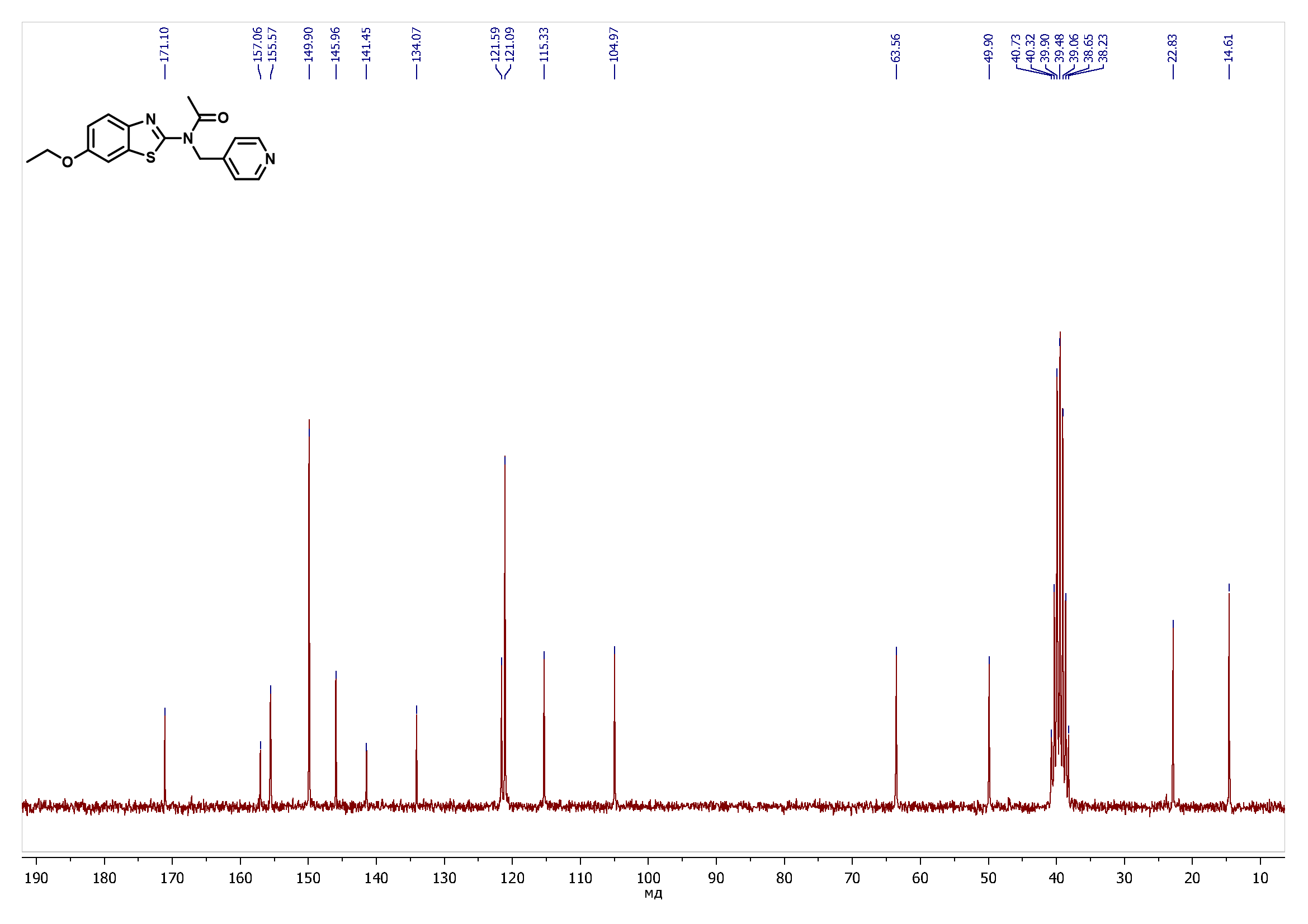
**

^1^H NMR spectrum (200 MHz, DMSO-d_6_) of compound BT-16

**
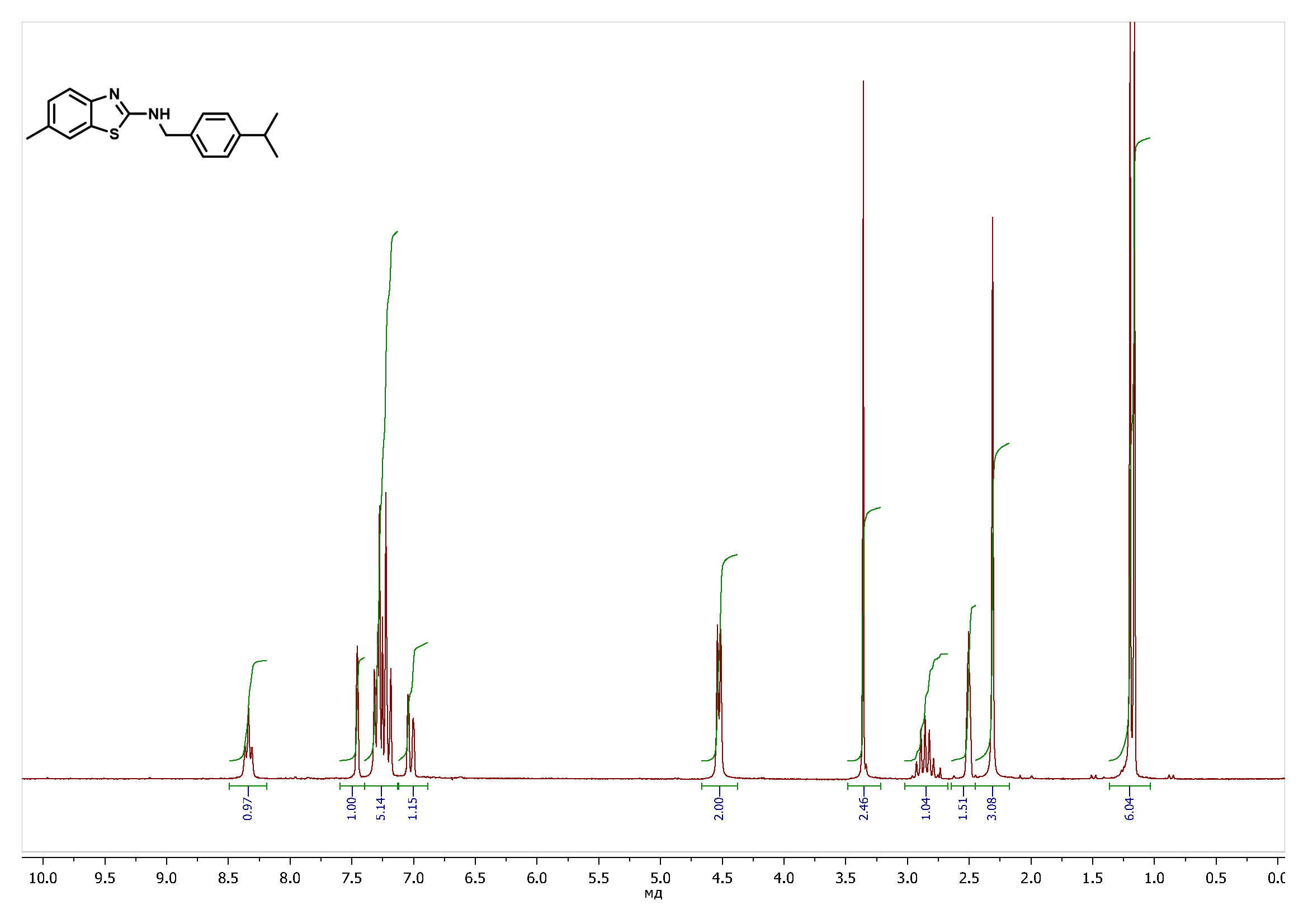
**

^13^C NMR spectrum (50 MHz, DMSO-d_6_) of compound BT-16

**
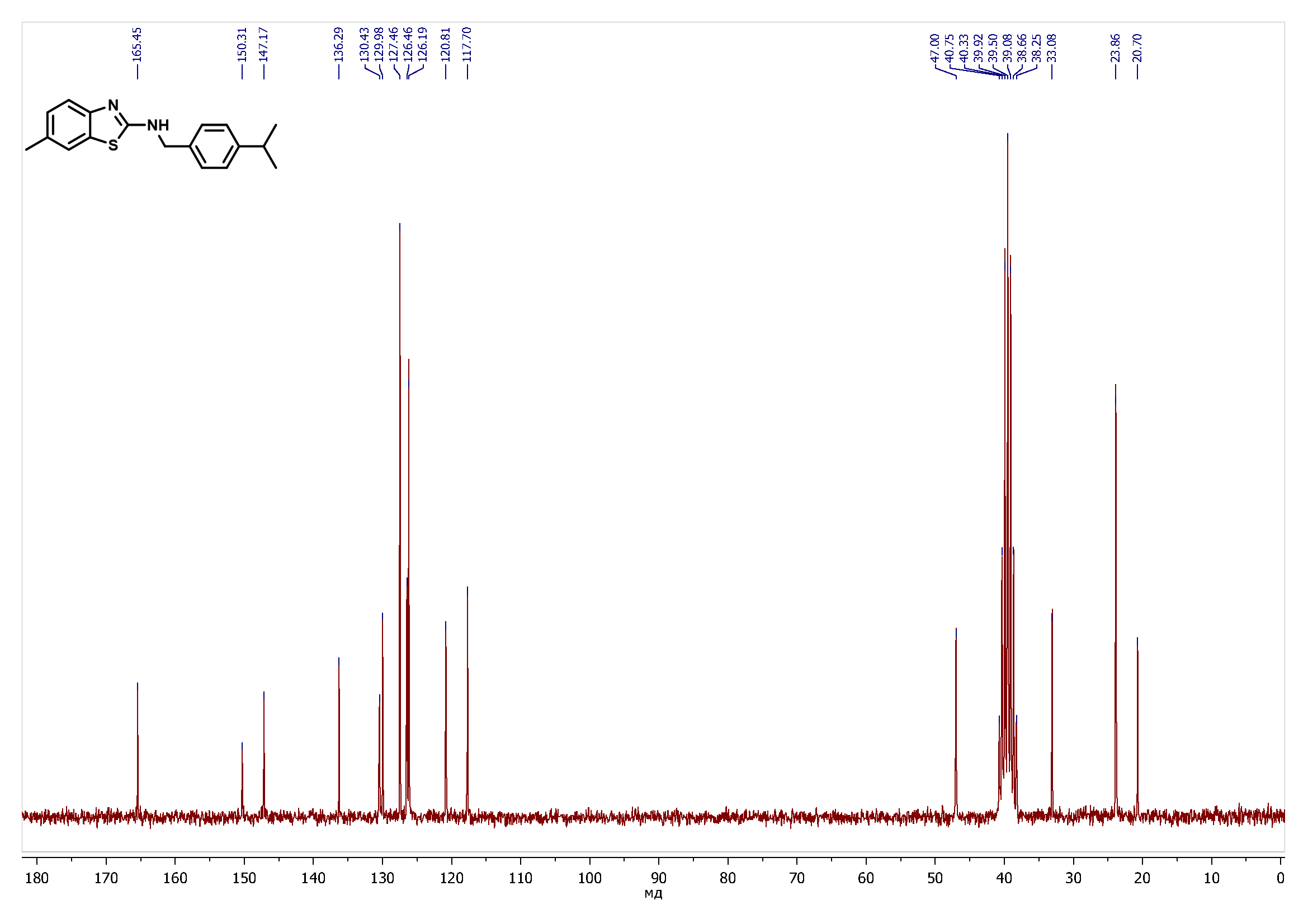
**

^1^H NMR spectrum (200 MHz, DMSO-d_6_) of compound BT-17

**
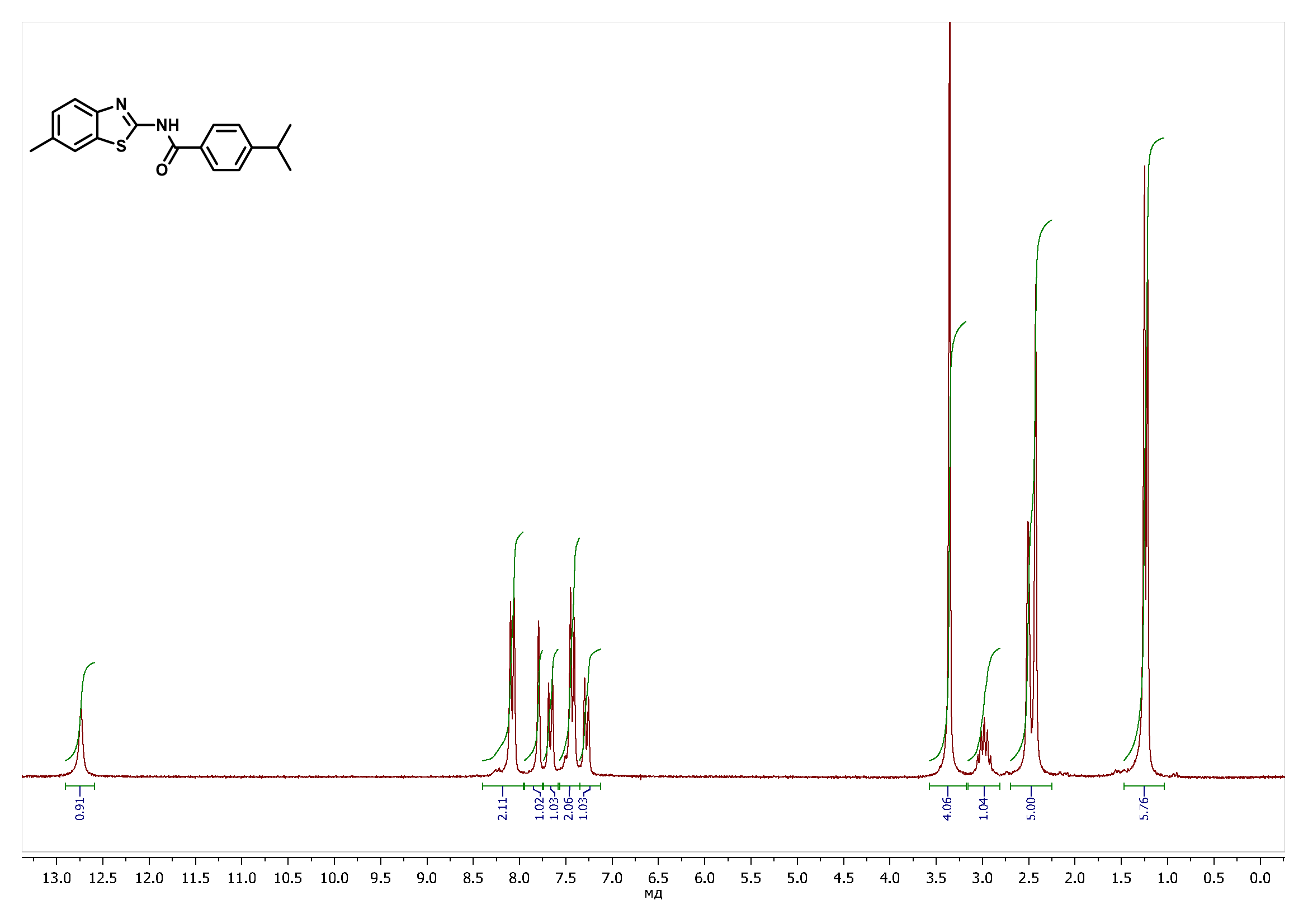
**

^13^C NMR spectrum (50 MHz, DMSO-d_6_) of compound BT-17

**
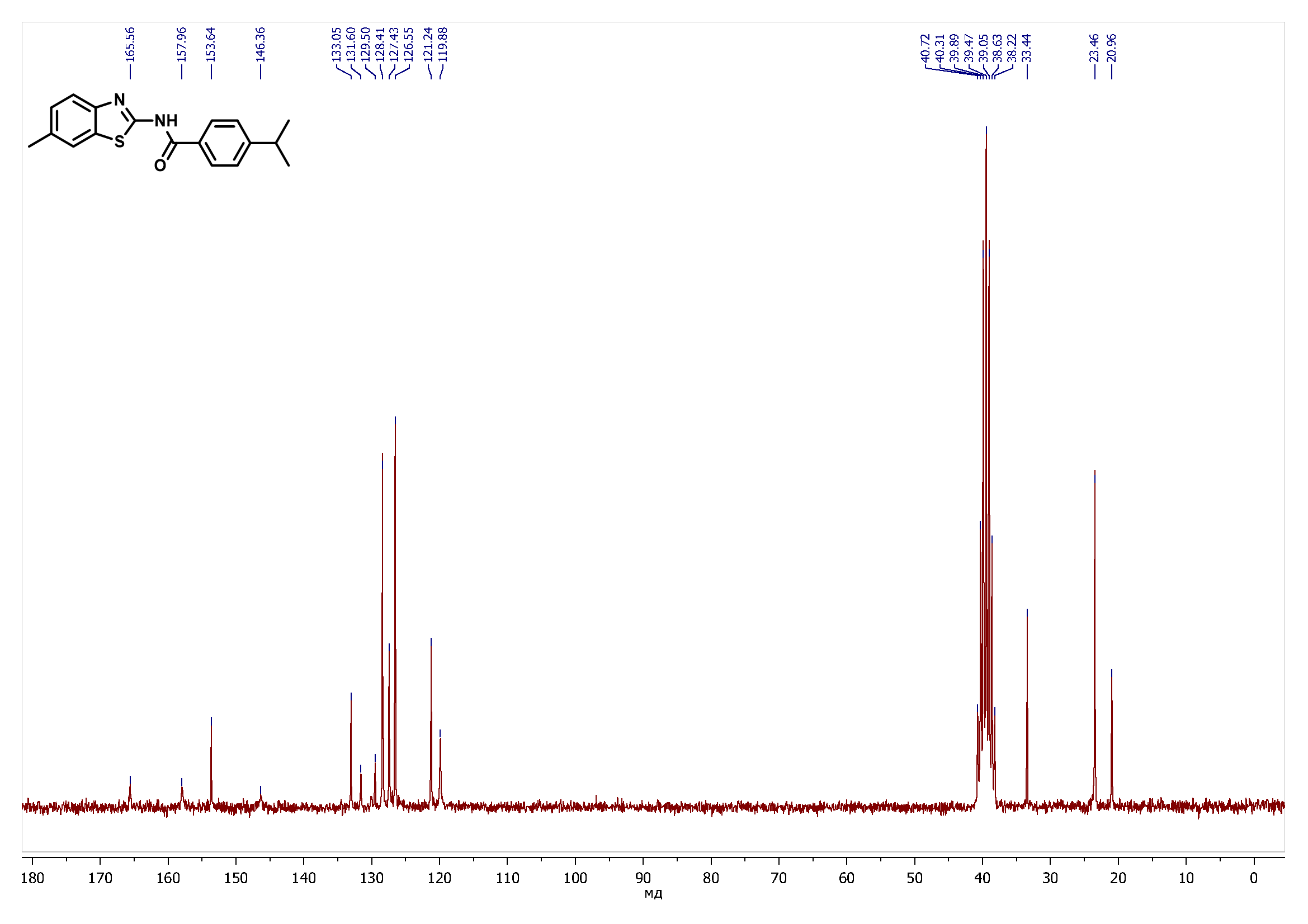
**

^1^H NMR spectrum (200 MHz, DMSO-d_6_) of compound BT-18

**
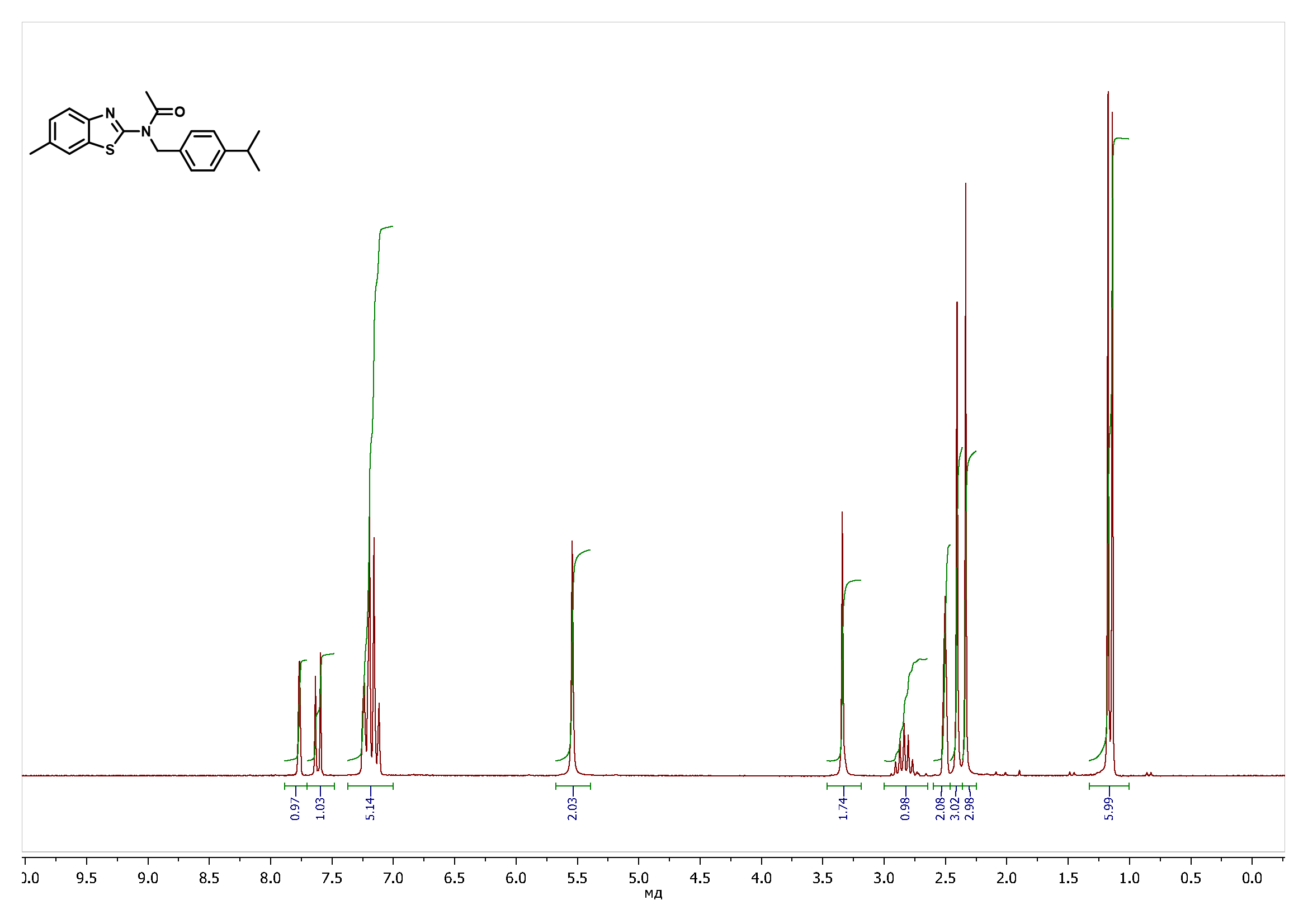
**

^13^C NMR spectrum (50 MHz, DMSO-d_6_) of compound BT-18

**
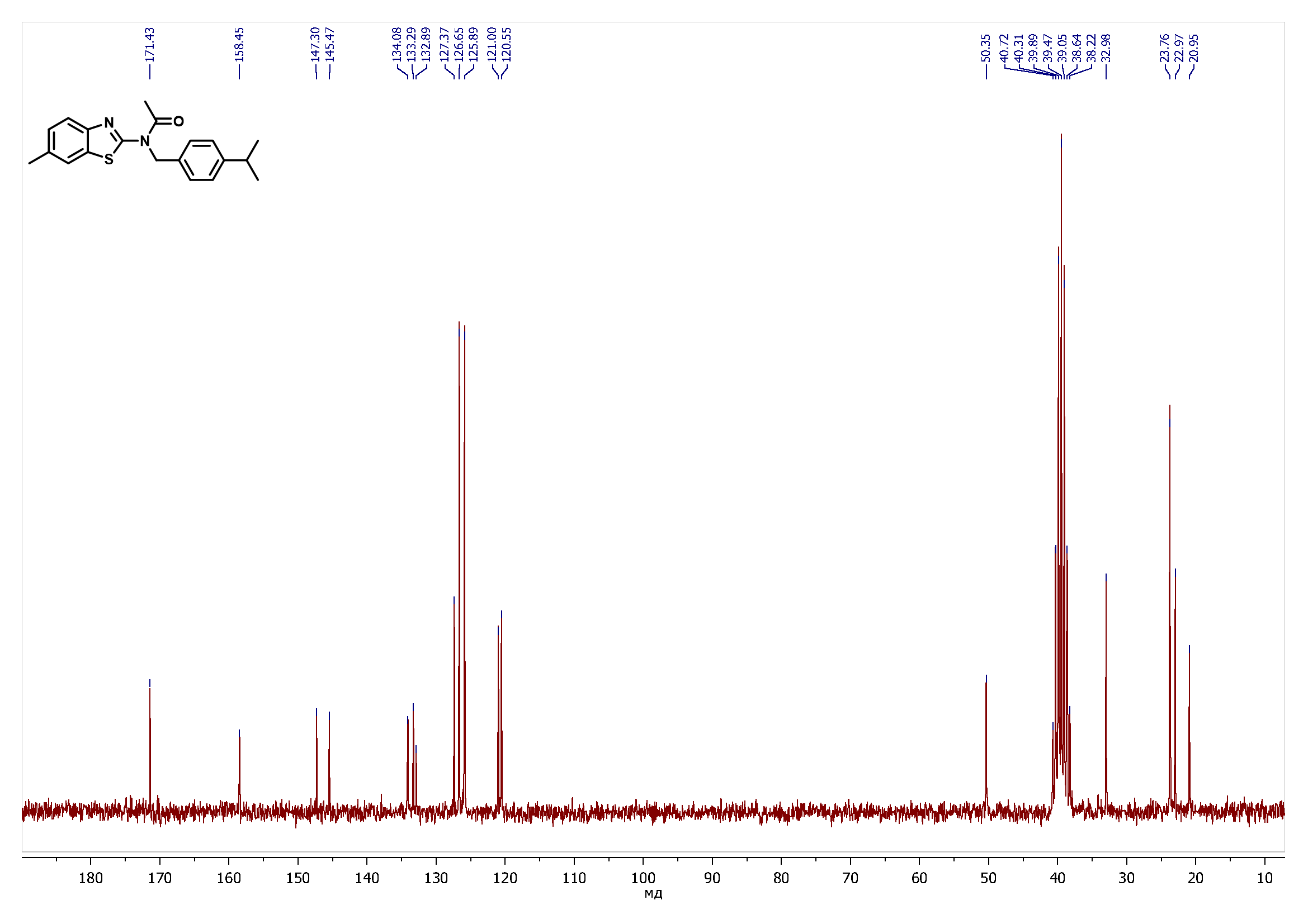
**

^1^H NMR spectrum (200 MHz, DMSO-d_6_) of compound BT-19

**
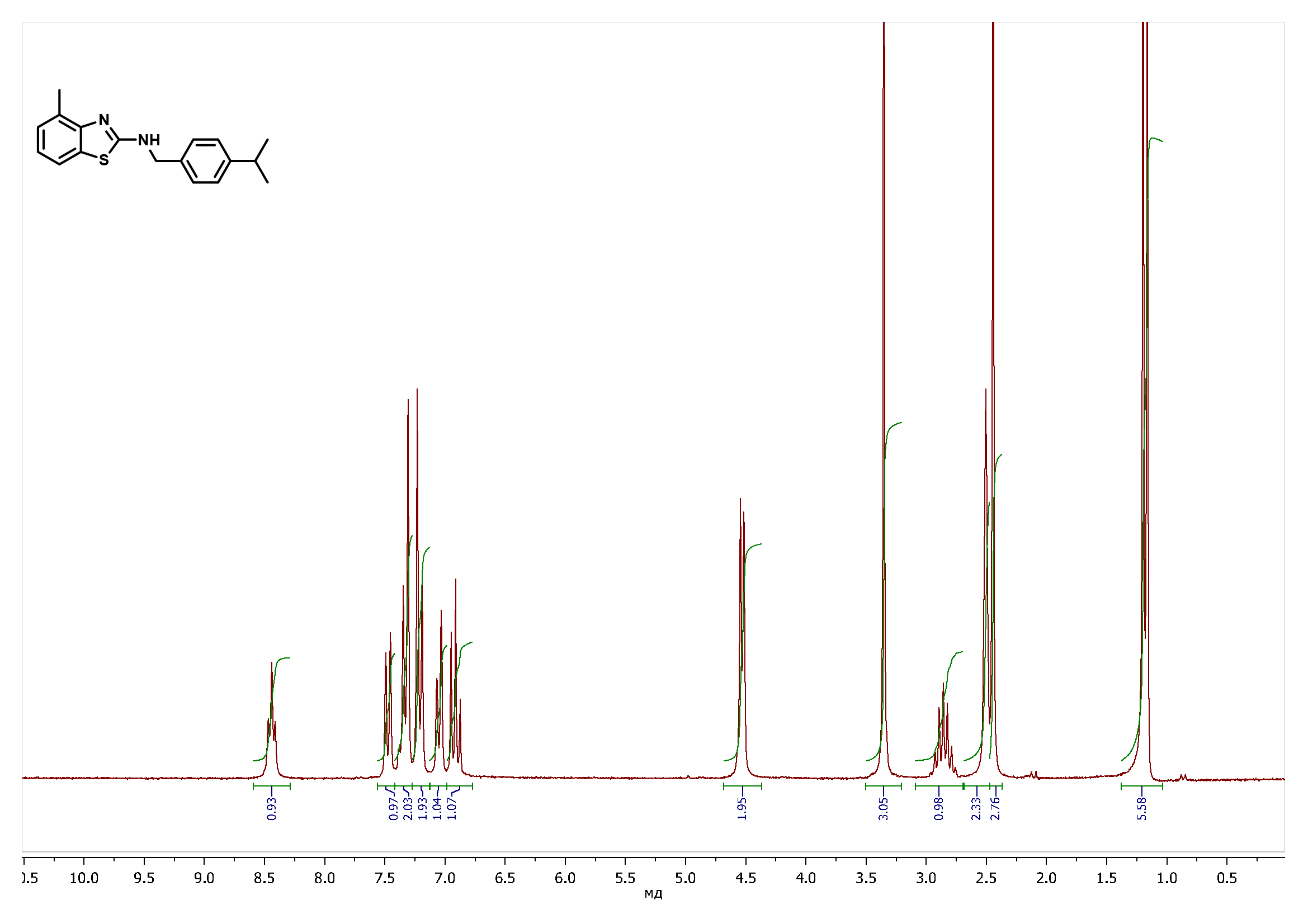
**

^13^C NMR spectrum (50 MHz, DMSO-d_6_) of compound BT-19

**
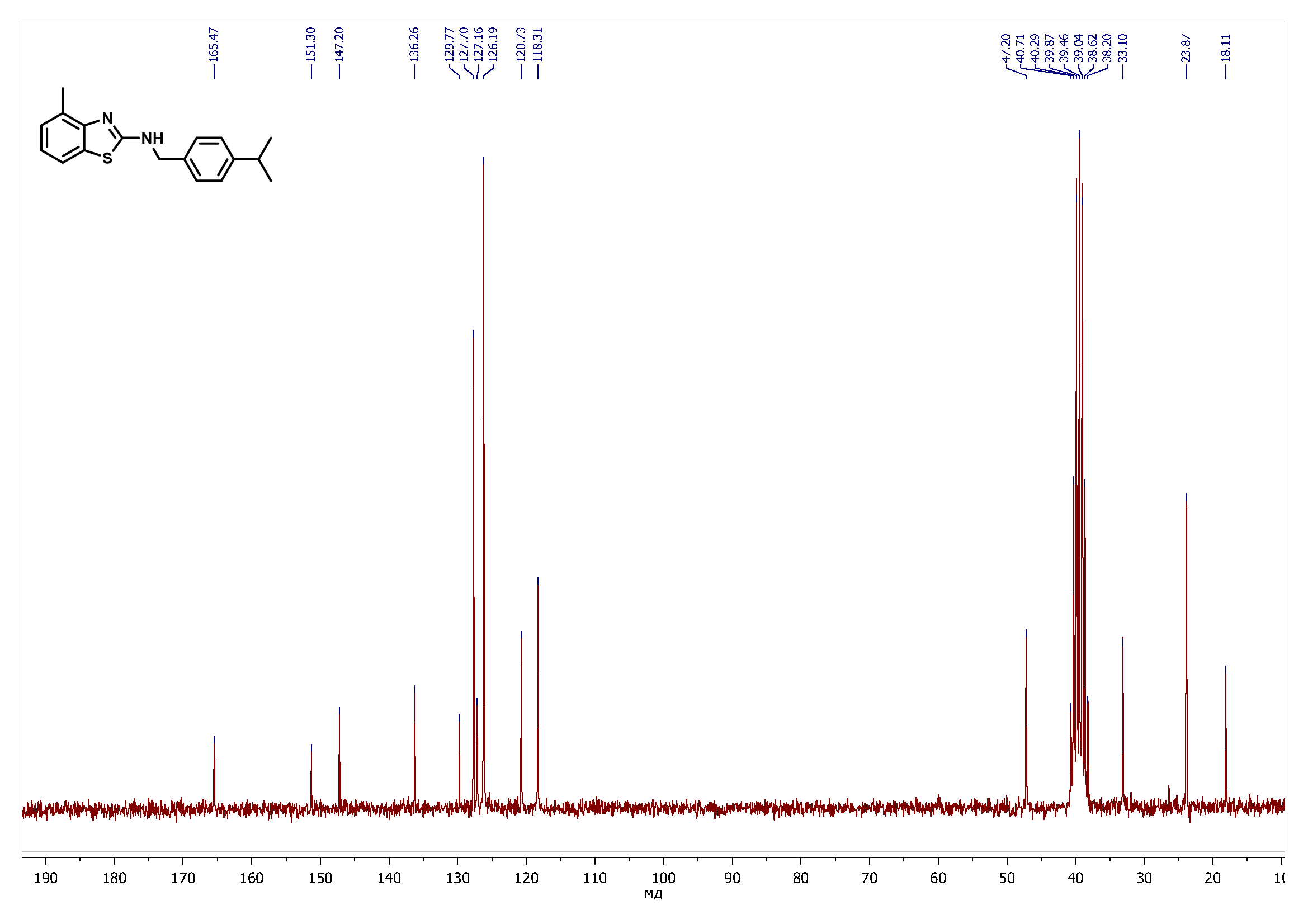
**

^1^H NMR spectrum (200 MHz, DMSO-d_6_) of compound BT-20

**
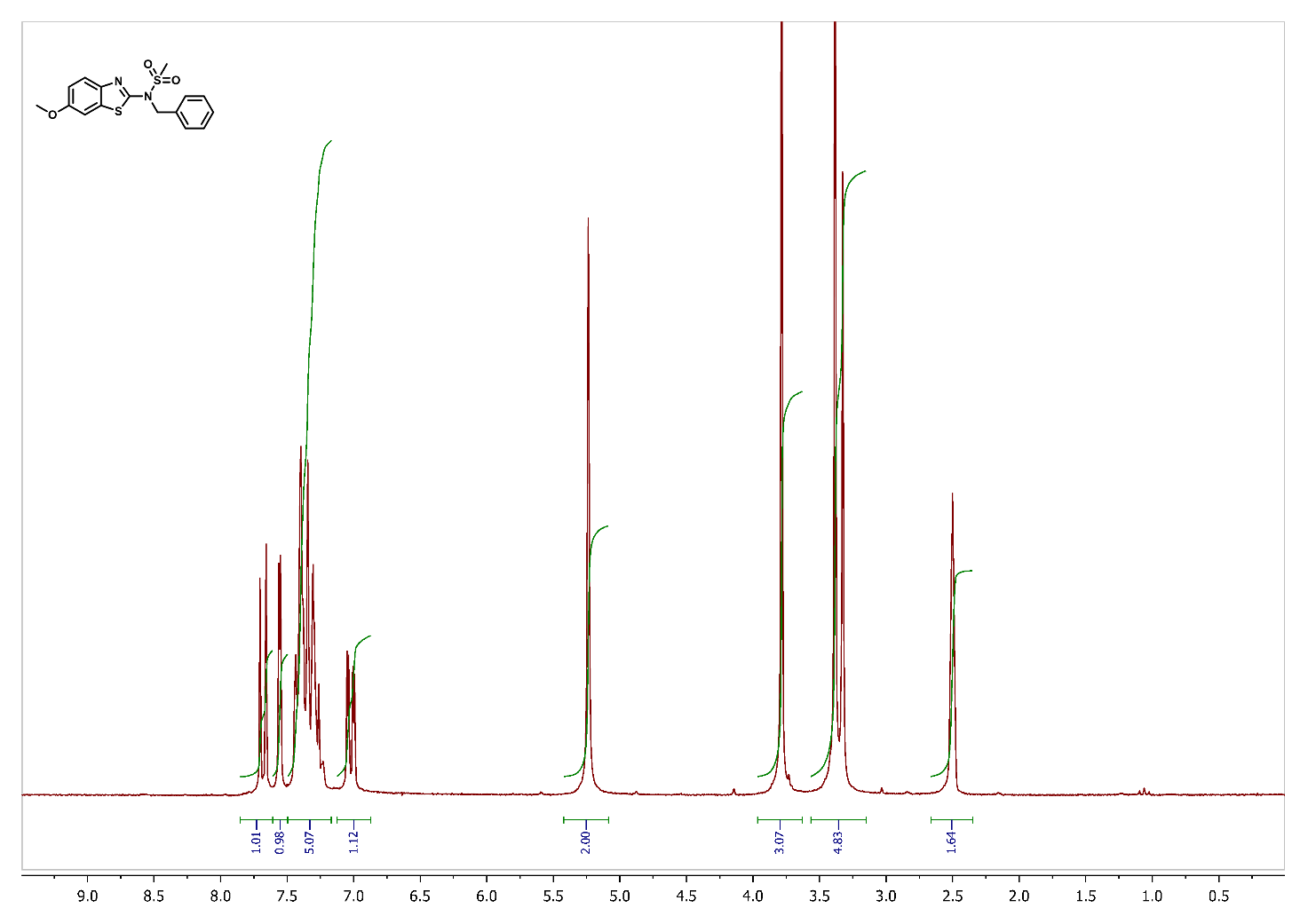
**

^13^C NMR spectrum (50 MHz, DMSO-d_6_) of compound BT-20

**
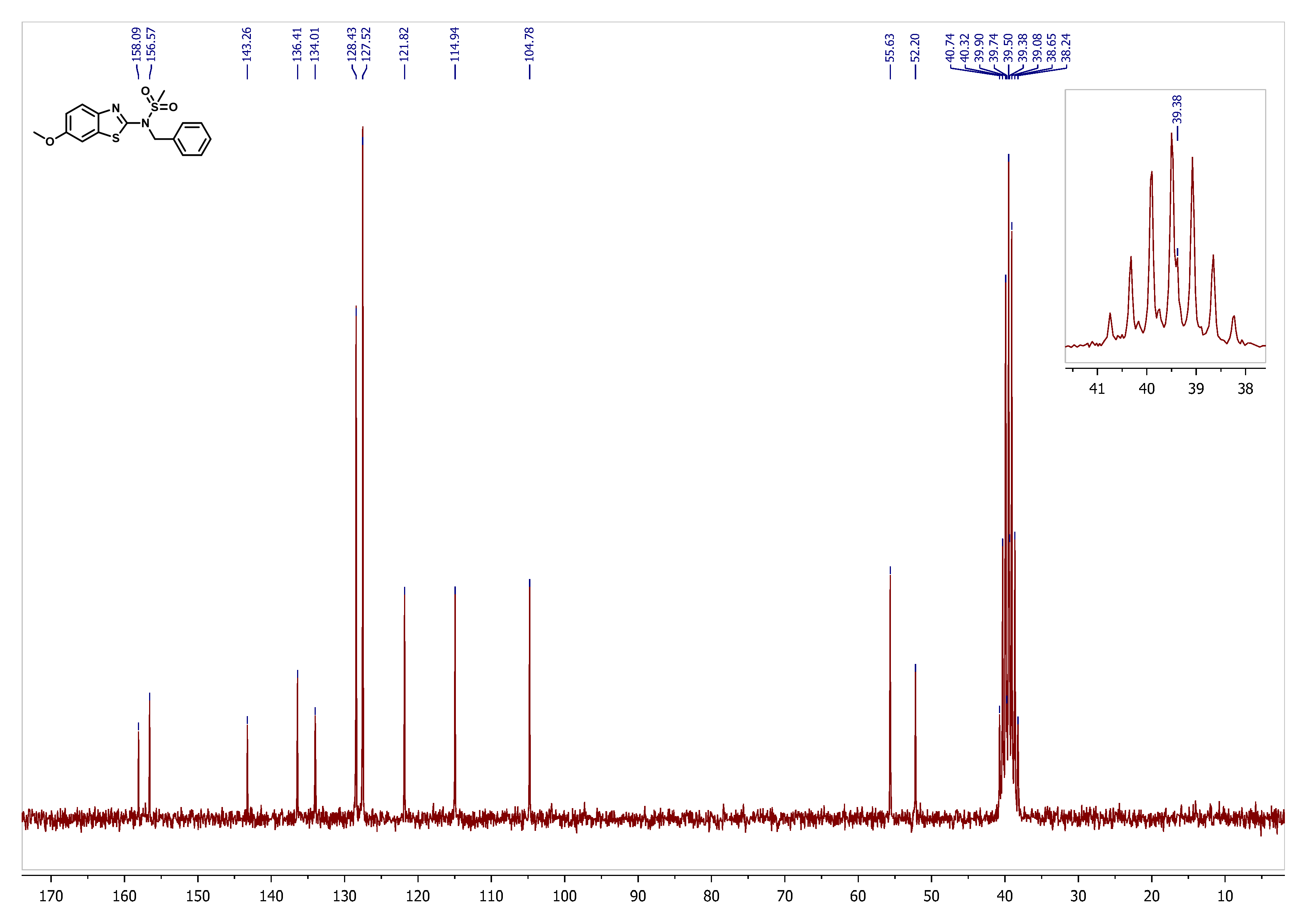
**

^1^H NMR spectrum (200 MHz, DMSO-d_6_) of compound BT-21

**
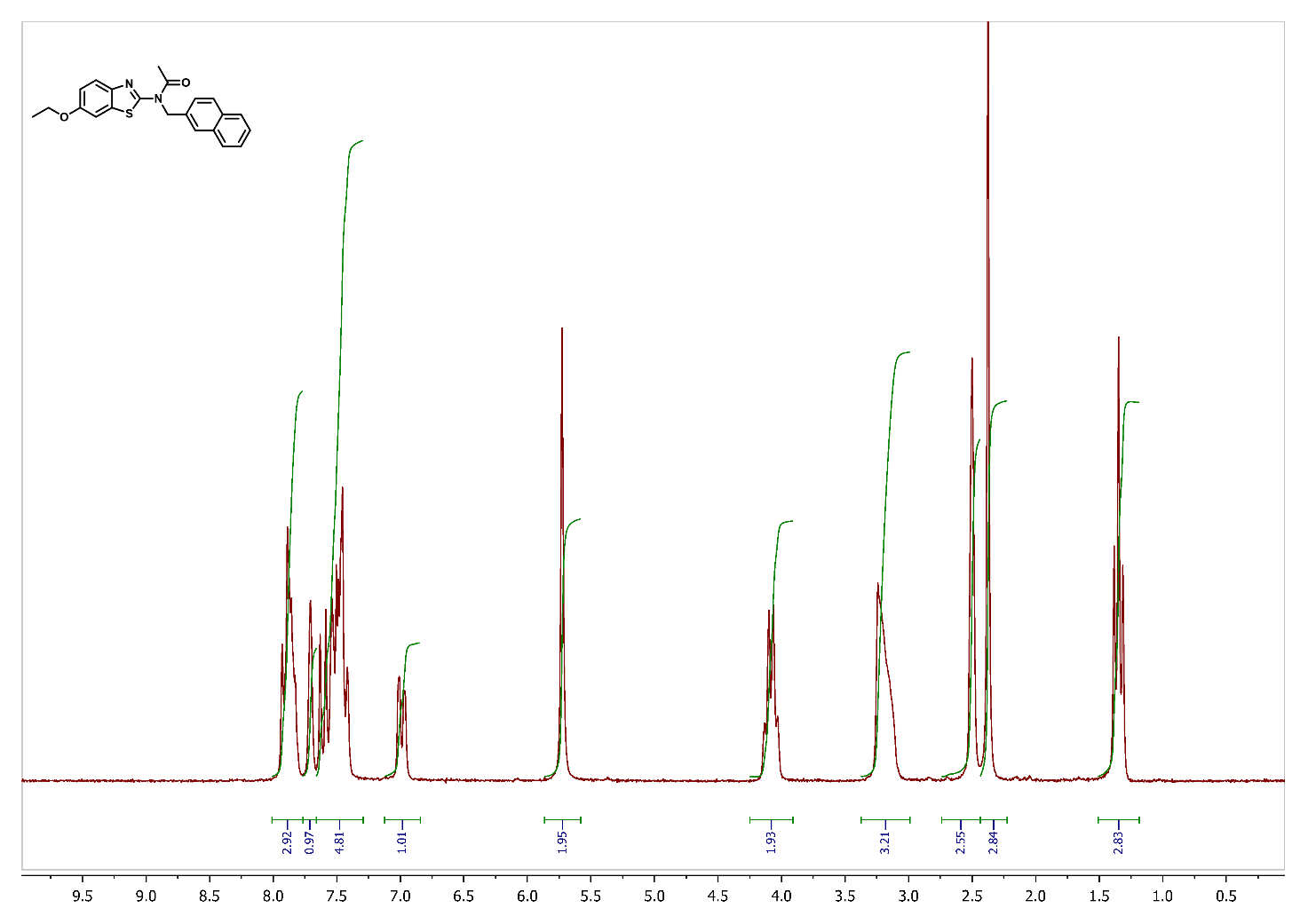
**

^13^C NMR spectrum (50 MHz, DMSO-d_6_) of compound BT-21

**
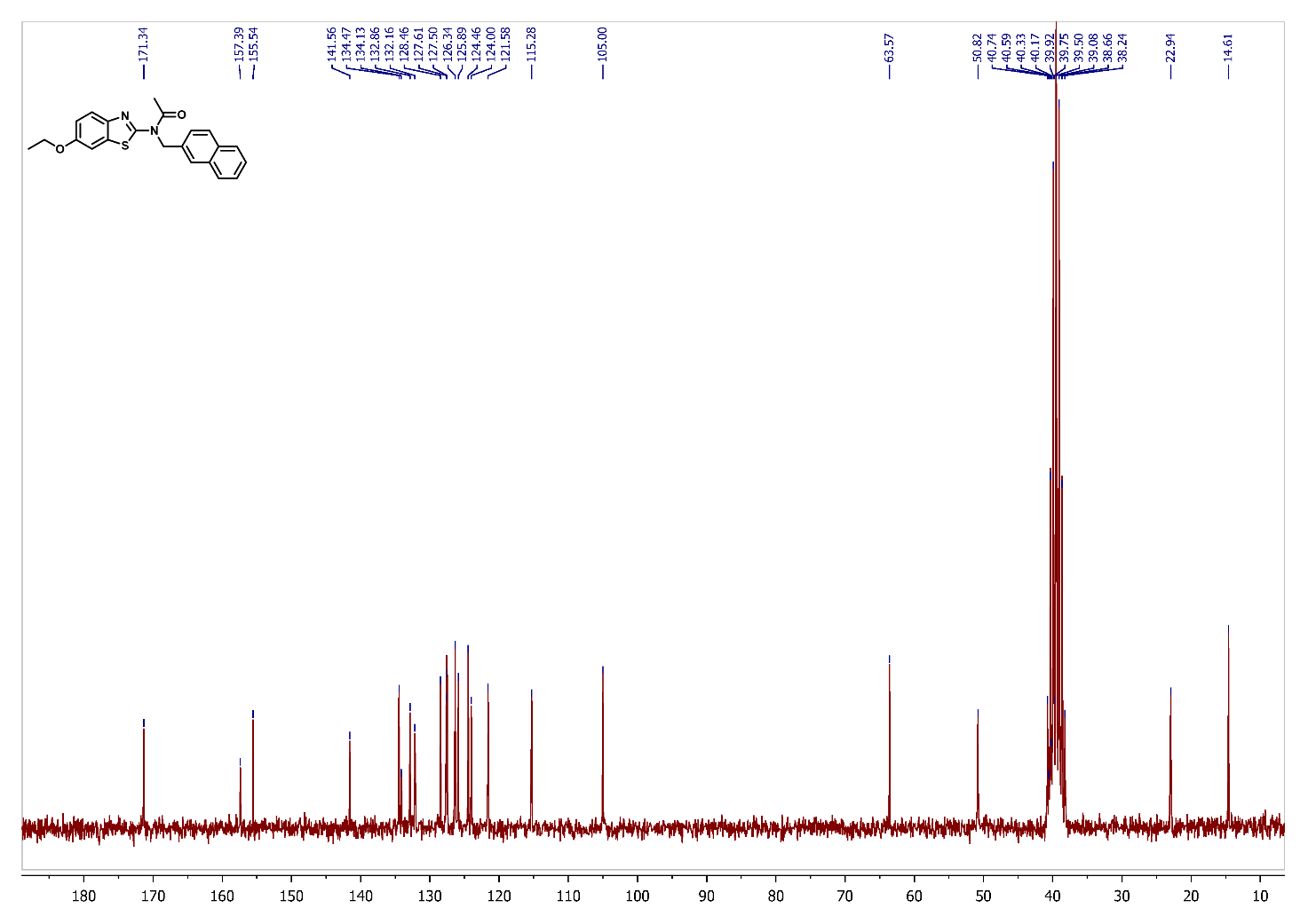
**

^1^H NMR spectrum (200 MHz, DMSO-d_6_) of compound BT-22

**
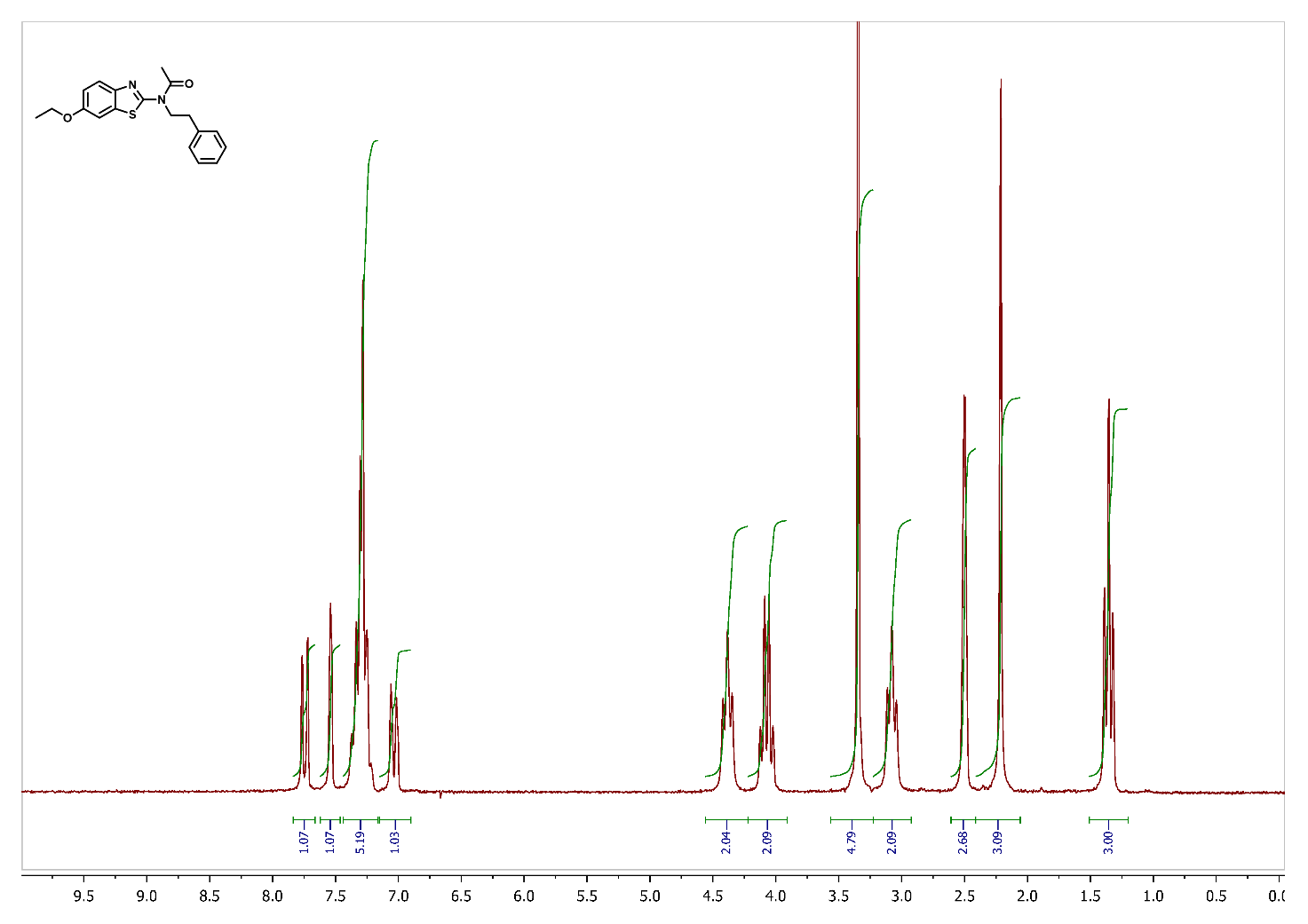
**

^13^C NMR spectrum (50 MHz, DMSO-d_6_) of compound BT-22

**
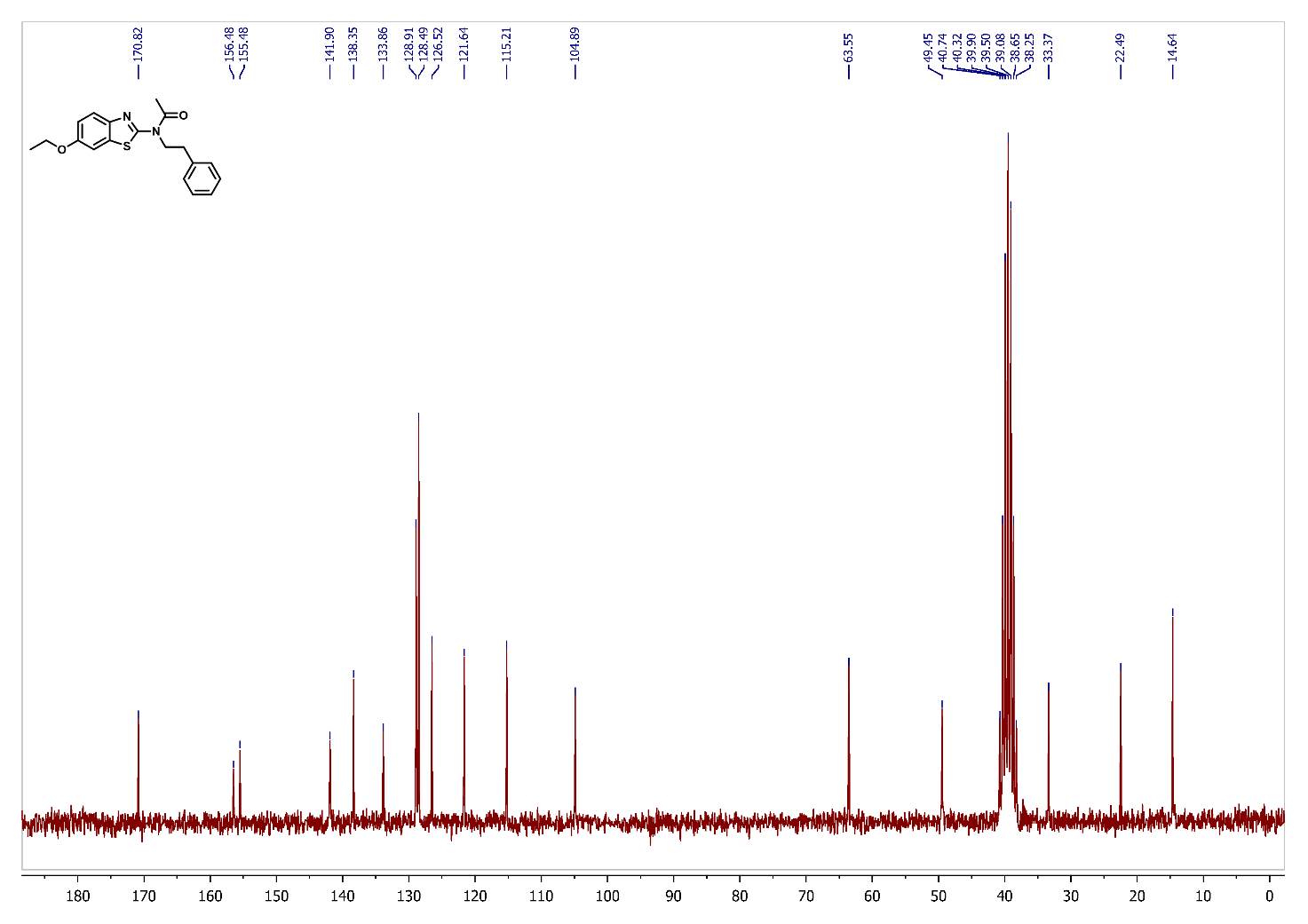
**

^1^H NMR spectrum (200 MHz, DMSO-d_6_) of compound BT-23

**
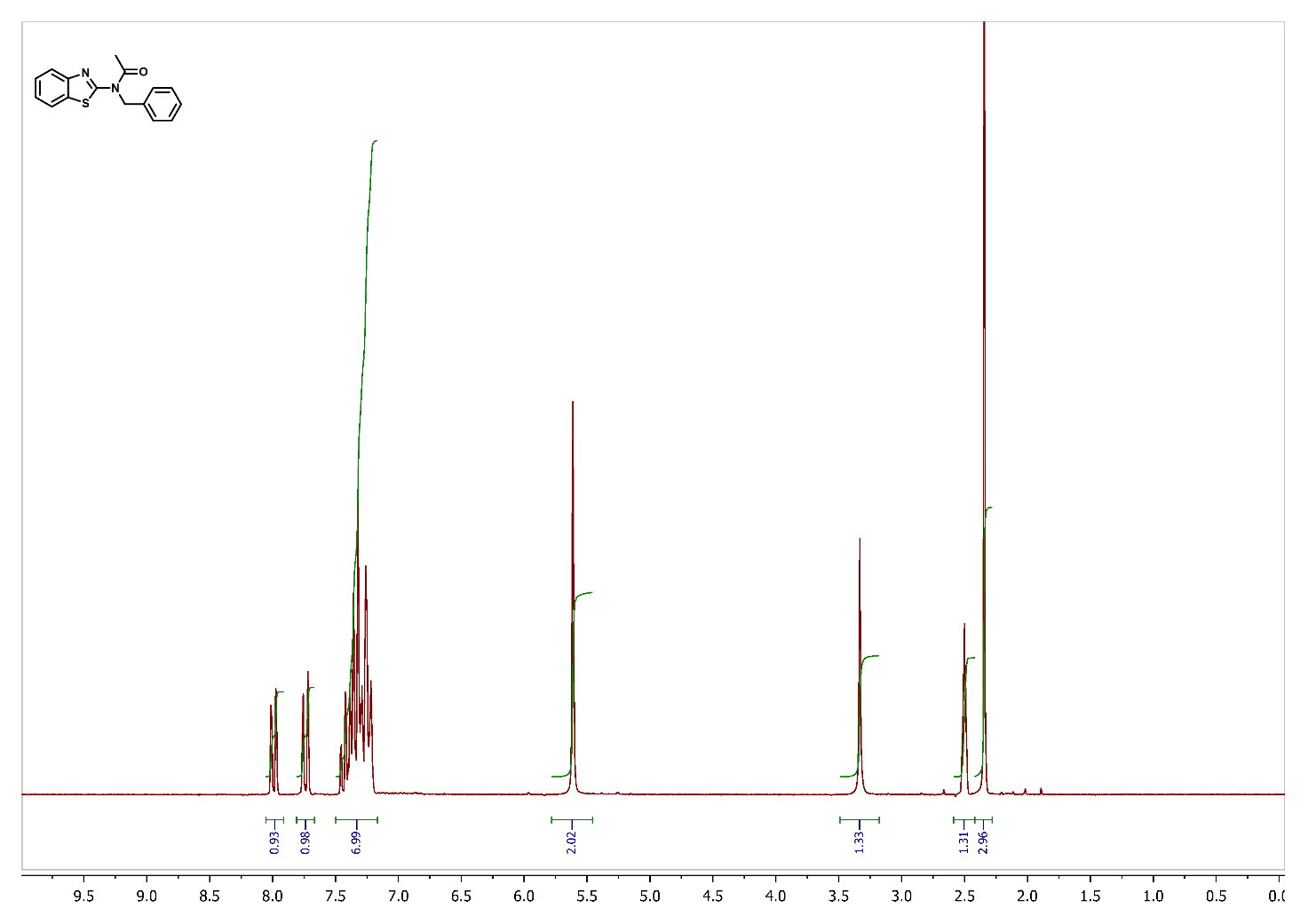
**

^13^C NMR spectrum (50 MHz, DMSO-d_6_) of compound BT-23

**
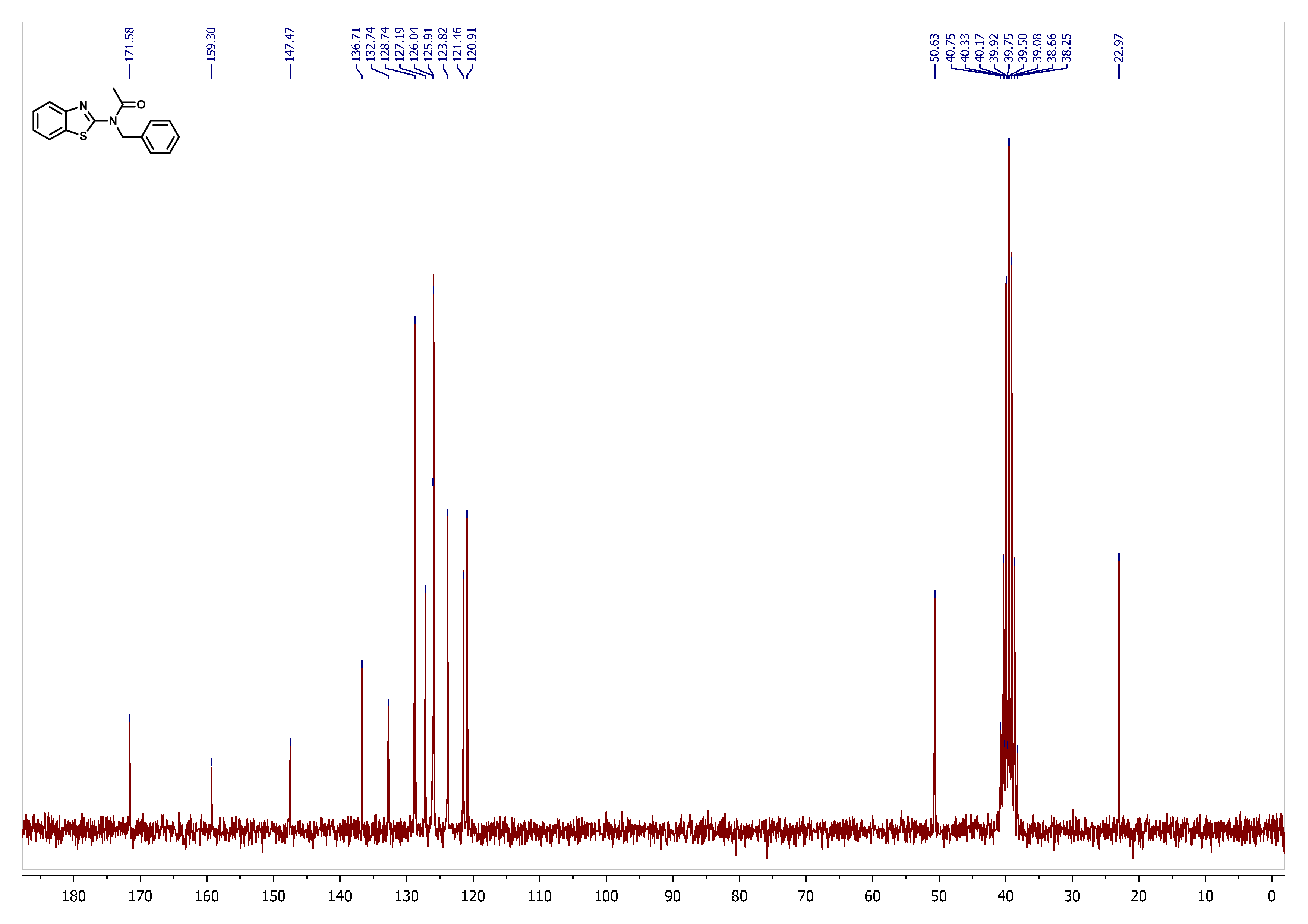
**

^1^H NMR spectrum (200 MHz, DMSO-d_6_) of compound BT-24

**
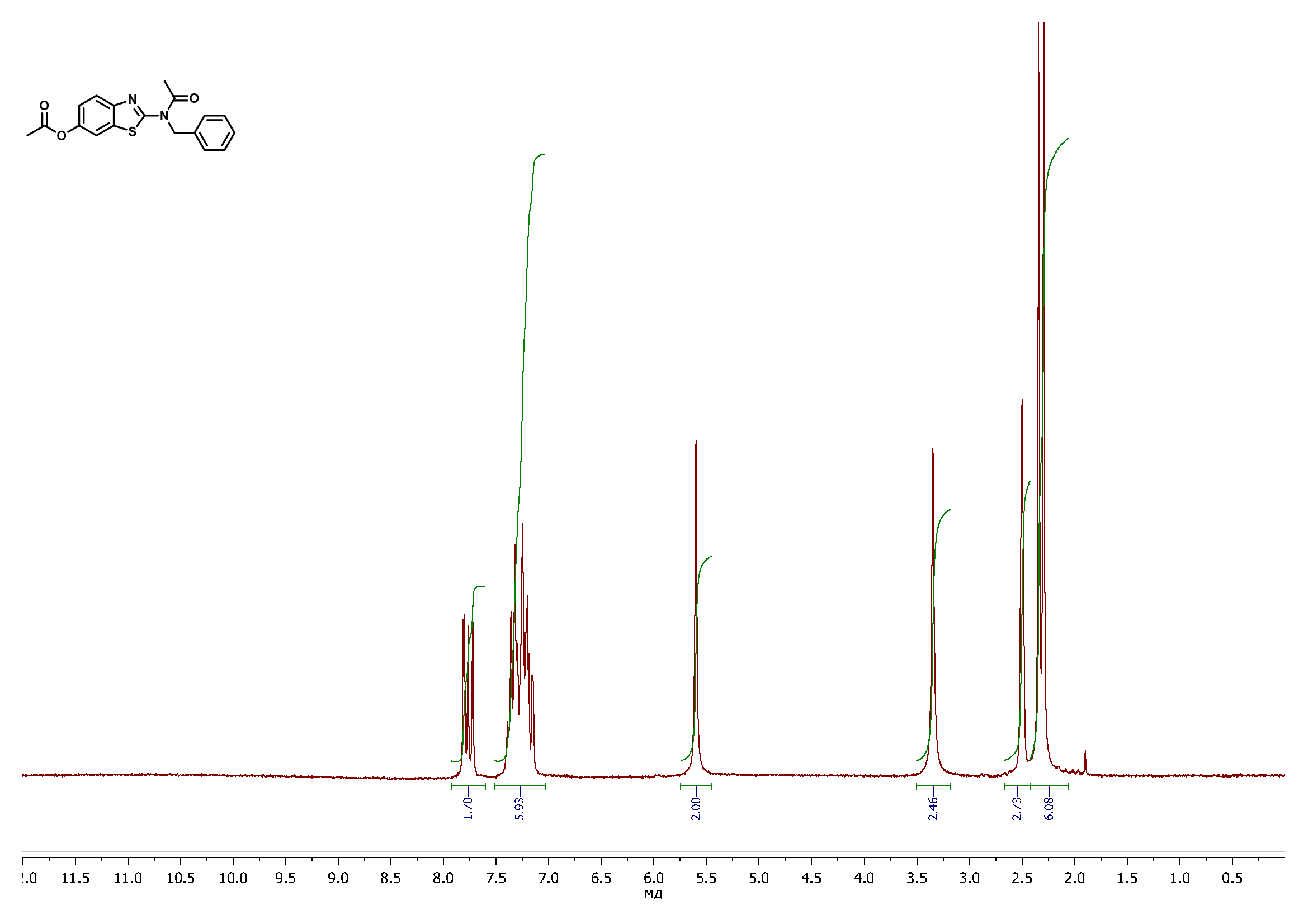
**

^13^C NMR spectrum (50 MHz, DMSO-d_6_) of compound BT-24

**
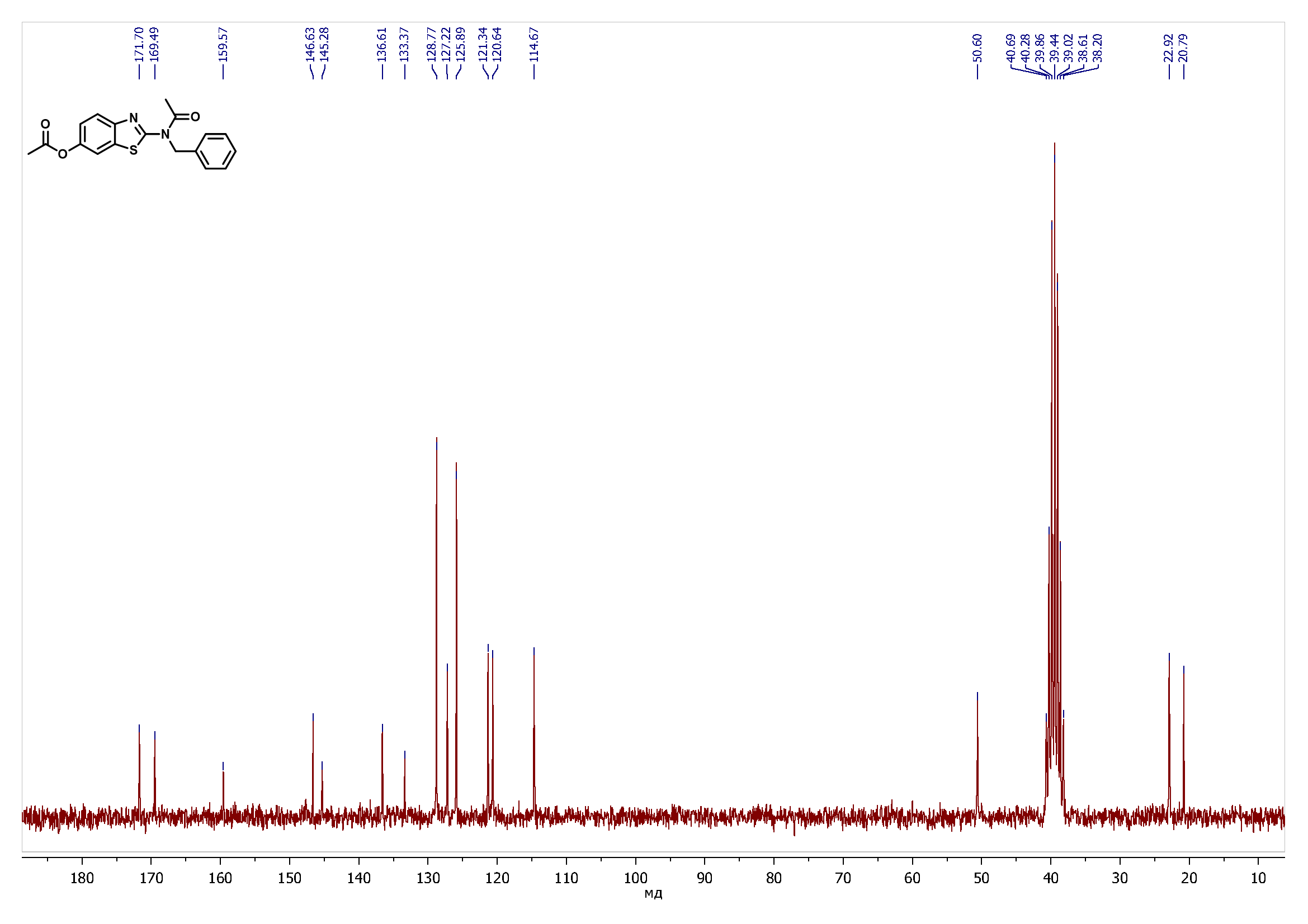
**

^1^H NMR spectrum (200 MHz, DMSO-d_6_) of compound BT-25

**
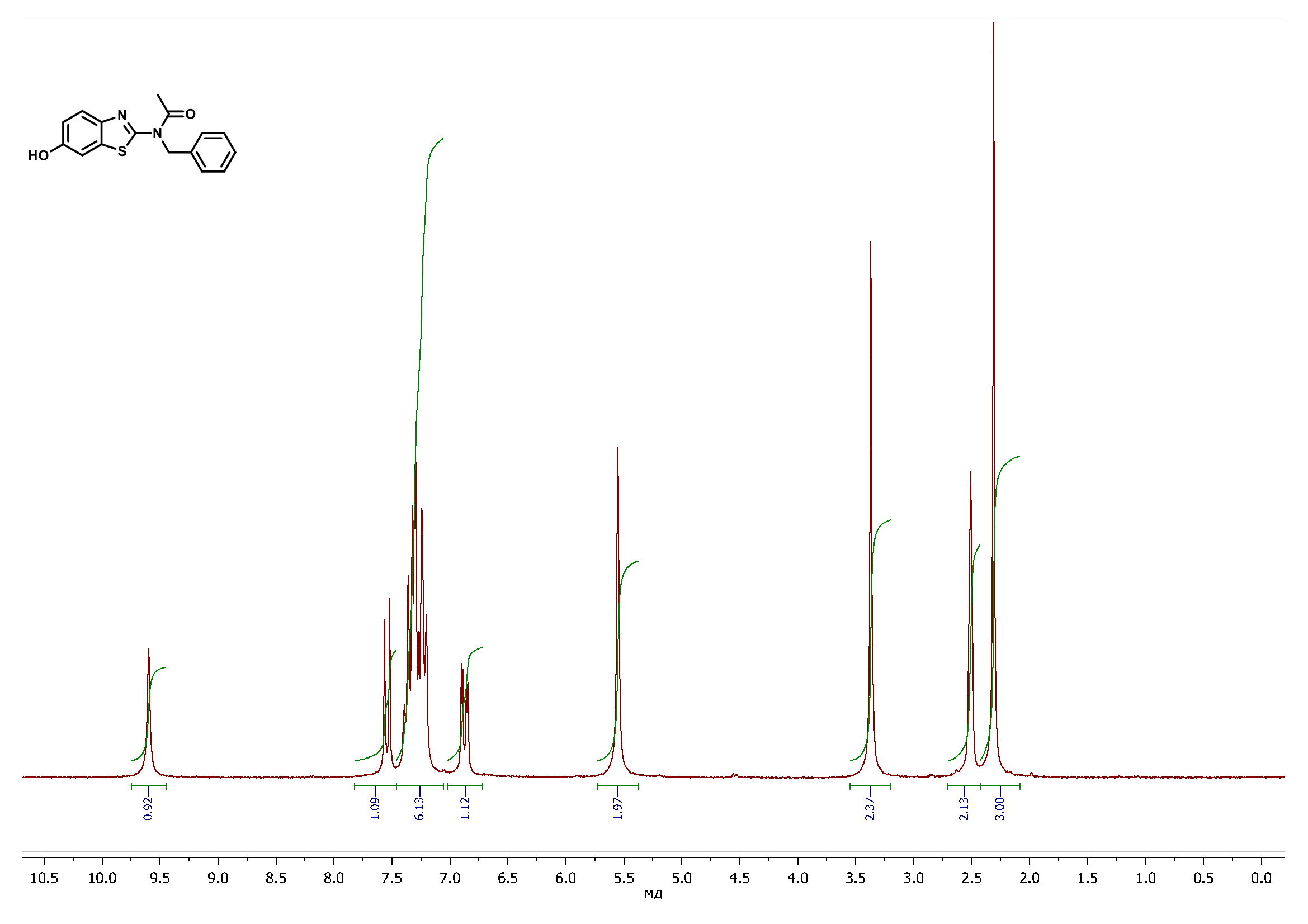
**

^13^C NMR spectrum (50 MHz, DMSO-d_6_) of compound BT-25

**
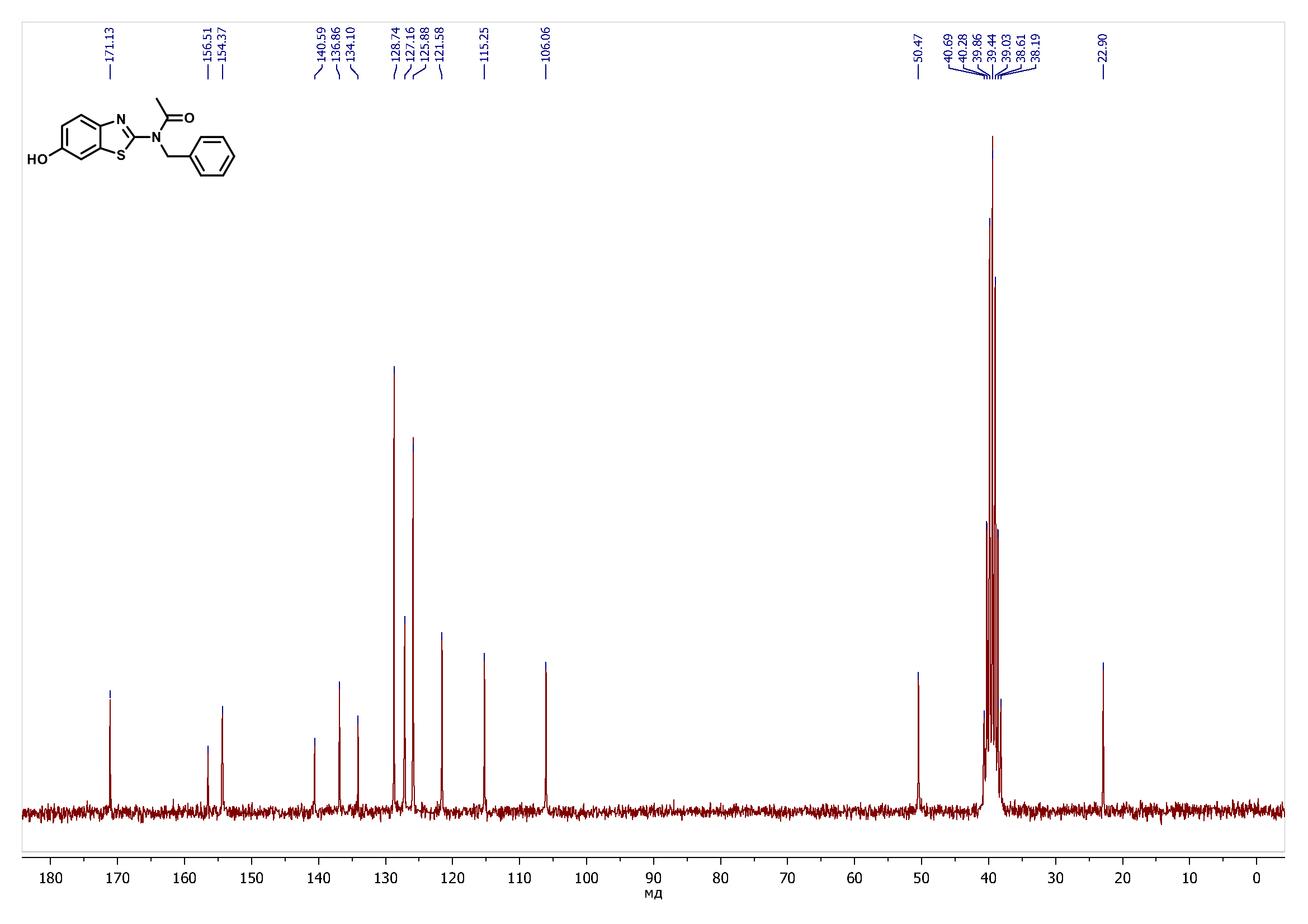
**

^1^H NMR spectrum (200 MHz, DMSO-d_6_) of compound BT-26

**
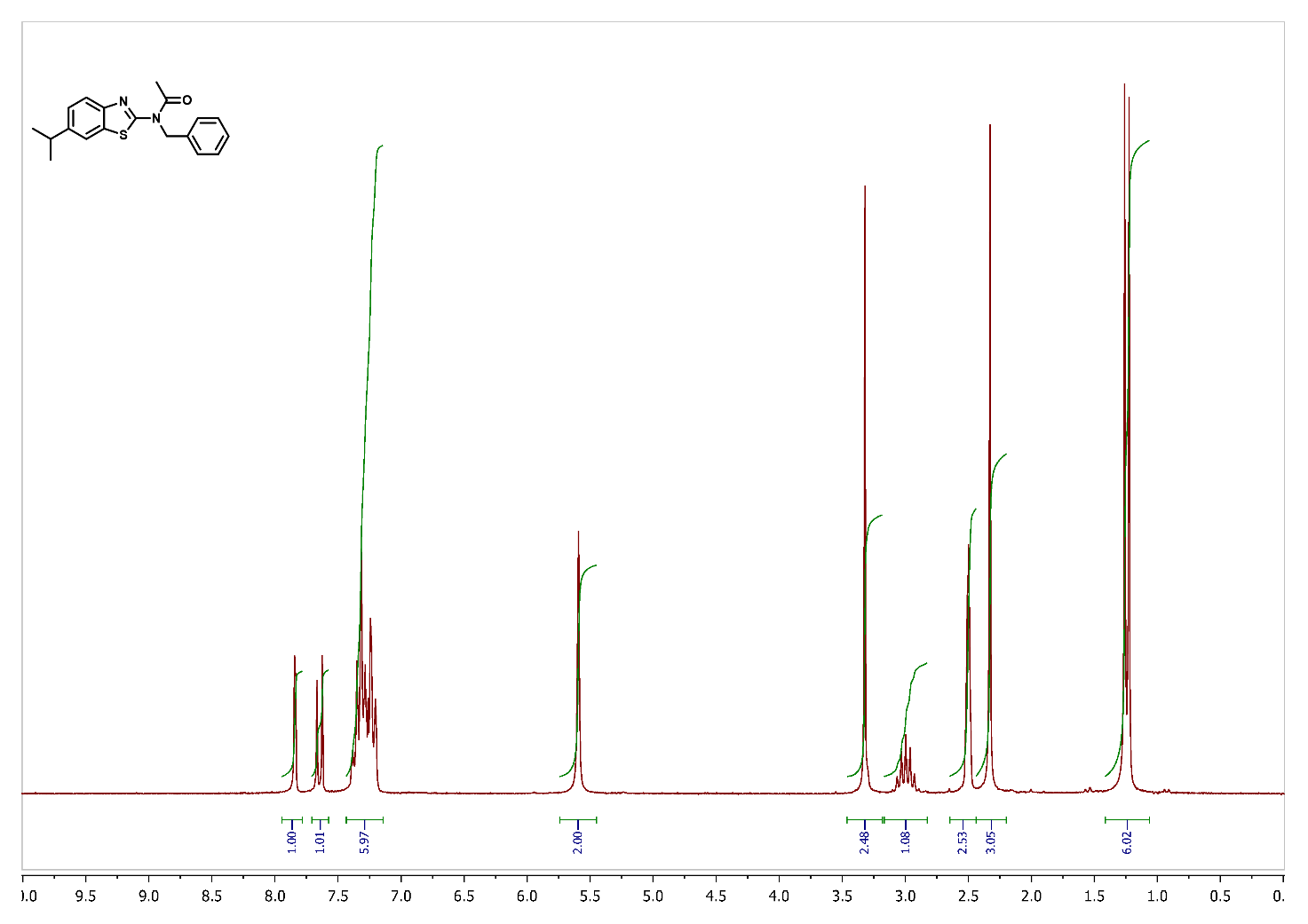
**

^13^C NMR spectrum (50 MHz, DMSO-d_6_) of compound BT-26

**
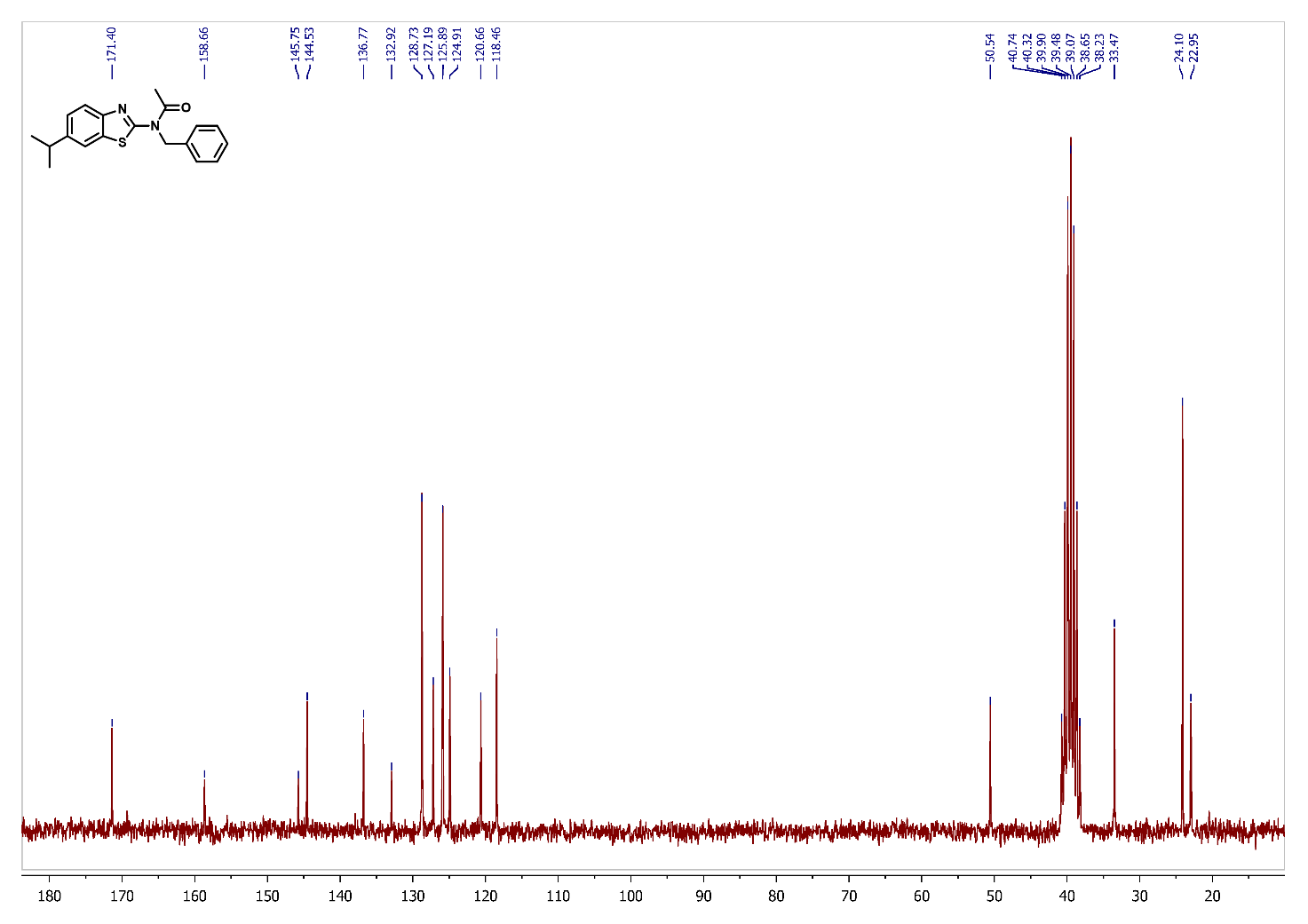
**

^1^H NMR spectrum (200 MHz, DMSO-d_6_) of compound BT-27

**
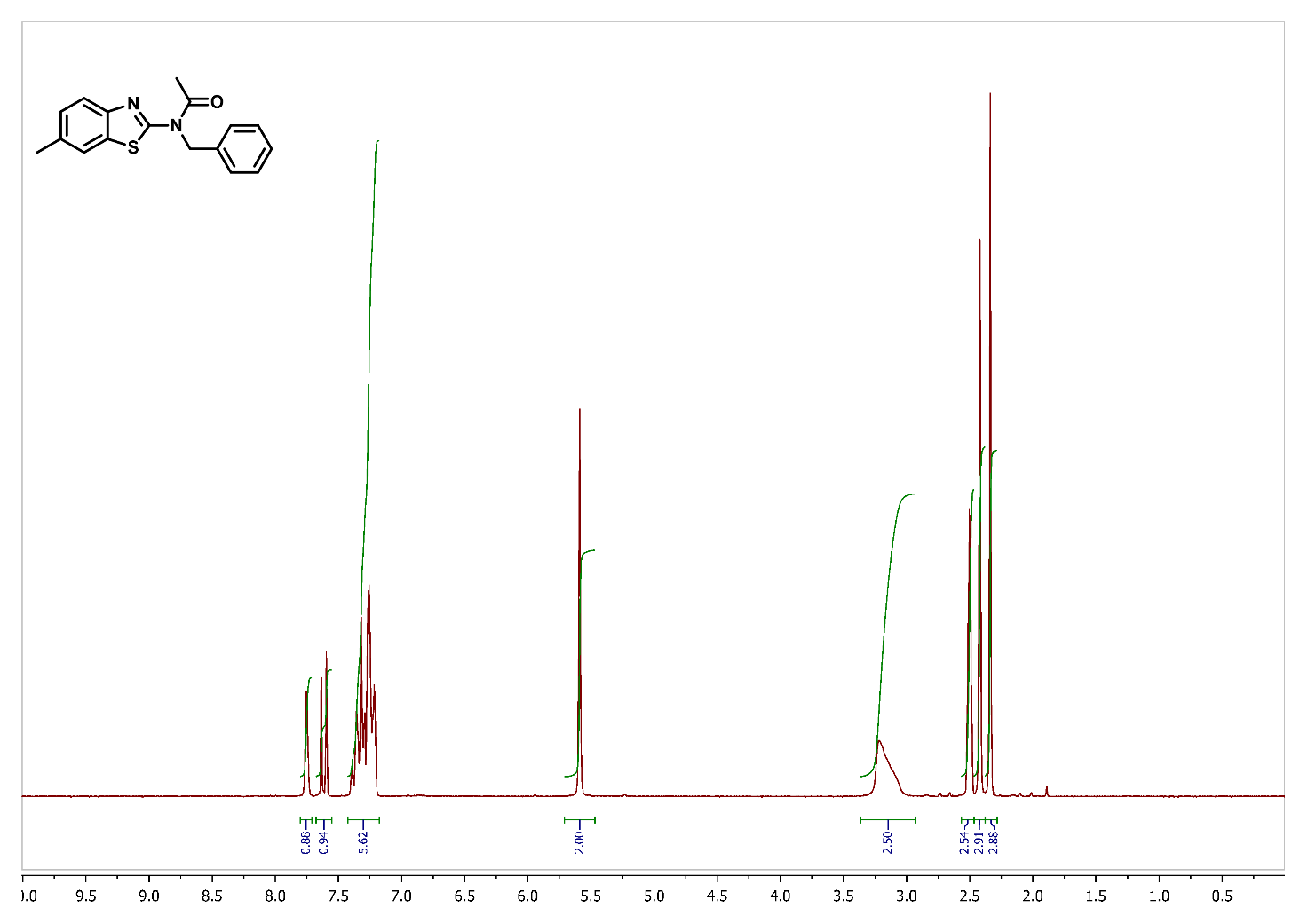
**

^13^C NMR spectrum (50 MHz, DMSO-d_6_) of compound BT-27

**
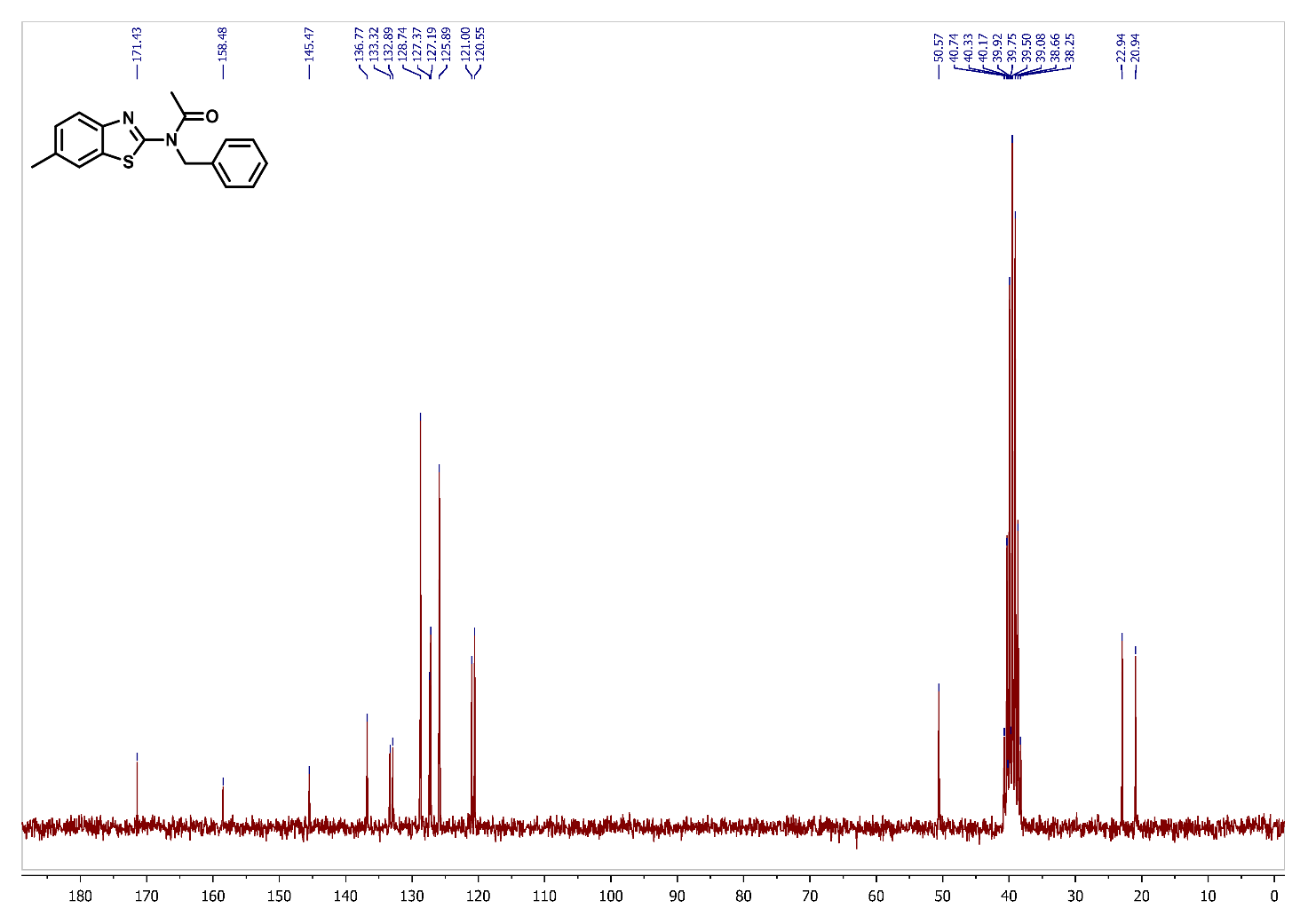
**

^1^H NMR spectrum (200 MHz, DMSO-d_6_) of compound BT-28

**
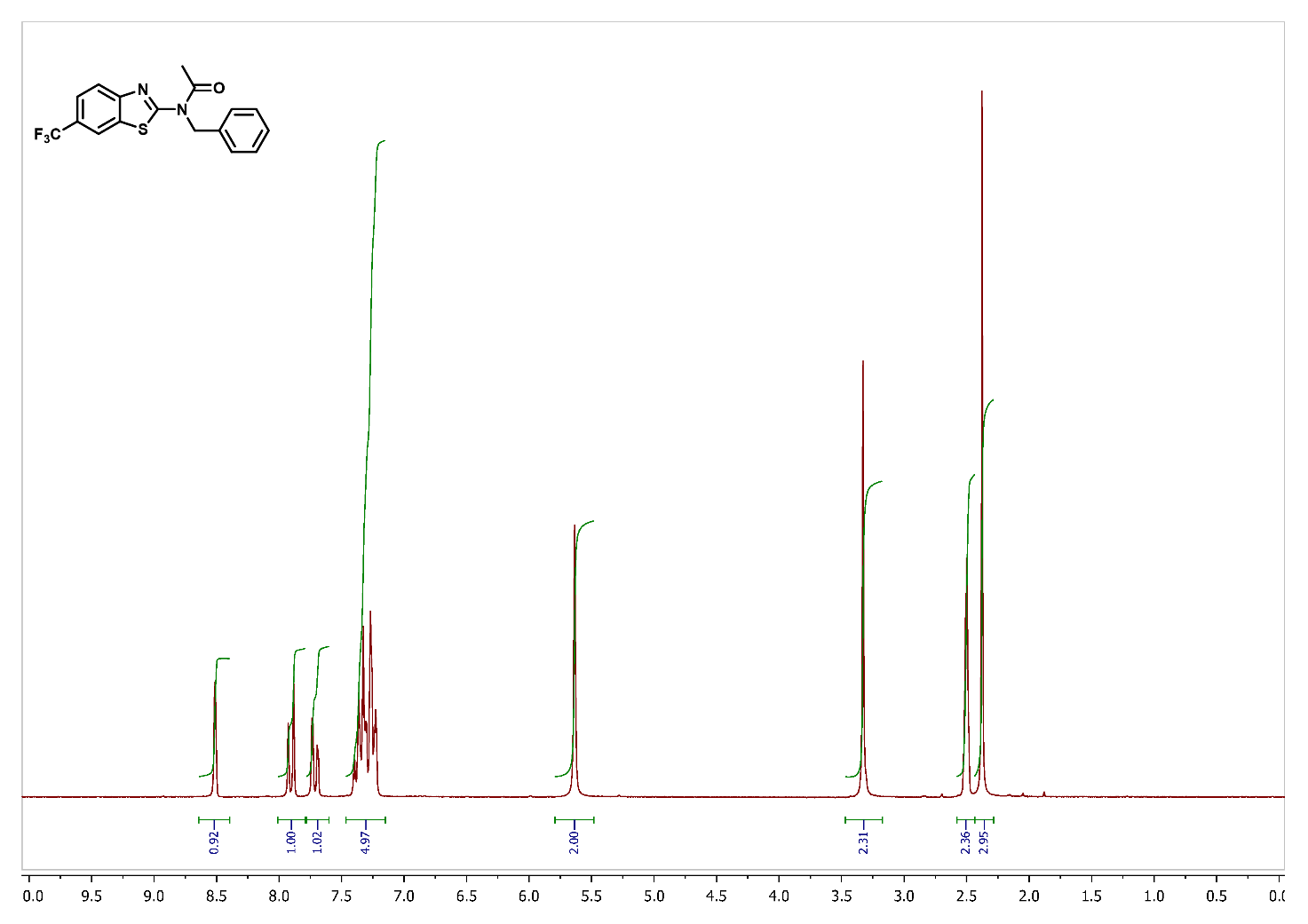
**

^13^C NMR spectrum (50 MHz, DMSO-d_6_) of compound BT-28

**
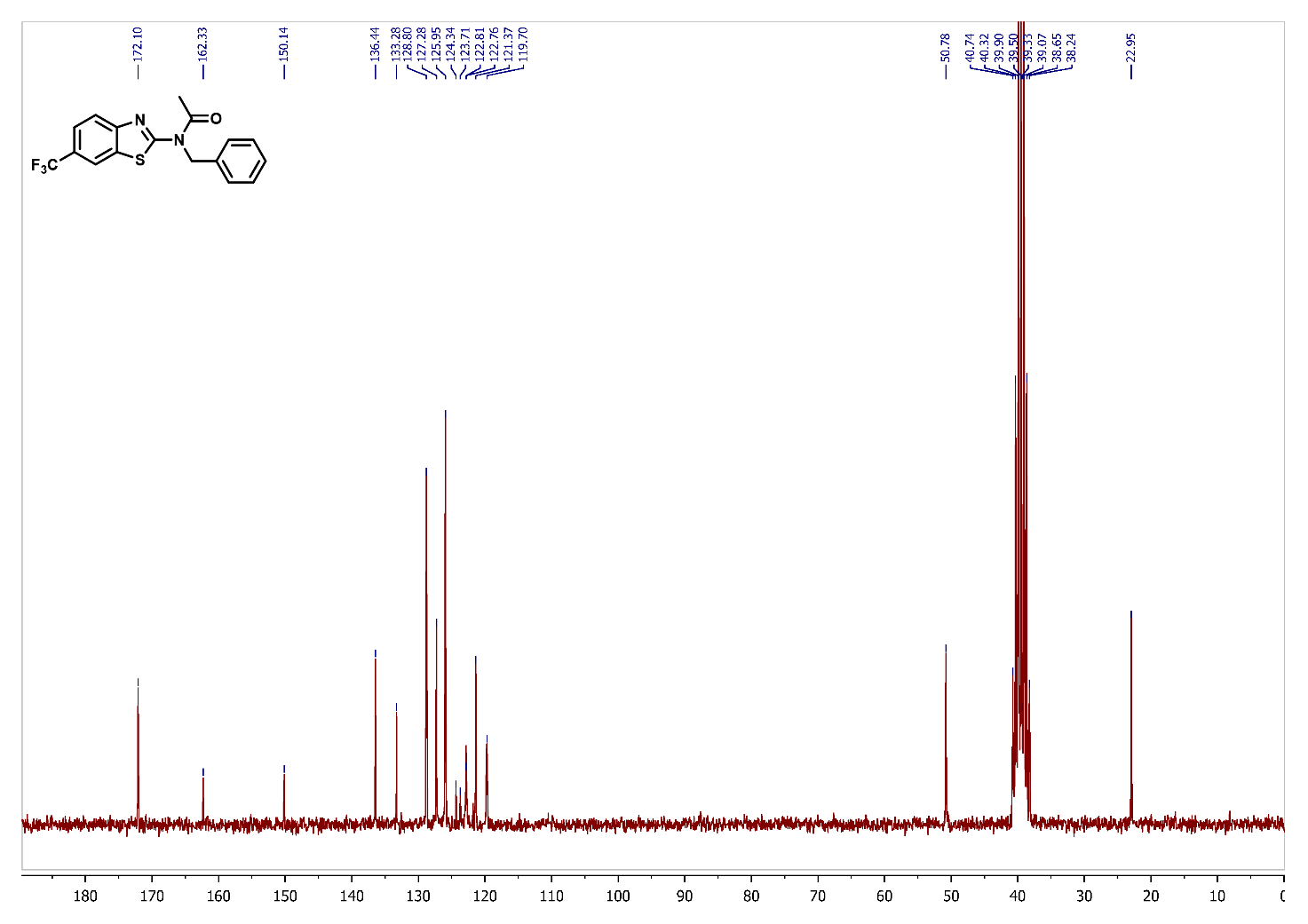
**

^1^H NMR spectrum (200 MHz, DMSO-d_6_) of compound BT-29

**
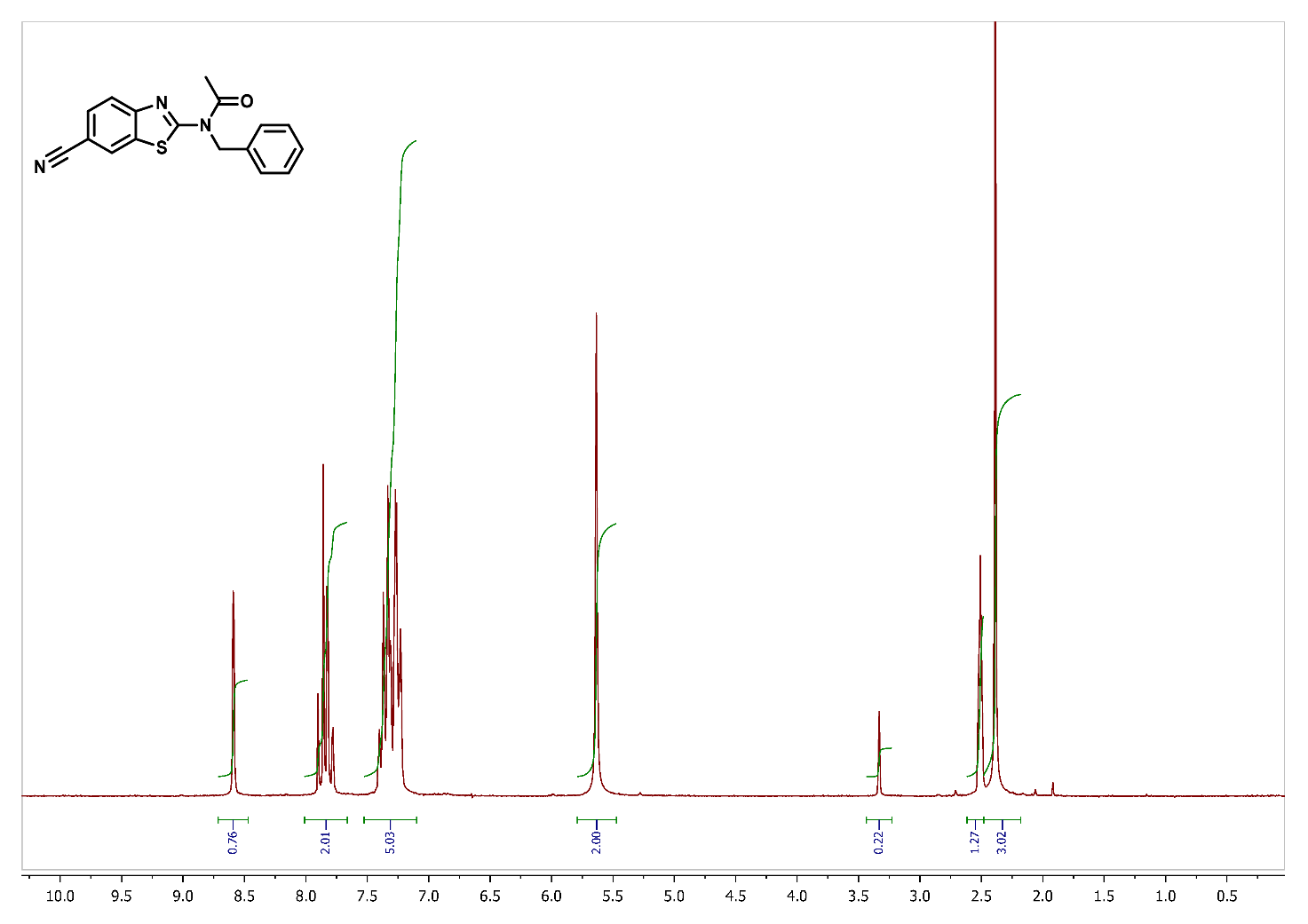
**

^13^C NMR spectrum (50 MHz, DMSO-d_6_) of compound BT-29

**
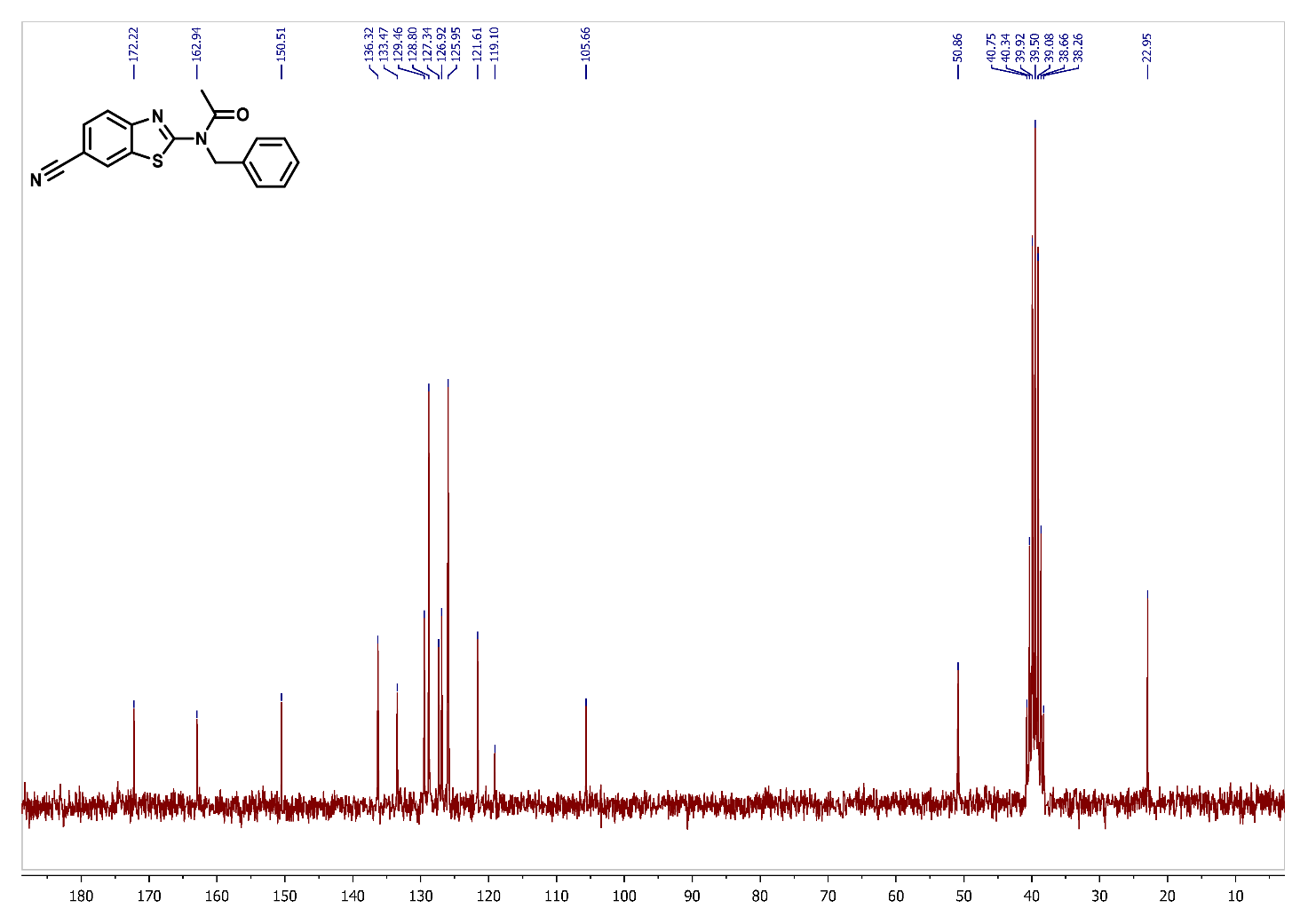
**

^1^H NMR spectrum (200 MHz, DMSO-d_6_) of compound BT-30

**
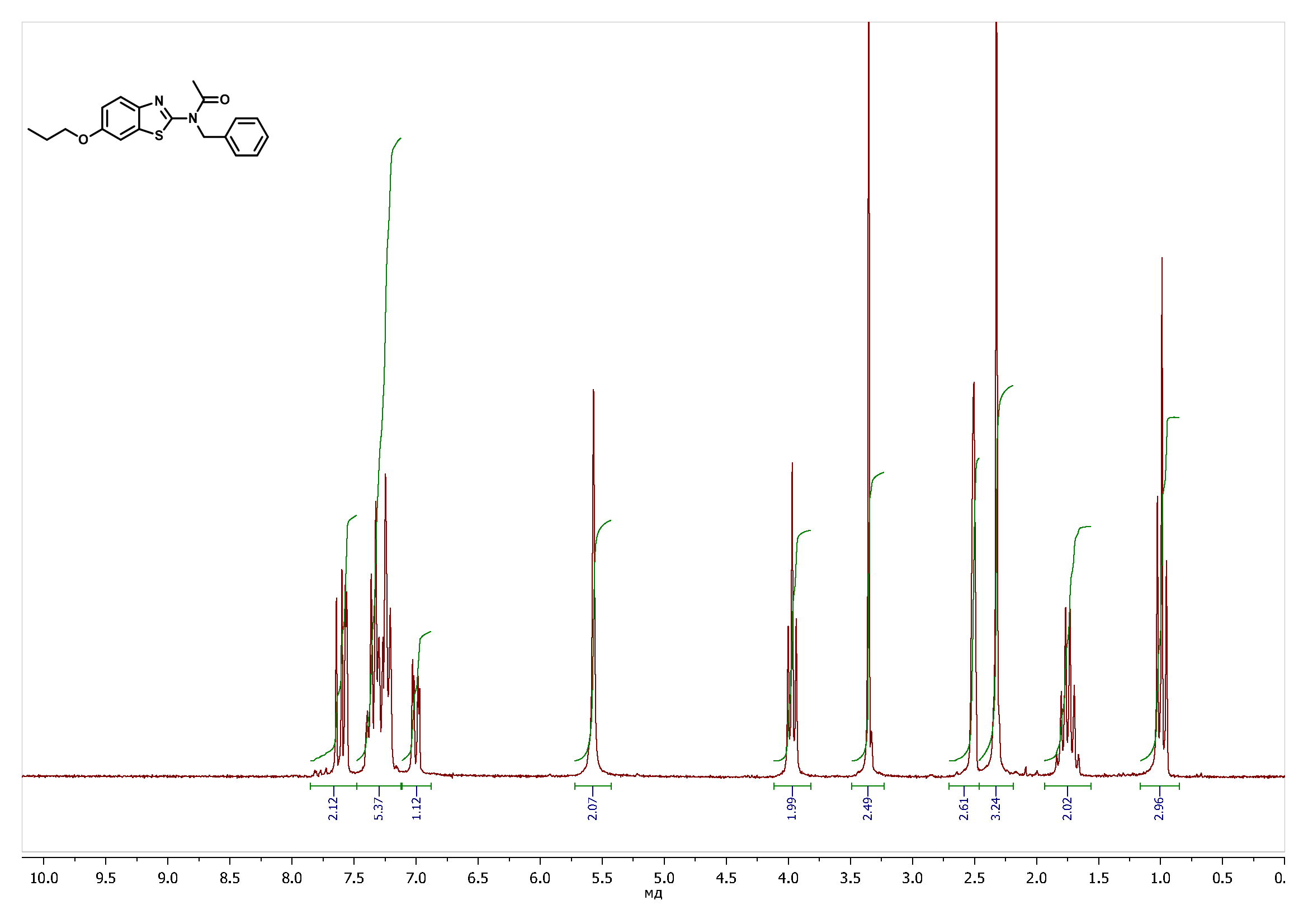
**

^13^C NMR spectrum (50 MHz, DMSO-d_6_) of compound BT-30

**
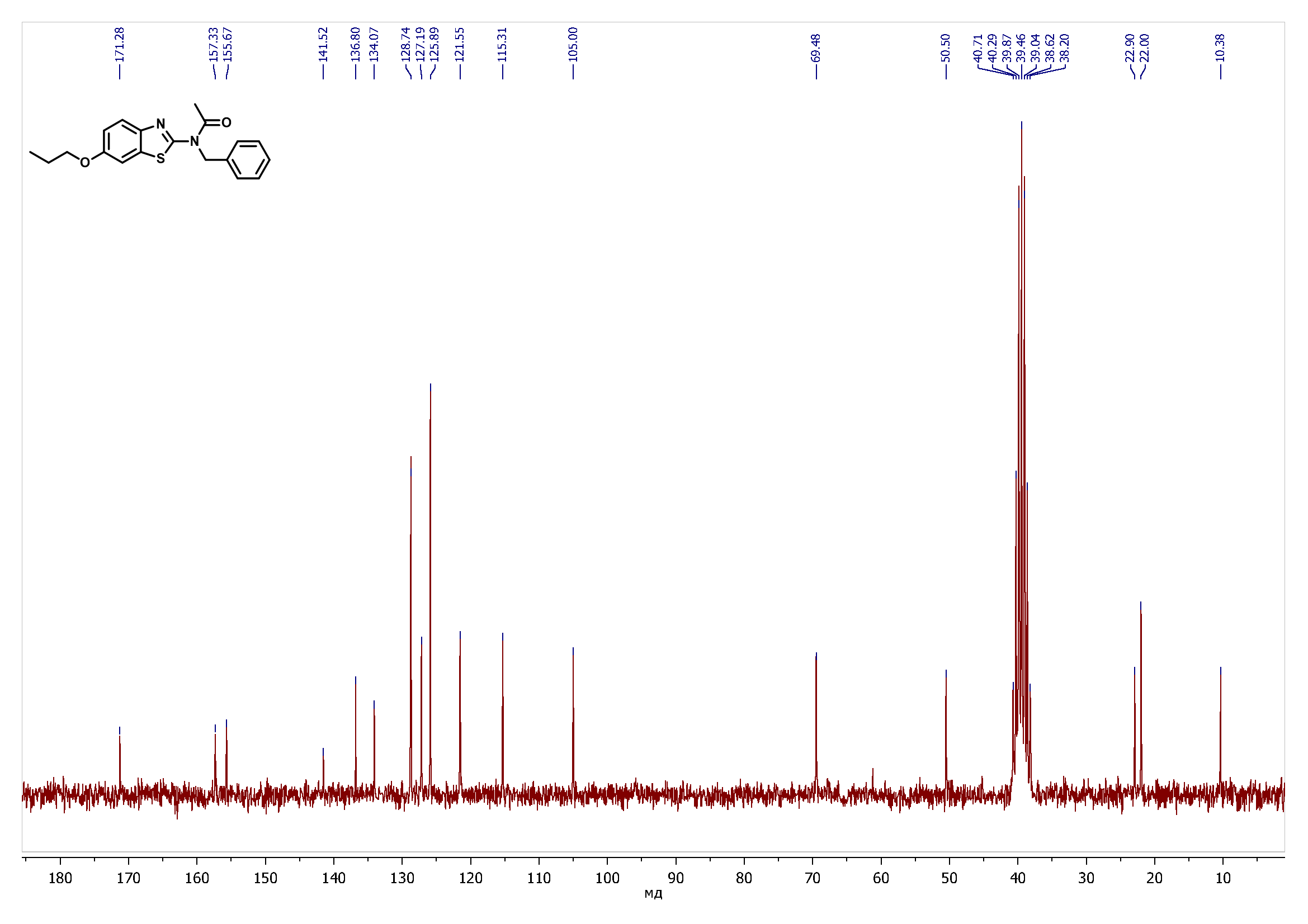
**

^1^H NMR spectrum (200 MHz, DMSO-d_6_) of compound BT-31

**
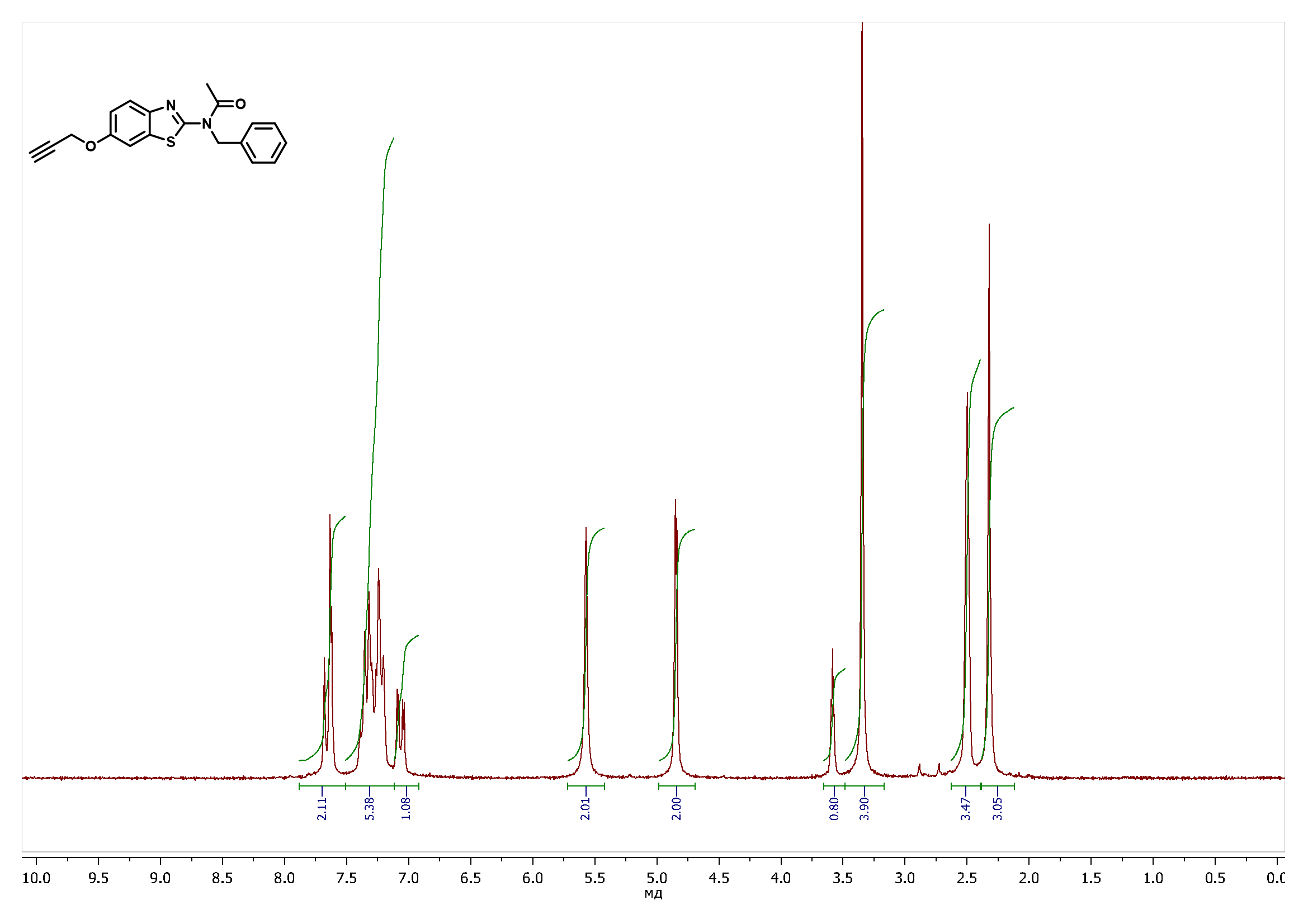
**

^13^C NMR spectrum (50 MHz, DMSO-d_6_) of compound BT-31

**
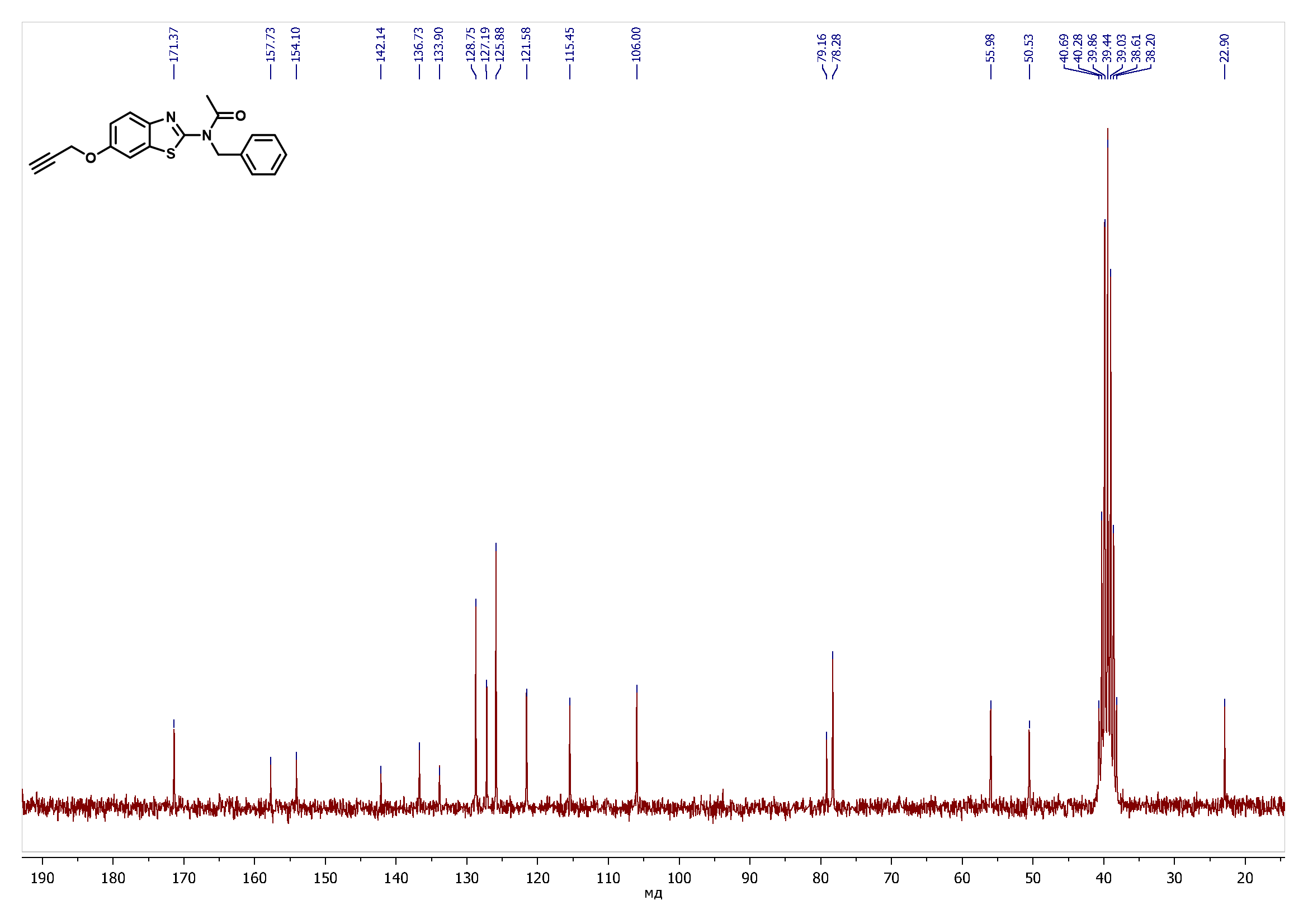
**

^1^H NMR spectrum (200 MHz, DMSO-d_6_) of compound BT-32

**
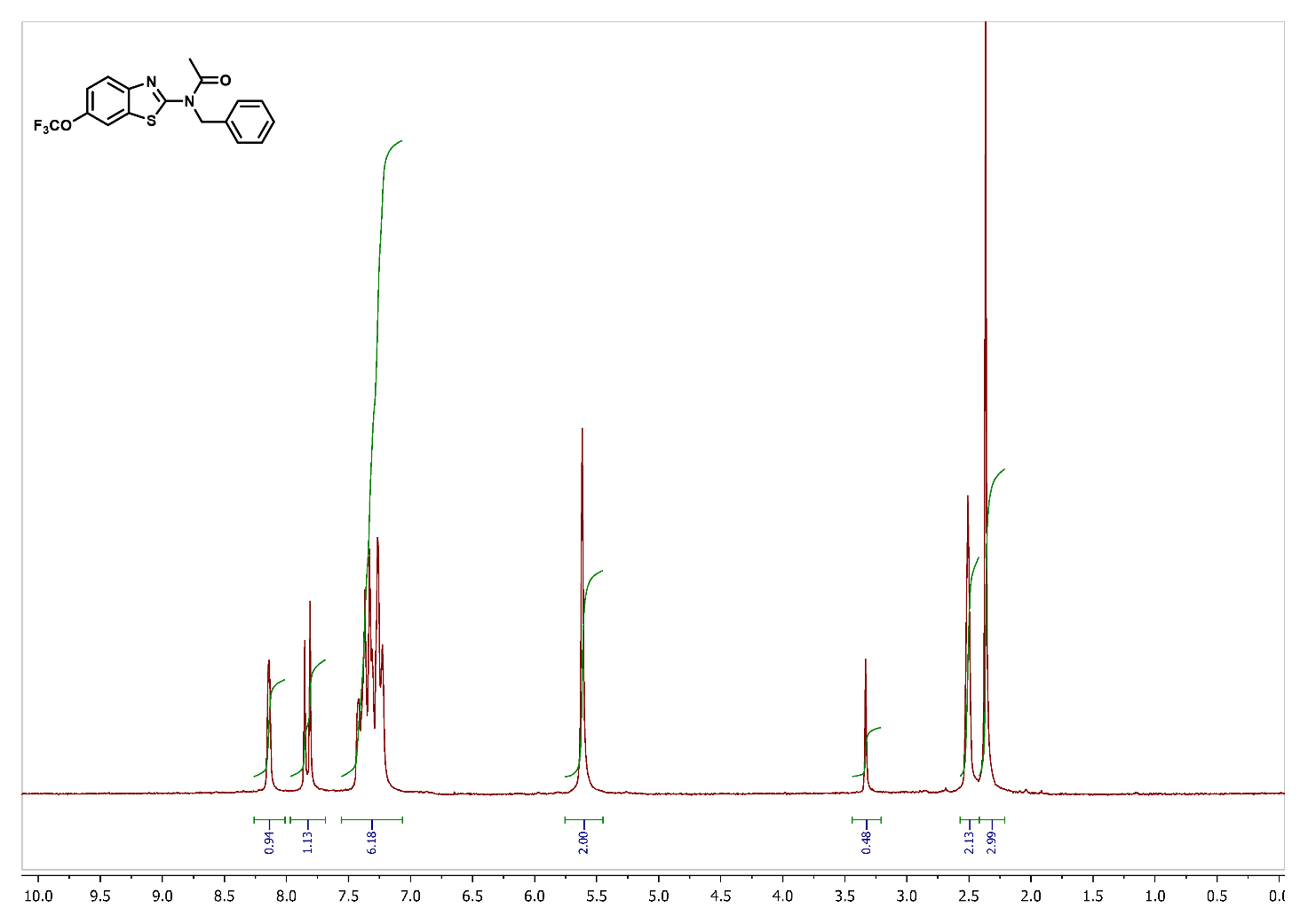
**

^13^C NMR spectrum (50 MHz, DMSO-d_6_) of compound BT-32

**
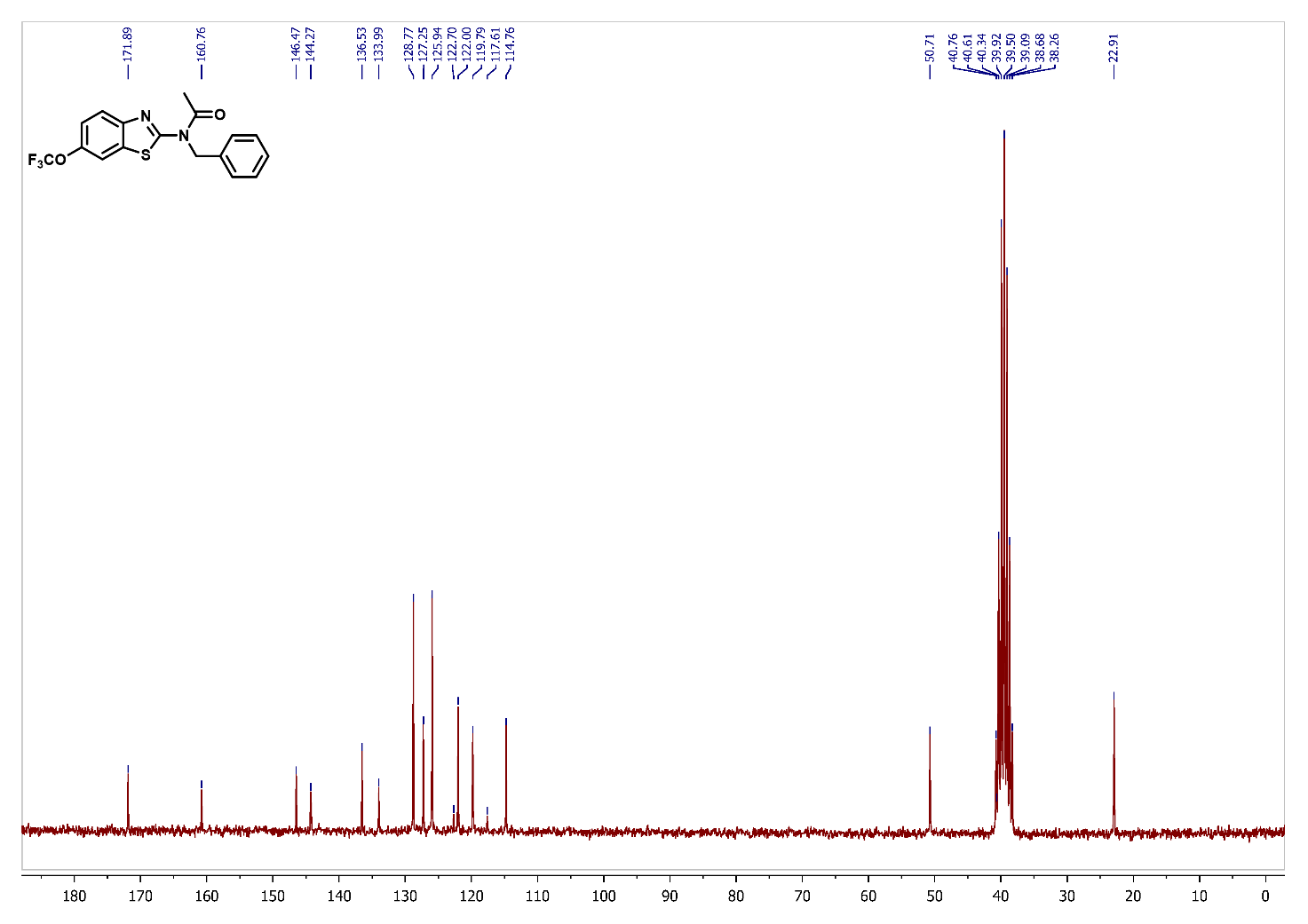
**

^1^H NMR spectrum (200 MHz, DMSO-d_6_) of compound BT-33

**
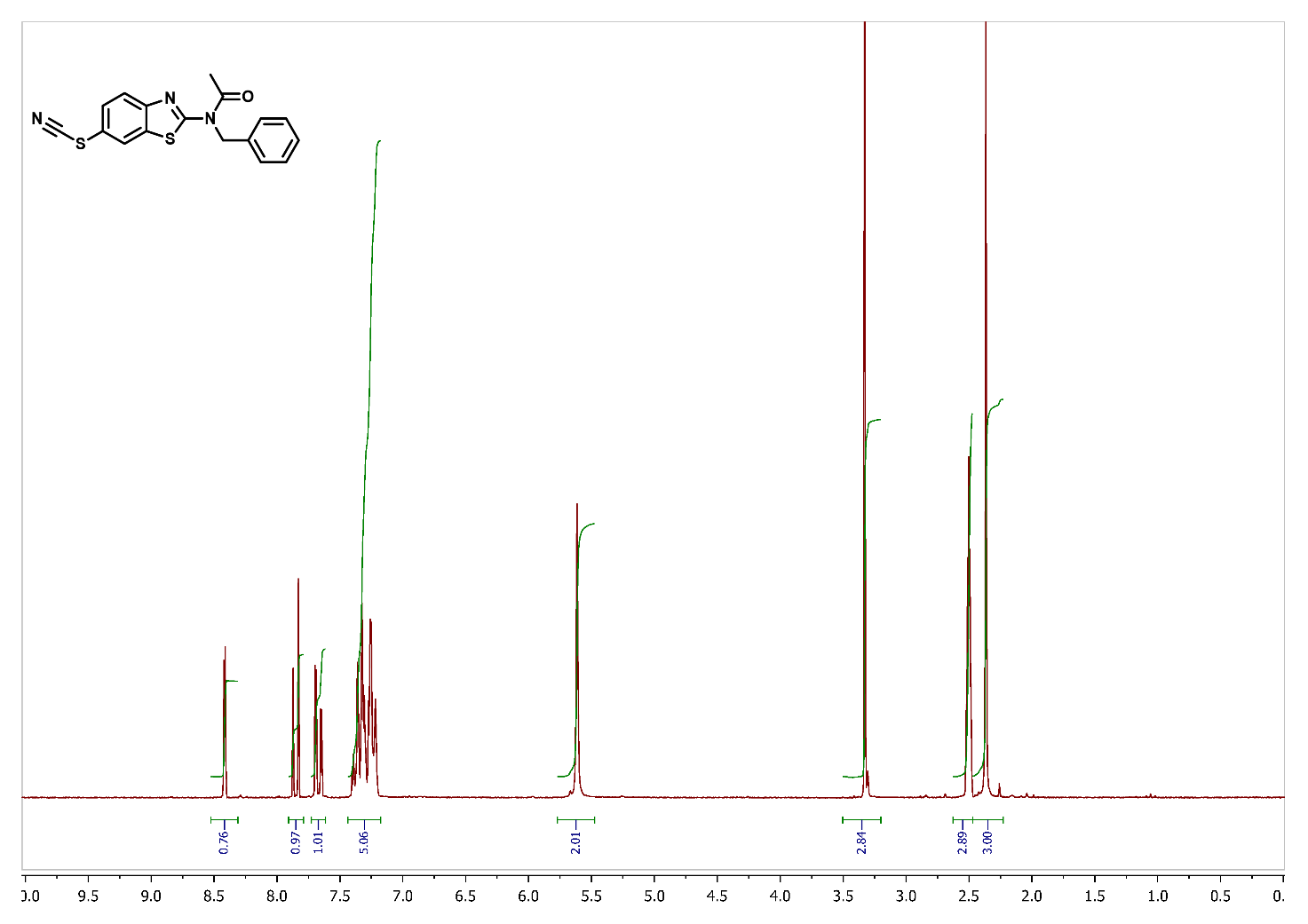
**

^13^C NMR spectrum (50 MHz, DMSO-d_6_) of compound BT-33

**
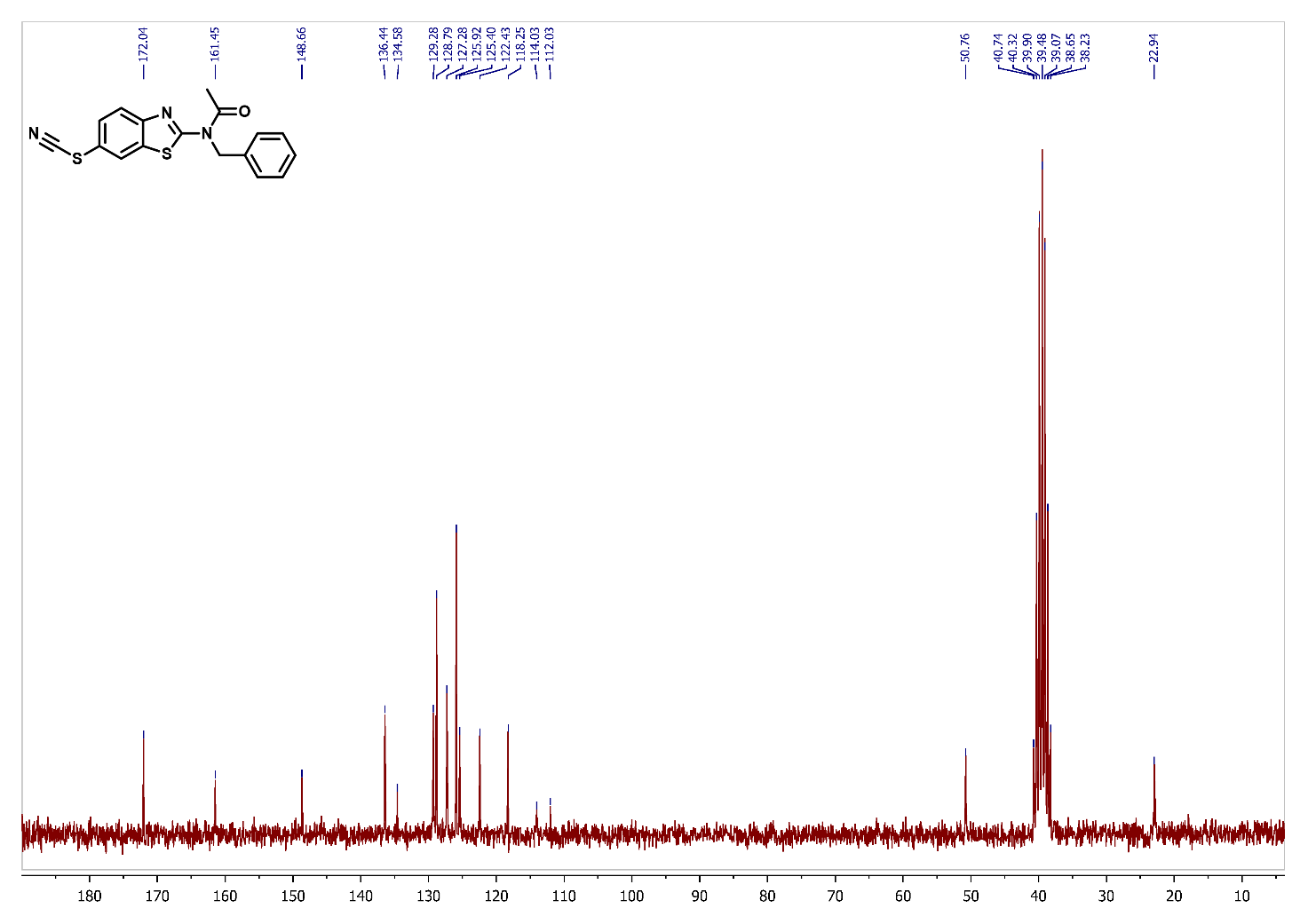
**

^1^H NMR spectrum (200 MHz, DMSO-d_6_) of compound BT-34

**
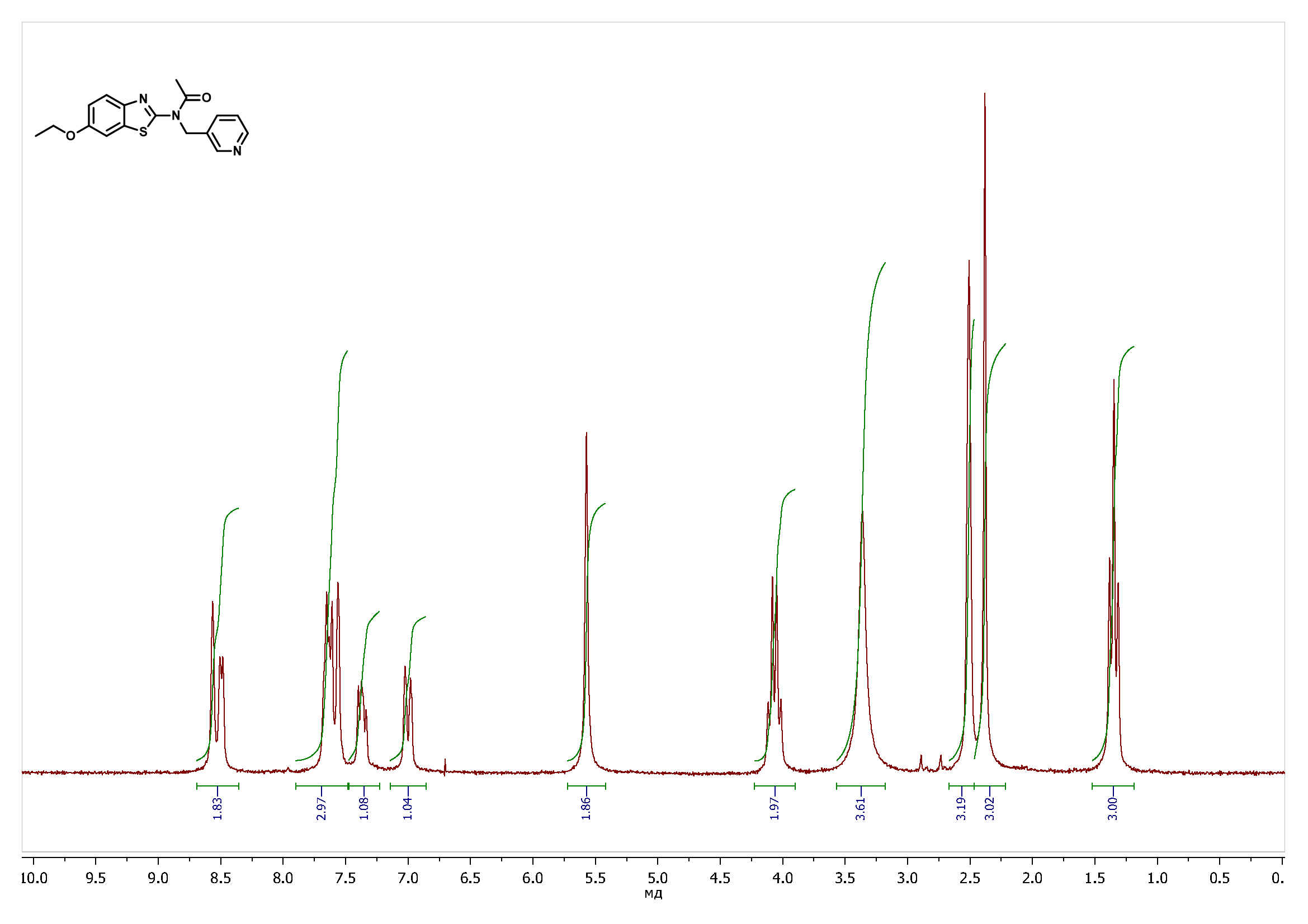
**

^13^C NMR spectrum (50 MHz, DMSO-d_6_) of compound BT-34

**
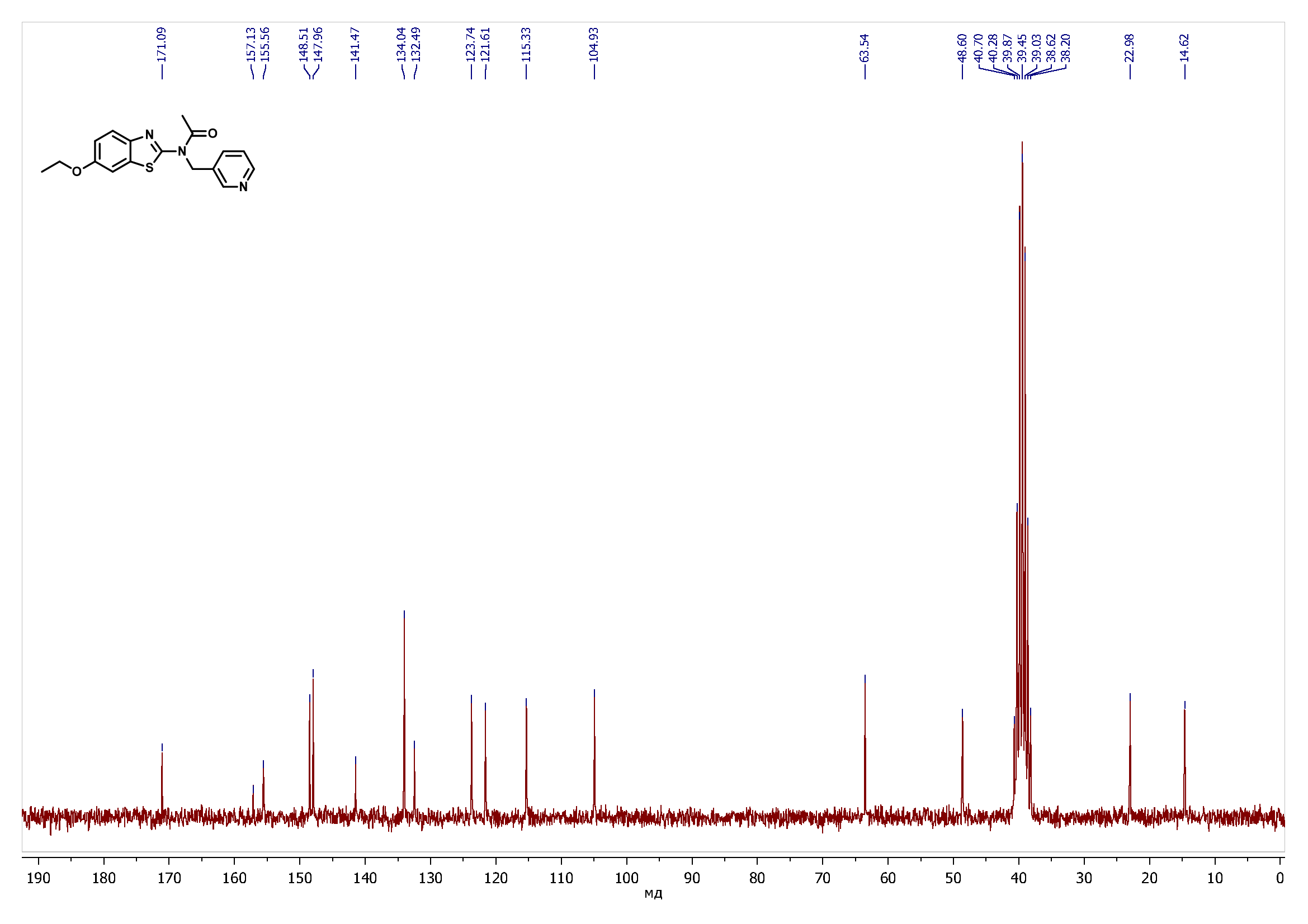
**

^1^H NMR spectrum (200 MHz, DMSO-d_6_) of compound BT-35

**
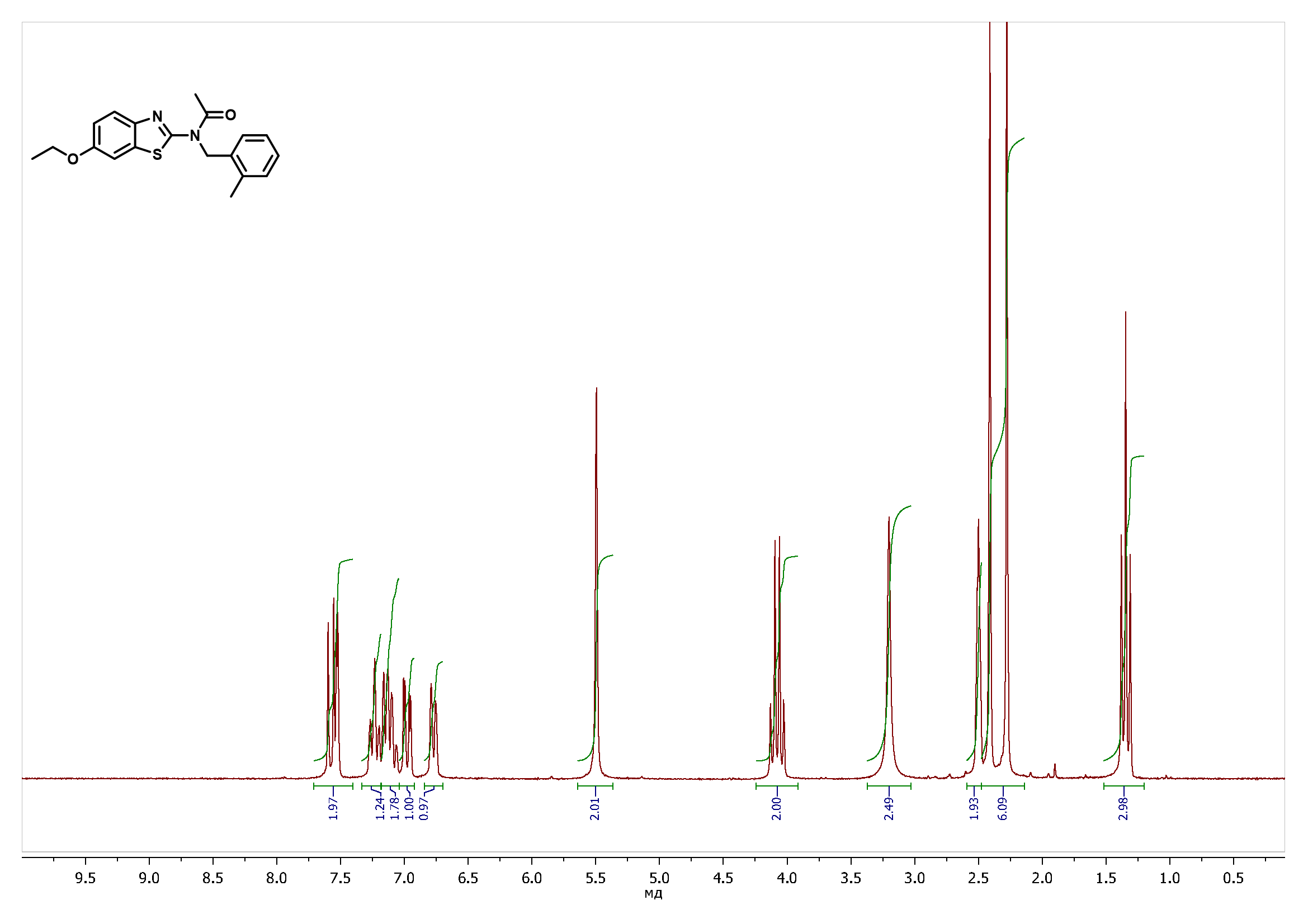
**

^13^C NMR spectrum (50 MHz, DMSO-d_6_) of compound BT-35

**
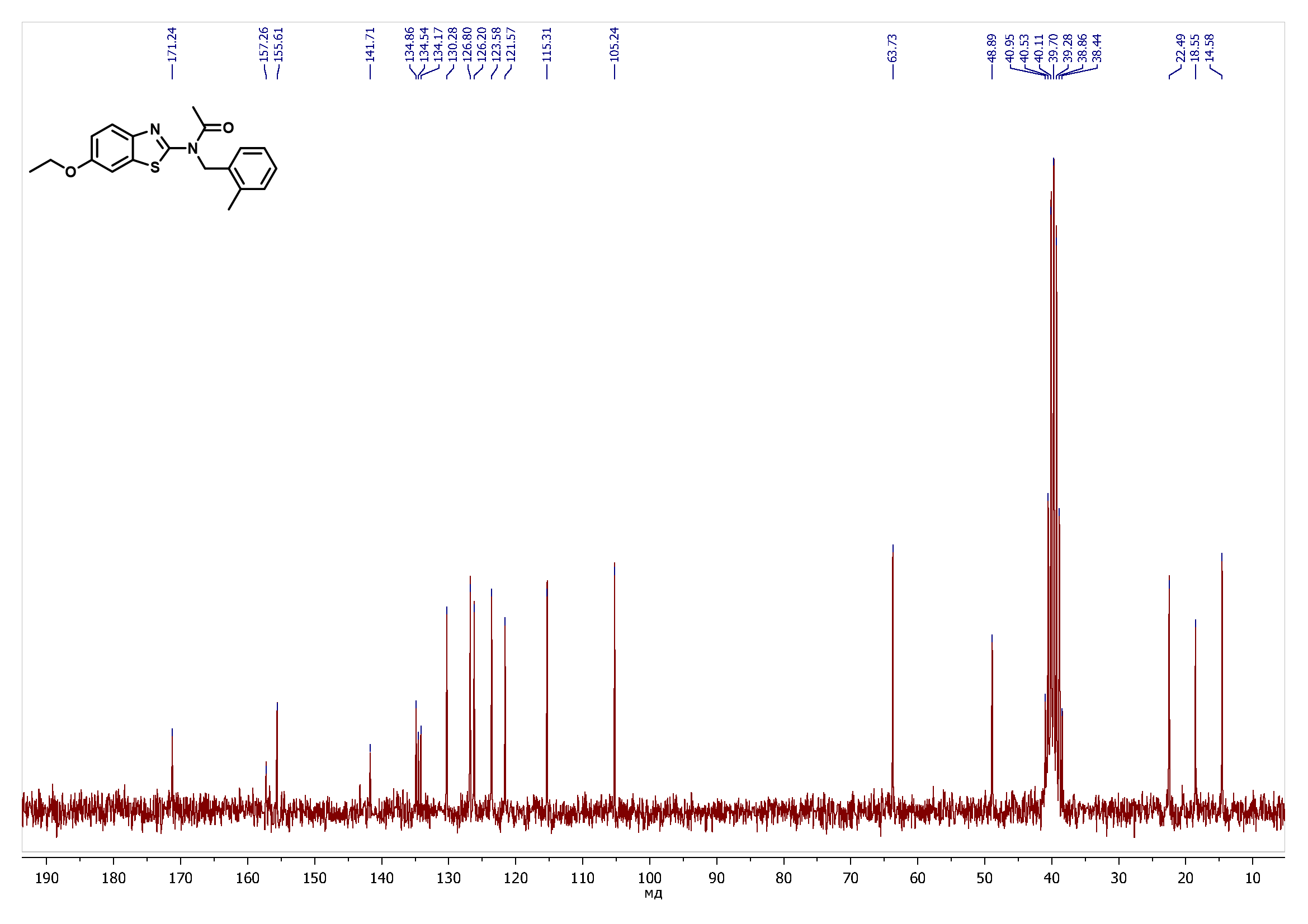
**

^1^H NMR spectrum (200 MHz, DMSO-d_6_) of compound BT-36

**
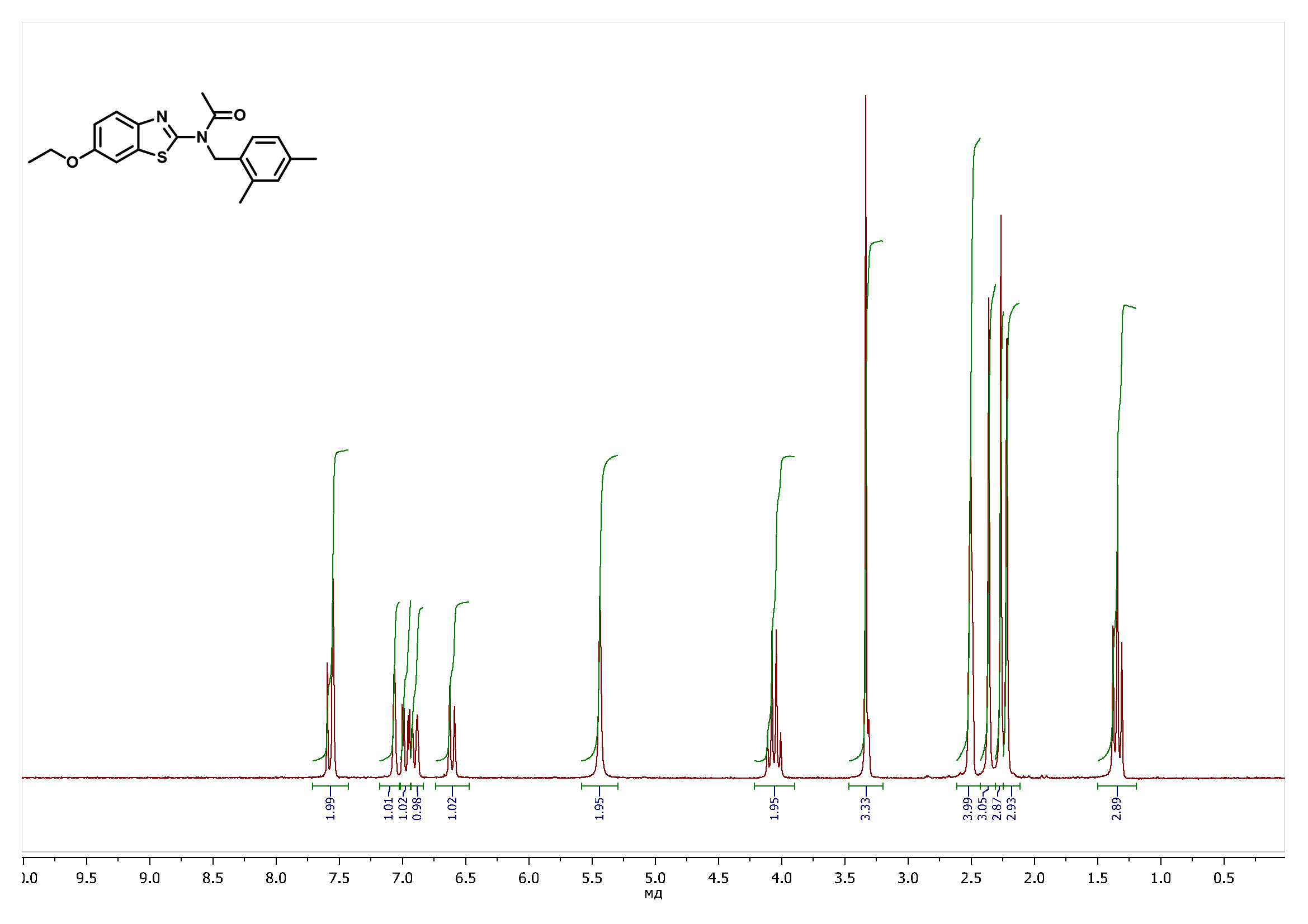
**

^13^C NMR spectrum (50 MHz, DMSO-d_6_) of compound BT-36

**
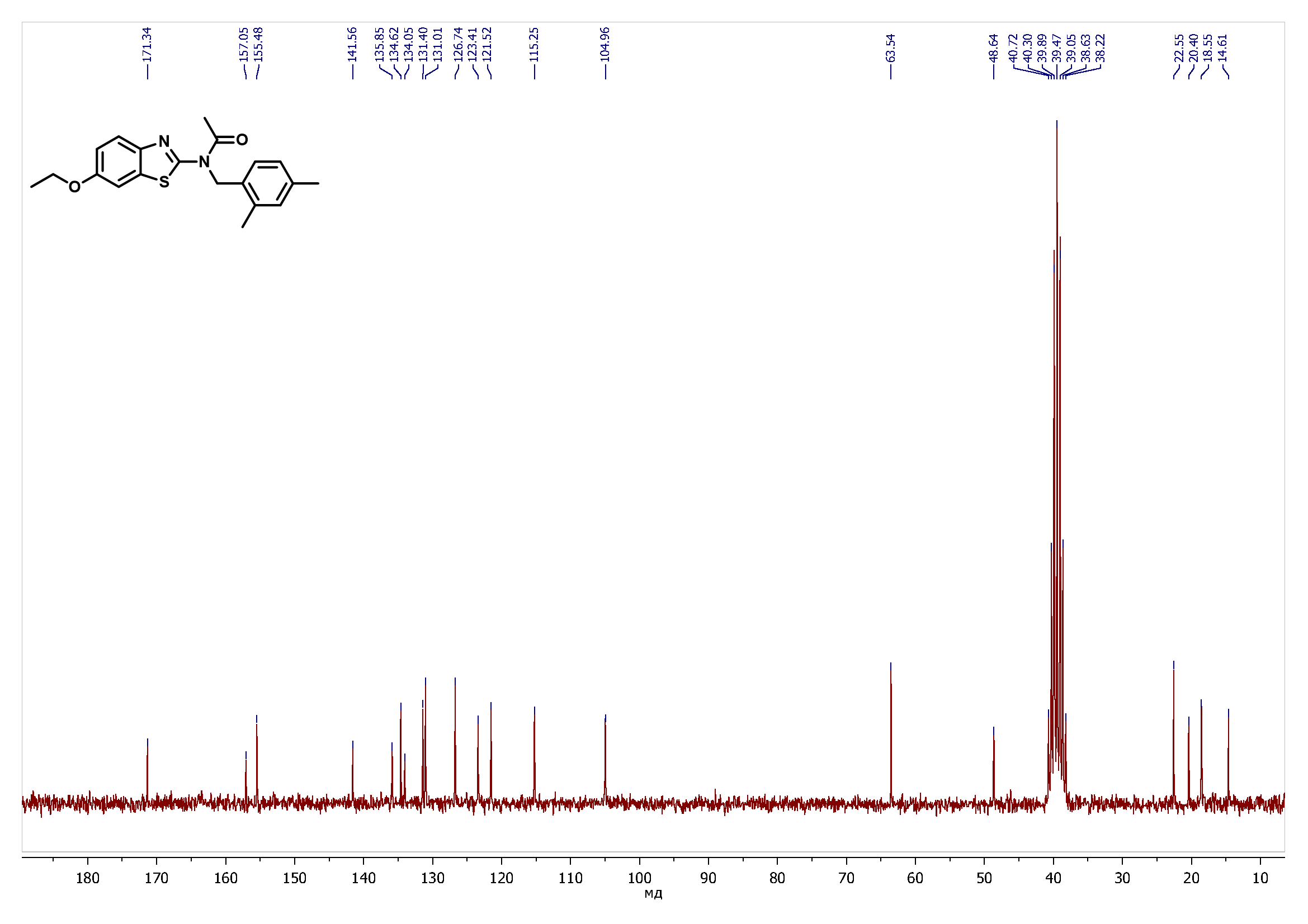
**

^1^H NMR spectrum (200 MHz, DMSO-d_6_) of compound BT-37

**
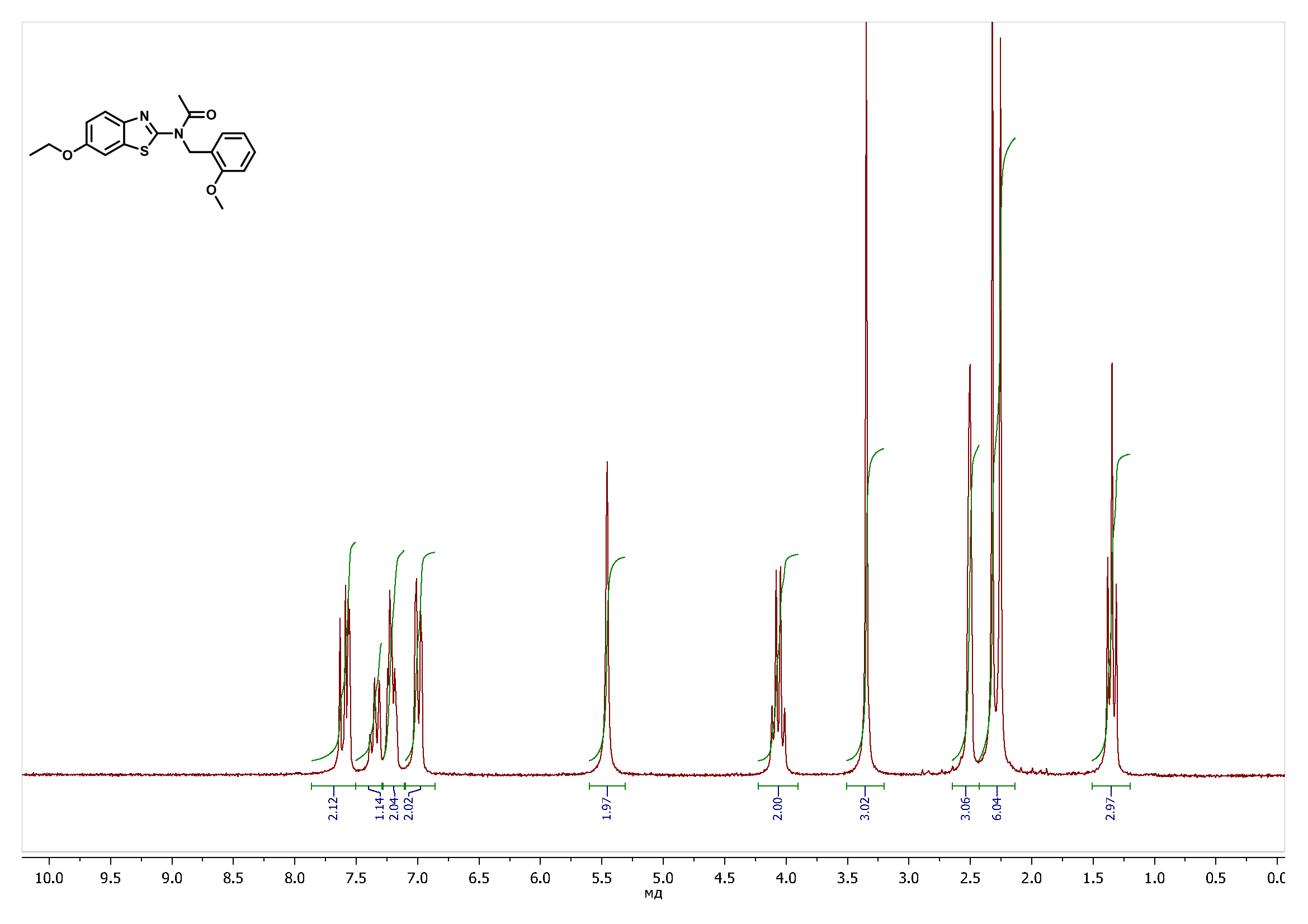
**

^13^C NMR spectrum (50 MHz, DMSO-d_6_) of compound BT-37

**
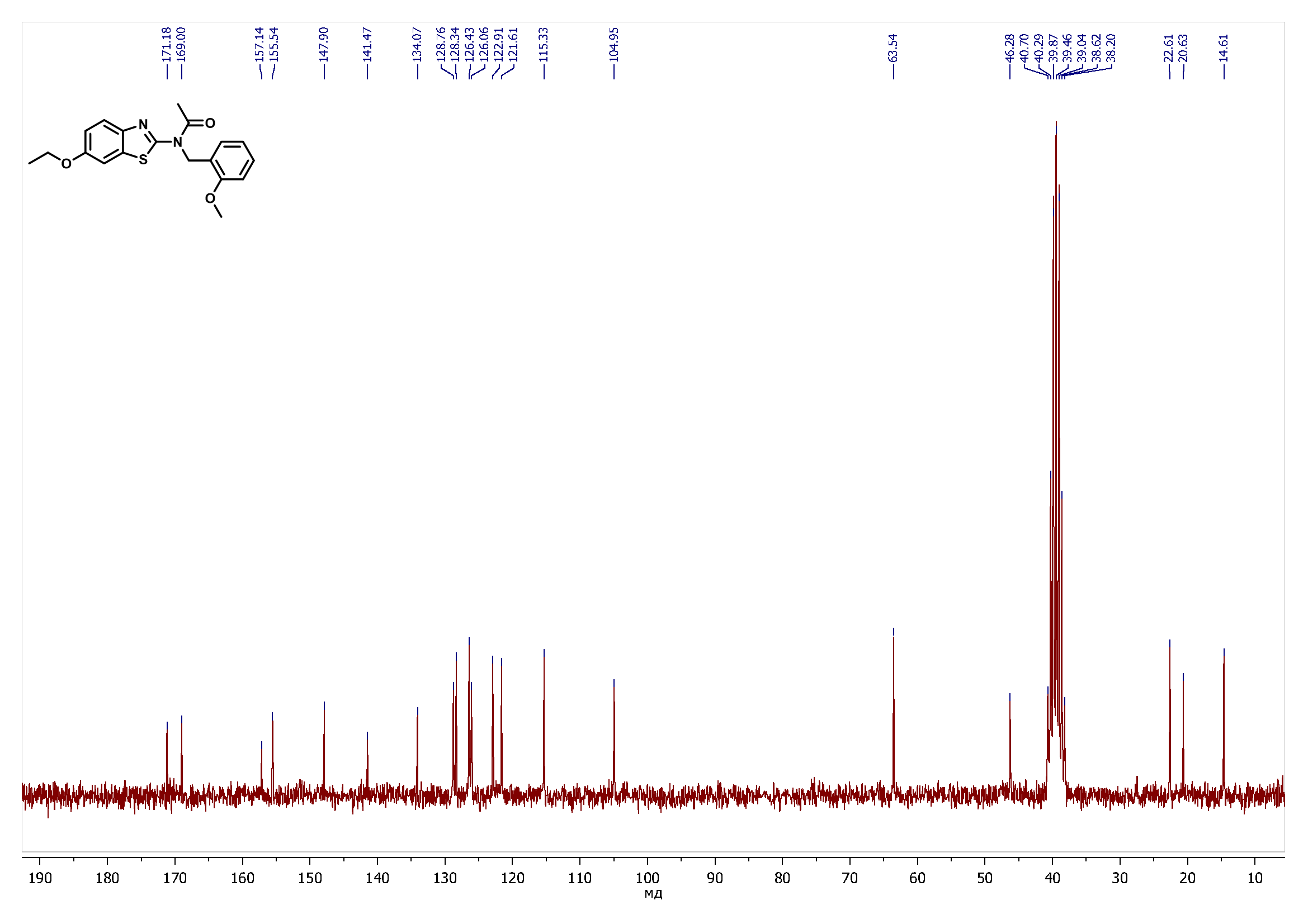
**

^1^H NMR spectrum (200 MHz, DMSO-d_6_) of compound BT-38

**
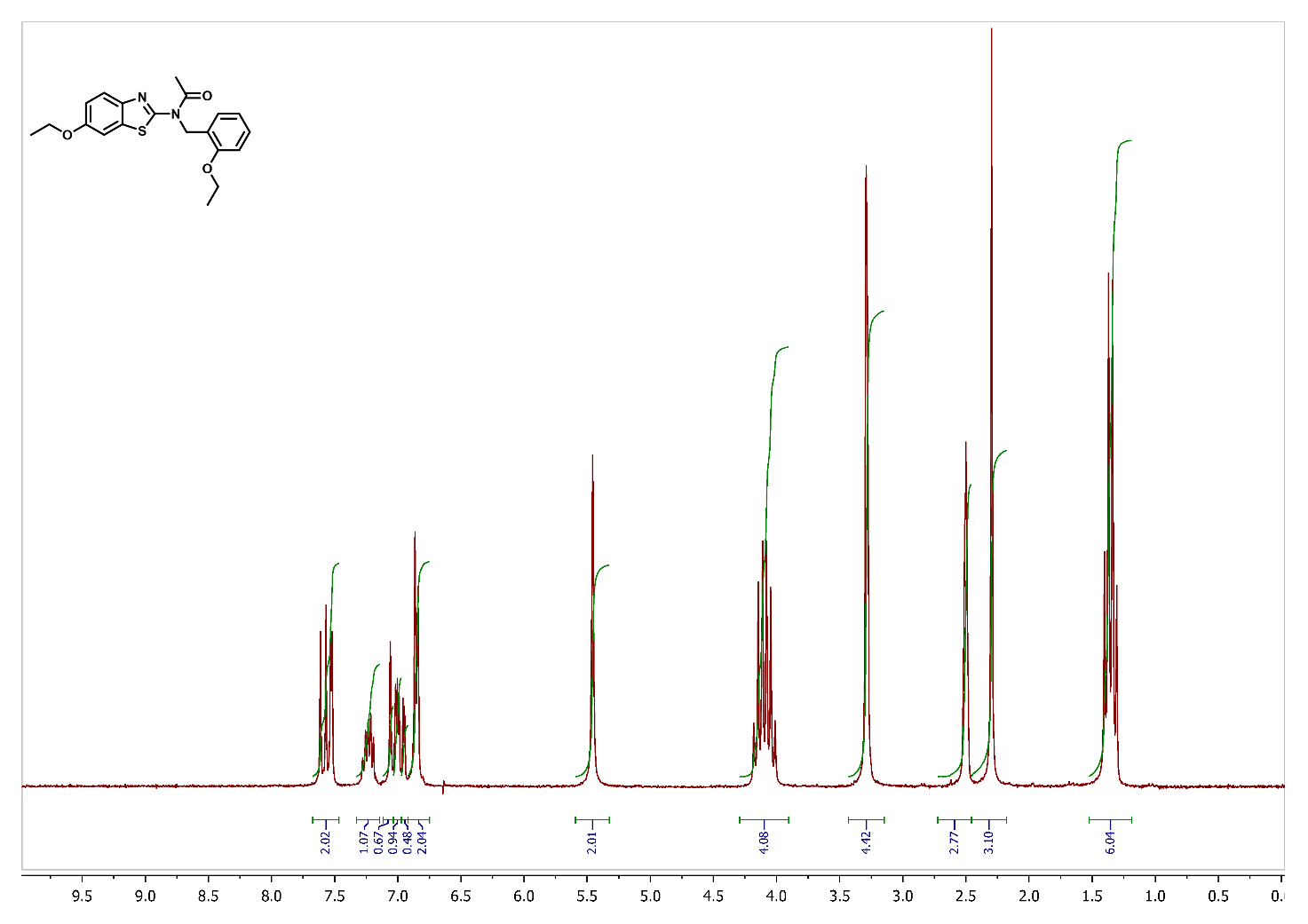
**

^13^C NMR spectrum (50 MHz, DMSO-d_6_) of compound BT-38

**
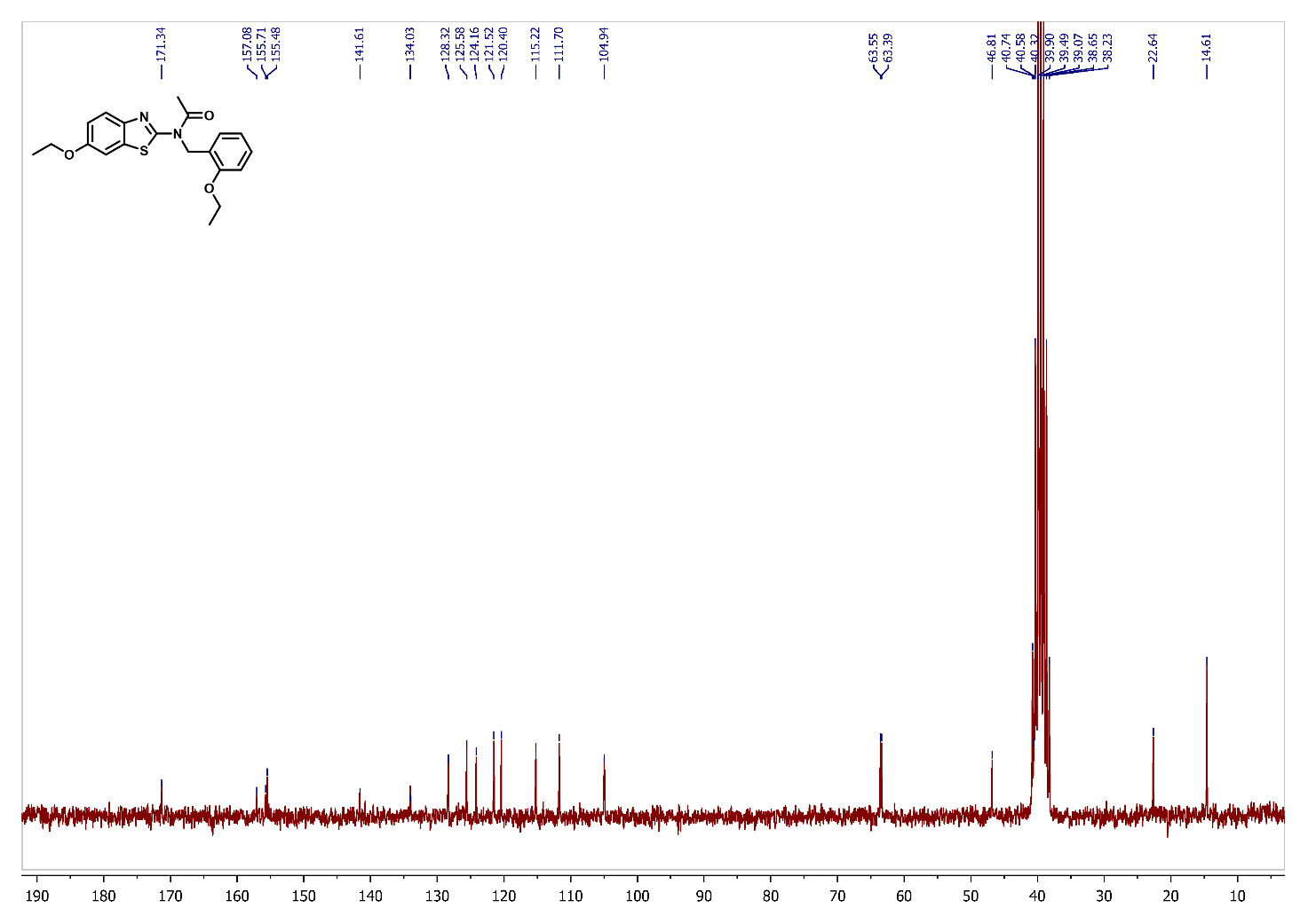
**

^1^H NMR spectrum (200 MHz, DMSO-d_6_) of compound BT-39

**
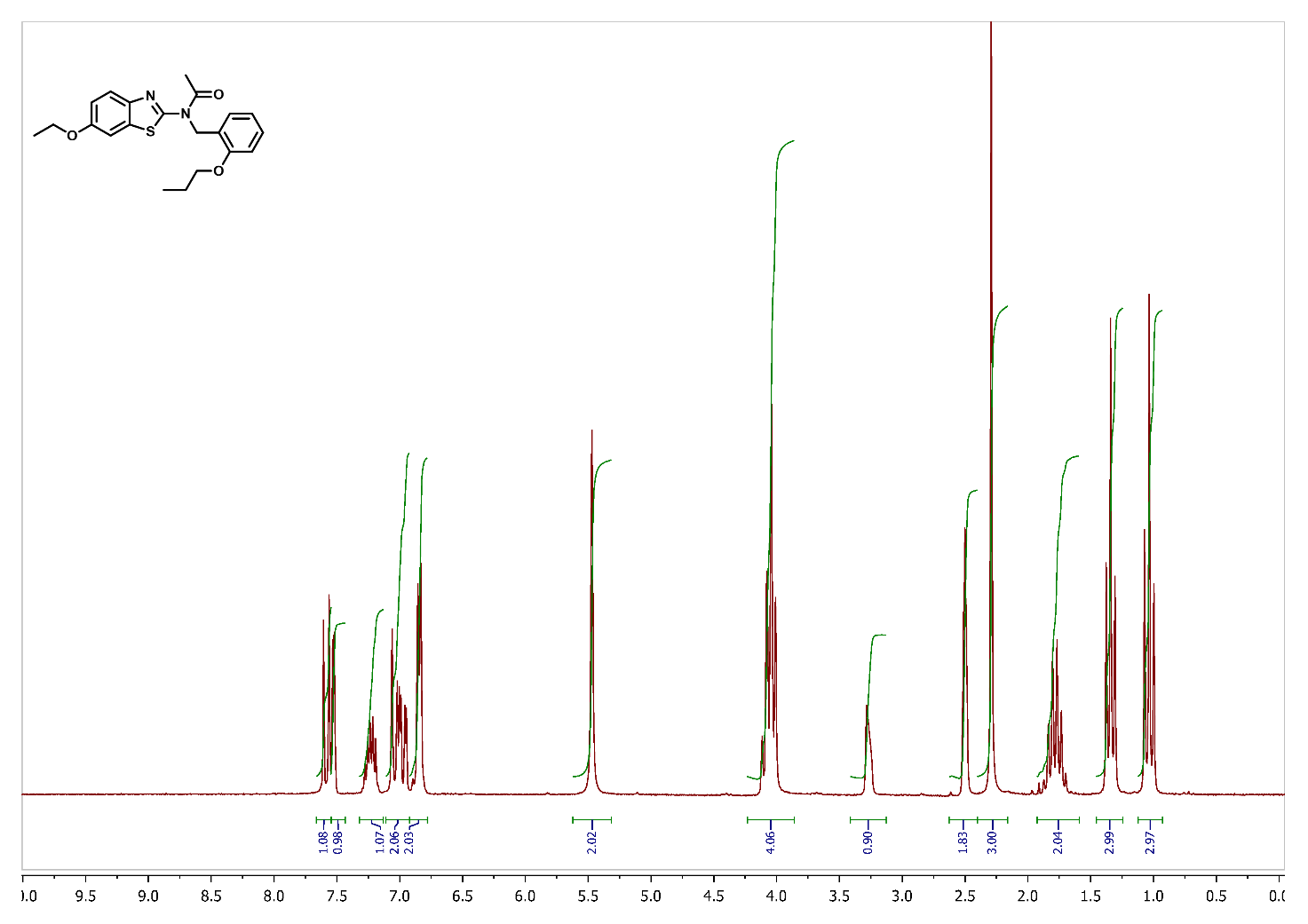
**

^13^C NMR spectrum (50 MHz, DMSO-d_6_) of compound BT-39


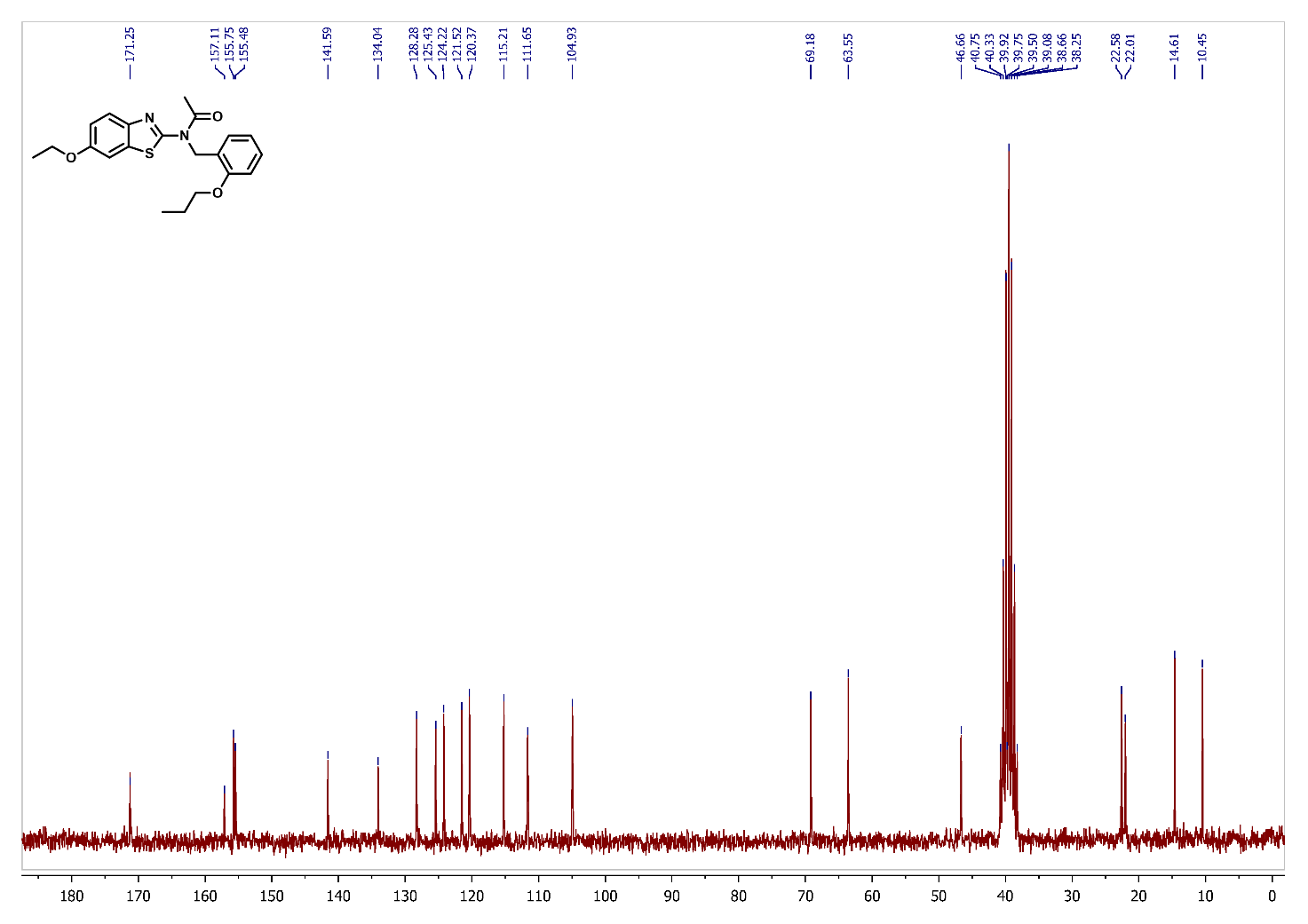


^1^H NMR spectrum (200 MHz, DMSO-d_6_) of compound BT-40

**
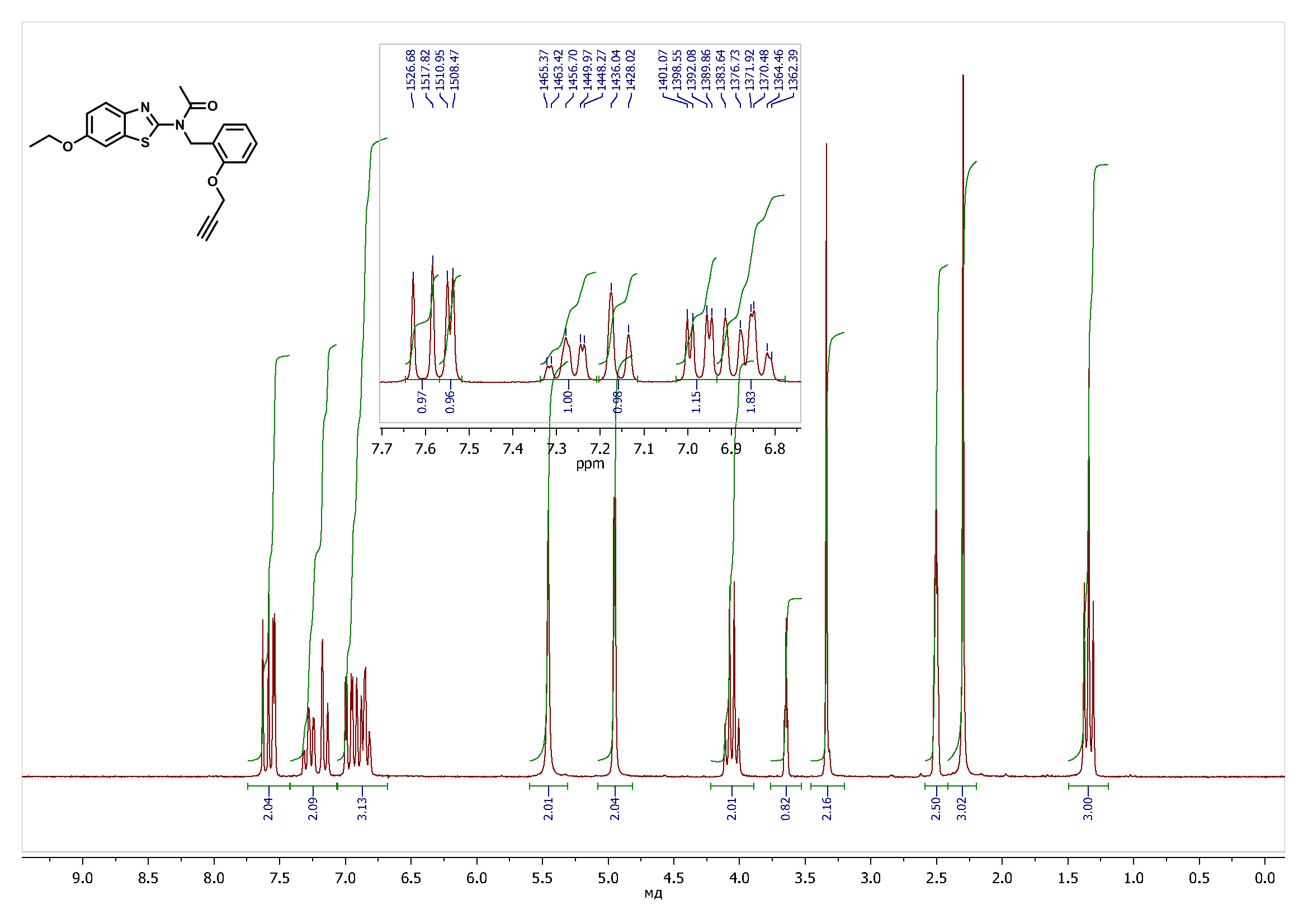
**

^13^C NMR spectrum (50 MHz, DMSO-d_6_) of compound BT-40

**
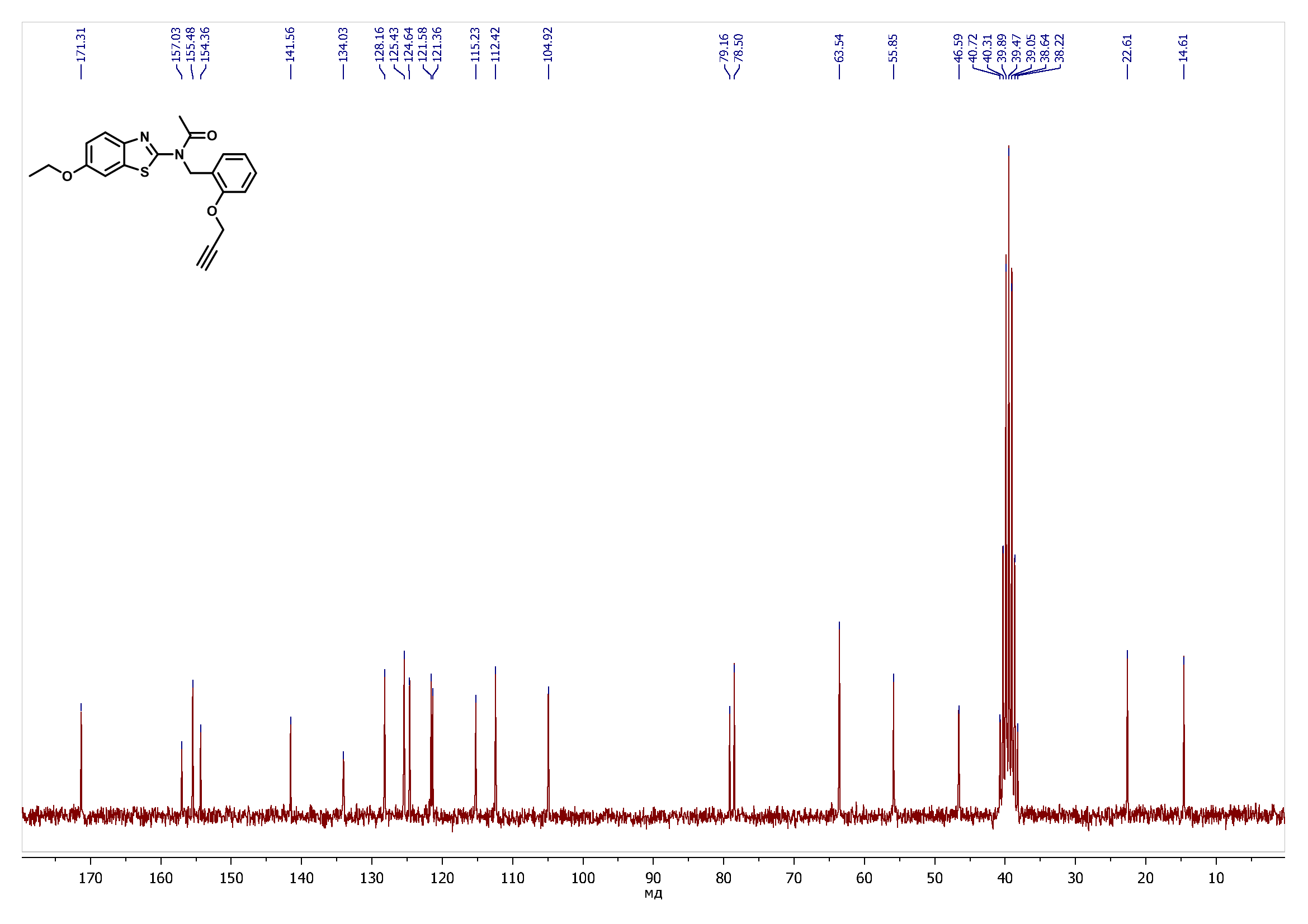
**

^1^H NMR spectrum (200 MHz, DMSO-d_6_) of compound BT-41

**
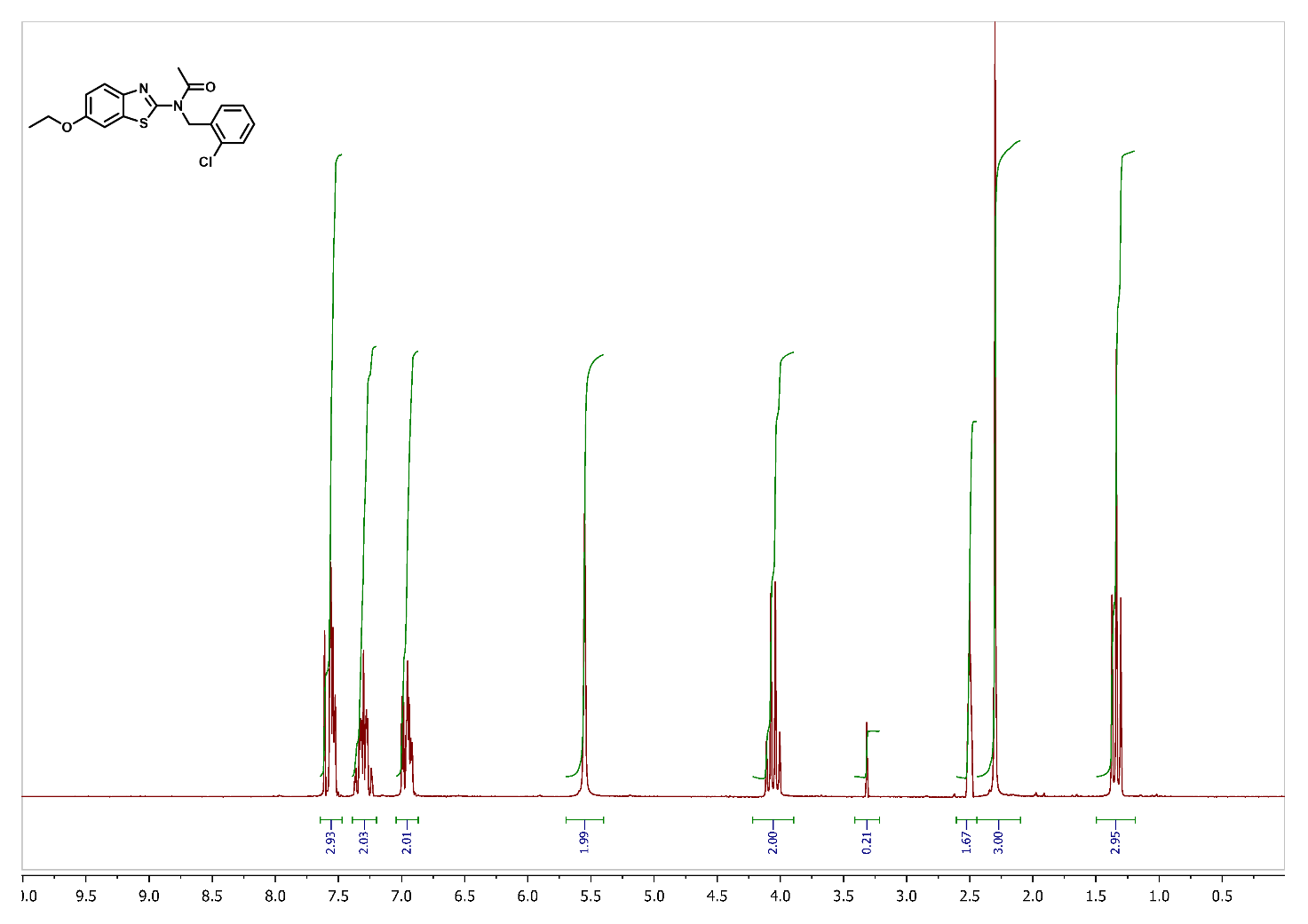
**

^13^C NMR spectrum (50 MHz, DMSO-d_6_) of compound BT-41

**
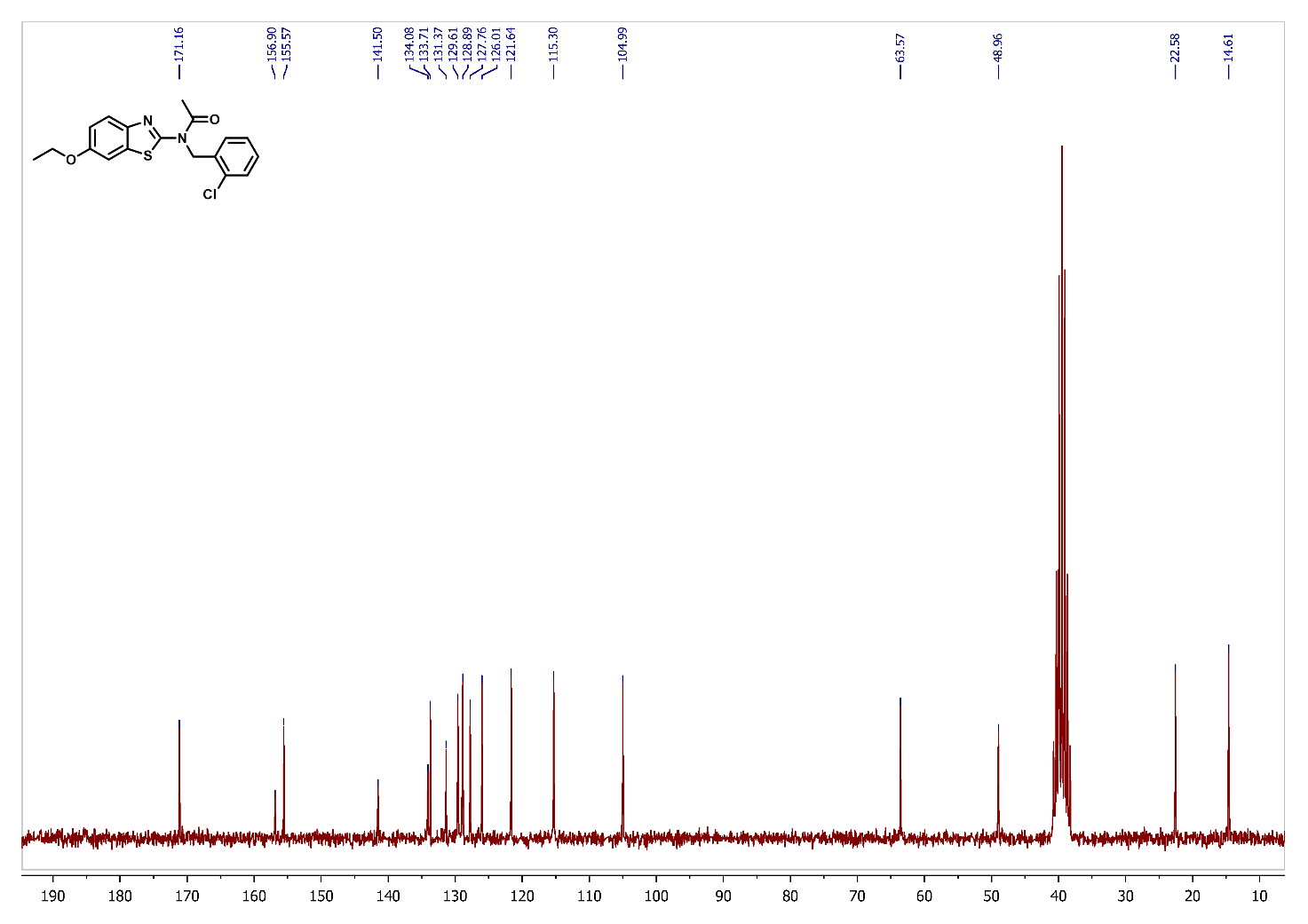
**

^1^H NMR spectrum (200 MHz, DMSO-d_6_) of compound BT-42

**
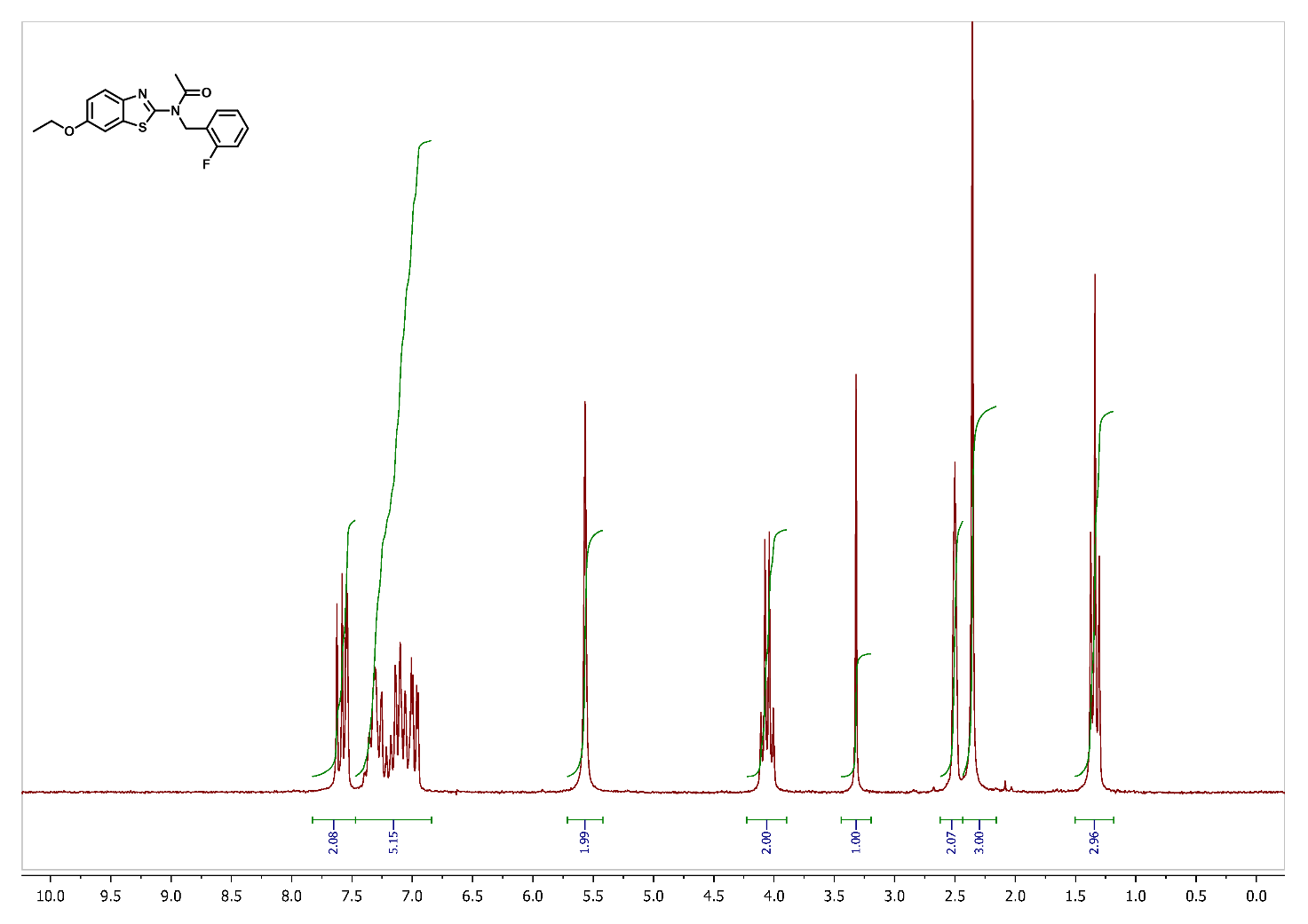
**

^13^C NMR spectrum (50 MHz, DMSO-d_6_) of compound BT-42

^1^H NMR spectrum (200 MHz, DMSO-d_6_) of compound BT-43

^13^C NMR spectrum (50 MHz, DMSO-d_6_) of compound BT-43

^1^H NMR spectrum (200 MHz, DMSO-d_6_) of compound BT-44

^13^C NMR spectrum (50 MHz, DMSO-d_6_) of compound BT-44

^1^H NMR spectrum (200 MHz, DMSO-d_6_) of compound BT-45

^13^C NMR spectrum (50 MHz, DMSO-d_6_) of compound BT-45

^1^H NMR spectrum (200 MHz, DMSO-d_6_) of compound BT-46

^13^C NMR spectrum (50 MHz, DMSO-d_6_) of compound BT-46

^1^H NMR spectrum (200 MHz, DMSO-d_6_) of compound BT-47

^13^C NMR spectrum (50 MHz, DMSO-d_6_) of compound BT-47

^1^H NMR spectrum (200 MHz, DMSO-d_6_) of compound BT-48

^13^C NMR spectrum (50 MHz, DMSO-d_6_) of compound BT-48

^1^H NMR spectrum (200 MHz, DMSO-d_6_) of compound BT-49

^13^C NMR spectrum (50 MHz, DMSO-d_6_) of compound BT-49

^1^H NMR spectrum (200 MHz, DMSO-d_6_) of compound BT-50

^13^C NMR spectrum (50 MHz, DMSO-d_6_) of compound BT-50

^1^H NMR spectrum (200 MHz, DMSO-d_6_) of compound BT-51

^13^C NMR spectrum (50 MHz, DMSO-d_6_) of compound BT-51

^1^H NMR spectrum (200 MHz, DMSO-d_6_) of compound BT-52

^13^C NMR spectrum (50 MHz, DMSO-d_6_) of compound BT-52

^1^H NMR spectrum (200 MHz, DMSO-d_6_) of compound BT-53

^13^C NMR spectrum (50 MHz, DMSO-d_6_) of compound BT-53
