# Supplementary material for: Modulating mycobacterial envelope integrity for antibiotic synergy with benzothiazoles
Source: Life Sci Alliance. 2024 May 14;7(7):e202302509. doi: 10.26508/lsa.202302509 (PMC11094368; doi:10.26508/lsa.202302509)
Supplement: Supplementary file 10 [file LSA-2023-02509_TableS9.docx]

**Table S9: Plasmids used in this work.** “Origin” denotes origin of replication, “int” denotes integrases. The genes *hyg* and *aph* confer resistance to hygromycin and kanamycin, respectively.

| **Name** | **Features** | **Reference** |
| --- | --- | --- |
| pMN016 | p_smyc_-*mspA*; c*olE1* origin; p*AL5000* origin; *hyg;* 6164 bp | (Mailaender *et al*, 2004) |
| pMN016-rv0164 | p_smyc_-*rv0164*; *colE1* origin; p*AL5000* origin; *hyg;* 5986 bp | This study |
| pMN016-mmar0407 | p_smyc_-*mmar0407*; *colE1* origin; *pAL5000* origin; *hyg;* 5962 bp | This study |
| pMN016-mmar0407_H73Y_ | p_smyc_-*mmar0407_H73Y_*; *colE1* origin; *pAL5000* origin; *hyg;* 5962 bp | This study |
| pML1357 | *colE1* origin; *xylE_m_;* p_smyc_-*gfp_m_^2+^; hyg;* Giles *int;* Giles *attP;* 6454 bp | (Huff *et al*, 2010) |
| pML1357-rv0164 | *colE1* origin; *xylE_m_;* p_smyc_-*rv0164^+^; hyg;* Giles *int;* Giles *attP;* 6204 bp | This study |
| pML1357-mmar0407 | *colE1* origin; *xylE_m_;* p_smyc_-*mmar0407; hyg;* Giles *int;* Giles *attP;* 6180 bp | This study |
| pSMT3-mspA | p_hsp60_-mspA; *pMP1* origin; *pAL5000* origin; *hyg;* 6333 bp | (Ates *et al*, 2015) |
| pSMT3-rv0164HA | p_hsp60_-*rv0164-HA*; *pMP1* origin; *pAL5000* origin; 6219 bp | This study |
| pLJR965 | L5 attP; L5 *int*; *aph;* *tetR^on^*; p_teto_-*Sth1 dCas9;* *oriE* origin; 8631 bp | (Rock *et al*, 2017) |
| pLJR965-*mmar_0407*_KD_ | L5 attP; L5 *int;* *aph;* *tetR^on^*; p_teto_-*Sth1 dCas9; sgRNA_mmar0407_*; *oriE* origin; 8629 bp | This study |
| pMS2-tdTomato | p_wmyc_-*tdTomato*; oriE(ColE1); PAL5000 origin; *hyg^R^*; 6132 bp | (Ho *et al*, 2021) |
| pTetDuo | *pAL5000* origin; *hyg*; *tetR^on^;* p_teto_-*gfp_m_^2+^*, p_smyc_*-tdtomato*, *colE1* origin, 7936bp | (Habjan *et al*, 2021) |
