# Supplementary material for: Modulating mycobacterial envelope integrity for antibiotic synergy with benzothiazoles
Source: Life Sci Alliance. 2024 May 14;7(7):e202302509. doi: 10.26508/lsa.202302509 (PMC11094368; doi:10.26508/lsa.202302509)
Supplement: Supplementary file 11 [file LSA-2023-02509_TableS10.docx]

**Table S10:** Oligonucleotides used in this study.

| **Name** | **Sequence 5’→3’** |
| --- | --- |
| E007 | ATCCGCATGCTTAATTAAGGGAGAACATGACGGCAATCTCGTGCTCACC |
| E008 | TTAATTAGCTAAAGCTTAGCTGGCCGCCAGCTGCT |
| E009 | ATCCGCATGCTTAATTAAGGGAGAACGTGCCCAGGTATGCTTCGCC |
| E010 | ATTAGCTAAAGCTTAGCCGGCGGTCAATTGTTC |
| F001 | aTgctagcatgacggcaatctcgtgctcaccgc |
| F002 | ATTCTAGATCACGCGTAGTCCGGCACGTCGTACGGGTAACTAGTGCTGGCCGCCAGCTGCTCG |
| E015 | AAACGCCAATGCCAGTTTTGAGC |
| E016 | GGGAGCTCAAAACTGGCATTGGC |
